# Transcriptional regulation of gene expression clusters in motor neurons following spinal cord injury

**DOI:** 10.1186/1471-2164-11-365

**Published:** 2010-06-09

**Authors:** Jesper Ryge, Ole Winther, Jacob Wienecke, Albin Sandelin, Ann-Charlotte Westerdahl, Hans Hultborn, Ole Kiehn

**Affiliations:** 1 Mammalian Locomotor Laboratory, Department of Neuroscience, Karolinska Institutet, Retzius väg 8, 171 77 Stockholm, Sweden; 2 Bioinformatics Centre, Department of Biology and Biotech Research and Innovation Centre (BRIC), University of Copenhagen, Ole Maaloes Vej 5, 2200 Copenhagen, Denmark; 3 DTU Informatics, Technical University of Denmark, 2800 Lyngby, Denmark, University of Copenhagen, Ole Maaloes Vej 5, 2200 Copenhagen, Denmark; 4 Department of Neuroscience and Pharmacology, University of Copenhagen, Blegdamsvej 3, 2200 Copenhagen, Denmark

## Abstract

**Background:**

Spinal cord injury leads to neurological dysfunctions affecting the motor, sensory as well as the autonomic systems. Increased excitability of motor neurons has been implicated in injury-induced spasticity, where the reappearance of self-sustained plateau potentials in the absence of modulatory inputs from the brain correlates with the development of spasticity.

**Results:**

Here we examine the dynamic transcriptional response of motor neurons to spinal cord injury as it evolves over time to unravel common gene expression patterns and their underlying regulatory mechanisms. For this we use a rat-tail-model with complete spinal cord transection causing injury-induced spasticity, where gene expression profiles are obtained from labeled motor neurons extracted with laser microdissection 0, 2, 7, 21 and 60 days post injury. Consensus clustering identifies 12 gene clusters with distinct time expression profiles. Analysis of these gene clusters identifies early immunological/inflammatory and late developmental responses as well as a regulation of genes relating to neuron excitability that support the development of motor neuron hyper-excitability and the reappearance of plateau potentials in the late phase of the injury response. Transcription factor motif analysis identifies differentially expressed transcription factors involved in the regulation of each gene cluster, shaping the expression of the identified biological processes and their associated genes underlying the changes in motor neuron excitability.

**Conclusions:**

This analysis provides important clues to the underlying mechanisms of transcriptional regulation responsible for the increased excitability observed in motor neurons in the late chronic phase of spinal cord injury suggesting alternative targets for treatment of spinal cord injury. Several transcription factors were identified as potential regulators of gene clusters containing elements related to motor neuron hyper-excitability, the manipulation of which potentially could be used to alter the transcriptional response to prevent the motor neurons from entering a state of hyper-excitability.

## Background

The mammalian central nervous system has limited capability for regeneration. Spinal cord injury therefore leads to neurological dysfunctions affecting the motor, sensory as well as the autonomic systems [1]. In the immediate phase following spinal cord injury the excitability of the motor networks caudal to the injury becomes depressed. This initial state of motor depression is often followed by a maladaptive increase in network excitability resulting in spasticity and/or pain [2-6]. The injury-induced spasticity is characterized by a disturbing hyper-reflexia causing prolonged muscle activity upon short activation of sensory afferents [7,8]. Increased excitability of spinal motor neurons, the cells that transduce the reflex response to the muscles, has been implicated in this pathophysiological state.

Under normal physiological conditions the motor output (gain) can be modulated by activation of channels in the motor neurons that conduct persistent inward currents, resulting in plateau potentials and sustained firing, leading to enhanced and prolonged muscle contraction [9-14]. The expression of plateau potentials depends on metabotropic receptor activation including activation of noradrenergic and/or serotonergic receptors. The neuromodulators that activate these receptors primarily originate from neurons located in the brainstem, which project descending fibers to the spinal cord. The ability to generate plateau potentials therefore disappears in motor neurons located caudal to a spinal cord injury [10,15-21]. They spontaneously reappear two to three weeks after injury due to chronic changes in motor neuron properties that parallel development of injury-induced spasticity [22-24]. To investigate the molecular mechanisms underlying the reappearance of plateau potentials after spinal cord injury we recently undertook a global gene expression study of motor neurons in the late phase of injury-induced spasticity [25] using the rat-tail-model with a complete spinal cord transection at the S2 segment, developed by Bennett and coworkers [6]. This work identified differential expression of genes relating to ion channels, neurotransmitter receptors and intracellular pathways 21 and 60 days post injury, supporting the observed increase in motor neuron excitability and the reappearance of plateau potentials [25]. In the present work we investigate the dynamic transcriptional response of motor neurons following spinal cord injury 0, 2, 7, 21 and 60 days post injury, enabling us to dissect out some of the regulatory mechanisms of transcription underlying the observed hyper-excitability. In the brain, such dynamic transcriptome analyses have been used to analyze the gene expression pattern of well-defined cell populations during development [26,27]. Comprehensive studies on the mechanisms of transcriptional regulation have mostly been conducted on simpler model systems with homogenous cell populations such as cell cultures [28-31]. In the present study the transcriptional response of motor neurons over time constitute a direct measure of cell-specific processes in a complex anatomical structure, allowing us in a similar fashion to examine the expression patterns and the underlying regulatory mechanisms of this response.

Cluster analysis of the gene expression time series identifies 12 time profiles reflecting combinations of early and late transcriptional regulations. Ontology analysis shows that these clusters contain groups of genes that define over-represented ontologies, indicating that each cluster profile reflects the timing of distinct biological processes as the motor neurons respond to the injury. Genes previously implicated in the development of the plateau potentials in injury-induced spasticity [32] are also identified as differentially expressed over time. The general injury response is paralleled by a response in the regulatory networks of transcription factors. Transcription factor motif analysis of the gene promoter sequences belonging to each time profile indicates a complex regulatory control of the different time profiles. Such transcription factors could prove to be potential targets for treatment of injury-induced spasticity as well as other aspects of the injury response, where experimental manipulation of their expression could be used to alter the transcriptional response of motor neurons preventing them from entering a state of hyper-excitability.

## Results

### Transcriptional response of motor neurons to injury

Spinal cord injury was inflicted by a complete spinal cord transection at the second sacral segment (S2), in effect disconnecting the spinal networks caudal to the lesion from the remaining part of the central nervous system. The injury causes complete paralysis of the tail, with no effect on bladder, bowel or hind limb functions [6,32]. The motor paralysis of the tail is followed by a slowly developing spasticity [33] in the weeks and months after injury. Clinical as well as electrophysiological evaluation of tail spasticity was performed at each time point, showing a progressive development of spasticity (or hyper-reflexia) saturating between 21 and 60 days post injury (Figure 2 in [32]).

To examine and compare the transcriptional response of the motor neurons in early and late post-injury phases GeneChip^® ^Rat Genome 230_2.0 Arrays (Affymetrix, RAT230 2 chip) were hybridized with RNA samples originating from motor neurons of uninjured control animals (n = 4) as well as animals 2 (n = 6), 7 (n = 5), 21 (n = 8) and 60 (n = 8) days post injury. A conglomerate classifier based on three well-established adjusted ANOVA test-statistics for microarray analysis (limma, Cyber-T and SAM) was used to identify significantly differentially expressed genes used for subsequent clustering, identifying 3,708 genes with a set false discovery rate (FDR) threshold of 0.02 [34].

### Consensus clustering unravels distinct gene expression time profiles

**Figure 1:**
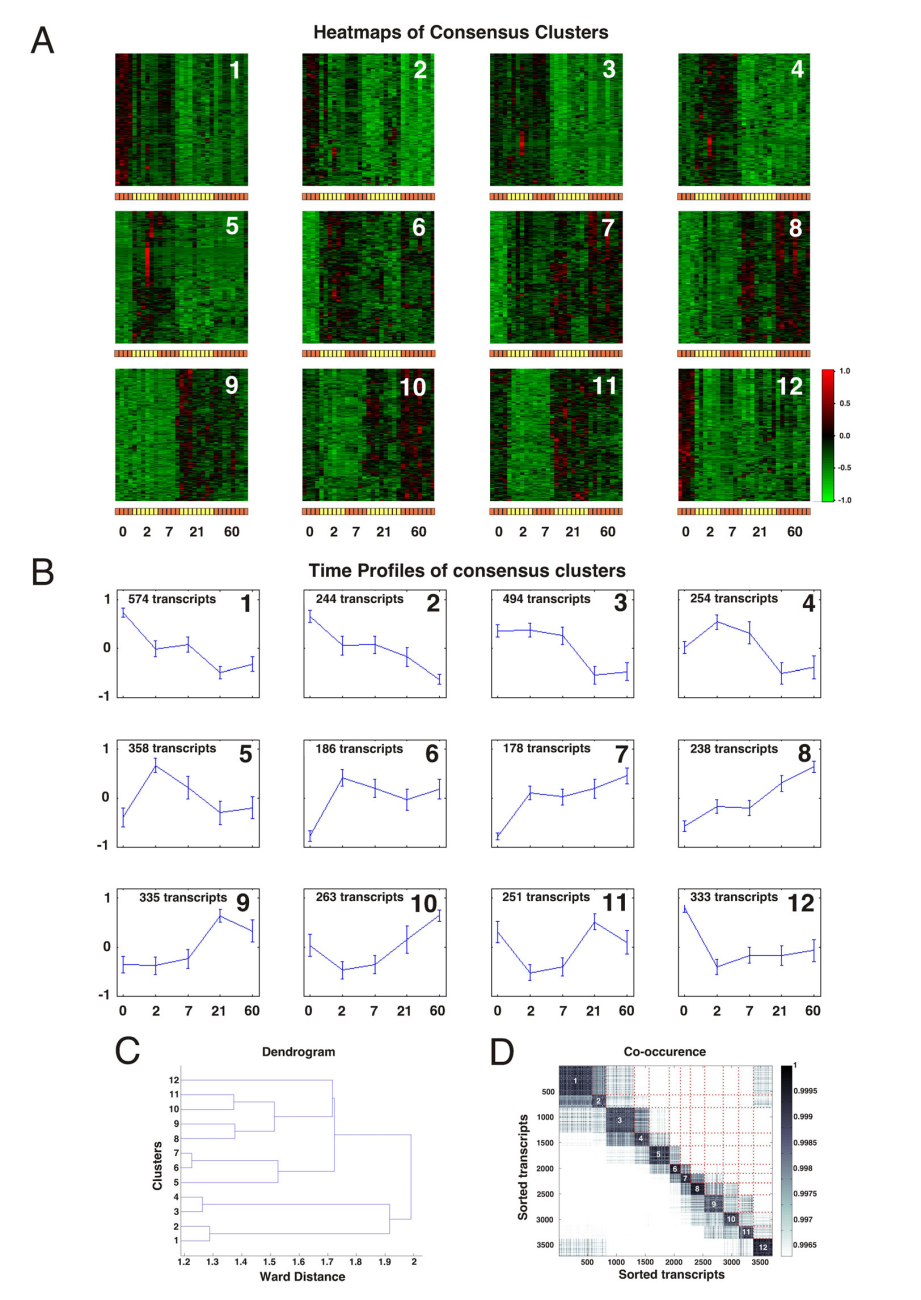
**Consensus clustering define distinct time profiles of gene expression**. Consensus clusters of the 3,708 most differentially expressed genes. **A**. Heatmaps of each consensus cluster illustrating the expression pattern of the genes contained in each cluster. The gene wise expression has been normalized according to eq. 1 resulting in expression between -1 and +1. Color Code: Red signifies up-regulation and green down-regulation compared to the gene-wise average (see color bar). The alternating colored bars below each heatmap illustrate the microarrays of each time point 0, 2, 7, 21 and 60 days post injury. **B**. The average expression time profile of each consensus cluster, plus/minus one standard deviation. **C**. Optimal leaf ordered dendrogram showing the Ward distance between each consensus cluster. **D**. Contour map of the (leaf ordered) co-occurrence matrix. The consensus clusters are indicated along the diagonal with numbers. This figure illustrates the gene overlap between clusters for the consecutive cluster runs with randomized initial settings.

In order to identify common expression profiles across time among the differentially expressed genes, transcripts were grouped into clusters of similar expression patterns using a robust consensus cluster algorithm developed by Grotkjaer et al. 2006 [35]. The consensus cluster algorithm is based on an averaging procedure conducted on multiple runs of K-means clustering (see "*Methods*"). This procedure amplifies common patterns in the expression profiles while suppressing non-reproducible features. To reduce miss-classification due to noise in the expression data (of non-differentially expressed genes) we use the consensus clustering on the most likely differentially expressed genes. With a 0.02 FDR level of significance 3,708 probe sets were included in the consensus cluster analysis. The analysis revealed the existence of 12 distinct time profiles (Figure 1A-1B) each containing 178-574 genes. Increasing the amount of consensus clusters beyond 12 did not reveal new patterns of expressions but rather breaks down the existing time profiles into sub-categories with very similar features. Heatmaps of each of the 12 consensus clusters, Figure 1A, show how their constituent genes change expression over time across all the microarrays in the study, where the expression levels for each transcript have been centered and normalized according to *eq 1*. Red color signifying expression above the average for a given transcript and green color signifies below average (see color bar). For each cluster the average level of (normalized) expression across all its constituent genes was calculated for each time point producing cluster-specific time profiles of expression, shown in Figure 1B- in the following termed cluster profiles. The similarity between cluster profiles is illustrated by the dendrogram of Figure 1C as well as in the contour map of the optimal leaf ordered co-occurrence matrix shown in Figure 1D. The co-occurrence contour map illustrates the degrees of gene overlap between the clusters in all the K-means cluster runs, where well defined squares represents very robust clusters while points falling outside these areas represents genes that occasionally fall into other clusters. The co-occurrence contour map is fairly well defined along the diagonal where overlapping genes for the most part are limited to neighboring clusters.

The expression time profiles fall into two main branches in the dendrogram, with profiles 1-4 in one branch and profiles 5-12 in the other. The first group (profiles 1-4) shares a pattern of late (21 and 60 days) down-regulation, whereas their early response varies (2 and 7 days). In the other group (profiles 5-12), all but profile 12 are characterized by an up-regulation either early (profile 5), late (profiles 8-10) or both (profiles 6-7). Profiles 10 and 11 have an early down-regulation followed by a subsequent up-regulation. Cluster profile 12 is characterized by a very substantial early down-regulation followed by a slight progressive increase in expression that remains below control levels. This makes it somewhat similar to cluster profiles 10 and 11, though as it remains below control levels even in the late phase its biological classification rather belongs to the group of down-regulated cluster profiles 1-4. The co-occurrence plot, Figure 1D, also illustrates this fact, where genes of profile 12 occasionally overlap with cluster profiles 1 and 2 as well as with cluster profiles 10 and 11. The full list of differentially expressed genes grouped according to their cluster identification is provided in Additional file 1.

The consensus clustering of the differentially expressed genes thus unravels gene clusters with distinct expression time profiles. We used these clusters for ontology analysis as well as to ascribe expression profiles to genes related to motor neuron excitability changes. Analysis of transcription factor regulation was performed on each cluster to identify potential mechanisms of regulation for the identified genes and ontologies.

### Ontology analysis of gene expression time profiles

Genes may be annotated according to well-defined ontologies such as biological processes, cellular location, biochemical pathways, protein families etc. highlighting different aspect of their function. Over-representation of genes within a cluster profile that share an ontology term strongly suggests that the cluster represents a set of genes that engage in or represent the identified ontology [36]. The ontology database maintained by the gene ontology (GO) consortium annotating genes according to biological processes (BP), molecular functions (MF) and cellular compartments (CC) as well as INTERPRO and KEGG pathways were all used in this analysis. Each ontology highlights a different aspect of gene functions, making it desirable to group together over-represented ontologies containing a predefined degree of gene overlap in order to fully appreciate the functional role of each of the 12 time profiles. Furthermore, since gene ontologies are structured as acyclic directed graphs where a gene is annotated from its most descriptive (lowest) level all the way up through the graph, it may also be desirable to group together the annotation terms within each branch in the ontology graph that share the same genes and within these extract the most representative ontology (the lowest level) to exclude redundant representations. DAVID functional ontology clustering accomplishes this task. The ontology analysis was run on each time profile with the total set of differentially expressed genes as background. The most representative term of each ontology cluster with a p-value below 0.03 was extracted, shown in Table 1. It is clear from this analysis that profiles 1-4 and 12, all signified by a late down-regulation, have several overlapping terms. Among the over-represented ontologies defined by the down-regulated genes contained in each of these clusters, we find "cell-cell adhesion" and "zinc finger" shared between profiles 1 and 3, ontologies relating to ribosomal processes shared in profiles 2 and 3, and ontologies relating to metabolic processes shared in profiles 3 and 4. Profile 12 contains genes that seem to be involved in mitochondrial energy production (ATP) and regulation of anion concentrations and signaling. There are some terms in cluster profiles 1-4 that also seem to indicate a down-regulation of mitochondrial processes and overall metabolism. The profiles signified by an up-regulation of transcripts somewhere along the time profile (cluster profiles 5-11) engage in different processes from those observed for cluster profiles 1-4 and 12. Profile 5 represents an early response of up-regulated genes primarily seen two days post injury involving processes of immunological and inflammatory responses. Profiles 6 and 7, though being similar in their expression pattern, have little overlap in their over-represented ontologies. Profile 6 seems to reflect activation of a broad repertoire of kinase signaling pathways as well as modification of the ribosomal machinery, which might correlate with the only over-represented term in profile 7, indicating a positive regulation of the transcriptional machinery relating to polymerase II. Profiles 8-11 all seem to contain ontologies relating to membrane bound activities such as active transport and neuronal signaling as well ontologies pertaining to engagement into neuronal developmental processes.

**Table 1:** Over-represented ontologies of each gene cluster

Ontology Term	Class	Count	Total	Terms
**Cluster 1**: 574 transcripts				

Cell-cell adhesion	BP	18	34	9
Ensheathment of neurons	BP	7	7	4
Zinc finger, C2H2-type	INTERPRO	11	11	4
L-amino acid transmembrane transporter activity	MF	7	9	8
Solute:cation symporter activity	MF	6	9	6
Nucleosome assembly	BP	6	14	10
				
**Cluster 2**: 244 transcripts				

Ribonucleoprotein complex	CC	31	54	16
Translation	BP	23	54	16
Intracellular part	CC	143	162	8
RNA splicing	BP	9	14	9
Ribonucleoprotein complex biogenesis	BP	10	14	4
Macromolecule metabolic process	BP	95	110	4
Mitochondrial ribosome	CC	4	4	4
Regulation of apoptosis	BP	16	37	12
				
**Cluster 3**: 494 transcripts				

Cell-cell adhesion	BP	22	44	11
Metal ion binding	MF	97	98	4
Cellular metabolic process	BP	198	217	4
Regulation of biosynthetic process	BP	12	19	4
Macromolecule biosynthetic process	BP	37	52	4
Ribosome	KEGG	12	38	9
Cell part	CC	294	294	8
Peptidase M, neutral zinc metallopeptidase, zinc binding	INTERPRO	5	8	4
Zinc finger, C2H2-type	INTERPRO	10	10	4
Ligase activity, forming carbon-nitrogen bonds	MF	13	19	6
Cytosolic large ribosomal subunit	CC	6	11	5
				
**Cluster 4**: 254 transcripts				

RNA metabolic process	BP	43	85	14
Cellular metabolic process	BP	108	117	4
Monooxygenase activity	MF	5	9	7
				
**Cluster 5**: 358 transcripts				

Inflammatory response	BP	21	50	4
Adaptive immune response	BP	8	12	16
Pancreatitis-associated protein	INTERPRO	4	7	6
Chemokine activity	MF	5	10	10
Ras	INTERPRO	10	20	8
GTP binding	MF	19	19	8
Biopolymer modification	BP	5	6	5
Immunoglobulin subtype	INTERPRO	9	12	8
Cytokine production	BP	6	10	8
				
**Cluster 6**: 186 transcripts				

Glycosyltransferase	PIR	8	10	12
Ribonucleotide binding	MF	32	37	8
Contractile fiber part	CC	4	4	4
Ras	INTERPRO	6	10	8
Tyrosine-specific protein kinase	PIR	4	10	9
Kinase activity	MF	18	25	19
Hydrolase activity, hydrolysing O-glycosyl compounds	MF	5	9	4
Biopolymer modification	BP	28	60	6
				
**Cluster 7**: 178 transcripts				

Positive regulation of transcription from RNA polymerase II	BP	7	45	25
				
**Cluster 8**: 238 transcripts				

EGF-like	INTERPRO	7	7	10
Anatomical structure development	BP	43	66	6
Membrane part	CC	75	75	4
Transporter activity	MF	32	35	8
Transcription coactivator activity	MF	8	14	4
Intracellular transport	BP	23	26	8
Integral to endoplasmic reticulum membrane	CC	5	8	7
Coated vesicle	CC	9	11	6
				
**Cluster 9**: 335 transcripts				

Protein amino acid phosphorylation	BP	28	64	14
Plasma membrane part	CC	31	53	4
Protein kinase, core	INTERPRO	16	22	6
Immunoglobulin-like	INTERPRO	13	13	5
Neurotransmitter transporter activity	MF	5	12	14
System development	BP	52	72	5
Gated channel activity	MF	13	28	28
Neurological system process	BP	29	30	4
ATP binding	MF	37	45	8
Cation transmembrane transporter activity	MF	19	39	8
Negative regulation of fibroblast proliferation	BP	4	10	11
Axogenesis	BP	9	18	14
Regulation of neurotransmitter levels	BP	9	14	6
				
**Cluster 10**: 263 transcripts				

Membrane part	CC	98	111	4
Glycolysis	BP	13	37	25
Transporter activity	MF	40	40	6
Purine ribonucleotide binding	MF	45	48	9
Positive regulation of nucleobase, nucleoside, nucleotide	BP	13	15	5
Synaptic transmission	BP	17	31	5
Phosphorylation activity	BP	24	49	25
Active transmembrane transporter activity	MF	16	31	47
Phosphorylation activity	MF	22	35	6
Neuron differentation	BP	14	20	14
Cell development	BP	34	37	11
Monovalent inorganic cation homeostasis	BP	5	12	10
Amine transport	BP	7	12	4
Ion exchanger activity	BP	10	10	11
				
**Cluster 11**: 251 transcripts				

Calycin	INTERPRO	10	10	6
Synaptic transmission	BP	18	27	5
DNA repair	BP	10	21	4
Neuron projection development	BP	11	24	17
Developmental process	BP	61	61	4
Glycealdehyde-3-phosphate dehydrogenase ()	MF	5	20	21
Activation of adenylatecyclase activity by G-protein signaling pathway	BP	4	9	7
				
**Cluster 12**: 333 transcripts				

Mitochondrial ATP synthesis coupled electron transport	BP	10	19	9
Mitochondrial part	CC	31	44	8
Oxidative phosphorylation	KEGG	21	28	5
Primary active transmembrane transporter activity	MF	15	26	13
Tricarboxylic acid cycle	BP	6	9	11
Ion transmembrane transporter activity	MF	23	37	10
ATPase activity, coupled to transmembrane movement of substances	MF	12	23	39
Alkali metal ion binding (K^+^)	MF	8	14	23
Anion channel activity	MF	5	14	23

*Class Ontology*: MF = molecular function, BP = biological process, CC = cellular component, PIR = protein information resource. *Count*: Number of genes in ontology. *Total*: Number of genes in ontology cluster. *Terms*: number of ontology terms in ontology cluster.

In conclusion, the ontology analysis ascribes several general functions to each time profile identifying their timing across the injury response and suggesting a common regulatory control of these.

### Differential expression of genes affecting motor neuron excitability

While the ontology analysis may identify general terms subject to regulation, a manifest focus of our study was to correlate changes in gene expression that can be linked to increased motor neuron excitability and injury-induced spasticity. In a previous study we examined the late (21 and 60 days post-injury) transcriptional response of motor neurons to their sham-operated counterpart [32]. That study focused on changes in three main categories: ion channels, receptors of neurotransmitters and intracellular pathways capable of modulating these. Here we extract the same categories of significantly differentially expressed genes. These genes are shown in Table 2 along with the cluster identity. The majority of these differentially expressed genes are identical to the genes reported in our previous study [32], with the two primary differences being that in the present case they are, 1) identified based on their differential expression across time as opposed to a static comparison to their sham-operated counterpart at each time point and 2) each gene is associated with a pattern of expression over time. Most importantly, we thus identify the same gene candidates subject to regulation with two different strategies for the choice of reference, supporting the robustness of our findings. But, in contrast to the static study (25), the dynamic response over time now enables us to expand our analysis and start unraveling some of the underlying regulatory mechanism shaping the observed patterns of gene expression within each cluster profile.

**Table 2:** Differentially expressed genes relating to motor neuron excitability

Probe IDs	Gene ID	Protein ID	Cluster
**Calcium Channels**			

1371039_at	*Cacnb4*	CAB4	4

1368398_at	*Cacna1h*	Cav3.2	8

1371175_a_at	*Cacna1b*	Cav2.2	9

1369706_at	*Cacng1*	Cacng1	10

1386939_a_at	*Cacna1a*	Cav2.1	11

**Sodim Channels**			

1379307_at	*Sap1*	SAP1	1

1369662_at	*Scn2a1*	Nav1.2	3

1368539_at	*Scn9a*	Nav1.7	5

1383435_at	*Scn3b*	SCN3B	5

1370850_at	*Scn3b*	SCN3B	5

1387010_s_at	*Scn1b*	SCN1B	8

1368351_at	*Scn10a*	Nav1.8	8

1388035_a_at	*Scn5a*	Nav1.5	9

**Potassium Channels**			

1370439_a_at	*Kcnc2*	Kv3.2	1

1369043_at	*Kcna4*	Kv1.4	2

1386770_x_at	*Kcne2*	KCNE2	2

1385226_at	*Kctd11*	KCD11	3

1387264_at	*Kcnk6*	TASK-1	5

1370958_at	*Kcnc3*	Kv3.3	8

1389120_at	*Kcnc3*	Kv3.3	9

1369847_at	*Kcnab1*	KCAB1	9

1369280_at	*Kcnk9*	TASK-3	9

1370595_a_at	*Kcnip4*	KCIP4	9

1370558_a_at	*Kcnc2*	Kv3.2	9

1387477_at	*Kcnk12*	THIK-2	10

1370545_at	*Kcna1*	Kv1.1	11

1368343_at	*Kcnh2*	Kv11.2	11

1368751_at	*Kcns3*	Kv9.3	12

1374582_at	*Kctd9*	KCD9	12

1368524_at	*Kcnc1*	Kv3.1	12

1370076_at	*Kcnj16*	Kir5.1	12

**Chloride Channels**			

1367772_at	Clns1a	ICLN	1

1367893_a_at	Clcc1	CLCC1	2

1378658_at	Clca6	CLCA6	9

1392453_at	Clcn3	CLCN3	10

1380547_at	Clcn3	CLCN3	10

1379932_at	Clcn4-2	CLCN4-2	12

**Calmodulin and CaM kinase**			

1369993_at	*Camk2g*	KCC2G	9

1398251_a_at	*Camk2b*	KCC2B	9

1369937_at	*Calm1*	CALM	11

1370853_at	*Camk2n1*	CK2N1	11

1368101_at	*Calm3*	CALM	12

**Calcium binding proteins**			

1369886_a_at	*Cabp1*	CaBP1	11

**IP3**			

1368005_at	*It r3*	ITPR3	7

**Glutamate Receptors**			

1387286_at	*Grm1*	mGluR1	1

1398889_at	*Grinl1a*	GL1AD	1

1396696_at	*Gria4*	GluR4	3

1369036_at	*Grik2*	GRIK2	4

1368572_a_at	*Grin1*	NR1	8

1368759_at	*Cacng2*	CCG2/TARP	10

1372724_at	*Grina*	NMDARA1	10

1369128_at	*Grik5*	GRIK5	10

1387559_at	*Grin3b*	NMDA3B	11

**GABA Receptors**			

1368170_at	*Slc6a1*	GAT1	1

1380170_at	*Gabarapl2*	GBRL2	1

1380828_at	*Gabra1*	GBRA1	1

1391653_at	*Gabrg2*	GBRG2	1

1370702_at	*Gabrr3*	GBRR3	9

1370804_at	*Gabarap*	GBRAP	10

1378842_at	*Gabarapl1*	GBRL1	11

1387383_at	*Gabbr2*	GBRR2	12

1369904_at	*Gabrb1*	GBRB1	12

1367783_at	*Gabarapl2*	GBRL2	12

**Glycine Receptors**			

1387696_a_at	*Glra2*	GLRA2	2

**Cholinergic Receptors**			

1370607_a_at	*Nrg1*	NRG1	9

1369845_at	*Chrna6*	ACHA6	9

1369252_a_at	*Chrna4*	ACHA4	9

1368615_a_at	*Slc18a3*	VAChT	10

1368734_at	*Chrnd*	ACHD	10

**Serotonin Recoptors**			

1369456_at	*Htr2b*	5HT2BR	5

1369119_a_at	*Htr7*	5HT7BR	11

**Adrenergic Receptors**			

1368534_at	*Adra1d*	ADA1D	8

1388757_at	*Adrbk1*	ARBK1	10

1369797_at	*Adra1a*	ADA1A	11

**Dopamin Receptors**			

1368602_at	*Slc6a3*	DAT	6

1368601_at	*Slc6a3*	DAT	9

1387520_at	*Drd4*	DRD4	9

1369856_at	*Drd5*	DRD5	11

1376345_at	*Drd1ip*	(*Caly*) CALY	12

**Cannabinoid Receptor**			

1369677_at Cnr1		CB1	1

**Anion Transporters**			

1367853_at	*Slc12a2*	NKCC1	1

1368082_at	*Slc4a2*	AE2	10

1368772_at	*Slc4a3*	AE3	10

To summarize the regulation of genes directly relating to motor neuron excitability, we find that most of the neuromodulator pathways (serotonergic, dopaminergic and adrenergic) seem to have a response of late up-regulation (clusters 8-10) while inhibitory neurotransmitter pathways (GABAergic and glycinergic) are in general down-regulated in the late phase of the injury response (clusters 1-2 and 12). The time series analysis also reveals regulation of genes coding for serotonergic (*Htr7*) and adrenergic (*Adrala*) receptors, which were not seen in the analysis of the late injury response [32] because they belong to time profile 11 with an initial suppression followed by a return to control levels. Other adrenergic receptor related genes overlap in the two studies, alpha 1D adrenoreceptor (*Adrald*, profile 8) and beta-adrenergic receptor kinase 1 (*Adrbkl*, profile 10) being up-regulated in the late phase. The dopamine reuptake transporter DAT (profiles 6 and 9) together with the gene coding for the dopamine receptor 4 (*Drd4*, profile 9) are up-regulated while a gene coding for the dopamine interacting protein Caly (profile 12) is down-regulated. The gene coding for dopamine receptor 5 (*Drd5*, profile 11) is subject to early down-regulation 2 and 7 days post injury, but returns to control levels in the late phase 60 days post injury. As in the preceding study [32] we find genes coding for GABAA subunits involved in channel trafficking and membrane incorporation to respond to the injury (*Gabarap*, profile10; *Gaparapll*, profile 11; *Gaparapl2*, profiles 1 and 12) in synergy with the down-regulation of the receptor subunits GABA_A _*α*_1 _(*Gabral*, profile 1), GABA_A _*γ*_2 _(*Gabrg2*, profile 1) and GABA_A _receptor *β*_1 _(*Gabrbl*, profile 12). One additional gene relating to GABA transmission is down-regulated, the GABA_B _receptor 2 (*Gabbr2*, profile 12).

The time analysis showed in accordance with [32] that the glutamatergic receptors seem to undergo a complex regulation, where several genes coding for different components of the NMDA receptor undergo regulation in late stages: *Grinl *(profile 8) and *Grina *(profile 10) are up-regulated and *Grinlla *(profile 1) is down-regulated. *Grin3b *also belong to the NMDA receptor complex, but seems to undergo early modulation with early down-regulation and a return to control levels in late phases (profile 11). The gene coding for the AMPA receptor regulator protein TARP is up-regulated (*Cacng2 *(stargazine), profile 10), suggesting an increased AMPA receptor mediated conductance.

With respect to the cholinergic system the time series analysis revealed similar patterns as was seen in the analysis of the late injury response [32], all genes being up-regulated in the late phases of the response. The up-regulation of genes coding for nicotinic alpha receptors 4 and 6 (*Chrna4 *and *Chrana6*, profile 9) together with the receptor subunit delta (*Chrnd*, profile 10) suggest an increased sensitivity to acetylcholine, while the up-regulation of the genes coding for vesicular acetylcholine transporter (VAChT, profile 10) as well as for NRG1 (*Nrg1*, profile 10) know to be involved in synaptic maturation suggest and increased release of acetylcholine.

Among the voltage gated ion channels, genes coding for Ca^2+ ^and Na^+ ^channel subunits are largely up-regulated in the late phase, while genes relating to K^+ ^have a more complex response with a balanced up- and down regulation of channel subunits. The genes coding for the Ca^2+ ^channel *α *subunits Cav3.2, Cav2.2 and the *γ*_1 _subunit (*Cacng1*, profile 10; *Caclh*, profile 8; *Cacna1b*, profile 9) are up-regulated in the late phase of the injury response, while only the gene coding for the Ca^2+ ^channel subunit *β*_4 _(*Cacnb4*, profile 4) exhibit a late down-regulation. Several genes of the Na^+ ^subunits also undergo regulation, where the genes coding for Na^+ ^a subunits Nav1.8 and Nav1.5 together with the *β*_1 _subunit (*Scn10a*, profile 8; *Scn5a*, profile 9; *Scn1b*, profile 8) exhibit late up-regulation, while only the gene coding for the a subunit Nav1.2 (*Scn2a1*) is down-regulated. Both the genes coding for the a subunit Nav1.7 (*Scn9a*) and the *β*_3 _subunit (*Scnb3*) belong to profile 5 with an early up-regulation and a return to control levels in the late phases. Ca^2+ ^binding proteins also exhibit a trend towards late phase up-regulation, i.e. CaM kinase related genes (*Camk2g *and *Camk2b*, profile 9) as well as one IP_3 _receptor (*Itpr3*, profile 7) and the Ca^2+ ^binding protein caldendrin (*Cabp1*, profile 11) are up-regulated. Two genes relating to calmodulin and CaM kinase (*Calm1 *and *Camk2n1*, profile 11) are transiently down-regulated in the early injury response, returning to control levels in the late part of the injury-response. The Cl^- ^reversal potential also seems to be subject to regulation towards a more depolarizing effect, suggested by the down-regulation of the gene coding for the Cl^- ^transporter NKCC1 (*Slc12a2*, profile 1) responsible for Cl^- ^extrusion and the up-regulation of the gene coding for the Cl^- ^symporters AE2 and AE3 (*Slc4a2 *and *Slc4a3*, profile 10) involved in Cl^-^accumulation inside the cell.

### The transcriptional regulation exerted by differentially expressed transcription factors

The common expression patterns of each consensus cluster suggest a common regulatory control of their associated genes. To reveal such common regulatory control, we looked for over-representation of transcription factor DNA binding sites in their proximal promoter regions, here set to 1000 base pairs (bp) upstream and 200 bp downstream of the coding region.

We focused the analysis on transcription factors that were associated with genes that exhibit differential expression in the time series. We find 34 genes coding for transcription factors to be differentially expressed in our data set out of the 119 transcription factor genes contained on the RAT230 2 chip. These genes are listed in Table 3 along with their cluster identity. To evaluate if these factors are likely to exert an influence on the regulation of the genes in each cluster, we predict their binding sites in respective gene promoters using computational models (see [37] for a review). Over-represented binding sites suggest a possible role for the corresponding transcription factor in the regulation of the tested set of genes. For this analysis we use ASAP [38] with position weight matrices (PWM) obtained from either JASPAR [39] or TRANSFAC [40] and the set of differentially expressed gene promoter sequences as background.

**Table 3:** Differentially expressed genes of transcription factors (TF) and over-representation (over-rep) of TF binding sequences within each gene cluster

	DEG of TF			
			
Clusters	Gene ID	Protein ID	TF over-rep	Motifs
**Cluster 1**	*Gtf2e2*	T2EB	E2F1	E2F1_Q3_01
	*Nfyc*	NFYC	FOXO1	E2F1_Q2_01
	*Olig1*	OLIG1		E2F1_Q6_01
	*Pbx3*	PBX3		FOXO1_01
	*Tfb2m*	TFB2M		FOXO1_02

**Cluster 2**	*E2f5*	E2F5	FOXO1	FOXO1_01
	*Morf4l1*	MO4L1		FOXO1_02

**Cluster 3**	*Aatf*	AATF	FOXO1	FOXO1_01
	*Btf3*	BTF3		FOXO1_02
	*Klf10*	KLF10		

**Cluster 4**	*Arid1b*	ARID1B	E2F1	E2F1_Q3_01
	*Gtf2ird1*	GT2D1	FOXO1	FOXO1_01
	*Irf9*	IRF9		FOXO1_02

**Cluster 5**	***Atf3***	**ATF3**	ATF4	ATF4_Q2
	***Myc***	**MYC**		
	*Tceb3*	**ELOA1**		
	*Ybx1*	YBX1		

**Cluster 6**			ATF4	ATF4_Q2
			E2F1	E2F1_Q3
			MYC	MYC_Q2
			SP1	SP1_01
				SP1_Q2_01
				SP1_Q4_01
				SP1_Q6
				SP1_Q6_01

**Cluster 7**	***E2f1***	**E2F1**	ATF3	ATF3_Q6
	***Foxo1***	**FOXO1**	ATF4	ATF4_Q2
	*Gtf3c1*	TF3C1	E2F1	E2F1_Q3
	***Sp1***	**SP1**	MYC	E2F1_Q3_01
			SP1	E2F1_Q4
				E2F1_Q4_01
				E2F1_Q6
				E2F1_Q6_01
				MYC_Q2
				SP1_01
				SP1_Q2_01
				SP1_Q4_01
				SP1_Q6
				SP1_Q6_01

**Cluster 8**	*Hsf4*	*HSF4*	ATF3	ATF3_Q6
	*Tbx3*	*TBX3*	ATF4	ATF4_Q2
			E2F1	E2F1_Q3
			MYC	E2F1_Q3_01
			SP1	E2F1_Q6
				MYC_Q2
				SP1_01
				SP1_Q2_01
				SP1_Q4_01
				SP1_Q6
				SP1_Q6_01

**Cluster 9**	*Arid1b*	*ARID1B*	ATF3	ATF3_Q6
	*Nr2f2*	*COT2*	E2F1	E2F1_Q3
	*Pou2f3*	*PO2F3*	SP1	E2F1_Q6
	*Runx3*	*RUNX3*		SP1_01
				SP1_Q2_01
				SP1_Q4_01
				SP1_Q6
				SP1_Q6_01

**Cluster 10**	***Atf4***	***ATF4***	ATF4	ATF4_Q2
	*Srebf1*	*SRBP1*	E2F1	E2F1_Q3
			MYC	E2F1_Q3_01
			SP1	E2F1_Q4
				E2F1_Q4_01
				E2F1_Q6
				E2F1_Q6_01
				MYC_Q2
				SP1_01
				SP1_Q2_01
				SP1_Q4_01
				SP1_Q6
				SP1_Q6_01

**Cluster 11**	*Dmrt1*	*DMRT1*	AFT3	ATF3_Q6
	*Gtf2h4*	*GTF2H4*	E2F1	E2F1_Q3
	*Zeb1*	*ZEB1*	MYC	MYC_Q2
			SP1	SP1_01
				SP1_Q2_01
				SP1_Q4_01
				SP1_Q6
				SP1_Q6_01

**Cluster 12**	*Nfia*	NFIA	AFT3	ATF3_Q6
	*Nkx6-2*	NKX6-2	E2F1	E2F1_Q3_01
			FOXO1	E2F1_Q4_01
				E2F1_Q6
				E2F1_Q6_01
				FOXO1_01

*DEG TF*: Differentially expressed genes (DEG) of transcription factors (TF). Genes of TF with annotated TRANSFAC motifs are highlighted in boldface. *TF over-rep*: TFs with over-represented binding sites in the set of genes belonging to the specified gene cluster. *Motifs*: Individual TRANSFAC motifs with Z scores above 3 based on ASAP conducted on the set of genes belonging to the specified gene cluster.

**Figure 2:**
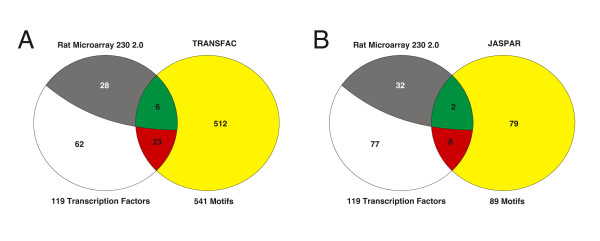
**Overlap between transcription factors and their binding motifs**. Transcription factor overlap between the 119 transcription factors included on the RAT230 2 chip and their binding motifs contained in TRANSFAC or JASPAR. **A**. Circle to the left represents the set of transcription factors included on the RAT230 2 chip, in total 119 of which 34 are differentially expressed. TRANFAC contains DNA binding motifs of 541 distinct transcription factors, 29 of which are on the array. Of these 6 are differentially expressed (green) and 23 are non-differentially expressed (red). Thus 28 (grey) differentially expressed transcription factors do not have binding motifs in TRANFAC, which on the other hand contains binding motifs of 512 transcription factors not contained on the microarray (yellow). **B**. Overlap between the transcription factors included on the RAT230 2 chip and their biding motifs contained in JASPAR. Same color code as in A.

There is presently little overlap between the transcription factors associated with the genes included on the RAT230 2 chip and the two databases containing their binding motifs, only 29 overlap with TRANSFAC and 10 with JASPAR (Figure 2A-B). Of these transcription factors (with both expression data and motif annotation), the ones associated with genes that do not display differential expression were excluded from the analysis, reducing the two sets of transcription factors to six for TRANSFAC (ATF3, ATF4, MYC, FOXO1, SP1 and E2F1) and two for JASPAR (SP1 and E2F1). We therefore used the TRANSFAC motifs for the promoter analysis. Because many of the TRANFAC motifs describe the same factor, this analysis includes 16 motifs. Patterns with a substantial enrichments are reported (Z score > 3, as in [38]), since these are likely to exert an influence in the regulation of the gene clusters, Table 3. Under-represented binding motifs signify that the corresponding transcription factor is very unlikely to exert any regulatory influence on the gene cluster under examination (Z score < -3, not included in this table).

**Figure 3:**
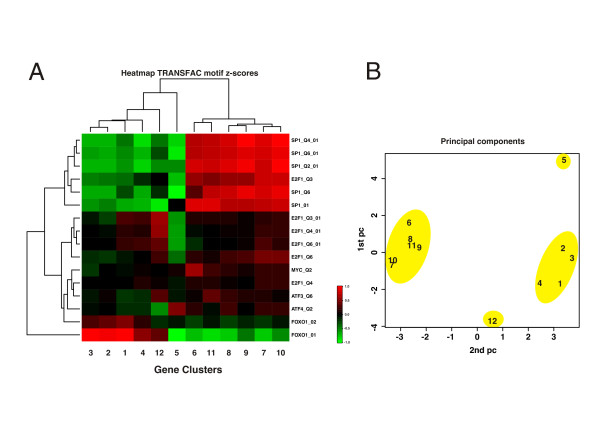
**Motif over-representation of differentially expressed transcription factors**. Over-representation statistics for each cluster profile of the 16 binding motifs contained in TRANSFAC representing the 6 differentially expressed transcription factors: ATF3, ATF4, E2F1, FOXO1, MYC, SP1. **A**. Heatmap of Z scores organized by two-way hierarchical clustering according to the Z score pattern across binding motifs and cluster profiles. For plotting purposes each Z score vector was normalized to unit variance. Over-represented genes are highlighted in red while under-represented transcription factors are shown in green (see color bar). It is clear that consensus clusters 1-4 and 6-11 group together, sharing common over- as well as under-represented transcription factor motifs. It is also striking that motifs over-represented in these groups typically will be under-represented in the other and vice versa. Consensus clusters 5 and 12 have different patterns of binding motifs Z scores. **B**. Principal components of the Z score vectors of each consensus cluster (columns of A) validate the pattern observed in A: cluster profiles 1-4 and 6-11 are closely grouped while cluster profiles 5 and 12 are separated from these.

To illustrate the balance between over- and under-representation of binding sites across gene clusters we next take advantage of the continuous range of Z scores, instead of only treating them as binary classifiers (over-representation or not). In combination across the gene clusters these values say something about the regulatory landscape, i.e. what binding sites are unchanged in most clusters, and what factors can explain the difference between clusters? We choose to visualize this as a hierarchical heatmap, where rows constitute the motif models and columns the gene clusters. In this representation the Z scores are organized by two-way hierarchical clustering, such that motifs that behave similarly in terms of over-representation will cluster together, as will the gene clusters with similar bindings site landscapes, Figure 3A. For plotting purposes each Z score vector (column) was normalized to unit variance. The heatmap of Z scores shows a clear pattern separating the expression profiles into groups sharing over-represented (red) as well as under-represented (green) sites. Motifs whose sites are over-represented in the group of clusters 1-4 are under-represented in the other major group clusters 6-11, and *vise versa*. The patterns of transcription factor binding site over- and under-representation are not identical for each of the consensus clusters, perhaps alluding to some degree of specific regulation within each cluster. Cluster profiles 5 and 12 have slightly separate motif binding patterns, though resembling clusters 6-11 and 1-4, respectively. The first two principal components of the Z score vectors of each time profile also reflect this clear separation, again reproducing the relationships obtained from the consensus clustering (Figure 1C), where profiles 1-4 and 6-11 group together, while clusters 5 and 12 are somewhat separated from these two main groups of regulation, Figure 3B.

By comparing the expression pattern of the differentially expressed genes coding for transcription factors (which cluster they belong to) with the time profile of the clusters where their binding sites are over-represented (target cluster) some general picture emerges. The differentially expressed genes of SP1 (*Sp1*) and E2F1 (*E2f1*) belong to cluster 7, which show an early up-regulation that is maintained throughout the injury response. The pattern of binding site over-representation suggests that SP1 may have an auto regulatory role, as its binding sites are over-represented in cluster profiles 6-11 and under-represented in cluster profiles 1-4 and 12. It thus seems to enhance expression of genes in clusters 6-11. The pattern of over-representation for the binding site of E2F1 on the other hand suggest that this transcription factor has a very broad activation potential as it seems to target clusters 1, 4 and 6-12. The expression of the gene coding for FOXO1 (*Foxo1*) follows time profile 7, and the over-represented binding sites of this transcription factor in clusters 1-4 and 12 suggests that it suppress the expression of genes in these clusters as they are mainly down-regulated. The gene of transcription factor ATF4 (*Atf4*) belongs to cluster profile 10 showing a late up-regulation. The binding sites of ATF4 are over-represented in cluster profile 5 so its late expression may be involved in the late suppression of genes in cluster profile 5. Cluster profile 5 on the other hand contains two differentially expressed genes coding for transcription factors, MYC (*Myc*) and ATF3 (*Atf3*). The binding sites of MYC are over-represented in the promoters of genes belonging to cluster profiles 6, 7, 8, 10 and 11. This could indicate a role for MYC in the positive regulation in the early response (2 and 7 days post injury) of cluster profiles 6-8, while it would have the opposite effect suppressing the expression of genes in clusters 10 and 11 (see *Discussion*). As the genes of cluster profile 5 reside to control levels in the late phase of the injury response (21 and 60 days post injury), it seems unlikely that transcription factors following this time profile exert any effect on gene regulation at these late time points. The binding sites of ATF3 are over-represented in profiles 7, 8, 9, 11 and 12, suggesting that this transcription factor affects the early up-regulation in clusters 7 and 8 while the profile of cluster 9 doesn't seem to undergo any significant regulation at these time points compared to control (day 0). As for transcription factor MYC it seems likely that ATF3 participate in the suppression of gene expression in the early response in clusters 11 and 12.

This analysis showed that although we could only ascribe motifs to 6 of the 34 transcription factors encoded by differentially expressed genes, the complex correlation between the timing of their gene expression and the down- or up-regulation of their putative cluster targets suggests an intricate interaction between the transcription factors in shaping the transcriptional response.

## Discussion

Excitability changes in motor neurons have been strongly implicated with the emergence of pathophysiological hyper-reflexia in late stages of spinal cord injury, since self-sustained activity can be induced in motor neurons upon brief stimuli of sensory afferents in the complete absence of descending fibers from the brain [3,5,9,23]. By focusing on the transcriptional time course of these cells in combination with transcription factor motif analysis we shed light on the regulatory mechanisms underlying the re-expression of these plateau potentials, a key mechanism behind the pathophysiology of spasticity. In particular, we use a robust consensus cluster algorithm [35] to identify distinct expression time profiles. This consensus cluster algorithm conducted on the 3,708 most differentially expressed genes identified 12 distinct time profiles. These expression time profiles separate the differentially expressed genes into groups that most likely are under common regulatory control and enable us to associate individual genes with a specific pattern of expression over time.

### Cluster analysis identify distinct time profiles that define the timing of general biological responses to injury

The 12 time profiles divide into two main groups relating to the late response, one of down-regulation (time profiles 1-4 and 12) and one of up-regulation (time profiles 6-10), Figure 1B and 1C. Besides these two main categories of late regulation expression patterns there are two clusters with a predominant early response, time profile 5 with an early up-regulation at day 2 and time profile 11 with an early down-regulation at days 2 and 7, which both falls back towards control levels 21 and 60 days post injury.

Ontology analysis of the genes associated with each cluster profile shows that the motor neurons engage in different biological processes as the transcriptional response evolves over time. In particular time profile 5 signifies a marked immunological and inflammatory response of the motor neurons in the early phase after injury, which return to control levels in the late phases. Such immunological processes are known to be pronounced in the early phase of spinal cord injury from studies conducted on entire spinal cord tissue [1,41-43], but have not previously been identified at the motor neuron level. This finding corroborate recent studies, indicating that a neuronal immune response is included in the repertoire of processes motor neurons can engage as a means of protection against damage [44]. Cluster profiles 1-4 as well as 12 all describe different patterns of transcript down-regulation. "Cell-cell adhesion" is clearly down-regulated in profiles 1 and 3, suggesting that the direct interaction of motor neurons with their neighboring cells are reduced. Synapse stripping, including the removal of synapses from the perikaryon and dendrites, is a pronounced phenomenon after axonal damage to motor neurons [44]. The down-regulation of genes related to "ensheathment of neurons" in cluster profile 1 suggests an effect of the injury on the myelination of motor neurons not previously associated with this neuronal population. Another prominent down-regulated mechanism involves mitochondrial related energy metabolism (time profile 12). The translational machinery is also down-regulated (profiles 2-3). The down-regulation of chromatin structures (profile 1) suggests that the DNA could be unfolding towards a more favorable transcriptional state, while "RNA splicing" of profile 2 suggests a reduction in the mRNA processing. Among the prominent up-regulated profiles, pathways relating to neuronal development (profiles 8-10), suggest that injury induce developmental processes as a late response. This finding indicates that a differentiated and mature neural population in the spinal cord is capable of re-engaging in developmental pathways, presumably attempting to ameliorate the conditions of the damaged spinal cord and compensate for the lack of inputs. It is also clear that plasma membrane transporter activity of various kinds are significantly up-regulated suggesting a very strong control of the electro-chemical transmembrane gradients, possibly also reflecting the changing chemical requirements of the motor neurons. The motor neurons also up-regulate processes directly relating to membrane excitability and neural transmission, suggesting that the motor neurons change their synaptic strength, both pre-synaptically through modulation of axon terminals with increased machinery for acetylcholine release and post-synaptically through modulation of receptor channels as well as changed membrane excitability.

### Differentially expressed genes relating to motor neuron excitability and injury-induced spasticity

Ontology analysis provides general terms of activity suggesting some biological functions of each cluster profile, but the over-represented ontologies only represent a relative small proportion of the genes contained in each cluster. To dissect out all the gene constituents that relate to changes in motor neuron excitability and injury-induced spasticity we therefore focused on genes involved in neural transmission. In a previous study we examined the late transcriptional response of motor neurons compared to their sham-operated counterparts 21 and 60 days post injury [32]. From this study it was clear that the motor neurons change their post-synaptic receptor composition moving towards a more excitable state through a reduction of the ionotropic GABAergic receptors and an increase of the ionotropic glutamatergic, adrenergic and cholinergic receptors. Ca^2+ ^and Na^+ ^ion channels also responded to the injury, where the most noticeable changes related to the modulation of persistent inward currents involved the ancillary subunits possibly changing the conductivity and membrane incorporation of existing ion channels. The functional consequences of these changes are discussed extensively in [32].

Extracting the differentially expressed genes affecting motor neuron excitability based on their changed expression over time identifies many of the same candidates, though the differential expression in the present case is based on mutual reference across the time points rather than pair wise comparisons within each time point with time-matched sham controls. The fact that the two different strategies of analysis identify many of the same gene candidates affecting motor neuron excitability supports the robustness of our findings. The time series analysis clearly shows that most of the genes relating to motor neuron excitability and injury-induced spasticity are found in clusters with late regulation, where genes coding for receptors and channels with excitatory effects are predominantly up-regulated while inhibitory receptors are primarily down-regulated. This shows that the observed progressive increase of the hyper-reflexia strongly correlates with the increased expression of genes enhancing motor neuron excitability and reversely correlates with the decreasing expression of the GABA_A _receptor system. This pattern of expression was also reflected in the ontology analysis, where late phase up-regulation of time profiles 9 and 10 contains terms of "gated channel activity", "regulation of neurotransmitter levels", "synaptic transmission" and "ion exchanger activity", while time profile 12 showing a general down-regulation as a response to the injury contains "alkali metal ion binding" and "anion channel activity" reflecting a decrease in anion channel signaling (K^+ ^and Cl^-^).

The apparent conclusion from this analysis is that many of the genes affecting motor neuron excitability share expression patterns, where the majority having an excitatory effect are up-regulated in late stages of the injury response (21 and 60 days post injury) falling into clusters 6-10 while the majority of the genes relating to inhibition are down-regulated in the late phases of the response falling into cluster profiles 1-4 and 12.

### Transcriptional control of gene clusters

The distinct expression pattern shared by the genes of each consensus cluster and their associated ontology terms suggest a common regulatory control of each gene cluster. This possibility was examined by matching transcription factor binding sites with core promoter sequences of the genes associated with each cluster using ASAP [38] with motifs obtained from the TRANSFAC database. This database is at present not fully annotated for all know transcriptions factors, and at the time of writing TRANSFAC contained motifs for 6 of the 34 transcription factors encoded by genes identified in the present study as differentially expressed (SP1, E2F1, FOXO1, ATF3, ATF4, MYC). Based on over-representation analysis of their binding sites we find that the expression pattern for the genes of these six transcription factors correlate nicely with the time profiles of their putative target gene clusters. The genes of SP1 (*Spl*), E2F1 (*E2fl*) and FOXO1 (*Foxo1*) all belong to time profile 7 with a common up-regulation throughout the injury response, but have different targets clusters.

The binding sites of SP1 are over-represented in profiles 6-11 suggesting a positive regulation by this transcription factor of the genes associated with these profiles. The binding sites of E2F1 are over-represented in gene clusters 1, 4 and 6-12 suggesting the interaction of this transcription factor with other proteins to focus its regulatory effect to fewer transcript targets. Interestingly the non-specific general activator proteins SP1 and E2F1 have been shown to interact to promote transcription [45] and in motor neurons they can drive the transcription of the motor neuron specific transcription factor HB9 [46]. Their common binding site over-representation in cluster profiles 6-11 therefore suggest that they participate in a general activation of transcription of their associated genes.

The binding sites of FOXO1 are over-represented in cluster profiles 1-4 plus 12 and it thus seems to have a suppressive effect. The fork-head transcription factor FOXO1 has not previously been associated with the spinal cord, but it has been found in other parts of the developing and adult brain [47].

ATF3 and ATF4 both belong to the mammalian activation transcription factor/cAMP responsive element-binding (CREB) protein family of transcription factors and they have both been associated with trauma relating to the spinal cord. ATF3 has been shown to be up-regulated in motor and sensory neurons subject to axotomy [48] as well as in spinal neurons post injury [49]. The up-regulation of ATF4 has been associated with ischemia of both brain and spinal cord [49]. The genes of these two transcription factors follow different expression profiles. The gene of ATF4 (*Atf4*) belongs to expression profile 10 and thus is subject to an initial repression followed by a late up-regulation. The binding site of ATF4 is over-represented in time profile 5 suggesting that its late expression is suppressing the genes of cluster 5 as they return to control levels after the initial up-regulation. Since the gene of ATF3 (*Atf3*) belongs to cluster 5 it therefore seems to be subject to the repression of ATF4. The early up-regulation of the gene coding for ATF3 (*Atf3*) on the other hand might correlate with initial up-regulation of expression in clusters with over-representation of its binding site, clusters 7, 8, and 9. The binding site of ATF3 is also over-represented in gene clusters 11 and 12 with early down-regulation of expression, thus ATF3 must work together with other transcription factors to explain this apparent opposing effect on the expression of its target genes.

MYC, a member of the myc-family of transcription factors, is a complex regulator of general transcriptional activation [50] and has been associated with immediate early injury response of neurons in the spinal cord [51]. The gene of this transcription factor belongs to cluster profile 5 together with the gene of ATF3. The binding site for MYC is over-represented in gene clusters 6-8 and 10-11 suggesting a role in the early regulation of the genes in these clusters.

The motif analysis could not be conducted on the full set of transcription factors with differential expression of their associated genes as not all of these were contained in TRANSFAC, which otherwise would have enabled us to make a more complete estimate of the regulatory network underlying the expression patterns observed in the motor neurons as a response to the injury. Apart from the six transcription factors with know motifs, we note that several of the other transcription factors with differential expression of their associated genes have been implicated with central nervous system development or its response to trauma. These include the down-regulated genes of the transcription factors E2F5 (*E2f5*) [51], GT2D1 (*Gtf2ird1*, synonymous with BEN) [52] and NFIA (*Nfia*) [53] as well as the up-regulated genes of transcription factors PBX3 (*Pbx3*) [54] and NRF2F (*Nrf2f*) [55]. In particular OLIG1 and NKX6-2 have been implicated in motor neuron differentiation early in development [56-58]. The down-regulation of their genes (*Olig1 *and *Nkx6-2*) compared to the un-injured state also suggests a role for these in the maintenance of normal motor neuron function and identity.

There is growing supporting evidence for a model where the pattern of neurogenesis is achieved through a mechanism of controlled repression of transcription upon a background of non-specific transcriptional activation [46,59,60]. It therefore seems like the developing nervous system is subject to general transcriptional activation by non-specific general-activator transcription factors while the cell specific processes are directed by controlled inhibition of transcription. If this mechanism applies to the adult organism, and in particular to the injury response of motor neurons observed in the present case, the down-regulation of a suppressor transcription factor could have the same effect as the up-regulation of a transcription factor enhancer targeting the same genes, i.e. induce transcription. The expression pattern of the genes coding for SP1, E2F1 and MYC together with their broad cluster targets suggest that these un-specific activators of transcription enhance the general transcriptional capacity of the motor neurons, while the expression pattern of genes coding for other more specific regulators of transcription like OLIG1 or NKX6-2 could function to shape the response by relief or activation of targeted suppression of specific sets of genes, supporting the hypothesis of suppressor mediated transcriptional specificity.

The observed combination of up- and down-regulated transcription factors therefore suggests a redirection of the transcriptional program, where the transcription factors of clusters 1-4 and 12 must be involved in the maintenance of normal motor neuron function and their down-regulation together with the up-regulated transcription factors of cluster profiles 5-10 suggest a dynamic transition to a new transcriptional state. It is also clear from our analysis that these transitions through different transcriptional states across time are mediated by the interactions of several transcription factors.

## Conclusion

The present study expand our previous work on the late transcriptional response of motor neurons following spinal cord injury by adding data from the early phase, resulting in a data set comprising days 0, 2, 7, 21 and 60 post injury. The consensus clustering with the subsequent ontology analysis enabled us to identify distinct expression time profiles from which we can describe the biological processes as they progress over time and correlate them to the pathophysiologal development of spinal cord injury. Extracting genes directly relating to motor neuron excitability further focus the analysis towards changes associated with injury-induced hyper-reflexia. The cluster identity of these genes in complement with the over-representation analysis on GO terms and transcription factor binding sites indicate some general mechanism of how the motor neurons regulate their membrane excitability as a response to the injury.

Our analysis clearly suggests that the transcriptional response of the motor neurons to injury is complex, and that the observed increased excitability is the result of many interacting factors. This study therefore provides a first step towards an understanding of the correlation between the transcriptional regulation in an individual cell population and the physiological state of a biologically complex system. In this light it therefore seems unlikely that the suppression of a single gene or protein relating to ion channels or receptors will have a significant effect in reducing motor neuron excitability to alleviate injury-induced spasticity. We therefore suggest an alternative approach, where the manipulation of the transcriptional regulators such as the identified transcription factors could be used to alter the transcriptional response to prevent the motor neurons from entering a state of hyper-excitability.

## Methods

### Spinal cord preparation

All handling of animals was approved by the Danish Animal Experiments Inspectorate. The handling and experimental procedures of the animals were conducted at University of Copenhagen (Denmark) and the isolated spinal cord tissue was further processed at Karolinska Institutet in Stockholm (Sweden).

Adult male Wistar rats (325-480 g) were used in this study. The animals used for microarray hybridization were separated into five groups: controls of un-injured animals (Control; n = 4), spinalized for 2 days (Spi-2; n = 6), spinalized for 7 days (Spi-7; n = 5), spinalized for 21 days (Spi-21; n = 8) and spinalized for 60 days (Spi-60, n = 8). The Spi-21 and Spi-60 samples were obtained from a previous study [32] and the remaining samples were produced as described therein. In short, laminectomy was performed on animals under anesthesia between the lumbar L2 and L3 vertebras and injury was inflicted on the spinal cord by removing 1-2 mm tissue at the sacral S2 segment. After spinalization, the wound was closed suturing muscles, muscle fascia and skin separately. Care was taken to relieve pain post-operatively. Until termination of the experiment the welfare of the rats were routinely checked (e.g. for signs of infections, motor loss or bladder dysfunction) and rats that showed signs of distress were immediately euthanized. Since the spinal cord injury was inflicted at the S2 level only the motor and sensory functions of the tail were affected leaving the bladder, bowel as well as hind limb functions intact. Motor neurons were labeled *in vivo *with Fluoro-Gold (Fluorochrome) as described in [25]. At the day of termination animals were anesthetized with pentobarbital (initially 20 mg/kg and then 5 mg/kg every 30 minute, Mebumal^®^, SA - Sygehus Apotekerne) and the sacrocaudal spinal cords were removed, snap-frozen in liquid nitrogen and stored at -80°C until further processed.

### Motor neuron extraction and microarray preparation

Fluoro-Gold labeled motor neurons (at the S3-S4 level) were laser microdissected and their RNA extracted and amplified as previously described [32]. In short, retrogradely Fluoro-Gold labeled motor neurons were isolated from 10 thin spinal cord cryosections using the Leica AS laser microdissection system (Leica Microsystems) at room temperature. From each rat the total RNA was isolated from 70-200 laser microdissected motor neurons using the PicoPure™ RNA Isolation Kit (Arcturus) and the messenger RNA (mRNA) fraction was amplified in a two round T7 linear amplification process using the RiboAmp™ HS RNA Amplification Kit (Arcturus). The complementary DNA (cDNA) product from the 2^nd ^round of the amplification process was used to generate biotin-labelled antisense RNA (aRNA) (GeneChip^® ^Expression 3'-Amplification Reagents for IVT Labeling, Affymetrix). The integrity and concentration of the amplified and biotinylated aRNA was assessed on an Agilent RNA chip with the Agilent 2100 bioanalyzer (Agilent Technologies) both before and after fragmentation. Only samples of good integrity were further used and 15 *μ*g of the fragmented samples were hybridized to GeneChip^® ^Rat Genome 230 2.0 Arrays (RAT230_2 chip, Affymetrix) and subsequently scanned. Each array always originated from a single animal. The Agilent analysis and microarray hybridizations were conducted at the Affymetrix core facility at Novum (Bioinformatics and Expression Analysis core facility, Department of Biosciences and Nutrition, Karolinska Institutet, Huddinge, Sweden).

### Microarray preprocessing

The microarray normalization and the analysis for detection of significantly differentially expressed genes was adopted from Ryge and colleagues [34]. We used the Affymetrix probe sets verbatim, but discarded those not included in the Ensembl database for the RAT230 2 chip prior to the statistical analysis, reducing the set of probes from 31,099 to 12,919. The microarrays were then background compensated, normalized and RMA (Robust Multi-array Average) expression summaries were calculated [34,61]. Additional background compensation was carried out on the expression summaries as described in [25]. Inspection of the normalized distributions showed that all microarray RMA profiles followed the average distribution throughout the intensity range, validating the microarray pre-processing steps (Additional file 2). The RMA expression summaries together with the raw CEL files for all microarrays were submitted to the Gene Expression Omnibus (GEO; http://www.ncbi.nlm.nih.gov/geo/) hosted by the National Center for Biotechnology Information and can be accesses under accession number GSE19701.

### Differentially expressed genes

To determine the significantly differentially expressed genes across the five time points adjusted ANOVA analysis' were performed using three different statistical procedures: Cyber-T, limma and SAM (described in [62-65]). Each time point was treated as a separate "biological condition", in essence identifying genes violating the null hypothesis of equal mean across all conditions. The resulting test statistics of all three procedures were then used to create a conglomerate ranking of each gene reflecting their degree of significance across all three tests as described in [34]. For the purpose of clustering a FDR cut-off of 0.02 was chosen classifying 3,708 out of 12,919 genes as differentially expressed. To compensate for multiple testing the p-values of Cyber-T and limma were converted to FDRs using the approach of Allison and colleagues [66], whereas the FDRs of SAM are based on a methodology of permutation and re-sampling of the data (i.e. these FDRs are output from the SAM analysis directly).

### Consensus Cluster analysis

ClusterLustre, a robust consensus clustering method, was used to group the set of differentially expressed genes into clusters of reliably classified gene expression patterns [35]. To avoid clustering according to magnitude but rather on common patterns of expression, the expression level of each transcript were normalized prior to clustering:(1)

for gene *n *= 1,...,*N *and microarray *m *= 1,...,*N*. Here *y*_*nm *_represents the RMA expression value of transcript *n *on chip *m *and  the average expression level of transcript *n *across all *M *microarrays. The denominator is used to confine the expression variance to the interval [-1,1]. The consensus clustering algorithm aims at producing robust clustering results by averaging over multiple clustering runs such that the sensitivity to settings and initialization are diminished. The algorithm works as follows (for more details see [35]). Initially, 30 scans with k-means in the interval k = 6...14, leading to a total of 9 * 30 = 270 clustering runs are performed. From these a co-occurrence matrix reflecting the pair wise probability of transcripts falling in the same cluster is made. The resulting co-occurrence matrix thus describes the pair wise empirical probability of transcripts falling in the same cluster throughout the 270 clustering runs. In the final step, one minus the co-occurrence matrix (= the dissimilarity matrix) is used as input to hierarchical clustering. This gives a robust clustering because genes that fall in the same cluster across most cluster runs will have a small dissimilarity and thus be grouped together, whereas this is not the case for genes that infrequently by chance are in the same cluster. So, consensus clustering gives clearer clusters than a direct application of hierarchical clustering on the data [35].

In the present study, we found 12 consensus clusters to be optimal in terms of representing distinct expression time profiles (each containing approximately 150-600 genes). A full list of the differentially expressed genes grouped according to their cluster ID is provided in Additional file 1.

### Over-representation analysis on identified gene clusters

#### Ontologies

The DAVID online ontology-cluster tool [67,68] was used on each of the 12 identified gene clusters to identity groups of over-represented ontologies sharing a gene overlap of minimum 70%, from which a significant representative term was extracted. Ontology clusters were deemed significant if they contained ontologies with p-values below 0.03.

#### Transcription factors

To identify transcription factors that may be involved in regulation of the identified gene clusters we performed motif analysis on each cluster looking for transcription factor binding sites in the core promoter sequences of their constituent genes. For this analysis we obtained the sequences from the Ensembl database of 1000 bp upstream and 200 bp downstream of the coding region of all differentially expressed genes using biomaRt. Each set of promoter sequences pertaining to a distinct time profile was analyzed for over- and under-represented transcription factor binding sites using ASAP [38] with position weight matrices (PWM) obtained from JASPAR [39] or TRANSFAC [40]. ASAP searches the set of promoter sequences using a transcription factor motif (i.e. PWM) and calculates the number of times a given binding site appears, producing a Z score reflecting the likelihood of its over-representation (positive) or under-representation (negative) compared to the amount of times it appears in a pre-defined background. In the present case the 3,708 differentially expressed genes were used as background for each cluster.

We note that the presented findings resting on this methodology are based on predictions and that more than one transcription factors may bind to the same target. This means that over-representation of a certain binding site for one transcription factor in a gene promoter region may not always predict the binding of the exact same factor.

### Differentially expressed genes relating to motor neuron excitability

Significantly differentially expressed genes relating to motor neuron excitability were extracted along with their corresponding cluster ID, belonging to the same categories of ion channels and neurotransmitter receptors as described in [25]. In particular genes relating to Ca^2+^, Na^+^, Cl^- ^and K^+ ^channels as well as genes relating to glutamatergic, GABAergic, glycinergic, cholinergic, serotonergic, adrenergic and dopaminergic receptors were identified and are shown in Table 3. Ca^2+ ^binding genes relating to calmodulin and IP_3 _were also included in this table.

### Software

The microarray analysis was done using R http://www.r-project.org/ and Bioconductor http://bioconductor.org. Cyber-T source code was obtained from the website http://cybert.microarray.ics.uci.edu/. Open source software was used for clustering (ClustreLustre, http://eivind.imm.dtu.dk/staff/winther/software.html) and promoter analysis (ASAP, http://asap.binf.ku.dk/Asap/Home.html). Separate scripts for integration of R and bioconductor data formats with these programs were developed and are included as supplementary material (Additional files 3 and 4).

## Authors' contributions

JR participated in the design of the study, participated in data acquisition, performed data analysis and wrote the manuscript. OW formed strategy for and participated in cluster analysis, participated in promoter analysis and participated in manuscript revision. JW performed the animal surgeries and participated in data acquisition. ACW performed the molecular work and participated in data acquisition. AS participated in promoter analysis and participated in manuscript revision. HH participated in design of study and participated in writing the manuscript. OK participated in the design and coordination of the study and wrote the manuscript. All authors read and approved the final manuscript.

## Supplementary Material

Additional file 1 **Table containing all differentially expressed genes and their consensus cluster ID**. Click here for file

Additional file 2 **Figure showing Validation of normalization**. Distributions of linear-normalized probe intensities (perfect match, PM) and RMA expression summaries. Quantile-quantile plots B and D illustrate the variation of the distribution tails of the distributions plotted in A and C. The nice overlap of both PM and RMA distributions validates the normalization procedure and the quality of the data obtained from different animals at different time points following spinal cord injury. **A**. Plot of linear-quantile-normalized PM distributions of each microarray. **B**. Quantile-quantile plot: linear-quantile-normalized PM distributions (shown in A) plotted against the average PM distribution. **C**. Distributions of normalized RMA expression summaries, based on PM values from A and B, which has undergone further quantile normalization. **D**. Quantile-quantile plot: normalized RMA expression summary distributions (shown in C) plotted against the average RMA distribution. Click here for file

Additional file 3 **R scripts source file**. An R source file containing scripts for probe annotation, export and import of expression values to and from ClustreLustre and cluster plots, extraction of sequences using biomaRt for ASAP analysis.
Click here for file

Additional file 4 **R scripts example**. Text file illustrating an example of how to run scripts (details on ClustreLustre and ASAP usage is described in original papers) Click here for file
