# Supplementary figures and images for: Transcriptional regulation of gene expression clusters in motor neurons following spinal cord injury
Source: BMC Genomics. 2010 Jun 9;11:365. doi: 10.1186/1471-2164-11-365 (PMC2900267; doi:10.1186/1471-2164-11-365)

A

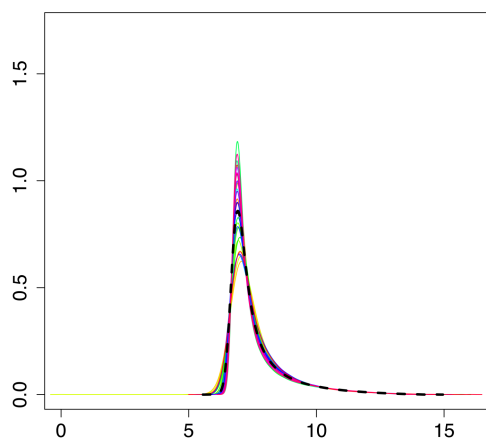

C

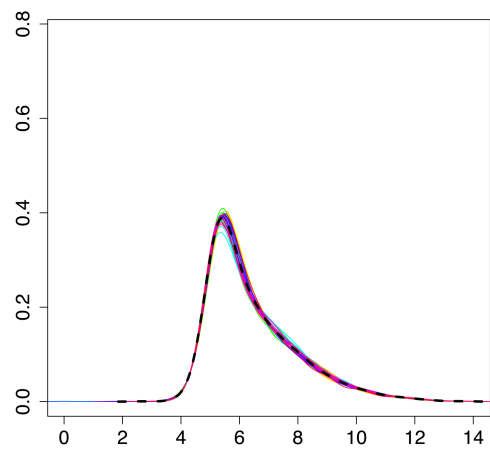

B

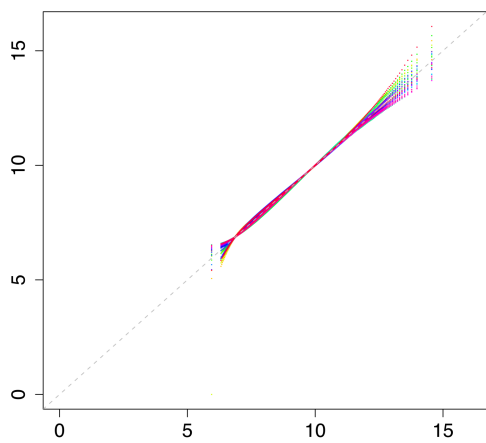

D

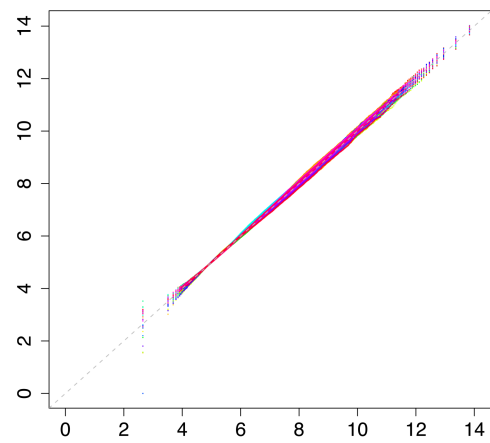

Supplement: Additional file 1 — Table containing all differentially expressed genes and their consensus cluster ID. [file 1471-2164-11-365-S1.PDF]
