# Supplementary material for: Transcriptional regulation of gene expression clusters in motor neurons following spinal cord injury
Source: BMC Genomics. 2010 Jun 9;11:365. doi: 10.1186/1471-2164-11-365 (PMC2900267; doi:10.1186/1471-2164-11-365)
Supplement: Additional file 2 — Figure showing Validation of normalization. Distributions of linear-normalized probe intensities (perfect match, PM) and RMA expression summaries. Quantile-quantile plots B and D illustrate the variation of the distribution tails of the distributions plotted in A and C. The nice overlap of both PM and RMA distributions validates the normalization procedure and the quality of the data obtained from different animals at different time points following spinal cord injury. A. Plot of linear-quantile-normalized PM distributions of each microarray. B. Quantile-quantile plot: linear-quantile-normalized PM distributions (shown in A) plotted against the average PM distribution. C. Distributions of normalized RMA expression summaries, based on PM values from A and B, which has undergone further quantile normalization. D. Quantile-quantile plot: normalized RMA expression summary distributions (shown in C) plotted against the average RMA distribution. [file 1471-2164-11-365-S2.HTML]

Gene List

| Affy ID | UniProt ID | Entrez Gene ID | Ensembl Gene URL | Ensembl Transcript URL | Gene Symbol | Description | Cluster ID |
| 1369677\_at | P20272 | 25248 | ENSRNOG00000008223 | ENSRNOT00000010850 | Cnr1 | Cannabinoid receptor 1 (CB1) (CB-R) (Brain-type cannabinoid receptor). [Source:UniProtKB/Swiss-Prot;Acc:P20272] | 1 |
| 1382479\_at |  | 311236 | ENSRNOG00000029798 | ENSRNOT00000044595 | NP\_001101223.1 | leucine rich repeat containing 4C [Source:RefSeq\_peptide;Acc:NP\_001101223] | 1 |
| 1368854\_at | P62762 | 24877 | ENSRNOG00000005345 | ENSRNOT00000007609 | Vsnl1 | Visinin-like protein 1 (VILIP) (Neural visinin-like protein 1) (NVL-1) (NVP-1) (21 kDa CABP). [Source:UniProtKB/Swiss-Prot;Acc:P62762] | 1 |
| 1383129\_at | Q6P689 | 362065 | ENSRNOG00000036816 | ENSRNOT00000055245 | LOC362065 | Integral membrane protein GPR177 precursor (Protein wntless homolog) (Protein evenness interrupted homolog) (EVI). [Source:UniProtKB/Swiss-Prot;Acc:Q6P689] | 1 |
| 1385559\_at |  | 497935 | ENSRNOG00000021540 | ENSRNOT00000012061 | RGD1563441\_predicted |  | 1 |
| 1383151\_at |  | 246150 | ENSRNOG00000026319 | ENSRNOT00000010118 | Akap9 | A kinase (PRKA) anchor protein (yotiao) 9 [Source:RefSeq\_peptide;Acc:NP\_001032170] | 1 |
| 1383327\_at | Q9JID1 | 64031 | ENSRNOG00000014779 | ENSRNOT00000020155 | Pdcd4 | Programmed cell death protein 4 (Death up-regulated gene protein). [Source:UniProtKB/Swiss-Prot;Acc:Q9JID1] | 1 |
| 1381564\_at |  | 289437 | ENSRNOG00000002054 | ENSRNOT00000002811  ENSRNOT00000061583  ENSRNOT00000061579 | NP\_001099463.1 | glomulin, FKBP associated protein [Source:RefSeq\_peptide;Acc:NP\_001099463] | 1 |
| 1388794\_at | P84586 | 679457  680944  307779  302855 | ENSRNOG00000028858  ENSRNOG00000012138  ENSRNOG00000000866 | ENSRNOT00000041672  ENSRNOT00000016184  ENSRNOT00000001154 | LOC680944  LOC302855 | Heterogeneous nuclear ribonucleoprotein G (hnRNP G) (RNA-binding motif protein, X chromosome). [Source:UniProtKB/Swiss-Prot;Acc:P84586]  RNA binding motif protein, X chromosome [Source:RefSeq\_peptide;Acc:NP\_001020834] | 1 |
| 1398884\_at |  | 300257 | ENSRNOG00000012985 | ENSRNOT00000017794 | NP\_001100264.2 | prefoldin 5 [Source:RefSeq\_peptide;Acc:NP\_001100264] | 1 |
| 1393343\_at |  | 301555 | ENSRNOG00000015633 | ENSRNOT00000021528 | NP\_001100393.1 | cullin 3 [Source:RefSeq\_peptide;Acc:NP\_001100393] | 1 |
| 1371387\_at | P80431 | 303393 | ENSRNOG00000028451 | ENSRNOT00000033847 | Cox7b | Cytochrome c oxidase polypeptide VIIb, mitochondrial precursor. [Source:UniProtKB/Swiss-Prot;Acc:P80431] | 1 |
| 1372000\_at |  |  | ENSRNOG00000017765 | ENSRNOT00000046789 | Net1 | neuroepithelial cell transforming gene 1 [Source:RefSeq\_peptide;Acc:NP\_001034112] | 1 |
| 1387950\_at | Q9WV50 | 192180 | ENSRNOG00000020391 | ENSRNOT00000027653 | Nip7 | 60S ribosome subunit biogenesis protein NIP7 homolog (PEachy) (KD93). [Source:UniProtKB/Swiss-Prot;Acc:Q9WV50] | 1 |
| 1369414\_at |  | 114095 | ENSRNOG00000020392 | ENSRNOT00000027666 | Stxbp3 | syntaxin binding protein 3 [Source:RefSeq\_peptide;Acc:NP\_446089] | 1 |
| 1378359\_at |  | 691335  682750 | ENSRNOG00000012446 | ENSRNOT00000040110 | LOC682750 |  | 1 |
| 1381903\_at |  |  | ENSRNOG00000005285 | ENSRNOT00000007221 | Fbxo33 | F-box protein 33 Gene [Source:MGI Symbol;Acc:MGI:1917861] | 1 |
| 1398807\_at | P35815 | 24667 | ENSRNOG00000030667 | ENSRNOT00000041993 | Ppm1b | Protein phosphatase 1B (EC 3.1.3.16) (Protein phosphatase 2C isoform beta) (PP2C-beta). [Source:UniProtKB/Swiss-Prot;Acc:P35815] | 1 |
| 1373123\_at |  | 306734 | ENSRNOG00000019108 | ENSRNOT00000025812 | RGD1310671\_predicted |  | 1 |
| 1390587\_x\_at |  |  | ENSRNOG00000026971 | ENSRNOT00000033772 |  |  | 1 |
| 1386950\_at | P62142 | 25594 | ENSRNOG00000004612 | ENSRNOT00000006190 | Ppp1cb | Serine/threonine-protein phosphatase PP1-beta catalytic subunit (EC 3.1.3.16) (PP-1B). [Source:UniProtKB/Swiss-Prot;Acc:P62142] | 1 |
| 1370389\_at |  | 192179 | ENSRNOG00000004613 | ENSRNOT00000049494 | Gpm6b | glycoprotein m6b [Source:RefSeq\_peptide;Acc:NP\_620201] | 1 |
| 1392498\_at |  | 500722  691318 | ENSRNOG00000004973 | ENSRNOT00000045295  ENSRNOT00000041378  ENSRNOT00000007595  ENSRNOT00000007596 | LOC691318 |  | 1 |
| 1373336\_at |  | 293546 | ENSRNOG00000016013 | ENSRNOT00000021441 | NP\_001099774.1 | G protein-coupled receptor, family C, group 5, member B [Source:RefSeq\_peptide;Acc:NP\_001099774] | 1 |
| 1376132\_at | Q53E76 | 362129 | ENSRNOG00000031038 | ENSRNOT00000059679 | LOC362129 | Glycosyltransferase-like domain-containing protein 1. [Source:UniProtKB/Swiss-Prot;Acc:Q53E76] | 1 |
| 1367853\_at |  | 83629 | ENSRNOG00000015971 | ENSRNOT00000021921 | Slc12a2 | solute carrier family 12, member 2 [Source:RefSeq\_peptide;Acc:NP\_113986] | 1 |
| 1398606\_at | Q5BJK8 | 310526 | ENSRNOG00000024213 | ENSRNOT00000037115 | Golph4 | Golgi integral membrane protein 4 (Golgi phosphoprotein 4) (Golgi integral membrane protein, cis) (GIMPc). [Source:UniProtKB/Swiss-Prot;Acc:Q5BJK8] | 1 |
| 1377844\_at |  | 299194 | ENSRNOG00000038166 | ENSRNOT00000058095 | Zadh1 | zinc binding alcohol dehydrogenase, domain containing 1 [Source:RefSeq\_peptide;Acc:NP\_001015009] | 1 |
| 1392590\_at | Q5U2Z7 |  | ENSRNOG00000002095 | ENSRNOT00000002857 | Arhgap24 | Rho GTPase-activating protein 24 (Rho-type GTPase-activating protein 24) (Down-regulated in nephrectomized rat kidney #2). [Source:UniProtKB/Swiss-Prot;Acc:Q5U2Z7] | 1 |
| 1377643\_at |  | 303991 | ENSRNOG00000001581 | ENSRNOT00000002148 | NP\_001100564.1 | homeo box D10 [Source:RefSeq\_peptide;Acc:NP\_001100564] | 1 |
| 1398398\_at |  | 368057 | ENSRNOG00000027246 | ENSRNOT00000036394 | RGD1564605\_predicted |  | 1 |
| 1368240\_a\_at | P68403 | 25023 | ENSRNOG00000012061 | ENSRNOT00000016442  ENSRNOT00000016418 | Prkcb1 | Protein kinase C beta type (EC 2.7.11.13) (PKC-beta) (PKC-B). [Source:UniProtKB/Swiss-Prot;Acc:P68403] | 1 |
| 1389413\_at |  | 685433  688044 | ENSRNOG00000022764 | ENSRNOT00000039261 | LOC685433 | similar to ecotropic viral integration site 2A (LOC685433), mRNA [Source:RefSeq\_dna;Acc:NM\_001044287] | 1 |
| 1387112\_at |  |  | ENSRNOG00000002419 | ENSRNOT00000003298 | Plp | Myelin proteolipid protein (PLP) (Lipophilin). [Source:UniProtKB/Swiss-Prot;Acc:P60203] | 1 |
| 1392958\_at |  | 691222  312937 | ENSRNOG00000014048 | ENSRNOT00000060790  ENSRNOT00000060789 | LOC691222 | LRRGT00003. [Source:UniProtKB/TrEMBL;Acc:Q6TXJ6] | 1 |
| 1373254\_at | Q6IFW6 | 450225 | ENSRNOG00000030170 | ENSRNOT00000036023 | Krt10 | Keratin, type I cytoskeletal 10 (Cytokeratin-10) (CK-10) (Keratin-10) (K10) (Type I keratin Ka10). [Source:UniProtKB/Swiss-Prot;Acc:Q6IFW6] | 1 |
| 1388977\_at | Q6AXM5 | 310773 | ENSRNOG00000017723 | ENSRNOT00000036551 | Cept1 | Choline/ethanolaminephosphotransferase 1 (EC 2.7.8.1) (EC 2.7.8.2). [Source:UniProtKB/Swiss-Prot;Acc:Q6AXM5] | 1 |
| 1399101\_at |  | 362251 | ENSRNOG00000019848 | ENSRNOT00000026964 | Rnpc2 | RNA-binding region (RNP1, RRM) containing 2 [Source:RefSeq\_peptide;Acc:NP\_001013225] | 1 |
| 1379401\_a\_at |  |  | ENSRNOG00000005285 | ENSRNOT00000007221 | Fbxo33 | F-box protein 33 Gene [Source:MGI Symbol;Acc:MGI:1917861] | 1 |
| 1376106\_at | Q68FV0 | 362691 | ENSRNOG00000007907 | ENSRNOT00000010927 | MGC94782 | Transmembrane protein 178 precursor. [Source:UniProtKB/Swiss-Prot;Acc:Q68FV0] | 1 |
| 1381993\_at | Q5M883 | 294141 | ENSRNOG00000000728 | ENSRNOT00000000955 | Clic2 | Chloride intracellular channel protein 2. [Source:UniProtKB/Swiss-Prot;Acc:Q5M883] | 1 |
| 1393228\_at | Q5XIH9 | 313521 | ENSRNOG00000018143 | ENSRNOT00000024393 | Gloxd1 | 4-hydroxyphenylpyruvate dioxygenase-like protein (Glyoxalase domain- containing protein 1). [Source:UniProtKB/Swiss-Prot;Acc:Q5XIH9] | 1 |
| 1388304\_at |  | 294964 | ENSRNOG00000011949 | ENSRNOT00000016051 | NP\_001099896.1 | NADH dehydrogenase (ubiquinone) 1 beta subcomplex, 5 [Source:RefSeq\_peptide;Acc:NP\_001099896] | 1 |
| 1390867\_at |  | 691335  682750 | ENSRNOG00000012446 | ENSRNOT00000040110 | LOC682750 |  | 1 |
| 1375205\_at |  | 301164 | ENSRNOG00000011871 | ENSRNOT00000061882  ENSRNOT00000061881 | Pcaf | p300/CBP-associated factor [Source:RefSeq\_peptide;Acc:NP\_001019423] | 1 |
| 1373773\_at |  | 306439 | ENSRNOG00000010731 | ENSRNOT00000014312 | Gpm6a | glycoprotein m6a [Source:RefSeq\_peptide;Acc:NP\_835206] | 1 |
| 1389367\_at |  | 295105 | ENSRNOG00000009276 | ENSRNOT00000057473  ENSRNOT00000012525 | Schip1 | Schip1 protein (Fragment). [Source:UniProtKB/TrEMBL;Acc:Q562A8] | 1 |
| 1377868\_at |  | 317405 | ENSRNOG00000037658 | ENSRNOT00000004260 | RGD1561019\_predicted |  | 1 |
| 1372755\_at |  | 362911 | ENSRNOG00000008615 | ENSRNOT00000011485 | Mal2 | MAL2 proteolipid protein [Source:RefSeq\_peptide;Acc:NP\_942081] | 1 |
| 1369543\_s\_at |  | 192210 | ENSRNOG00000017876 | ENSRNOT00000024218 | Gs3 | putative regulation protein GS3 [Source:RefSeq\_peptide;Acc:NP\_620211] | 1 |
| 1376440\_at |  | 315000 | ENSRNOG00000008987 | ENSRNOT00000011891 | Rnf139\_predicted | TRC8 (Fragment). [Source:UniProtKB/TrEMBL;Acc:Q9JLC5] | 1 |
| 1389623\_at |  | 315305 | ENSRNOG00000032162 | ENSRNOT00000055390 | Atf1 | Atf1 protein (Fragment). [Source:UniProtKB/TrEMBL;Acc:Q5BK34] | 1 |
| 1391524\_at |  | 315843 | ENSRNOG00000008652 | ENSRNOT00000011864  ENSRNOT00000036864 | RGD1564964\_predicted |  | 1 |
| 1393267\_at | Q812D1 | 313323 | ENSRNOG00000011498 | ENSRNOT00000016130 | Psip1 | PC4 and SFRS1-interacting protein (Lens epithelium-derived growth factor). [Source:UniProtKB/Swiss-Prot;Acc:Q812D1] | 1 |
| 1390439\_at | Q5PQP2 | 299864 | ENSRNOG00000004220 | ENSRNOT00000005705 | Ebag9 | Receptor-binding cancer antigen expressed on SiSo cells (Estrogen receptor-binding fragment-associated gene 9 protein). [Source:UniProtKB/Swiss-Prot;Acc:Q5PQP2] | 1 |
| 1379257\_at |  | 307514 | ENSRNOG00000026050 | ENSRNOT00000038247 | NP\_001100867.1 | erythrocyte protein band 4.1-like 4a [Source:RefSeq\_peptide;Acc:NP\_001100867] | 1 |
| 1392506\_at |  | 288256 | ENSRNOG00000028014 | ENSRNOT00000033745 | Cryzl1 | crystallin, zeta (quinone reductase)-like 1 [Source:RefSeq\_peptide;Acc:NP\_001013062] | 1 |
| 1374768\_at | Q5XIJ8 | 362994 | ENSRNOG00000031424 | ENSRNOT00000051186  ENSRNOT00000046034 | LOC362994 | Coiled-coil domain-containing protein 65. [Source:UniProtKB/Swiss-Prot;Acc:Q5XIJ8] | 1 |
| 1384971\_at | Q4QR86 | 295971 | ENSRNOG00000012262 | ENSRNOT00000016677 | RGD1309720 | DEP domain-containing protein 7. [Source:UniProtKB/Swiss-Prot;Acc:Q4QR86] | 1 |
| 1392912\_at | Q6AYK6 | 289144 | ENSRNOG00000002572 | ENSRNOT00000003586 | Cacybp | Calcyclin-binding protein (CacyBP). [Source:UniProtKB/Swiss-Prot;Acc:Q6AYK6] | 1 |
| 1389685\_at |  | 360764 | ENSRNOG00000000982 | ENSRNOT00000001296 | Zfp655 | zinc finger protein 655 [Source:RefSeq\_peptide;Acc:NP\_001008363] | 1 |
| 1372675\_at |  | 288269 | ENSRNOG00000021399 | ENSRNOT00000030279 | RGD1306954 | similar to RIKEN cDNA 1110004E09 (RGD1306954), mRNA [Source:RefSeq\_dna;Acc:NM\_001008288] | 1 |
| 1368433\_at |  | 116482 | ENSRNOG00000005149 | ENSRNOT00000007223 | Sacm1l | SAC1 (supressor of actin mutations 1, homolog)-like [Source:RefSeq\_peptide;Acc:NP\_446250] | 1 |
| 1370201\_at | P07171 | 83839 | ENSRNOG00000007456 | ENSRNOT00000010845 | Calb1 | Calbindin (Vitamin D-dependent calcium-binding protein, avian-type) (Calbindin D28) (D-28K) (Spot 35 protein). [Source:UniProtKB/Swiss-Prot;Acc:P07171] | 1 |
| 1388192\_at |  | 65026 | ENSRNOG00000029893 | ENSRNOT00000040080 | Rwdd3 |  | 1 |
| 1367544\_at |  | 300813 | ENSRNOG00000012842 | ENSRNOT00000017918 | NP\_001100306.1 | ring finger protein 111 [Source:RefSeq\_peptide;Acc:NP\_001100306] | 1 |
| 1389338\_at |  | 293114 | ENSRNOG00000022732 | ENSRNOT00000038279 | NP\_001099750.1 |  | 1 |
| 1389651\_at | Q9R0R3 | 58812 | ENSRNOG00000003984 | ENSRNOT00000005331 | Apln | Apelin precursor (APJ endogenous ligand) [Contains: Apelin-36; Apelin- 31; Apelin-28; Apelin-13]. [Source:UniProtKB/Swiss-Prot;Acc:Q9R0R3] | 1 |
| 1395268\_at |  | 297930 | ENSRNOG00000006328 | ENSRNOT00000009047 | Wwp1 | WW domain containing E3 ubiquitin protein ligase 1 [Source:RefSeq\_peptide;Acc:NP\_001019928] | 1 |
| 1386962\_at | Q9QW07 | 25031 | ENSRNOG00000033119 | ENSRNOT00000045393  ENSRNOT00000042853 | Plcb4 | 1-phosphatidylinositol-4,5-bisphosphate phosphodiesterase beta-4 (EC 3.1.4.11) (Phosphoinositide phospholipase C) (Phospholipase C- beta-4) (PLC-beta-4). [Source:UniProtKB/Swiss-Prot;Acc:Q9QW07] | 1 |
| 1377616\_at |  | 297903 | ENSRNOG00000016338 | ENSRNOT00000022249 | RGD1310681\_predicted |  | 1 |
| 1393091\_at |  | 310086 | ENSRNOG00000013121 | ENSRNOT00000017571 | RGD1311241\_predicted |  | 1 |
| 1373147\_at |  | 306129 | ENSRNOG00000010102 | ENSRNOT00000057789 | Fbxl3 | Fbxl3 protein (Fragment). [Source:UniProtKB/TrEMBL;Acc:Q562A1] | 1 |
| 1369345\_at |  | 116699 | ENSRNOG00000018382 | ENSRNOT00000025013 | Inpp4b | Type II inositol-3,4-bisphosphate 4-phosphatase (EC 3.1.3.66) (Inositol polyphosphate 4-phosphatase type II). [Source:UniProtKB/Swiss-Prot;Acc:Q9QWG5] | 1 |
| 1379561\_at | Q505J9 | 309532 | ENSRNOG00000010861 | ENSRNOT00000014684 | Atad1 | ATPase family AAA domain-containing protein 1. [Source:UniProtKB/Swiss-Prot;Acc:Q505J9] | 1 |
| 1374508\_at |  | 688095  680233 | ENSRNOG00000015847 | ENSRNOT00000021328 | LOC688095 |  | 1 |
| 1376689\_at |  |  | ENSRNOG00000008247 | ENSRNOT00000010929 | RGD1305457 | similar to RIKEN cDNA 1700023M03 (RGD1305457), mRNA [Source:RefSeq\_dna;Acc:NM\_001009430] | 1 |
| 1374649\_at | P0C643 | 361714 | ENSRNOG00000021098 | ENSRNOT00000028646 | Rasgrp2\_predicted | RAS guanyl-releasing protein 2 (Calcium and DAG-regulated guanine nucleotide exchange factor I) (CalDAG-GEFI). [Source:UniProtKB/Swiss-Prot;Acc:P0C643] | 1 |
| 1384921\_at |  |  | ENSRNOG00000037146 | ENSRNOT00000056054 | BB181834 | Cortexin-2 [Source:UniProtKB/Swiss-Prot;Acc:Q3URE8] | 1 |
| 1383171\_at | Q5U2T7 | 289307 | ENSRNOG00000002695 | ENSRNOT00000003618 | Tfb2m | Mitochondrial dimethyladenosine transferase 2, mitochondrial precursor (EC 2.1.1.-) (S-adenosylmethionine-6-N', N'-adenosyl(rRNA) dimethyltransferase 2) (Mitochondrial 12S rRNA dimethylase 2) (Mitochondrial transcription factor B2) (mtTFB2). [Source:UniProtKB/Swiss-Prot;Acc:Q5U2T7] | 1 |
| 1387455\_a\_at | P98166 | 25696 | ENSRNOG00000027491 | ENSRNOT00000035814 | Vldlr | Very low-density lipoprotein receptor precursor (VLDL receptor) (VLDL- R). [Source:UniProtKB/Swiss-Prot;Acc:P98166] | 1 |
| 1367685\_at | P62982  P62989 | 498837  683631  81777  679976  681260  687748 | ENSRNOG00000020492  ENSRNOG00000027417  ENSRNOG00000033677  ENSRNOG00000004426  ENSRNOG00000034246  ENSRNOG00000037062  ENSRNOG00000018208 | ENSRNOT00000027780  ENSRNOT00000036265  ENSRNOT00000040289  ENSRNOT00000005872  ENSRNOT00000049535  ENSRNOT00000049610  ENSRNOT00000024516 | LOC366051  Rps27a  RS27A\_RAT | 40S ribosomal protein S27a. [Source:UniProtKB/Swiss-Prot;Acc:P62982] | 1 |
| 1384869\_at |  | 289440 | ENSRNOG00000023389 | ENSRNOT00000037101 | NP\_001099464.1 | abhydrolase domain containing 7 [Source:RefSeq\_peptide;Acc:NP\_001099464] | 1 |
| 1378656\_at |  | 362142 | ENSRNOG00000007127 | ENSRNOT00000009396 | NP\_001102053.1 | Bardet-Biedl syndrome 5 homolog [Source:RefSeq\_peptide;Acc:NP\_001102053] | 1 |
| 1383375\_at | Q4V888 | 362490 | ENSRNOG00000006697 | ENSRNOT00000009375 | Tmem55a | Transmembrane protein 55A (EC 3.1.3.-) (Type II phosphatidylinositol 4,5-bisphosphate 4-phosphatase) (PtdIns-4,5-P2 4-Ptase II). [Source:UniProtKB/Swiss-Prot;Acc:Q4V888] | 1 |
| 1382382\_at | Q5RJL0 | 295619 | ENSRNOG00000021472 | ENSRNOT00000034449 | RGD1308367 | Ermin (Juxtanodin) (JN). [Source:UniProtKB/Swiss-Prot;Acc:Q5RJL0] | 1 |
| 1370044\_at | Q8R5H8 | 140930 | ENSRNOG00000030069  ENSRNOG00000030463 | ENSRNOT00000046832  ENSRNOT00000047613  ENSRNOT00000045329  ENSRNOT00000045821 | Faim | Fas apoptotic inhibitory molecule 1 (rFAIM). [Source:UniProtKB/Swiss-Prot;Acc:Q8R5H8] | 1 |
| 1397556\_at |  | 288108 | ENSRNOG00000039017 | ENSRNOT00000059666  ENSRNOT00000059664 | NP\_001099351.1 | Mak3 homolog [Source:RefSeq\_peptide;Acc:NP\_001099351] | 1 |
| 1385229\_at |  | 306081 | ENSRNOG00000013306 | ENSRNOT00000017765 | NP\_001100750.1 | protocadherin 20 [Source:RefSeq\_peptide;Acc:NP\_001100750] | 1 |
| 1398401\_at |  | 306066 | ENSRNOG00000009034 | ENSRNOT00000012440 | Tdrd3 | tudor domain containing 3 [Source:RefSeq\_peptide;Acc:NP\_001012043] | 1 |
| 1389040\_at |  |  | ENSRNOG00000028411 | ENSRNOT00000035666 | LOC288165 | PEST proteolytic signal-containing nuclear protein (PEST-containing nuclear protein) (PCNP) (Liver regeneration-related protein LRRG084). [Source:UniProtKB/Swiss-Prot;Acc:Q7TP40] | 1 |
| 1375977\_at |  | 84593 | ENSRNOG00000017059 | ENSRNOT00000022941 | Cetn2 | Centrosomal protein centrin 2 (Fragment). [Source:UniProtKB/TrEMBL;Acc:Q99MJ7] | 1 |
| 1384236\_at |  |  | ENSRNOG00000009388 | ENSRNOT00000012422 | 1110032A04Rik | RIKEN cDNA 1110032A04 gene Gene [Source:MGI Symbol;Acc:MGI:1913433] | 1 |
| 1373051\_at | Q6AZ61 | 246046 | ENSRNOG00000012178 | ENSRNOT00000016887 | Lmbrd1 | LMBR1 domain-containing protein 1 (Liver regeneration p53-related protein). [Source:UniProtKB/Swiss-Prot;Acc:Q6AZ61] | 1 |
| 1368470\_at | Q62867 | 25455 | ENSRNOG00000007351 | ENSRNOT00000009758 | Ggh | Gamma-glutamyl hydrolase precursor (EC 3.4.19.9) (Gamma-Glu-X carboxypeptidase) (Conjugase) (GH). [Source:UniProtKB/Swiss-Prot;Acc:Q62867] | 1 |
| 1370459\_at |  | 246323 | ENSRNOG00000004708 | ENSRNOT00000006257 | Aard | A5D3 protein [Source:RefSeq\_peptide;Acc:NP\_659561] | 1 |
| 1367772\_at | Q04753 | 65160 | ENSRNOG00000012788 | ENSRNOT00000017702 | Clns1a | Methylosome subunit pICln (Chloride conductance regulatory protein ICln) (I(Cln)) (Chloride channel, nucleotide sensitive 1A). [Source:UniProtKB/Swiss-Prot;Acc:Q04753] | 1 |
| 1394412\_at |  |  | ENSRNOG00000004731 | ENSRNOT00000033427 | Tmem16c\_predicted |  | 1 |
| 1380403\_at |  | 363110 | ENSRNOG00000009599 | ENSRNOT00000012864 | NP\_001102243.1 | RWD domain containing 2 [Source:RefSeq\_peptide;Acc:NP\_001102243] | 1 |
| 1368151\_at | P43244 | 29150 | ENSRNOG00000019875 | ENSRNOT00000026949  ENSRNOT00000060707 | Matr3 | Matrin-3 (Nuclear scaffold protein P130/MAT3). [Source:UniProtKB/Swiss-Prot;Acc:P43244] | 1 |
| 1388458\_at |  | 288003 | ENSRNOG00000001816 | ENSRNOT00000002487 | NP\_001099339.1 | replication factor C (activator 1) 4 [Source:RefSeq\_peptide;Acc:NP\_001099339] | 1 |
| 1373016\_at | Q6P6R2 | 298942 | ENSRNOG00000006364 | ENSRNOT00000060075  ENSRNOT00000008980 | Dld | Dihydrolipoyl dehydrogenase, mitochondrial precursor (EC 1.8.1.4) (Dihydrolipoamide dehydrogenase). [Source:UniProtKB/Swiss-Prot;Acc:Q6P6R2] | 1 |
| 1370439\_a\_at |  | 246153 | ENSRNOG00000004077 | ENSRNOT00000005700  ENSRNOT00000005690 | Kcnc2 | Potassium voltage-gated channel subfamily C member 2 (Voltage-gated potassium channel subunit Kv3.2) (KSHIIIA). [Source:UniProtKB/Swiss-Prot;Acc:P22462] | 1 |
| 1370554\_at | Q91Y78 | 114094  679036  498560 | ENSRNOG00000009530  ENSRNOG00000006401 | ENSRNOT00000012842  ENSRNOT00000008390 | Uchl3  UCHL3\_RAT | similar to ubiqutin carboxyl-terminal hydrolase l3 (predicted) (RGD1561196\_predicted), mRNA [Source:RefSeq\_dna;Acc:NM\_001110165]  Ubiquitin carboxyl-terminal hydrolase isozyme L3 (EC 3.4.19.12) (UCH- L3) (Ubiquitin thioesterase L3). [Source:UniProtKB/Swiss-Prot;Acc:Q91Y78] | 1 |
| 1399058\_at |  | 292244 | ENSRNOG00000014582 | ENSRNOT00000020064 | NP\_001099675.1 | mitochondrial ribosomal protein L18 [Source:RefSeq\_peptide;Acc:NP\_001099675] | 1 |
| 1381922\_at |  | 252854 | ENSRNOG00000013407 | ENSRNOT00000018476  ENSRNOT00000036648 | Slc5a11 | Sodium-cotransporter rkST1 (Fragment). [Source:UniProtKB/TrEMBL;Acc:Q9Z1F2] | 1 |
| 1373152\_at | Q6AY61 | 308807 | ENSRNOG00000017307 | ENSRNOT00000023239 | Prss23 | Serine protease 23 precursor (EC 3.4.21.-). [Source:UniProtKB/Swiss-Prot;Acc:Q6AY61] | 1 |
| 1388504\_at |  | 314949 | ENSRNOG00000004420 | ENSRNOT00000006209 | MGC116373 | similar to HR21spA (MGC116373), mRNA [Source:RefSeq\_dna;Acc:NM\_001025701] | 1 |
| 1377867\_at |  | 313837 | ENSRNOG00000005705 | ENSRNOT00000007581  ENSRNOT00000049672 | RGD1562284\_predicted |  | 1 |
| 1373074\_at | Q5U2X7 | 307210 | ENSRNOG00000015142 | ENSRNOT00000020376 | RGD1307279 | TIM21-like protein, mitochondrial precursor. [Source:UniProtKB/Swiss-Prot;Acc:Q5U2X7] | 1 |
| 1392486\_at | Q6AYA5 | 312132 | ENSRNOG00000006206 | ENSRNOT00000008677 | Tmem106b | Transmembrane protein 106B. [Source:UniProtKB/Swiss-Prot;Acc:Q6AYA5] | 1 |
| 1389539\_at |  |  | ENSRNOG00000013574 | ENSRNOT00000018413 | RGD1309388\_predicted |  | 1 |
| 1388802\_at | Q3MKQ2 | 501625 | ENSRNOG00000033844 | ENSRNOT00000048996  ENSRNOT00000057110 | Bex1 | Protein BEX1 (Brain-expressed X-linked protein 1 homolog) (EG2RVC). [Source:UniProtKB/Swiss-Prot;Acc:Q3MKQ2] | 1 |
| 1384466\_at |  | 493574 | ENSRNOG00000006199 | ENSRNOT00000008312 | LOC493574 | notch1-induced protein [Source:RefSeq\_peptide;Acc:NP\_001008387] | 1 |
| 1390638\_at |  | 316539 | ENSRNOG00000013213 | ENSRNOT00000041689 | RGD1560587\_predicted |  | 1 |
| 1391005\_at |  | 313101 | ENSRNOG00000005641 | ENSRNOT00000061128 | NP\_001101389.1 | F-box and leucine-rich repeat protein 4 [Source:RefSeq\_peptide;Acc:NP\_001101389] | 1 |
| 1374585\_at | Q6AYG5 | 361465 | ENSRNOG00000011622 | ENSRNOT00000016358  ENSRNOT00000015440 | Echdc1 | Enoyl-CoA hydratase domain-containing protein 1. [Source:UniProtKB/Swiss-Prot;Acc:Q6AYG5] | 1 |
| 1367668\_a\_at | Q6P7B9 | 83792 | ENSRNOG00000013279 | ENSRNOT00000018090 | Scd2 | Acyl-CoA desaturase 2 (EC 1.14.19.1) (Stearoyl-CoA desaturase 2) (Fatty acid desaturase 2) (Delta(9)-desaturase 2). [Source:UniProtKB/Swiss-Prot;Acc:Q6P7B9] | 1 |
| 1373874\_at | Q99P55 | 81536 | ENSRNOG00000005175 | ENSRNOT00000006913 | Sgpp1 | Sphingosine-1-phosphate phosphatase 1 (EC 3.1.3.-) (Sphingosine-1- phosphatase 1) (SPPase1) (Spp1). [Source:UniProtKB/Swiss-Prot;Acc:Q99P55] | 1 |
| 1376587\_at | Q7TSL3 | 301674 | ENSRNOG00000016396 | ENSRNOT00000021998 | Fbxo11 | F-box only protein 11. [Source:UniProtKB/Swiss-Prot;Acc:Q7TSL3] | 1 |
| 1370360\_at | Q9QZX9 | 192229 | ENSRNOG00000024924 | ENSRNOT00000037795 | RGD621352 | Uncharacterized protein C3orf34 homolog. [Source:UniProtKB/Swiss-Prot;Acc:Q9QZX9] | 1 |
| 1376189\_at |  | 313604 | ENSRNOG00000013855 | ENSRNOT00000018520 | NP\_001101453.1 | zinc finger, MYM domain containing 1 [Source:RefSeq\_peptide;Acc:NP\_001101453] | 1 |
| 1377806\_at |  |  | ENSRNOG00000009819 | ENSRNOT00000013387 | Vezf1\_predicted | similar to splicing factor, arginine/serine-rich 1 (ASF/SF2) (LOC689890), mRNA [Source:RefSeq\_dna;Acc:NM\_001109552] | 1 |
| 1394022\_at |  | 291023 | ENSRNOG00000016099 | ENSRNOT00000021550 | Id4 | inhibitor of DNA binding 4 [Source:RefSeq\_peptide;Acc:NP\_783172] | 1 |
| 1379945\_at |  | 500262 | ENSRNOG00000013390 | ENSRNOT00000017889 | NP\_001102720.1 | similar to T-cell activation kelch repeat protein (predicted) (RGD1563166\_predicted), mRNA [Source:RefSeq\_dna;Acc:NM\_001109250] | 1 |
| 1376585\_at |  | 687870  685207  362517 | ENSRNOG00000007328 | ENSRNOT00000009678 | LOC687870 | mitochondrial ribosomal protein L50 [Source:RefSeq\_peptide;Acc:NP\_001102135] | 1 |
| 1388908\_at |  | 291075 | ENSRNOG00000016369 | ENSRNOT00000022022 | Peci | peroxisomal delta3, delta2-enoyl-Coenzyme A isomerase [Source:RefSeq\_peptide;Acc:NP\_001006967] | 1 |
| 1367870\_at | Q9JLZ1 | 58815 | ENSRNOG00000016227 | ENSRNOT00000022406 | Txnl2 | Glutaredoxin-3 (Thioredoxin-like protein 2) (PKC-interacting cousin of thioredoxin) (PKCq-interacting protein) (PKC-theta-interacting protein). [Source:UniProtKB/Swiss-Prot;Acc:Q9JLZ1] | 1 |
| 1368170\_at | P23978 | 79212 | ENSRNOG00000006527 | ENSRNOT00000009705 | Slc6a1 | Sodium- and chloride-dependent GABA transporter 1. [Source:UniProtKB/Swiss-Prot;Acc:P23978] | 1 |
| 1374413\_at | Q5BJT2 | 363869 | ENSRNOG00000000921 | ENSRNOT00000061229 | Ubl3 | Ubiquitin-like protein 3 precursor (Membrane-anchored ubiquitin-fold protein) (MUB). [Source:UniProtKB/Swiss-Prot;Acc:Q5BJT2] | 1 |
| 1372066\_at |  | 293058 | ENSRNOG00000019426 | ENSRNOT00000026385 | RGD1310022 |  | 1 |
| 1380170\_at | P60522 | 501441  64670 | ENSRNOG00000019425 | ENSRNOT00000026274  ENSRNOT00000048998 | Gabarapl2 | Gamma-aminobutyric acid receptor-associated protein-like 2 (GABA(A) receptor-associated protein-like 2) (Ganglioside expression factor 2) (GEF-2). [Source:UniProtKB/Swiss-Prot;Acc:P60522] | 1 |
| 1372710\_at | Q62896 | 29631 | ENSRNOG00000011008 | ENSRNOT00000014810 | Bet1 | BET1 homolog (Golgi vesicular membrane-trafficking protein p18) (rBET1). [Source:UniProtKB/Swiss-Prot;Acc:Q62896] | 1 |
| 1373896\_at | P21707 | 25716 | ENSRNOG00000006426 | ENSRNOT00000048880 | Syt1 | Synaptotagmin-1 (Synaptotagmin I) (SytI) (p65). [Source:UniProtKB/Swiss-Prot;Acc:P21707] | 1 |
| 1367808\_at | P62078 | 64372 | ENSRNOG00000009888 | ENSRNOT00000013188 | Timm8b | Mitochondrial import inner membrane translocase subunit Tim8 B (Deafness dystonia protein 2 homolog). [Source:UniProtKB/Swiss-Prot;Acc:P62078] | 1 |
| 1368344\_at | P18088 | 24379 | ENSRNOG00000000007 | ENSRNOT00000000008 | Gad1 | Glutamate decarboxylase 1 (EC 4.1.1.15) (Glutamate decarboxylase 67 kDa isoform) (GAD-67) (67 kDa glutamic acid decarboxylase). [Source:UniProtKB/Swiss-Prot;Acc:P18088] | 1 |
| 1373563\_at |  | 362392 | ENSRNOG00000017747 | ENSRNOT00000048399 | NP\_001102106.1 | similar to putative nucleic acid binding protein RY-1; EST AI449063 (predicted) (RGD1310925\_predicted), mRNA [Source:RefSeq\_dna;Acc:NM\_001108636] | 1 |
| 1388615\_at | P62836 | 295347 | ENSRNOG00000032463 | ENSRNOT00000047836 | Rap1a | Ras-related protein Rap-1A precursor (Ras-related protein Krev-1). [Source:UniProtKB/Swiss-Prot;Acc:P62836] | 1 |
| 1387436\_at | Q9WVC0 | 64551 | ENSRNOG00000000713  ENSRNOG00000006545 | ENSRNOT00000000903  ENSRNOT00000008838 | Sept7 | Septin-7 (CDC10 protein homolog). [Source:UniProtKB/Swiss-Prot;Acc:Q9WVC0] | 1 |
| 1387380\_at | O35458 | 83612 | ENSRNOG00000015393 | ENSRNOT00000020720 | Slc32a1 | Vesicular inhibitory amino acid transporter (GABA and glycine transporter) (Vesicular GABA transporter) (rGVAT) (Solute carrier family 32 member 1). [Source:UniProtKB/Swiss-Prot;Acc:O35458] | 1 |
| 1368105\_at | Q9JJW1 | 64521 | ENSRNOG00000023338 | ENSRNOT00000035605 | Tspan2 | Tetraspanin-2 (Tspan-2). [Source:UniProtKB/Swiss-Prot;Acc:Q9JJW1] | 1 |
| 1384285\_at | Q5XIE5 | 364878 | ENSRNOG00000023013 | ENSRNOT00000039177 | March3 | E3 ubiquitin-protein ligase MARCH3 (EC 6.3.2.-) (Membrane-associated RING finger protein 3) (Membrane-associated RING-CH protein III) (MARCH-III). [Source:UniProtKB/Swiss-Prot;Acc:Q5XIE5] | 1 |
| 1368275\_at | O35532 | 140910 | ENSRNOG00000032297 | ENSRNOT00000044171 | Sc4mol | C-4 methylsterol oxidase (EC 1.14.13.72) (Methylsterol monooxygenase) (Neuropep 1) (RANP-1). [Source:UniProtKB/Swiss-Prot;Acc:O35532] | 1 |
| 1385547\_at | Q5RJL0 | 295619 | ENSRNOG00000021472 | ENSRNOT00000034449 | RGD1308367 | Ermin (Juxtanodin) (JN). [Source:UniProtKB/Swiss-Prot;Acc:Q5RJL0] | 1 |
| 1371823\_at |  | 313304 | ENSRNOG00000029885 | ENSRNOT00000044240 | Stag2\_predicted |  | 1 |
| 1368643\_at | Q99MU5 | 171413 | ENSRNOG00000007568 | ENSRNOT00000010524 | Spata6 | Spermatogenesis-associated protein 6 precursor (Kinesin-related protein). [Source:UniProtKB/Swiss-Prot;Acc:Q99MU5] | 1 |
| 1369304\_at | P27213 | 29498 | ENSRNOG00000009250 | ENSRNOT00000012434 | Pts | 6-pyruvoyl tetrahydrobiopterin synthase precursor (EC 4.2.3.12) (PTPS) (PTP synthase). [Source:UniProtKB/Swiss-Prot;Acc:P27213] | 1 |
| 1377624\_at |  | 312054 | ENSRNOG00000007098 | ENSRNOT00000009340 | Gtpbp10 | GTP-binding protein 10 [Source:RefSeq\_peptide;Acc:NP\_001094285] | 1 |
| 1388523\_at | Q498E0 | 298370 | ENSRNOG00000008090 | ENSRNOT00000010700 | Txndc12 | Thioredoxin domain-containing protein 12 precursor (EC 1.8.4.2). [Source:UniProtKB/Swiss-Prot;Acc:Q498E0] | 1 |
| 1367802\_at | Q06226 | 29517 | ENSRNOG00000011815 | ENSRNOT00000040736  ENSRNOT00000061157  ENSRNOT00000016121 | Sgk | Serine/threonine-protein kinase Sgk1 (EC 2.7.11.1) (Serum/glucocorticoid-regulated kinase 1). [Source:UniProtKB/Swiss-Prot;Acc:Q06226] | 1 |
| 1390990\_at | P62142 | 25594 | ENSRNOG00000004612 | ENSRNOT00000006190 | Ppp1cb | Serine/threonine-protein phosphatase PP1-beta catalytic subunit (EC 3.1.3.16) (PP-1B). [Source:UniProtKB/Swiss-Prot;Acc:P62142] | 1 |
| 1393231\_at |  |  | ENSRNOG00000024647 | ENSRNOT00000029115 | Ppp4r2 | protein phosphatase 4, regulatory subunit 2 Gene [Source:MGI Symbol;Acc:MGI:3027896] | 1 |
| 1371360\_at | Q6JE36 | 299923 | ENSRNOG00000007393 | ENSRNOT00000010811  ENSRNOT00000036847 | Ndrg1 | Protein NDRG1 (N-myc downstream-regulated gene 1 protein) (Protein Ndr1). [Source:UniProtKB/Swiss-Prot;Acc:Q6JE36] | 1 |
| 1380828\_at | P62813 | 29705 | ENSRNOG00000003512 | ENSRNOT00000004725 | Gabra1 | Gamma-aminobutyric acid receptor subunit alpha-1 precursor (GABA(A) receptor subunit alpha-1). [Source:UniProtKB/Swiss-Prot;Acc:P62813] | 1 |
| 1367541\_at |  | 502632 | ENSRNOG00000008469 | ENSRNOT00000011303 | RGD1566062\_predicted |  | 1 |
| 1388563\_at |  | 294734 | ENSRNOG00000013002 | ENSRNOT00000036658 | NP\_001099880.1 | similar to RIKEN cDNA 1700034P14 (predicted) (RGD1305492\_predicted), mRNA [Source:RefSeq\_dna;Acc:NM\_001106410] | 1 |
| 1373044\_at |  | 295975 | ENSRNOG00000012738 | ENSRNOT00000017409 | RGD1565840\_predicted |  | 1 |
| 1368564\_at |  | 84487 | ENSRNOG00000016147 | ENSRNOT00000022383 | Slc17a6 | solute carrier family 17 (sodium-dependent inorganic phosphate cotransporter), member 6 [Source:RefSeq\_peptide;Acc:NP\_445879] | 1 |
| 1388827\_at |  | 689408  687799  289784  498894 | ENSRNOG00000038771  ENSRNOG00000007026 | ENSRNOT00000040085  ENSRNOT00000048052 | LOC689408  RGD1560813\_predicted | H2A histone family, member V [Source:RefSeq\_peptide;Acc:NP\_001099489] | 1 |
| 1373906\_at |  |  | ENSRNOG00000022347 | ENSRNOT00000046325 | Fam173b | family with sequence similarity 173, member B Gene [Source:MGI Symbol;Acc:MGI:1915323] | 1 |
| 1374953\_at |  | 500420 | ENSRNOG00000028185 | ENSRNOT00000030559 | LOC500420 | LOC500420 protein (Fragment). [Source:UniProtKB/TrEMBL;Acc:Q3SWT2] | 1 |
| 1367733\_at | P27139 | 54231 | ENSRNOG00000009629 | ENSRNOT00000013354 | Ca2 | Carbonic anhydrase 2 (EC 4.2.1.1) (Carbonic anhydrase II) (Carbonate dehydratase II) (CA-II). [Source:UniProtKB/Swiss-Prot;Acc:P27139] | 1 |
| 1388953\_at | Q811S9 | 290556 | ENSRNOG00000028461 | ENSRNOT00000033677  ENSRNOT00000061230 | Gnl3 | Guanine nucleotide-binding protein-like 3 (Nucleolar GTP-binding protein 3) (Nucleostemin). [Source:UniProtKB/Swiss-Prot;Acc:Q811S9] | 1 |
| 1388780\_at |  | 307861 | ENSRNOG00000010712 | ENSRNOT00000014660 | Terf2ip | telomeric repeat binding factor 2, interacting protein [Source:RefSeq\_peptide;Acc:NP\_001013161] | 1 |
| 1388896\_at |  |  | ENSRNOG00000011294 | ENSRNOT00000016290 | Pigk | phosphatidylinositol glycan anchor biosynthesis, class K [Source:RefSeq\_peptide;Acc:NP\_001011953] | 1 |
| 1398262\_at | P09330 | 24689 | ENSRNOG00000004160 | ENSRNOT00000005615 | Prps2 | Ribose-phosphate pyrophosphokinase 2 (EC 2.7.6.1) (Phosphoribosyl pyrophosphate synthetase II) (PRS-II). [Source:UniProtKB/Swiss-Prot;Acc:P09330] | 1 |
| 1371434\_at |  | 691807  686691 | ENSRNOG00000014654 | ENSRNOT00000019647 | LOC691807 | LOC691807 protein. [Source:UniProtKB/TrEMBL;Acc:Q498U0] | 1 |
| 1372827\_at | Q6DGG0 | 361967  501204  365587 | ENSRNOG00000039269  ENSRNOG00000027408  ENSRNOG00000037230 | ENSRNOT00000047599  ENSRNOT00000037890  ENSRNOT00000056255 | Ppid  RGD1560149\_predicted | 40 kDa peptidyl-prolyl cis-trans isomerase (EC 5.2.1.8) (PPIase) (Rotamase) (Cyclophilin-40) (CYP-40). [Source:UniProtKB/Swiss-Prot;Acc:Q6DGG0] | 1 |
| 1388135\_at | Q63528 | 59102 | ENSRNOG00000013005 | ENSRNOT00000017549  ENSRNOT00000041803 | Rpa2 | Replication protein A 32 kDa subunit (RP-A) (RF-A) (Replication factor-A protein 2) (p32). [Source:UniProtKB/Swiss-Prot;Acc:Q63528] | 1 |
| 1390803\_at |  | 317612 | ENSRNOG00000027761 | ENSRNOT00000038432 | NP\_001101729.1 | HIV TAT specific factor 1 [Source:RefSeq\_peptide;Acc:NP\_001101729] | 1 |
| 1382235\_at |  |  | ENSRNOG00000016691 | ENSRNOT00000060037 | RGD1306809\_predicted | UPF0465 protein C5orf33 homolog. [Source:UniProtKB/Swiss-Prot;Acc:Q1HCL7] | 1 |
| 1388410\_at |  | 289827 | ENSRNOG00000008079 | ENSRNOT00000010663 | Ugp2 | UDP-glucose pyrophosphorylase 2 [Source:RefSeq\_peptide;Acc:NP\_001019914] | 1 |
| 1394854\_at |  | 297758 | ENSRNOG00000007291 | ENSRNOT00000049741 | Terf1 | telomeric repeat binding factor 1 [Source:RefSeq\_peptide;Acc:NP\_001012482] | 1 |
| 1379307\_at |  | 266809 | ENSRNOG00000014139 | ENSRNOT00000019256 | Sap1 | sodium channel associated protein 1 [Source:RefSeq\_peptide;Acc:NP\_714962] | 1 |
| 1372927\_at |  | 687870  685207  362517 | ENSRNOG00000007328 | ENSRNOT00000009678 | LOC687870 | mitochondrial ribosomal protein L50 [Source:RefSeq\_peptide;Acc:NP\_001102135] | 1 |
| 1371420\_at |  | 494529 | ENSRNOG00000037087 | ENSRNOT00000055893 | LOC494529 | 92Aa-Protein [Source:RefSeq\_peptide;Acc:NP\_001009974] | 1 |
| 1373835\_at |  | 306436 | ENSRNOG00000010502 | ENSRNOT00000014015 | Fbxo8 | F-box only protein 8 [Source:RefSeq\_peptide;Acc:NP\_001012050] | 1 |
| 1395199\_at |  | 311371  691947 | ENSRNOG00000038907  ENSRNOG00000016459 | ENSRNOT00000059474  ENSRNOT00000059473  ENSRNOT00000022437 | Eif3s1\_predicted | eukaryotic translation initiation factor 3, subunit 1 alpha [Source:RefSeq\_peptide;Acc:NP\_001071138] | 1 |
| 1386439\_at |  | 311621 | ENSRNOG00000029799 | ENSRNOT00000040156 | Tomm34\_predicted | translocase of outer mitochondrial membrane 34 [Source:RefSeq\_peptide;Acc:NP\_001037709] | 1 |
| 1388491\_at |  | 294079 | ENSRNOG00000017396 | ENSRNOT00000023562 | NP\_001099842.1 | membrane-associated ring finger (C3HC4) 5 [Source:RefSeq\_peptide;Acc:NP\_001099842] | 1 |
| 1368194\_at | Q924S1 | 170919 | ENSRNOG00000017731 | ENSRNOT00000024213 | Agpat4 | 1-acyl-sn-glycerol-3-phosphate acyltransferase delta (EC 2.3.1.51) (1- AGP acyltransferase 4) (1-AGPAT 4) (Lysophosphatidic acid acyltransferase delta) (LPAAT-delta) (1-acylglycerol-3-phosphate O- acyltransferase 4). [Source:UniProtKB/Swiss-Prot;Acc:Q924S1] | 1 |
| 1373721\_at |  | 307491 | ENSRNOG00000016087 | ENSRNOT00000021797  ENSRNOT00000060537 | Zmat2 | zinc finger, matrin type 2 [Source:RefSeq\_peptide;Acc:NP\_001014034] | 1 |
| 1388800\_at |  | 64633 | ENSRNOG00000012180 | ENSRNOT00000016813 | Rab5a | RAB5A, member RAS oncogene family [Source:RefSeq\_peptide;Acc:NP\_073183] | 1 |
| 1388799\_at | Q5XHZ6 | 362303 | ENSRNOG00000010453 | ENSRNOT00000013932 | Klhl7 | Kelch-like protein 7. [Source:UniProtKB/Swiss-Prot;Acc:Q5XHZ6] | 1 |
| 1375884\_at | Q501L3 | 500941 | ENSRNOG00000022245 | ENSRNOT00000034482 | MGC105560 | Coiled-coil domain-containing protein KIAA1826 homolog. [Source:UniProtKB/Swiss-Prot;Acc:Q501L3] | 1 |
| 1398919\_at |  | 302247 | ENSRNOG00000000340 | ENSRNOT00000000382  ENSRNOT00000049255 | RGD1304704 | LRRGT00192. [Source:UniProtKB/TrEMBL;Acc:Q6QI16] | 1 |
| 1371577\_at | Q66HF1 | 301458 | ENSRNOG00000011849 | ENSRNOT00000015852 | Ndufs1 | NADH-ubiquinone oxidoreductase 75 kDa subunit, mitochondrial precursor (EC 1.6.5.3) (EC 1.6.99.3). [Source:UniProtKB/Swiss-Prot;Acc:Q66HF1] | 1 |
| 1372421\_at | P30919 | 290923 | ENSRNOG00000000108 | ENSRNOT00000000120 | Aga | N(4)-(Beta-N-acetylglucosaminyl)-L-asparaginase precursor (EC 3.5.1.26) (Glycosylasparaginase) (Aspartylglucosaminidase) (N4-(N- acetyl-beta-glucosaminyl)-L-asparagine amidase) (AGA) [Contains: Glycosylasparaginase alpha chain; Glycosylasparaginase beta c [Source:UniProtKB/Swiss-Prot;Acc:P30919] | 1 |
| 1377829\_at |  | 315423 | ENSRNOG00000006792 | ENSRNOT00000009318 | NP\_001101594.1 | centrosomal protein 57 [Source:RefSeq\_peptide;Acc:NP\_001101594] | 1 |
| 1378964\_at | Q6P5P3 | 309196 | ENSRNOG00000019464 | ENSRNOT00000026376 | Ttc9c | Tetratricopeptide repeat protein 9C (TPR repeat protein 9C). [Source:UniProtKB/Swiss-Prot;Acc:Q6P5P3] | 1 |
| 1398642\_at |  |  | ENSRNOG00000017826 | ENSRNOT00000024041 | Mtrr | 5-methyltetrahydrofolate-homocysteine methyltransferase reductase [Source:RefSeq\_peptide;Acc:NP\_001034092] | 1 |
| 1399130\_at | Q5U1Z8 | 502782 | ENSRNOG00000006858 | ENSRNOT00000008973 | LOC502782 | Protein preY, mitochondrial precursor. [Source:UniProtKB/Swiss-Prot;Acc:Q5U1Z8] | 1 |
| 1389197\_at |  | 288091 | ENSRNOG00000003014 | ENSRNOT00000051428 | RGD1306248 | RGD1306248 protein (Fragment). [Source:UniProtKB/TrEMBL;Acc:Q5BJN4] | 1 |
| 1387200\_at | Q9WUQ3 | 60394 | ENSRNOG00000028648 | ENSRNOT00000002827 | Olig1 | Oligodendrocyte transcription factor 1 (Oligo1) (Olg-1 bHLH protein). [Source:UniProtKB/Swiss-Prot;Acc:Q9WUQ3] | 1 |
| 1373054\_at |  |  | ENSRNOG00000017846 | ENSRNOT00000024354 | Cdw92 | Choline transporter-like protein 1 (Solute carrier family 44 member 1) (CD92 antigen). [Source:UniProtKB/Swiss-Prot;Acc:Q8VII6] | 1 |
| 1388022\_a\_at | O35303 | 114114 | ENSRNOG00000001813 | ENSRNOT00000002478  ENSRNOT00000002477  ENSRNOT00000002479  ENSRNOT00000002482  ENSRNOT00000002485 | Dnm1l | Dynamin-1-like protein (EC 3.6.5.5) (Dynamin-like protein). [Source:UniProtKB/Swiss-Prot;Acc:O35303] | 1 |
| 1374503\_at |  | 311876 | ENSRNOG00000022162 | ENSRNOT00000029600 | NP\_001101304.1 | pre B-cell leukemia transcription factor 3 [Source:RefSeq\_peptide;Acc:NP\_001101304] | 1 |
| 1371379\_at | P38718 | 289182 | ENSRNOG00000003150 | ENSRNOT00000004206 | RGD1563422\_predicted | Brain protein 44 (Protein 0-44). [Source:UniProtKB/Swiss-Prot;Acc:P38718] | 1 |
| 1376285\_at | Q5PQS4 | 314543 | ENSRNOG00000003242 | ENSRNOT00000004463 | Gulp1 | PTB domain-containing engulfment adapter protein 1 (GULP) (PTB domain adapter protein CED-6) (Cell death protein 6 homolog). [Source:UniProtKB/Swiss-Prot;Acc:Q5PQS4] | 1 |
| 1374233\_at |  | 308009 | ENSRNOG00000010230 | ENSRNOT00000013845 | RGD1308326\_predicted |  | 1 |
| 1388141\_at |  | 170895 | ENSRNOG00000015711 | ENSRNOT00000021735 | Cetn3 | Centrosomal protein centrin 3 (Fragment). [Source:UniProtKB/TrEMBL;Acc:Q91ZZ8] | 1 |
| 1392891\_at |  |  | ENSRNOG00000001098 | ENSRNOT00000001464 | Pds5b | PDS5, regulator of cohesion maintenance, homolog B (S. cerevisiae) Gene [Source:MGI (curated);Acc:Pds5b-001] | 1 |
| 1383271\_at |  | 314799 | ENSRNOG00000004183 | ENSRNOT00000005740 | NP\_001101560.1 | coiled-coil domain containing 59 [Source:RefSeq\_peptide;Acc:NP\_001101560] | 1 |
| 1389569\_at | Q4QQT6 | 294799 | ENSRNOG00000018021 | ENSRNOT00000024285 | Bxdc2 | Brix domain-containing protein 2 (Ribosome biogenesis protein Brix). [Source:UniProtKB/Swiss-Prot;Acc:Q4QQT6] | 1 |
| 1389257\_at |  | 362375 | ENSRNOG00000006810 | ENSRNOT00000008948 | Lancl2\_predicted | LanC lantibiotic synthetase component C-like 2 [Source:RefSeq\_peptide;Acc:NP\_001014209] | 1 |
| 1379784\_at |  | 308718 | ENSRNOG00000012322 | ENSRNOT00000016728 | Pex7 | peroxisome biogenesis factor 7 [Source:RefSeq\_peptide;Acc:NP\_001029319] | 1 |
| 1398937\_at |  | 289693 | ENSRNOG00000003844 | ENSRNOT00000005126 | Dhx15\_predicted |  | 1 |
| 1373093\_at | P05432 | 313729 | ENSRNOG00000018275 | ENSRNOT00000024606 | Errfi1 | ERBB receptor feedback inhibitor 1 (Mitogen-inducible gene 6 protein homolog) (Mig-6) (Gene 33 polypeptide). [Source:UniProtKB/Swiss-Prot;Acc:P05432] | 1 |
| 1389455\_at |  | 295461 | ENSRNOG00000023373 | ENSRNOT00000038509 | NP\_001099944.1 | SEC24 related gene family, member B [Source:RefSeq\_peptide;Acc:NP\_001099944] | 1 |
| 1369004\_at | P51156 | 171111 | ENSRNOG00000003131 | ENSRNOT00000004249 | Rab26 | Ras-related protein Rab-26. [Source:UniProtKB/Swiss-Prot;Acc:P51156] | 1 |
| 1373151\_at | Q5BJS2 | 499615 | ENSRNOG00000014153 | ENSRNOT00000019057 | LHFP\_RAT | Lipoma HMGIC fusion partner precursor. [Source:UniProtKB/Swiss-Prot;Acc:Q5BJS2] | 1 |
| 1398883\_at |  | 500008 | ENSRNOG00000021254 | ENSRNOT00000061083 | RGD1562992\_predicted |  | 1 |
| 1373084\_at |  |  | ENSRNOG00000007333 | ENSRNOT00000009810 | Wdr20 | Wdr20 protein (Fragment). [Source:UniProtKB/TrEMBL;Acc:Q5BJW6] | 1 |
| 1398364\_at | Q5U2S0 | 362626 | ENSRNOG00000017309 | ENSRNOT00000023289  ENSRNOT00000056120 | RGD1359529 | UPF0471 protein C1orf63 homolog. [Source:UniProtKB/Swiss-Prot;Acc:Q5U2S0] | 1 |
| 1390127\_at | Q2VUH7 | 363062 | ENSRNOG00000010260 | ENSRNOT00000039045 | Dixdc1 | Dixin (DIX domain-containing protein 1) (Coiled-coil protein DIX1) (Coiled-coil-DIX1). [Source:UniProtKB/Swiss-Prot;Acc:Q2VUH7] | 1 |
| 1371386\_at |  | 683420  690938 | ENSRNOG00000016779 | ENSRNOT00000022539 | RGD1306643\_predicted |  | 1 |
| 1372878\_at | Q562A2 |  | ENSRNOG00000011627 | ENSRNOT00000016196 | ZFR\_RAT | Zinc finger RNA-binding protein. [Source:UniProtKB/Swiss-Prot;Acc:Q562A2] | 1 |
| 1391776\_at |  | 363162 | ENSRNOG00000010149 | ENSRNOT00000013467 | RGD1305283\_predicted |  | 1 |
| 1373383\_at | Q6P6Q6 | 299514 | ENSRNOG00000004492 | ENSRNOT00000006185 | Mterfd1 | mTERF domain-containing protein 1, mitochondrial precursor. [Source:UniProtKB/Swiss-Prot;Acc:Q6P6Q6] | 1 |
| 1384295\_at | Q6AYB4 | 307133 | ENSRNOG00000015212 | ENSRNOT00000020854  ENSRNOT00000057922 | Hspa14 | Heat shock 70 kDa protein 14. [Source:UniProtKB/Swiss-Prot;Acc:Q6AYB4] | 1 |
| 1377785\_at | Q5XI69 | 287595 | ENSRNOG00000004549 | ENSRNOT00000006926 | Dhx40 | Probable ATP-dependent RNA helicase DHX40 (EC 3.6.1.-) (DEAH box protein 40). [Source:UniProtKB/Swiss-Prot;Acc:Q5XI69] | 1 |
| 1374638\_at |  | 305581 | ENSRNOG00000005601 | ENSRNOT00000007853 | NP\_001100712.1 | peroxisomal biogenesis factor 13 [Source:RefSeq\_peptide;Acc:NP\_001100712] | 1 |
| 1367775\_at | P70473 | 25284 | ENSRNOG00000018662 | ENSRNOT00000040701  ENSRNOT00000025323 | Amacr | Alpha-methylacyl-CoA racemase (EC 5.1.99.4) (2-methylacyl-CoA racemase) (2-arylpropionyl-CoA epimerase). [Source:UniProtKB/Swiss-Prot;Acc:P70473] | 1 |
| 1373719\_at |  |  | ENSRNOG00000007172 | ENSRNOT00000010311 | Map4k3 | Mitogen-activated protein kinase kinase kinase kinase 3 (EC 2.7.11.1) (MAPK/ERK kinase kinase kinase 3) (MEK kinase kinase 3) (MEKKK 3) (Germinal center kinase-related protein kinase) (GLK). [Source:UniProtKB/Swiss-Prot;Acc:Q924I2] | 1 |
| 1376883\_at | Q66HC4 | 295663 | ENSRNOG00000007979 | ENSRNOT00000010489 | MGC93920 | Pyridoxal phosphate phosphatase PHOSPHO2 (EC 3.1.3.74). [Source:UniProtKB/Swiss-Prot;Acc:Q66HC4] | 1 |
| 1370426\_a\_at | P11507 | 29693 | ENSRNOG00000001285 | ENSRNOT00000024347 | Atp2a2 | Sarcoplasmic/endoplasmic reticulum calcium ATPase 2 (EC 3.6.3.8) (Calcium pump 2) (SERCA2) (SR Ca(2+)-ATPase 2) (Calcium-transporting ATPase sarcoplasmic reticulum type, slow twitch skeletal muscle isoform) (Endoplasmic reticulum class 1/2 Ca(2+) ATPase). [Source:UniProtKB/Swiss-Prot;Acc:P11507] | 1 |
| 1388755\_at |  | 58817 | ENSRNOG00000004657 | ENSRNOT00000006369 | NP\_001099202.1 | SEC23A (S. cerevisiae) (predicted) [Source:RefSeq\_peptide;Acc:NP\_001099202] | 1 |
| 1398257\_at | Q63345 | 24558 | ENSRNOG00000000775 | ENSRNOT00000001008 | Mog | Myelin-oligodendrocyte glycoprotein precursor. [Source:UniProtKB/Swiss-Prot;Acc:Q63345] | 1 |
| 1371657\_at |  | 308508 | ENSRNOG00000021113 | ENSRNOT00000028672 | Q4G010\_RAT | Uble1b protein (Fragment). [Source:UniProtKB/TrEMBL;Acc:Q4G010] | 1 |
| 1376985\_at |  |  | ENSRNOG00000002556 | ENSRNOT00000003540 | Tsr2 | TSR2, 20S rRNA accumulation, homolog (S. cerevisiae) Gene [Source:MGI (curated);Acc:Tsr2-001] | 1 |
| 1383570\_at |  | 363210 | ENSRNOG00000011756 | ENSRNOT00000015638 | NP\_001102261.1 | PHD finger protein 3 [Source:RefSeq\_peptide;Acc:NP\_001102261] | 1 |
| 1398255\_at | Q63424 | 60577 | ENSRNOG00000002305 | ENSRNOT00000003189 | Slc15a2 | Oligopeptide transporter, kidney isoform (Peptide transporter 2) (Kidney H(+)/peptide cotransporter) (Solute carrier family 15 member 2). [Source:UniProtKB/Swiss-Prot;Acc:Q63424] | 1 |
| 1383144\_at |  | 687406  308879  312616 | ENSRNOG00000006432 | ENSRNOT00000008526 | Trnt1 | tRNA nucleotidyl transferase, CCA-adding, 1 [Source:RefSeq\_peptide;Acc:NP\_001019432] | 1 |
| 1399135\_at | Q68FQ7 | 300189 | ENSRNOG00000007065 | ENSRNOT00000009326 | MGC94954 | RNA polymerase II-associated protein 3. [Source:UniProtKB/Swiss-Prot;Acc:Q68FQ7] | 1 |
| 1386979\_at |  | 170907 | ENSRNOG00000024039 | ENSRNOT00000038685 | Tpo1 | developmentally regulated protein TPO1 [Source:RefSeq\_peptide;Acc:NP\_596886] | 1 |
| 1379243\_at |  | 315167 | ENSRNOG00000008569 | ENSRNOT00000011484 | Ndufa6\_predicted |  | 1 |
| 1372709\_at |  | 298943 | ENSRNOG00000007884 | ENSRNOT00000010631 | Bcap29 | B-cell receptor-associated protein 29 [Source:RefSeq\_peptide;Acc:NP\_001006981] | 1 |
| 1392979\_at | Q6AYK6 | 289144 | ENSRNOG00000002572 | ENSRNOT00000003586 | Cacybp | Calcyclin-binding protein (CacyBP). [Source:UniProtKB/Swiss-Prot;Acc:Q6AYK6] | 1 |
| 1374086\_at |  | 307178 | ENSRNOG00000008659 | ENSRNOT00000011448 | Arhgap21\_predicted |  | 1 |
| 1374846\_at | Q5M9G1 | 498008 | ENSRNOG00000003203 | ENSRNOT00000004272 | Clp1 | Protein HEXIM1 (Cardiac lineage protein 1). [Source:UniProtKB/Swiss-Prot;Acc:Q5M9G1] | 1 |
| 1393081\_at |  |  | ENSRNOG00000031671 | ENSRNOT00000051804 | Rasgef1a | RasGEF domain family, member 1A Gene [Source:MGI Symbol;Acc:MGI:1917977] | 1 |
| 1388307\_at | Q7TNK0 | 294421 | ENSRNOG00000029360 | ENSRNOT00000050552  ENSRNOT00000001066 | Serinc1 | Serine incorporator 1 (Tumor differentially expressed protein 2) (Tumor differentially expressed 1 protein-like). [Source:UniProtKB/Swiss-Prot;Acc:Q7TNK0] | 1 |
| 1386023\_at | Q8K4Y5 | 252892 | ENSRNOG00000014758 | ENSRNOT00000020411 | Lgi1 | Leucine-rich glioma-inactivated protein 1 precursor. [Source:UniProtKB/Swiss-Prot;Acc:Q8K4Y5] | 1 |
| 1370817\_at | Q9WTR7 | 266758 | ENSRNOG00000017036 | ENSRNOT00000022958 | Sec11l3 | Signal peptidase complex catalytic subunit SEC11C (EC 3.4.-.-) (SEC11 homolog C) (SEC11-like protein 3) (Microsomal signal peptidase 21 kDa subunit) (SPase 21 kDa subunit) (SPC21). [Source:UniProtKB/Swiss-Prot;Acc:Q9WTR7] | 1 |
| 1372205\_at |  |  | ENSRNOG00000018709 | ENSRNOT00000025481 | NP\_001100701.1 | zinc finger protein 278 [Source:RefSeq\_peptide;Acc:NP\_001100701] | 1 |
| 1389521\_at |  | 289089 | ENSRNOG00000002618 | ENSRNOT00000059863 | Ivns1abp\_predicted | influenza virus NS1A binding protein [Source:RefSeq\_peptide;Acc:NP\_001040550] | 1 |
| 1368262\_at | Q9WTR8 | 59265 | ENSRNOG00000002821 | ENSRNOT00000003840 | Phlpp | PH domain leucine-rich repeat protein phosphatase (EC 3.1.3.16) (Pleckstrin homology domain-containing family E protein 1) (Suprachiasmatic nucleus circadian oscillatory protein) (Phlpp1). [Source:UniProtKB/Swiss-Prot;Acc:Q9WTR8] | 1 |
| 1387152\_at | Q9QYK3 | 58839  499670 | ENSRNOG00000000641  ENSRNOG00000030494 | ENSRNOT00000000794  ENSRNOT00000056444 | Nrbf2  RGD1560263\_predicted | Nuclear receptor-binding factor 2 (NRBF-2). [Source:UniProtKB/Swiss-Prot;Acc:Q9QYK3] | 1 |
| 1370387\_at | P51538 | 171352 | ENSRNOG00000001379 | ENSRNOT00000001863 | Cyp3a9 | Cytochrome P450 3A9 (EC 1.14.14.1) (CYPIIIA9) (P450-OLF3) (Olfactive) (3AH15). [Source:UniProtKB/Swiss-Prot;Acc:P51538] | 1 |
| 1389163\_at |  | 313264 | ENSRNOG00000010303 | ENSRNOT00000013675 | Trim32 | tripartite motif protein 32 [Source:RefSeq\_peptide;Acc:NP\_001012103] | 1 |
| 1371323\_at |  |  | ENSRNOG00000005512 | ENSRNOT00000007567 | Ndufa4 | NADH dehydrogenase (ubiquinone) 1 alpha subcomplex, 4 Gene [Source:MGI Symbol;Acc:MGI:107686] | 1 |
| 1372343\_at |  | 295050 | ENSRNOG00000013434 | ENSRNOT00000017993 | NP\_001099902.1 | exosome component 8 [Source:RefSeq\_peptide;Acc:NP\_001099902] | 1 |
| 1386632\_at | Q4V888 | 362490 | ENSRNOG00000006697 | ENSRNOT00000009375 | Tmem55a | Transmembrane protein 55A (EC 3.1.3.-) (Type II phosphatidylinositol 4,5-bisphosphate 4-phosphatase) (PtdIns-4,5-P2 4-Ptase II). [Source:UniProtKB/Swiss-Prot;Acc:Q4V888] | 1 |
| 1376711\_at | Q99P82 | 84588 | ENSRNOG00000010263 | ENSRNOT00000014359 | Cldn11 | Claudin-11. [Source:UniProtKB/Swiss-Prot;Acc:Q99P82] | 1 |
| 1398968\_at | Q5RKH0 | 360477 | ENSRNOG00000003065 | ENSRNOT00000004159 | RGD1309459 | Nuclear protein NP60. [Source:UniProtKB/Swiss-Prot;Acc:Q5RKH0] | 1 |
| 1388431\_at |  | 361295 | ENSRNOG00000016800 | ENSRNOT00000041836  ENSRNOT00000032454 | Ss18 | Ss18 protein (Fragment). [Source:UniProtKB/TrEMBL;Acc:Q5XI66] | 1 |
| 1398895\_at | Q6AYQ1 | 361171 | ENSRNOG00000017956 | ENSRNOT00000030960 | Golga7 | Golgin subfamily A member 7. [Source:UniProtKB/Swiss-Prot;Acc:Q6AYQ1] | 1 |
| 1373957\_at |  |  | ENSRNOG00000021441 | ENSRNOT00000061858 | Reln | Reelin precursor (EC 3.4.21.-). [Source:UniProtKB/Swiss-Prot;Acc:P58751] | 1 |
| 1382080\_at |  | 498530 | ENSRNOG00000009984 | ENSRNOT00000013287 | RGD1563689\_predicted |  | 1 |
| 1398891\_at |  |  | ENSRNOG00000008566 | ENSRNOT00000011619 | NP\_001100103.1 | mitochondrial ribosomal protein L15 [Source:RefSeq\_peptide;Acc:NP\_001100103] | 1 |
| 1374602\_at |  | 29544 | ENSRNOG00000000549 | ENSRNOT00000000660 | Tspyl | testis-specific protein, Y-encoded-like [Source:RefSeq\_peptide;Acc:NP\_001013051] | 1 |
| 1398374\_at | Q5FVI3 | 311346 | ENSRNOG00000009143 | ENSRNOT00000012636 | RGD1307128 | Leucine-rich repeat-containing protein 57. [Source:UniProtKB/Swiss-Prot;Acc:Q5FVI3] | 1 |
| 1385868\_at |  | 302405 | ENSRNOG00000022267 | ENSRNOT00000057640 | RGD1566367\_predicted |  | 1 |
| 1375024\_at |  | 499731 | ENSRNOG00000028328 | ENSRNOT00000032874 | RGD1559623\_predicted |  | 1 |
| 1369070\_at | O88177 | 116718 | ENSRNOG00000009718 | ENSRNOT00000013233 | Pex12 | Peroxisome assembly protein 12 (Peroxin-12) (Peroxisome assembly factor 3) (PAF-3). [Source:UniProtKB/Swiss-Prot;Acc:O88177] | 1 |
| 1382057\_at |  | 361773 | ENSRNOG00000017018 | ENSRNOT00000022886 | RGD1307158\_predicted |  | 1 |
| 1371320\_at | Q5XIE8 | 290364 | ENSRNOG00000016271 | ENSRNOT00000023037 | Itm2b | Integral membrane protein 2B. [Source:UniProtKB/Swiss-Prot;Acc:Q5XIE8] | 1 |
| 1388368\_at | P68943 | 305391 | ENSRNOG00000003592 | ENSRNOT00000004912 | MED28\_RAT | Mediator of RNA polymerase II transcription subunit 28 (Mediator complex subunit 28). [Source:UniProtKB/Swiss-Prot;Acc:P68943] | 1 |
| 1389164\_at |  | 362377 | ENSRNOG00000007304 | ENSRNOT00000010241 | NP\_001102101.1 | hect domain and RLD 3 [Source:RefSeq\_peptide;Acc:NP\_001102101] | 1 |
| 1367606\_at | P49242 | 29288 | ENSRNOG00000011893  ENSRNOG00000004732 | ENSRNOT00000016329  ENSRNOT00000006275 | Rps3a | 40S ribosomal protein S3a (V-fos transformation effector protein) [Contains: 40S ribosomal protein S3b]. [Source:UniProtKB/Swiss-Prot;Acc:P49242] | 1 |
| 1394966\_at |  | 310812 | ENSRNOG00000016878 | ENSRNOT00000022905 | NP\_001101190.1 |  | 1 |
| 1371415\_at | Q5M9I5 | 366448 | ENSRNOG00000012550 | ENSRNOT00000016751 | Uqcrh | Cytochrome b-c1 complex subunit 6, mitochondrial precursor (Ubiquinol- cytochrome c reductase complex 11 kDa protein) (Cytochrome c1 non-heme 11 kDa protein) (Mitochondrial hinge protein) (Complex III subunit VIII) (Complex III subunit 6). [Source:UniProtKB/Swiss-Prot;Acc:Q5M9I5] | 1 |
| 1379290\_at |  | 300963 | ENSRNOG00000008410 | ENSRNOT00000056531  ENSRNOT00000031313 | LOC300963 | similar to centrosome protein Cep63 (LOC300963), mRNA [Source:RefSeq\_dna;Acc:NM\_001037772] | 1 |
| 1372506\_at |  | 287716 | ENSRNOG00000024118  ENSRNOG00000036806 | ENSRNOT00000038391  ENSRNOT00000027957 | Psme3 | proteaseome (prosome, macropain) 28 subunit, 3 [Source:RefSeq\_peptide;Acc:NP\_001011894] | 1 |
| 1368712\_at |  | 25165 | ENSRNOG00000004268 | ENSRNOT00000005697  ENSRNOT00000005648 | Znf386 | zinc finger protein 386 (Kruppel-like) [Source:RefSeq\_peptide;Acc:NP\_062566] | 1 |
| 1388753\_at |  | 311642 | ENSRNOG00000006052 | ENSRNOT00000008478  ENSRNOT00000051675 | Sulf2 | sulfatase 2 [Source:RefSeq\_peptide;Acc:NP\_001030099] | 1 |
| 1374156\_at |  | 314259 | ENSRNOG00000008788 | ENSRNOT00000012005 | NP\_001101504.1 | membrane protein, palmitoylated 5 (MAGUK p55 subfamily member 5) [Source:RefSeq\_peptide;Acc:NP\_001101504] | 1 |
| 1371301\_at | P17077 | 29257  314434  305275  306511  366500 | ENSRNOG00000030476  ENSRNOG00000032054  ENSRNOG00000033207  ENSRNOG00000030809  ENSRNOG00000030621  ENSRNOG00000026716  ENSRNOG00000031879  ENSRNOG00000011346  ENSRNOG00000038240  ENSRNOG00000029056  ENSRNOG00000031087 | ENSRNOT00000042201  ENSRNOT00000042622  ENSRNOT00000042106  ENSRNOT00000048551  ENSRNOT00000041324  ENSRNOT00000038927  ENSRNOT00000042609  ENSRNOT00000052231  ENSRNOT00000058215  ENSRNOT00000041351  ENSRNOT00000054878 | RGD1559948\_predicted  RGD1559566\_predicted    RGD1561987\_predicted  Ehd2  RGD1561789\_predicted | 60S ribosomal protein L9. [Source:UniProtKB/Swiss-Prot;Acc:P17077] | 1 |
| 1398268\_at | Q62725 | 25337 | ENSRNOG00000010735 | ENSRNOT00000056651 | Nfyc | Nuclear transcription factor Y subunit gamma (Nuclear transcription factor Y subunit C) (NF-YC) (CAAT-box DNA-binding protein subunit C) (CCAAT-binding transcription factor subunit C) (CBF-C). [Source:UniProtKB/Swiss-Prot;Acc:Q62725] | 1 |
| 1388873\_at |  |  | ENSRNOG00000016963 | ENSRNOT00000022873  ENSRNOT00000022822 | Q3KR60\_RAT | Trip12 protein. [Source:UniProtKB/TrEMBL;Acc:Q3KR60] | 1 |
| 1367777\_at | Q64591 | 117543  683217  685471 | ENSRNOG00000008236  ENSRNOG00000038203 | ENSRNOT00000011330  ENSRNOT00000058168  ENSRNOT00000058167 | Decr1  LOC685471 | 2,4-dienoyl-CoA reductase, mitochondrial precursor (EC 1.3.1.34) (2,4- dienoyl-CoA reductase [NADPH]) (4-enoyl-CoA reductase [NADPH]). [Source:UniProtKB/Swiss-Prot;Acc:Q64591] | 1 |
| 1387896\_at | P11915 | 25541 | ENSRNOG00000011413 | ENSRNOT00000015420 | Scp2 | Non-specific lipid-transfer protein (EC 2.3.1.176) (Propanoyl-CoA C- acyltransferase) (NSL-TP) (Sterol carrier protein 2) (SCP-2) (Sterol carrier protein X) (SCP-X) (SCP-chi) (SCPX). [Source:UniProtKB/Swiss-Prot;Acc:P11915] | 1 |
| 1396025\_at |  | 310780 | ENSRNOG00000019738 | ENSRNOT00000026750 | NM\_001107715.1 | G protein-coupled receptor 61 (predicted) (Gpr61\_predicted), mRNA [Source:RefSeq\_dna;Acc:NM\_001107715] | 1 |
| 1368122\_at | Q9EPZ8 | 84508 | ENSRNOG00000007272 | ENSRNOT00000009622 | Rnf103 | RING finger protein 103 (Zinc finger protein 103) (Zfp-103) (Protein ADRG34). [Source:UniProtKB/Swiss-Prot;Acc:Q9EPZ8] | 1 |
| 1377192\_a\_at |  | 300786 | ENSRNOG00000030225 | ENSRNOT00000048302 | Clpx | caseinolytic peptidase X [Source:RefSeq\_peptide;Acc:NP\_001007804] | 1 |
| 1384023\_at |  | 317395 | ENSRNOG00000031286 | ENSRNOT00000052281 | RGD1560898\_predicted |  | 1 |
| 1391673\_at | Q6REY9 | 367085 | ENSRNOG00000025624 | ENSRNOT00000035989  ENSRNOT00000059078 | Arhgap20 | Rho GTPase-activating protein 20 (Rho-type GTPase-activating protein 20) (RA and RhoGAP domain-containing protein) (RARhoGAP). [Source:UniProtKB/Swiss-Prot;Acc:Q6REY9] | 1 |
| 1382096\_at |  | 290214 | ENSRNOG00000016828 | ENSRNOT00000022664  ENSRNOT00000022674 | NM\_001106034.1 | chemokine-like factor super family 5 (predicted) (Cklfsf5\_predicted), mRNA [Source:RefSeq\_dna;Acc:NM\_001106034] | 1 |
| 1370180\_at | Q99MY2 | 94267 | ENSRNOG00000009094 | ENSRNOT00000012363 | Nudt4 | Diphosphoinositol polyphosphate phosphohydrolase 2 (EC 3.6.1.52) (DIPP-2) (rDIPP2) (Diadenosine 5',5'''-P1,P6-hexaphosphate hydrolase 2) (EC 3.6.1.-) (Nucleoside diphosphate-linked moiety X motif 4) (Nudix motif 4). [Source:UniProtKB/Swiss-Prot;Acc:Q99MY2] | 1 |
| 1386648\_at |  |  | ENSRNOG00000011968 | ENSRNOT00000016033 | Dnajc6 | DnaJ (Hsp40) homolog, subfamily C, member 6 Gene [Source:MGI (curated);Acc:Dnajc6-001] | 1 |
| 1371973\_at | Q641X8 | 299872 | ENSRNOG00000027690  ENSRNOG00000037383 | ENSRNOT00000038340  ENSRNOT00000056568 | Eif3s6 | Eukaryotic translation initiation factor 3 subunit E (Eukaryotic translation initiation factor 3 subunit 6) (eIF-3 p48) (eIF3e). [Source:UniProtKB/Swiss-Prot;Acc:Q641X8] | 1 |
| 1369770\_at | P28646 | 25033 | ENSRNOG00000022714 | ENSRNOT00000012609 | Sstr1 | Somatostatin receptor type 1 (SS1R) (SRIF-2). [Source:UniProtKB/Swiss-Prot;Acc:P28646] | 1 |
| 1368182\_at | P33124 | 117243 | ENSRNOG00000026745 | ENSRNOT00000048924  ENSRNOT00000030715  ENSRNOT00000030760  ENSRNOT00000058817 | Acsl6 | Long-chain-fatty-acid--CoA ligase 6 (EC 6.2.1.3) (Long-chain acyl-CoA synthetase 6) (LACS 6) (Long-chain-fatty-acid--CoA ligase, brain isozyme). [Source:UniProtKB/Swiss-Prot;Acc:P33124] | 1 |
| 1373375\_at |  |  | ENSRNOG00000012206 | ENSRNOT00000017485 | Rab6ip1 | DENN/MADD domain containing 5A Gene [Source:MGI (curated);Acc:Rab6ip1-001] | 1 |
| 1377194\_a\_at | Q4V897 | 308820 | ENSRNOG00000009462 | ENSRNOT00000012781 | RGD1308637 | Coiled-coil domain-containing protein 90B, mitochondrial precursor. [Source:UniProtKB/Swiss-Prot;Acc:Q4V897] | 1 |
| 1373983\_at |  |  | ENSRNOG00000001238 | ENSRNOT00000057788 | LOC360807 | Arginine/serine-rich coiled-coil protein 2. [Source:UniProtKB/Swiss-Prot;Acc:Q5PQR4] | 1 |
| 1386934\_at | P28570 | 50690 | ENSRNOG00000032701 | ENSRNOT00000025702 | Slc6a8 | Sodium- and chloride-dependent creatine transporter 1 (CT1) (Creatine transporter 1) (CHOT1) (Solute carrier family 6 member 8). [Source:UniProtKB/Swiss-Prot;Acc:P28570] | 1 |
| 1371921\_at |  |  | ENSRNOG00000005796 | ENSRNOT00000008041 | Catna1 | catenin (cadherin-associated protein), alpha 1, 102kDa [Source:RefSeq\_peptide;Acc:NP\_001007146] | 1 |
| 1371853\_at | P0C2B9 | 299743 | ENSRNOG00000025964 | ENSRNOT00000011332 | RT32\_RAT | Mitochondrial 28S ribosomal protein S32, mitochondrial precursor (S32mt) (MRP-S32). [Source:UniProtKB/Swiss-Prot;Acc:P0C2B9] | 1 |
| 1395020\_at |  | 314262 | ENSRNOG00000010650 | ENSRNOT00000014257 | NP\_001101506.1 | pleckstrin homology domain containing, family H (with MyTH4 domain) member 1 [Source:RefSeq\_peptide;Acc:NP\_001101506] | 1 |
| 1382399\_at | Q4FZU3 | 303346 | ENSRNOG00000022502 | ENSRNOT00000030749 | RGD1309863 | Coiled-coil domain-containing protein 55. [Source:UniProtKB/Swiss-Prot;Acc:Q4FZU3] | 1 |
| 1397704\_at |  | 691979  683635 | ENSRNOG00000015921 | ENSRNOT00000021320 | LOC691979 |  | 1 |
| 1394082\_at |  | 311078 | ENSRNOG00000004911 | ENSRNOT00000006605 | LOC311078 | Similar to T-Brain-1. [Source:UniProtKB/TrEMBL;Acc:Q4V8E2] | 1 |
| 1372286\_at |  | 302313 | ENSRNOG00000003786 | ENSRNOT00000005165 | Tspan6 | Tspan6 protein (Fragment). [Source:UniProtKB/TrEMBL;Acc:Q5RJZ3] | 1 |
| 1387286\_at | P23385 | 24414 | ENSRNOG00000014290 | ENSRNOT00000019319 | Grm1 | Metabotropic glutamate receptor 1 precursor (mGluR1). [Source:UniProtKB/Swiss-Prot;Acc:P23385] | 1 |
| 1371335\_at | Q6P7A2 | 300677  315608 | ENSRNOG00000028884  ENSRNOG00000026833 | ENSRNOT00000050878  ENSRNOT00000021060  ENSRNOT00000021321 | MGC72942  Ube4a | ATP synthase, H+ transporting, mitochondrial F0 complex, subunit G [Source:RefSeq\_peptide;Acc:NP\_997681]  Ubiquitin conjugation factor E4 A. [Source:UniProtKB/Swiss-Prot;Acc:Q6P7A2] | 1 |
| 1390699\_at |  | 309307 | ENSRNOG00000026942 | ENSRNOT00000039428 | RGD1311595 | RGD1311595 protein (Fragment). [Source:UniProtKB/TrEMBL;Acc:Q3KRD9] | 1 |
| 1398813\_at | Q99MI7 | 117553 | ENSRNOG00000006221 | ENSRNOT00000008893 | Ube1c | NEDD8-activating enzyme E1 catalytic subunit (EC 6.3.2.-) (Ubiquitin- like modifier-activating enzyme 3) (Ubiquitin-activating enzyme 3) (NEDD8-activating enzyme E1C) (Ubiquitin-activating enzyme E1C). [Source:UniProtKB/Swiss-Prot;Acc:Q99MI7] | 1 |
| 1371840\_at | P48303 | 29733 | ENSRNOG00000013683 | ENSRNOT00000018318 | Edg1 | Sphingosine 1-phosphate receptor Edg-1 (Sphingosine 1-phosphate receptor 1) (S1P1). [Source:UniProtKB/Swiss-Prot;Acc:P48303] | 1 |
| 1394710\_at | Q4KMA0 | 316051 | ENSRNOG00000010096 | ENSRNOT00000013450 | Azi2 | 5-azacytidine-induced protein 2. [Source:UniProtKB/Swiss-Prot;Acc:Q4KMA0] | 1 |
| 1367550\_a\_at |  | 362545 | ENSRNOG00000007527 | ENSRNOT00000009897 | NP\_001102140.1 | TM2 domain containing 1 [Source:RefSeq\_peptide;Acc:NP\_001102140] | 1 |
| 1399159\_a\_at | P63025 | 29528 | ENSRNOG00000030055 | ENSRNOT00000051554 | Vamp3 | Vesicle-associated membrane protein 3 (VAMP-3) (Synaptobrevin-3) (Cellubrevin) (CEB). [Source:UniProtKB/Swiss-Prot;Acc:P63025] | 1 |
| 1368782\_at | P30680 | 54305 | ENSRNOG00000002793 | ENSRNOT00000003735 | Sstr2 | Somatostatin receptor type 2 (SS2R) (SRIF-1). [Source:UniProtKB/Swiss-Prot;Acc:P30680] | 1 |
| 1373547\_at | Q5M888 | 307008 | ENSRNOG00000015523 | ENSRNOT00000020794 | RGD1308147 | UPF0415 protein C7orf25 homolog. [Source:UniProtKB/Swiss-Prot;Acc:Q5M888] | 1 |
| 1389482\_at | Q5PPL1 | 312437 | ENSRNOG00000007124 | ENSRNOT00000009327 | RGD1306495 | Lysine-rich coiled-coil protein 1. [Source:UniProtKB/Swiss-Prot;Acc:Q5PPL1] | 1 |
| 1387865\_at | P70583 | 497778 | ENSRNOG00000017412  ENSRNOG00000007221 | ENSRNOT00000023387  ENSRNOT00000009549 | Dut | Deoxyuridine 5'-triphosphate nucleotidohydrolase (EC 3.6.1.23) (dUTPase) (dUTP pyrophosphatase) (PPAR-interacting protein 4) (PIP4). [Source:UniProtKB/Swiss-Prot;Acc:P70583] | 1 |
| 1392640\_at | Q32Q86 | 299691 | ENSRNOG00000006622 | ENSRNOT00000009124  ENSRNOT00000037233 | Cry1 | Cryptochrome-1. [Source:UniProtKB/Swiss-Prot;Acc:Q32Q86] | 1 |
| 1384089\_at |  | 360797 | ENSRNOG00000000895 | ENSRNOT00000001196 | NP\_001101803.1 | RAB guanine nucleotide exchange factor (GEF) 1 [Source:RefSeq\_peptide;Acc:NP\_001101803] | 1 |
| 1389364\_at |  | 361089 | ENSRNOG00000024022 | ENSRNOT00000039423 | NP\_001101860.1 | Nedd4 family interacting protein 2 [Source:RefSeq\_peptide;Acc:NP\_001101860] | 1 |
| 1391443\_at |  | 498489 | ENSRNOG00000014192 | ENSRNOT00000020074 | NP\_001102569.1 | similar to chromosome 14 open reading frame 35 (predicted) (RGD1559923\_predicted), mRNA [Source:RefSeq\_dna;Acc:NM\_001109099] | 1 |
| 1373049\_at |  | 501195 | ENSRNOG00000032398 | ENSRNOT00000045749 | NP\_001102556.1 | similar to D1Ertd622e protein (predicted) (RGD1562136\_predicted), mRNA [Source:RefSeq\_dna;Acc:NM\_001109086] | 1 |
| 1379271\_at |  | 500616 | ENSRNOG00000028504 | ENSRNOT00000020337 | NP\_001102744.1 | similar to Suppressor of cytokine signaling 5 (predicted) (RGD1564914\_predicted), mRNA [Source:RefSeq\_dna;Acc:NM\_001109274] | 1 |
| 1399050\_at |  | 289276 | ENSRNOG00000004481 | ENSRNOT00000005961 | NP\_001099445.1 | adenylosuccinate synthetase, non muscle [Source:RefSeq\_peptide;Acc:NP\_001099445] | 1 |
| 1367686\_at | Q9R2C1 | 80881 | ENSRNOG00000011763 | ENSRNOT00000017868 | RAMP4 | Stress-associated endoplasmic reticulum protein 1 (Ribosome-attached membrane protein 4). [Source:UniProtKB/Swiss-Prot;Acc:Q9R2C1] | 1 |
| 1398866\_at |  | 245903 | ENSRNOG00000019885 | ENSRNOT00000026952 | Magi3 | membrane associated guanylate kinase, WW and PDZ domain containing 3 [Source:RefSeq\_peptide;Acc:NP\_620784] | 1 |
| 1393128\_at | Q566R3 | 295228 | ENSRNOG00000018969 | ENSRNOT00000056923 | RGD1311086 | G patch domain-containing protein 4. [Source:UniProtKB/Swiss-Prot;Acc:Q566R3] | 1 |
| 1384377\_at |  | 364995 | ENSRNOG00000019817 | ENSRNOT00000026817 | NP\_001102368.1 | DEAD (Asp-Glu-Ala-Asp) box polypeptide 28 [Source:RefSeq\_peptide;Acc:NP\_001102368] | 1 |
| 1389028\_at | Q9JLI4 | 116464 | ENSRNOG00000018288 | ENSRNOT00000024714 | Ncoa6 | Nuclear receptor coactivator 6 (Amplified in breast cancer protein 3) (Cancer-amplified transcriptional coactivator ASC-2) (Activating signal cointegrator 2) (ASC-2) (Peroxisome proliferator-activated receptor-interacting protein) (PPAR-interacting protei [Source:UniProtKB/Swiss-Prot;Acc:Q9JLI4] | 1 |
| 1394363\_at |  | 361293 | ENSRNOG00000023376 | ENSRNOT00000039377 | NP\_001101893.1 | RIO kinase 3 [Source:RefSeq\_peptide;Acc:NP\_001101893] | 1 |
| 1372356\_at |  | 408223 | ENSRNOG00000027012 | ENSRNOT00000029615 | Usp54 | ubiquitin specific protease 54 [Source:RefSeq\_peptide;Acc:NP\_001008863] | 1 |
| 1382137\_at |  | 291793 | ENSRNOG00000029370 | ENSRNOT00000041878 | NP\_001099632.1 | abhydrolase domain containing 3 [Source:RefSeq\_peptide;Acc:NP\_001099632] | 1 |
| 1398889\_at |  | 679772  689776  689718  192147  691765  690664  501353 | ENSRNOG00000016300 | ENSRNOT00000023071 | Grinl1a | glutamate receptor, ionotropic, N-methyl D-aspartate-like 1A [Source:RefSeq\_peptide;Acc:NP\_899652] | 1 |
| 1393274\_at | Q5XI79 | 298748 | ENSRNOG00000005279 | ENSRNOT00000007000 | RGD1311578 | UPF0511 protein C2orf56 homolog, mitochondrial precursor. [Source:UniProtKB/Swiss-Prot;Acc:Q5XI79] | 1 |
| 1373284\_at | A4V8B4 | 299116 | ENSRNOG00000005264 | ENSRNOT00000007314 | Sav1\_predicted | Protein salvador homolog 1 (45 kDa WW domain protein) (rWW45). [Source:UniProtKB/Swiss-Prot;Acc:A4V8B4] | 1 |
| 1398385\_at |  |  | ENSRNOG00000007701 | ENSRNOT00000010675 | Thoc7 | THO complex 7 homolog (Drosophila) Gene [Source:MGI Symbol;Acc:MGI:1913481] | 1 |
| 1383876\_at |  | 313365 | ENSRNOG00000008075 | ENSRNOT00000011336 | Ift74 | coiled-coil domain containing 2 [Source:RefSeq\_peptide;Acc:NP\_001007002] | 1 |
| 1396142\_at |  | 301371 | ENSRNOG00000016201 | ENSRNOT00000021899  ENSRNOT00000041108 | Mrps9 | Mrps9 protein (Fragment). [Source:UniProtKB/TrEMBL;Acc:Q5I0K4] | 1 |
| 1377145\_at |  | 294614 | ENSRNOG00000023651 | ENSRNOT00000039652 | RGD1562101\_predicted |  | 1 |
| 1386344\_at | P58366 | 114506 | ENSRNOG00000010960 | ENSRNOT00000015561 | Ank | Progressive ankylosis protein homolog (ANK). [Source:UniProtKB/Swiss-Prot;Acc:P58366] | 1 |
| 1383477\_at |  |  | ENSRNOG00000003545 | ENSRNOT00000060045 | Uchl5 | ubiquitin carboxyl-terminal hydrolase L5 [Source:RefSeq\_peptide;Acc:NP\_001012149] | 1 |
| 1395387\_at | P83829 | 373066 | ENSRNOG00000001294 | ENSRNOT00000001742 | Cdv1 | Intraflagellar transport 81 (Carnitine deficiency-associated protein expressed in ventricle 1) (CDV-1 protein). [Source:UniProtKB/Swiss-Prot;Acc:P83829] | 1 |
| 1383147\_at |  |  | ENSRNOG00000017707 | ENSRNOT00000050179 | Abi2 | abl-interactor 2 [Source:RefSeq\_peptide;Acc:NP\_775166] | 1 |
| 1369939\_at | P62898 | 25309  679794  682626  683306  689459 | ENSRNOG00000010452 | ENSRNOT00000014058 | Cycs | Cytochrome c, somatic. [Source:UniProtKB/Swiss-Prot;Acc:P62898] | 1 |
| 1389386\_at |  | 363169 | ENSRNOG00000004085 | ENSRNOT00000005440 | NP\_001104308.1 |  | 1 |
| 1389090\_at | Q8CG07 | 282835 | ENSRNOG00000017040 | ENSRNOT00000023332 | Wrnip1 | ATPase WRNIP1 (Werner helicase-interacting protein 1). [Source:UniProtKB/Swiss-Prot;Acc:Q8CG07] | 1 |
| 1388789\_at |  | 362857 | ENSRNOG00000006987 | ENSRNOT00000009154 | NP\_001102209.1 |  | 1 |
| 1370282\_at | Q62908 | 29317 | ENSRNOG00000003772 | ENSRNOT00000005041 | Csrp2 | Cysteine and glycine-rich protein 2 (Cysteine-rich protein 2) (CRP2) (Smooth muscle cell LIM protein) (SmLIM). [Source:UniProtKB/Swiss-Prot;Acc:Q62908] | 1 |
| 1373445\_at |  | 361221 | ENSRNOG00000014818 | ENSRNOT00000038476 | NP\_001101878.1 | nucleolar protein 8 [Source:RefSeq\_peptide;Acc:NP\_001101878] | 1 |
| 1390789\_at |  | 315973 | ENSRNOG00000010940 | ENSRNOT00000038383 | NP\_001101651.1 | acyl-Coenzyme A dehydrogenase family, member 11 [Source:RefSeq\_peptide;Acc:NP\_001101651] | 1 |
| 1384252\_at |  | 301381 | ENSRNOG00000011658 | ENSRNOT00000015600 | NP\_001100379.1 |  | 1 |
| 1373904\_at |  | 300839 | ENSRNOG00000010642 | ENSRNOT00000014301 | NP\_001100309.1 | LysM, putative peptidoglycan-binding, domain containing 2 [Source:RefSeq\_peptide;Acc:NP\_001100309] | 1 |
| 1372443\_at |  | 292462 | ENSRNOG00000014303 | ENSRNOT00000062020 | NP\_001099687.1 | low density lipoprotein receptor-related protein 11 [Source:RefSeq\_peptide;Acc:NP\_001099687] | 1 |
| 1368563\_at | Q9R1T5 | 79251 | ENSRNOG00000019659 | ENSRNOT00000049311  ENSRNOT00000026743 | Aspa | Aspartoacylase (EC 3.5.1.15) (Aminoacylase-2) (ACY-2). [Source:UniProtKB/Swiss-Prot;Acc:Q9R1T5] | 1 |
| 1388136\_at | Q9WV97 | 171139 | ENSRNOG00000008222 | ENSRNOT00000010871 | Timm9 | Mitochondrial import inner membrane translocase subunit Tim9. [Source:UniProtKB/Swiss-Prot;Acc:Q9WV97] | 1 |
| 1368083\_at | Q9R1A0 | 84389 | ENSRNOG00000031656 | ENSRNOT00000049423 | Ccnh | Cyclin-H. [Source:UniProtKB/Swiss-Prot;Acc:Q9R1A0] | 1 |
| 1384877\_at | Q8CHM1 | 286758 | ENSRNOG00000013358 | ENSRNOT00000018091 | Aqp11 | Aquaporin-11 (AQP-11). [Source:UniProtKB/Swiss-Prot;Acc:Q8CHM1] | 1 |
| 1378854\_at |  |  | ENSRNOG00000017826 | ENSRNOT00000024041 | Mtrr | 5-methyltetrahydrofolate-homocysteine methyltransferase reductase [Source:RefSeq\_peptide;Acc:NP\_001034092] | 1 |
| 1372801\_at |  | 361323 | ENSRNOG00000003958 | ENSRNOT00000005260 | Commd10 | COMM domain containing 10 [Source:RefSeq\_peptide;Acc:NP\_001004276] | 1 |
| 1374874\_at |  |  | ENSRNOG00000005106 | ENSRNOT00000006968 | Cc2d2a | coiled-coil and C2 domain containing 2A Gene [Source:MGI (curated);Acc:Cc2d2a-001] | 1 |
| 1391475\_at |  | 313842 | ENSRNOG00000006929 | ENSRNOT00000009175 | Hnrpll\_predicted |  | 1 |
| 1398893\_at | Q5U2S1 | 291609 | ENSRNOG00000013562 | ENSRNOT00000018499 | Ndfip1 | NEDD4 family-interacting protein 1. [Source:UniProtKB/Swiss-Prot;Acc:Q5U2S1] | 1 |
| 1368038\_at | Q9WVJ4 | 64531 | ENSRNOG00000006399 | ENSRNOT00000009122 | Synj2bp | Synaptojanin-2-binding protein (Mitochondrial outer membrane protein 25) (NPW16). [Source:UniProtKB/Swiss-Prot;Acc:Q9WVJ4] | 1 |
| 1381042\_at |  | 361389 | ENSRNOG00000018296 | ENSRNOT00000024689 | NP\_001101915.1 | anaphase promoting complex subunit 10 [Source:RefSeq\_peptide;Acc:NP\_001101915] | 1 |
| 1389476\_at |  | 294978 | ENSRNOG00000026660 | ENSRNOT00000023150 | RGD1307100 |  | 1 |
| 1393722\_at |  | 302288 | ENSRNOG00000003578 | ENSRNOT00000004784 | NP\_001100402.1 | fem-1 homolog c [Source:RefSeq\_peptide;Acc:NP\_001100402] | 1 |
| 1385838\_a\_at |  | 362545 | ENSRNOG00000007527 | ENSRNOT00000009897 | NP\_001102140.1 | TM2 domain containing 1 [Source:RefSeq\_peptide;Acc:NP\_001102140] | 1 |
| 1368029\_at | P10824  P08753 | 25643  25686 | ENSRNOG00000019465 | ENSRNOT00000026710 | Gnai3 | Guanine nucleotide-binding protein G(k) subunit alpha (G(i) alpha-3). [Source:UniProtKB/Swiss-Prot;Acc:P08753] | 1 |
| 1387105\_at | Q9ERU2 | 360389 | ENSRNOG00000013379 | ENSRNOT00000018050 | Zfp422 | Zinc finger protein 22 (Krueppel-type zinc finger protein Krox-25). [Source:UniProtKB/Swiss-Prot;Acc:Q9ERU2] | 1 |
| 1387358\_at | P61212 | 64187 | ENSRNOG00000005763 | ENSRNOT00000007623 | Arl1 | ADP-ribosylation factor-like protein 1. [Source:UniProtKB/Swiss-Prot;Acc:P61212] | 1 |
| 1392654\_at |  | 361473 | ENSRNOG00000018773 | ENSRNOT00000025398 | LOC361473 | similar to mitochondrial translational release factor 1-like (LOC361473), mRNA [Source:RefSeq\_dna;Acc:NM\_001025723] | 1 |
| 1371843\_at | Q5XID0 | 361315 | ENSRNOG00000014564 | ENSRNOT00000020385 | Yipf5 | Protein YIPF5 (YIP1 family member 5) (YPT-interacting protein 1 A). [Source:UniProtKB/Swiss-Prot;Acc:Q5XID0] | 1 |
| 1372765\_a\_at |  | 291075  291076 | ENSRNOG00000016369  ENSRNOG00000029549 | ENSRNOT00000022022  ENSRNOT00000060166  ENSRNOT00000048923 | Peci  RGD1310224 | peroxisomal delta3, delta2-enoyl-Coenzyme A isomerase [Source:RefSeq\_peptide;Acc:NP\_001006967]  similar to RIKEN cDNA 1810022C23 (RGD1310224), mRNA [Source:RefSeq\_dna;Acc:NM\_001009275] | 1 |
| 1367654\_at |  | 83720 | ENSRNOG00000030954 | ENSRNOT00000046467  ENSRNOT00000046502 | Fath | fat tumor suppressor homolog [Source:RefSeq\_peptide;Acc:NP\_114007] | 1 |
| 1387040\_at | Q64349 | 25263 | ENSRNOG00000015445 | ENSRNOT00000020870 | Mal | Myelin and lymphocyte protein (T-lymphocyte maturation-associated protein) (17 kDa myelin vesicular protein) (MVP17) (NS 3). [Source:UniProtKB/Swiss-Prot;Acc:Q64349] | 1 |
| 1371720\_at |  | 687253  680747 | ENSRNOG00000018647 | ENSRNOT00000025213 | LOC687253 |  | 1 |
| 1389154\_at |  | 305348 | ENSRNOG00000002759 | ENSRNOT00000003779 | Lias | lipoic acid synthetase [Source:RefSeq\_peptide;Acc:NP\_001012037] | 1 |
| 1383731\_at | Q6AYA5 | 312132 | ENSRNOG00000006206 | ENSRNOT00000008677 | Tmem106b | Transmembrane protein 106B. [Source:UniProtKB/Swiss-Prot;Acc:Q6AYA5] | 1 |
| 1392930\_at |  | 294948 | ENSRNOG00000013253 | ENSRNOT00000018548 | NP\_001099895.1 | armadillo repeat containing 1 [Source:RefSeq\_peptide;Acc:NP\_001099895] | 1 |
| 1368658\_at | P20294 | 25707 | ENSRNOG00000012460 | ENSRNOT00000016690 | Cntf | Ciliary neurotrophic factor (CNTF). [Source:UniProtKB/Swiss-Prot;Acc:P20294] | 1 |
| 1372714\_at | Q5PQM0 | 312135 | ENSRNOG00000006690 | ENSRNOT00000009450 | RGD1307778 | Transmembrane protein 168. [Source:UniProtKB/Swiss-Prot;Acc:Q5PQM0] | 1 |
| 1369968\_at | P63090 | 24924 | ENSRNOG00000011946 | ENSRNOT00000016088 | Ptn | Pleiotrophin precursor (PTN) (Heparin-binding growth-associated molecule) (HB-GAM) (Heparin-binding growth factor 8) (HBGF-8) (Osteoblast-specific factor 1) (OSF-1) (Heparin-binding neutrophic factor) (HBNF) (Heparin-binding brain mitogen) (HBBM). [Source:UniProtKB/Swiss-Prot;Acc:P63090] | 1 |
| 1389325\_at | Q6NX65 | 494345 | ENSRNOG00000010147 | ENSRNOT00000013585 | MGC72992 | Programmed cell death protein 10. [Source:UniProtKB/Swiss-Prot;Acc:Q6NX65] | 1 |
| 1377869\_at | Q9ET55 | 171555  310395 | ENSRNOG00000010799 | ENSRNOT00000014348 | Ccrn4l | Nocturnin (CCR4 protein homolog) (Fragment). [Source:UniProtKB/Swiss-Prot;Acc:Q9ET55] | 1 |
| 1370193\_at | Q78EG7 | 29463 | ENSRNOG00000011771 | ENSRNOT00000016237 | Ptp4a1 | Protein tyrosine phosphatase type IVA protein 1 (EC 3.1.3.48) (Protein-tyrosine phosphatase 4a1) (Protein-tyrosine phosphatase of regenerating liver 1) (PRL-1). [Source:UniProtKB/Swiss-Prot;Acc:Q78EG7] | 1 |
| 1370230\_at | P21571 | 500560  94271 | ENSRNOG00000001551 | ENSRNOT00000002116 | Atp5j | ATP synthase-coupling factor 6, mitochondrial precursor (ATPase subunit F6). [Source:UniProtKB/Swiss-Prot;Acc:P21571] | 1 |
| 1388471\_at | Q568Z0 | 314683 | ENSRNOG00000007587 | ENSRNOT00000010024 | Tcp11l2 | T-complex protein 11-like 2. [Source:UniProtKB/Swiss-Prot;Acc:Q568Z0] | 1 |
| 1393027\_at | Q6AYT5 | 292267 | ENSRNOG00000019489 | ENSRNOT00000026367 | RGD1305235 | UPF0364 protein C6orf211 homolog. [Source:UniProtKB/Swiss-Prot;Acc:Q6AYT5] | 1 |
| 1390343\_at | P39947 | 114839 | ENSRNOG00000007719 | ENSRNOT00000010655 | Ccnc | Cyclin-C. [Source:UniProtKB/Swiss-Prot;Acc:P39947] | 1 |
| 1371389\_at | Q5U2R6 | 306766 | ENSRNOG00000017133 | ENSRNOT00000060777  ENSRNOT00000023026 | LOC306766 | UPF0498 protein KIAA1191 homolog (liver regeneration-related protein LRRG011). [Source:UniProtKB/Swiss-Prot;Acc:Q5U2R6] | 1 |
| 1389088\_at | Q9JKL8 | 64622 | ENSRNOG00000010975 | ENSRNOT00000014595 | Adnp | Activity-dependent neuroprotector homeobox protein (Activity-dependent neuroprotective protein). [Source:UniProtKB/Swiss-Prot;Acc:Q9JKL8] | 1 |
| 1375521\_at | Q6I7R5 | 367909 | ENSRNOG00000028585 | ENSRNOT00000017138 | TCAL8\_RAT | Transcription elongation factor A protein-like 8 (TCEA-like protein 8) (Transcription elongation factor S-II protein-like 8) (Up-regulated in nephrectomized rat kidney #1). [Source:UniProtKB/Swiss-Prot;Acc:Q6I7R5] | 1 |
| 1399045\_at | Q10473 | 79214 | ENSRNOG00000016207 | ENSRNOT00000022117 | Galnt1 | Polypeptide N-acetylgalactosaminyltransferase 1 (EC 2.4.1.41) (Protein-UDP acetylgalactosaminyltransferase 1) (UDP- GalNAc:polypeptide N-acetylgalactosaminyltransferase 1) (Polypeptide GalNAc transferase 1) (GalNAc-T1) (pp-GaNTase 1) [Contains: Polypeptid [Source:UniProtKB/Swiss-Prot;Acc:Q10473] | 1 |
| 1386981\_at | P53987 | 25027 | ENSRNOG00000019996 | ENSRNOT00000027234 | Slc16a1 | Monocarboxylate transporter 1 (MCT 1) (Solute carrier family 16 member 1). [Source:UniProtKB/Swiss-Prot;Acc:P53987] | 1 |
| 1386793\_at |  | 499094 | ENSRNOG00000019406 | ENSRNOT00000033531  ENSRNOT00000026252 | LOC499094 | similar to zinc finger protein 61 [Source:RefSeq\_peptide;Acc:NP\_001017512] | 1 |
| 1372177\_at |  | 294753 | ENSRNOG00000011797 | ENSRNOT00000015781 | Mocs2 | molybdenum cofactor synthesis 2 [Source:RefSeq\_peptide;Acc:NP\_001007634] | 1 |
| 1370296\_at | P11915 | 25541 | ENSRNOG00000011413 | ENSRNOT00000015420 | Scp2 | Non-specific lipid-transfer protein (EC 2.3.1.176) (Propanoyl-CoA C- acyltransferase) (NSL-TP) (Sterol carrier protein 2) (SCP-2) (Sterol carrier protein X) (SCP-X) (SCP-chi) (SCPX). [Source:UniProtKB/Swiss-Prot;Acc:P11915] | 1 |
| 1388617\_at |  | 361239 | ENSRNOG00000017577 | ENSRNOT00000023914 | Bphl | biphenyl hydrolase-like (serine hydrolase, breast epithelial mucin-associated antigen) [Source:RefSeq\_peptide;Acc:NP\_001032283] | 1 |
| 1375538\_at | P53676 | 171126 | ENSRNOG00000011461 | ENSRNOT00000016387 | Ap3m1 | AP-3 complex subunit mu-1 (Adapter-related protein complex 3 mu-1 subunit) (Mu3A-adaptin) (Mu-adaptin 3A) (AP-3 adapter complex mu3A subunit) (Clathrin coat assembly protein AP47 homolog 1) (Clathrin coat-associated protein AP47 homolog 1) (Golgi adaptor [Source:UniProtKB/Swiss-Prot;Acc:P53676] | 1 |
| 1370328\_at |  | 171548 | ENSRNOG00000016343 | ENSRNOT00000022301 | Dkk3 | dickkopf homolog 3 [Source:RefSeq\_peptide;Acc:NP\_612528] | 1 |
| 1393018\_at |  |  | ENSRNOG00000039096 | ENSRNOT00000059794 | AC102287.16 | Putative uncharacterized protein [Source:UniProtKB/TrEMBL;Acc:Q3V438] | 1 |
| 1382189\_at | P34900 | 25615 | ENSRNOG00000004936 | ENSRNOT00000007255 | Sdc2 | Syndecan-2 precursor (SYND2) (Fibroglycan) (Heparan sulfate proteoglycan core protein) (HSPG). [Source:UniProtKB/Swiss-Prot;Acc:P34900] | 1 |
| 1373411\_at |  | 301068 | ENSRNOG00000018848 | ENSRNOT00000025615 | NP\_001100337.1 | eukaryotic translation initiation factor 1B [Source:RefSeq\_peptide;Acc:NP\_001100337] | 1 |
| 1398989\_at |  | 304092 | ENSRNOG00000002021 | ENSRNOT00000002769 | Son | Son protein (Fragment). [Source:UniProtKB/TrEMBL;Acc:Q6PDU3] | 1 |
| 1382264\_at |  |  | ENSRNOG00000005330 | ENSRNOT00000049944 | Crebbp | CREB binding protein [Source:RefSeq\_peptide;Acc:NP\_596872] | 1 |
| 1388963\_at |  | 304900 | ENSRNOG00000005667 | ENSRNOT00000007660 | Astn1 |  | 1 |
| 1388642\_at |  | 300514 | ENSRNOG00000030391 | ENSRNOT00000046590 | Ei24 | etoposide induced 2.4 mRNA [Source:RefSeq\_peptide;Acc:NP\_001020831] | 1 |
| 1390821\_at |  | 393088  393089  393090  116742  393091  393092 | ENSRNOG00000020119 | ENSRNOT00000027393  ENSRNOT00000027383  ENSRNOT00000027377  ENSRNOT00000027372  ENSRNOT00000027349  ENSRNOT00000048626  ENSRNOT00000027343  ENSRNOT00000027340 | Pcdhac2 | protocadherin alpha 13 [Source:RefSeq\_peptide;Acc:NP\_446386] | 1 |
| 1388822\_at |  | 364981 | ENSRNOG00000003853 | ENSRNOT00000005124 | Scoc | short coiled-coil protein [Source:RefSeq\_peptide;Acc:NP\_001013253] | 1 |
| 1399008\_at |  |  | ENSRNOG00000002940 | ENSRNOT00000035592 | Q99MD2\_RAT | Serologically defined breast cancer antigen NY-BR-16-like protein (Fragment). [Source:UniProtKB/TrEMBL;Acc:Q99MD2] | 1 |
| 1367976\_at | Q64560 | 81815 | ENSRNOG00000011194 | ENSRNOT00000015161  ENSRNOT00000015393 | Tpp2 | Tripeptidyl-peptidase 2 (EC 3.4.14.10) (Tripeptidyl-peptidase II) (TPP-II) (Tripeptidyl aminopeptidase). [Source:UniProtKB/Swiss-Prot;Acc:Q64560] | 1 |
| 1370920\_at |  | 296753 | ENSRNOG00000010601 | ENSRNOT00000014307 | NP\_001100045.1 | serine/arginine-rich protein specific kinase 2 [Source:RefSeq\_peptide;Acc:NP\_001100045] | 1 |
| 1377713\_at |  | 314374 | ENSRNOG00000004709 | ENSRNOT00000006604 | NP\_001101517.1 | checkpoint suppressor 1 [Source:RefSeq\_peptide;Acc:NP\_001101517] | 1 |
| 1393082\_at | Q8R4R9 | 171010 | ENSRNOG00000016368 | ENSRNOT00000022046 | Ppp1r14c | Protein phosphatase 1 regulatory subunit 14C (PKC-potentiated PP1 inhibitory protein) (Kinase-enhanced PP1 inhibitor). [Source:UniProtKB/Swiss-Prot;Acc:Q8R4R9] | 1 |
| 1382689\_at | Q5FVN2 | 361626 | ENSRNOG00000010752 | ENSRNOT00000016224 | RGD1310870 | Transmembrane protein 41B. [Source:UniProtKB/Swiss-Prot;Acc:Q5FVN2] | 1 |
| 1370286\_at | Q9JHE5 | 29642 | ENSRNOG00000006305 | ENSRNOT00000039002 | Slc38a2 | Sodium-coupled neutral amino acid transporter 2 (Amino acid transporter A2) (System A transporter 1) (System A amino acid transporter 2) (System N amino acid transporter 2) (Solute carrier family 38 member 2). [Source:UniProtKB/Swiss-Prot;Acc:Q9JHE5] | 1 |
| 1382924\_at |  | 294088 | ENSRNOG00000018944 | ENSRNOT00000025736 | NP\_001099843.1 | pantothenate kinase 1 [Source:RefSeq\_peptide;Acc:NP\_001099843] | 1 |
| 1382283\_at | Q6IN36 | 117538 | ENSRNOG00000018406 | ENSRNOT00000024922 | Waspip | WAS/WASL-interacting protein family member 1 (Wiskott-Aldrich syndrome protein-interacting protein) (WASP-interacting protein). [Source:UniProtKB/Swiss-Prot;Acc:Q6IN36] | 1 |
| 1370834\_at | Q9ESG5 | 84406 | ENSRNOG00000010598 | ENSRNOT00000014078 | Hs3st1 | Heparan sulfate glucosamine 3-O-sulfotransferase 1 precursor (EC 2.8.2.23) (Heparan sulfate D-glucosaminyl 3-O-sulfotransferase 1) (Heparan sulfate 3-O-sulfotransferase 1). [Source:UniProtKB/Swiss-Prot;Acc:Q9ESG5] | 1 |
| 1388989\_at |  | 686327  688712  361923  688103  680575  303576 | ENSRNOG00000033819  ENSRNOG00000011817  ENSRNOG00000029173  ENSRNOG00000036783 | ENSRNOT00000045060  ENSRNOT00000015756  ENSRNOT00000040366  ENSRNOT00000055169 | LOC688712 | ribosomal protein L22 like 1 [Source:RefSeq\_peptide;Acc:NP\_001102018] | 1 |
| 1367895\_at | P61980 | 117282 | ENSRNOG00000019113  ENSRNOG00000013579  ENSRNOG00000029014 | ENSRNOT00000025980  ENSRNOT00000025916  ENSRNOT00000025961  ENSRNOT00000018164  ENSRNOT00000045659  ENSRNOT00000056989  ENSRNOT00000056988 | Hnrpk  AC116389.12 | Heterogeneous nuclear ribonucleoprotein K (hnRNP K) (dC stretch- binding protein) (CSBP). [Source:UniProtKB/Swiss-Prot;Acc:P61980] | 1 |
| 1394077\_at | Q6SA80 | 295588 | ENSRNOG00000004624 | ENSRNOT00000006111 | Rnd3 | Rho-related GTP-binding protein RhoE precursor (Rho family GTPase 3) (Rnd3). [Source:UniProtKB/Swiss-Prot;Acc:Q6SA80] | 1 |
| 1377794\_at |  | 360574 | ENSRNOG00000014209 | ENSRNOT00000019044 | RGD1311017\_predicted |  | 1 |
| 1388544\_at |  | 296973 | ENSRNOG00000010133 | ENSRNOT00000013569 | Bpgm | 2,3-bisphosphoglycerate mutase [Source:RefSeq\_peptide;Acc:NP\_955414] | 1 |
| 1377593\_at |  | 296394 | ENSRNOG00000010993 | ENSRNOT00000016150 | NP\_001100014.1 | dolichol-phosphate (beta-D) mannosyltransferase 1 [Source:RefSeq\_peptide;Acc:NP\_001100014] | 1 |
| 1372667\_at | Q68FU5 | 361740 | ENSRNOG00000024197  ENSRNOG00000027161 | ENSRNOT00000029509  ENSRNOT00000037500 | RGD1359158 | Uncharacterized protein C9orf85 homolog. [Source:UniProtKB/Swiss-Prot;Acc:Q68FU5] | 1 |
| 1367818\_at | Q63159 | 29309 | ENSRNOG00000009974 | ENSRNOT00000013384 | Coq3 | Hexaprenyldihydroxybenzoate methyltransferase, mitochondrial precursor (EC 2.1.1.114) (Dihydroxyhexaprenylbenzoate methyltransferase) (3,4- dihydroxy-5-hexaprenylbenzoate methyltransferase) (DHHB methyltransferase) (DHHB-MT) (DHHB-MTase). [Source:UniProtKB/Swiss-Prot;Acc:Q63159] | 1 |
| 1378668\_at |  |  | ENSRNOG00000031995 | ENSRNOT00000047282 | 2810008M24Rik | RIKEN cDNA 2810008M24 gene Gene [Source:MGI Symbol;Acc:MGI:1922866] | 1 |
| 1372320\_at |  | 317464 | ENSRNOG00000004016 | ENSRNOT00000005457 | Msl31 | male-specific lethal-3 homolog 1 [Source:RefSeq\_peptide;Acc:NP\_001014133] | 1 |
| 1389941\_at | Q4V8C5 | 498910 | ENSRNOG00000016991 | ENSRNOT00000023455 | Arl2bp | ADP-ribosylation factor-like protein 2-binding protein (ARF-like 2- binding protein) (Binder of ARF2 protein 1). [Source:UniProtKB/Swiss-Prot;Acc:Q4V8C5] | 1 |
| 1388983\_at |  | 296050 | ENSRNOG00000005884 | ENSRNOT00000008364 | NP\_001099966.1 |  | 1 |
| 1371623\_at |  | 684207  685888 | ENSRNOG00000028087 | ENSRNOT00000035033 | LOC685888 |  | 1 |
| 1370228\_at | P12346 | 300965  24825 | ENSRNOG00000030625 | ENSRNOT00000039884  ENSRNOT00000012725  ENSRNOT00000012946  ENSRNOT00000045628 | Tf | Serotransferrin precursor (Transferrin) (Siderophilin) (Beta-1-metal- binding globulin) (Liver regeneration-related protein LRRG03). [Source:UniProtKB/Swiss-Prot;Acc:P12346] | 1 |
| 1398784\_at | O35796 | 29681 | ENSRNOG00000006949 | ENSRNOT00000037517 | C1qbp | Complement component 1 Q subcomponent-binding protein, mitochondrial precursor (Glycoprotein gC1qBP) (C1qBP) (GC1q-R protein). [Source:UniProtKB/Swiss-Prot;Acc:O35796] | 1 |
| 1388603\_a\_at | Q80W96 | 685052  681980  290985 | ENSRNOG00000018343 | ENSRNOT00000024781 | Hbld2 | Iron-sulfur cluster assembly 1 homolog, mitochondrial precursor (HESB- like domain-containing protein 2) (Iron sulfur assembly protein IscA). [Source:UniProtKB/Swiss-Prot;Acc:Q80W96] | 1 |
| 1394884\_s\_at | P28042 | 54304 | ENSRNOG00000012100 | ENSRNOT00000016217 | Ssbp1 | Single-stranded DNA-binding protein, mitochondrial precursor (Mt-SSB) (MtSSB) (P16). [Source:UniProtKB/Swiss-Prot;Acc:P28042] | 1 |
| 1372605\_at |  | 500005 | ENSRNOG00000008613 | ENSRNOT00000061122  ENSRNOT00000011666 | LOC500005 | LOC500005 protein (Fragment). [Source:UniProtKB/TrEMBL;Acc:Q5M7V0] | 1 |
| 1377631\_at |  | 362285 | ENSRNOG00000009531 | ENSRNOT00000013105 | NP\_001102081.1 | procollagen, type IX, alpha 3 [Source:RefSeq\_peptide;Acc:NP\_001102081] | 1 |
| 1388727\_at | Q6YDN7 | 366381 | ENSRNOG00000029785 | ENSRNOT00000040894 | Cdc26 | Anaphase-promoting complex subunit CDC26 (Cell division cycle protein 26) (Protein BWK-2). [Source:UniProtKB/Swiss-Prot;Acc:Q6YDN7] | 1 |
| 1398917\_at | P05426 | 297755 | ENSRNOG00000006992  ENSRNOG00000013425  ENSRNOG00000012492 | ENSRNOT00000009431  ENSRNOT00000017930  ENSRNOT00000016662 | Rpl7    LOC292518 | 60S ribosomal protein L7. [Source:UniProtKB/Swiss-Prot;Acc:P05426] | 1 |
| 1367730\_at | P41542 | 56042 | ENSRNOG00000002301 | ENSRNOT00000003277 | Vdp | General vesicular transport factor p115 (Protein USO1 homolog) (Transcytosis-associated protein) (TAP) (Vesicle-docking protein). [Source:UniProtKB/Swiss-Prot;Acc:P41542] | 1 |
| 1399114\_at |  | 306516 | ENSRNOG00000014422 | ENSRNOT00000058872 | NP\_001100788.2 | general transcription factor II E, polypeptide 2 (beta subunit) [Source:RefSeq\_peptide;Acc:NP\_001100788] | 1 |
| 1399081\_at |  | 305549  499085  691164 | ENSRNOG00000006329 | ENSRNOT00000009319 | Peli1 | Peli1 protein (Fragment). [Source:UniProtKB/TrEMBL;Acc:Q562B8] | 1 |
| 1377125\_at |  |  | ENSRNOG00000011968 | ENSRNOT00000016033 | Dnajc6 | DnaJ (Hsp40) homolog, subfamily C, member 6 Gene [Source:MGI (curated);Acc:Dnajc6-001] | 1 |
| 1373672\_at |  |  | ENSRNOG00000033772 | ENSRNOT00000059950  ENSRNOT00000022194 | MGC94010 | similar to SPI6 [Source:RefSeq\_peptide;Acc:NP\_001007733] | 1 |
| 1372563\_at |  |  | ENSRNOG00000011613 | ENSRNOT00000056439 | Q5XI45\_RAT | RGD1308143 protein (Fragment). [Source:UniProtKB/TrEMBL;Acc:Q5XI45] | 1 |
| 1398804\_at | Q6DKG0 | 64472 | ENSRNOG00000018417 | ENSRNOT00000025096 | Mak10 | Protein MAK10 homolog (Embryonic growth-associated protein) (Corneal wound-healing-related protein). [Source:UniProtKB/Swiss-Prot;Acc:Q6DKG0] | 1 |
| 1390306\_at |  | 299236 | ENSRNOG00000003732 | ENSRNOT00000004955 | NP\_001100220.1 | fibronectin leucine rich transmembrane protein 2 [Source:RefSeq\_peptide;Acc:NP\_001100220] | 1 |
| 1368043\_at | Q99N27 | 84471 | ENSRNOG00000017029 | ENSRNOT00000022960 | Snx1 | Sorting nexin-1. [Source:UniProtKB/Swiss-Prot;Acc:Q99N27] | 1 |
| 1375934\_at |  | 315911 | ENSRNOG00000011120 | ENSRNOT00000016100 | LOC679381 |  | 1 |
| 1372115\_at |  | 363188 | ENSRNOG00000015813 | ENSRNOT00000061531 | LOC363188 |  | 1 |
| 1369200\_at | P21588 | 58813 | ENSRNOG00000011071 | ENSRNOT00000015057 | Nt5e | 5'-nucleotidase precursor (EC 3.1.3.5) (Ecto-5'-nucleotidase) (5'-NT) (CD73 antigen). [Source:UniProtKB/Swiss-Prot;Acc:P21588] | 1 |
| 1379626\_at |  | 316164 | ENSRNOG00000012942 | ENSRNOT00000017556 | Satb1 | special AT-rich sequence binding protein 1 [Source:RefSeq\_peptide;Acc:NP\_001012129] | 1 |
| 1389064\_at |  | 302288 | ENSRNOG00000003578 | ENSRNOT00000004784 | NP\_001100402.1 | fem-1 homolog c [Source:RefSeq\_peptide;Acc:NP\_001100402] | 1 |
| 1388909\_at |  | 306270 | ENSRNOG00000019760 | ENSRNOT00000026751 | NP\_001100765.1 | oxidoreductase NAD-binding domain containing 1 [Source:RefSeq\_peptide;Acc:NP\_001100765] | 1 |
| 1388105\_at | Q62834 | 116656 | ENSRNOG00000017770 | ENSRNOT00000024016 | D123 | Cell division cycle protein 123 homolog (Protein D123). [Source:UniProtKB/Swiss-Prot;Acc:Q62834] | 1 |
| 1384394\_at |  |  | ENSRNOG00000002039 | ENSRNOT00000061630  ENSRNOT00000002788 | Evi5 | ecotropic viral integration site 5 Gene [Source:MGI (curated);Acc:Evi5-001] | 1 |
| 1399107\_at |  | 297971 | ENSRNOG00000023035 | ENSRNOT00000035840 | NP\_001100114.1 | similar to RIKEN cDNA 1810030N24 (predicted) (RGD1305158\_predicted), mRNA [Source:RefSeq\_dna;Acc:NM\_001106644] | 1 |
| 1386247\_at |  | 306471 | ENSRNOG00000011116 | ENSRNOT00000014828 | Snx25 | Snx25 protein (Fragment). [Source:UniProtKB/TrEMBL;Acc:Q5RJT4] | 1 |
| 1387911\_at | Q08603 | 25533 | ENSRNOG00000009992 | ENSRNOT00000013660 | Rabggtb | Geranylgeranyl transferase type-2 subunit beta (EC 2.5.1.60) (Geranylgeranyl transferase type II subunit beta) (Rab geranylgeranyltransferase subunit beta) (Rab geranyl- geranyltransferase subunit beta) (Rab GG transferase beta) (Rab GGTase beta). [Source:UniProtKB/Swiss-Prot;Acc:Q08603] | 1 |
| 1371052\_at | Q62809 | 25495 | ENSRNOG00000023683 | ENSRNOT00000032388 | Nog | Noggin precursor (Fragment). [Source:UniProtKB/Swiss-Prot;Acc:Q62809] | 1 |
| 1367992\_at | P27682 | 25719 | ENSRNOG00000007542 | ENSRNOT00000010679 | Sgne1 | Neuroendocrine protein 7B2 precursor (Secretogranin-5) (Secretogranin V) (Secretory granule endocrine protein I) [Contains: N-terminal peptide; C-terminal peptide]. [Source:UniProtKB/Swiss-Prot;Acc:P27682] | 1 |
| 1389782\_at |  | 679430  294499  683599 | ENSRNOG00000000551 | ENSRNOT00000000662 | RGD1305587\_predicted |  | 1 |
| 1373083\_at | Q66H88 | 619549 | ENSRNOG00000015268 | ENSRNOT00000020475 | Ppapdc2 | Presqualene diphosphate phosphatase (EC 3.1.3.-) (Phosphatidic acid phosphatase type 2 domain-containing protein 2). [Source:UniProtKB/Swiss-Prot;Acc:Q66H88] | 1 |
| 1379694\_at |  | 362132 | ENSRNOG00000029447 | ENSRNOT00000051953 | RGD1561337\_predicted | enhancer of polycomb homolog 2 [Source:RefSeq\_peptide;Acc:NP\_001102051] | 1 |
| 1381972\_at | Q56AP7 | 297498 | ENSRNOG00000006534 | ENSRNOT00000008873 | Crbn | Protein cereblon. [Source:UniProtKB/Swiss-Prot;Acc:Q56AP7] | 1 |
| 1386088\_at | Q5EB62 | 291709 | ENSRNOG00000017091 | ENSRNOT00000023032  ENSRNOT00000061094 | RGD1305072 | Solute carrier family 25 member 46. [Source:UniProtKB/Swiss-Prot;Acc:Q5EB62] | 1 |
| 1376630\_at |  |  | ENSRNOG00000024460 | ENSRNOT00000033822 | Tarsl2 | threonyl-tRNA synthetase-like 2 [Source:RefSeq\_peptide;Acc:NP\_001014042] | 1 |
| 1384763\_at |  | 362319 | ENSRNOG00000008585 | ENSRNOT00000011377 | RGD1306626 | similar to RIKEN cDNA 4930500J03 (RGD1306626), mRNA [Source:RefSeq\_dna;Acc:NM\_001039612] | 1 |
| 1391559\_at | Q5U2T1 | 287472 | ENSRNOG00000012579 | ENSRNOT00000016840 | Tlcd1 | TLC domain-containing protein 1 precursor. [Source:UniProtKB/Swiss-Prot;Acc:Q5U2T1] | 1 |
| 1368565\_at | P24942 | 29483 | ENSRNOG00000016163 | ENSRNOT00000022319 | Slc1a3 | Excitatory amino acid transporter 1 (Solute carrier family 1 member 3) (Sodium-dependent glutamate/aspartate transporter 1) (GLAST-1) (Glial glutamate transporter). [Source:UniProtKB/Swiss-Prot;Acc:P24942] | 1 |
| 1368536\_at |  |  | ENSRNOG00000004089 | ENSRNOT00000051139  ENSRNOT00000005561 | Enpp2 | ectonucleotide pyrophosphatase/phosphodiesterase 2 Gene [Source:MGI Symbol;Acc:MGI:1321390] | 1 |
| 1378092\_at |  | 290230 | ENSRNOG00000019840 | ENSRNOT00000026914 | NP\_001099509.1 | similar to magnesium-dependent phosphatase-1 (predicted) (RGD1311147\_predicted), mRNA [Source:RefSeq\_dna;Acc:NM\_001106039] | 1 |
| 1379590\_at |  | 498425 | ENSRNOG00000009639 | ENSRNOT00000012767 | LOC498425 | similar to U2 small nuclear ribonucleoprotein auxiliary factor 35 kDa subunit related-protein 1 (U2(RNU2) small nuclear RNA auxillary factor 1-like 1) (SP2) (LOC498425), mRNA [Source:RefSeq\_dna;Acc:NM\_001017504] | 1 |
| 1388937\_at |  | 362900 | ENSRNOG00000009658 | ENSRNOT00000013344 | Rnf19\_predicted |  | 1 |
| 1373812\_at |  | 83571 | ENSRNOG00000007249 | ENSRNOT00000010222  ENSRNOT00000049848 | Cdkn1b | cyclin-dependent kinase inhibitor 1B (p27, kip1) [Source:RefSeq\_peptide;Acc:NP\_113950] | 1 |
| 1371483\_at |  | 310378 | ENSRNOG00000026842 | ENSRNOT00000033627 | Nnt | nicotinamide nucleotide transhydrogenase [Source:RefSeq\_peptide;Acc:NP\_001013175] | 1 |
| 1382726\_at |  | 364952 | ENSRNOG00000014293 | ENSRNOT00000019301 | NP\_001102364.1 | naked cuticle 1 homolog [Source:RefSeq\_peptide;Acc:NP\_001102364] | 1 |
| 1373362\_at | P32551 | 293448 | ENSRNOG00000036742 | ENSRNOT00000021514 | Uqcrc2 | Cytochrome b-c1 complex subunit 2, mitochondrial precursor (Ubiquinol- cytochrome-c reductase complex core protein 2) (Core protein II) (Complex III subunit 2). [Source:UniProtKB/Swiss-Prot;Acc:P32551] | 1 |
| 1368249\_at |  | 85497 | ENSRNOG00000017808 | ENSRNOT00000024011  ENSRNOT00000057198 | Klf15 | Kruppel-like factor 15 [Source:RefSeq\_peptide;Acc:NP\_445988] | 1 |
| 1371634\_at | Q5HZA9 | 293113 | ENSRNOG00000022748 | ENSRNOT00000038333 | RGD1305677 | Transmembrane protein 126A. [Source:UniProtKB/Swiss-Prot;Acc:Q5HZA9] | 1 |
| 1379262\_at |  | 302640 | ENSRNOG00000003782 | ENSRNOT00000005033 | LOC302640 | acyl-Coenzyme A thioesterase 2, mitochondrial [Source:RefSeq\_peptide;Acc:NP\_001013982] | 1 |
| 1368104\_at | Q9JJW1 | 64521 | ENSRNOG00000023338 | ENSRNOT00000035605 | Tspan2 | Tetraspanin-2 (Tspan-2). [Source:UniProtKB/Swiss-Prot;Acc:Q9JJW1] | 1 |
| 1387078\_at | Q62784 | 80849 | ENSRNOG00000017660 | ENSRNOT00000051918 | Inpp4a | Type I inositol-3,4-bisphosphate 4-phosphatase (EC 3.1.3.66) (Inositol polyphosphate 4-phosphatase type I). [Source:UniProtKB/Swiss-Prot;Acc:Q62784] | 1 |
| 1378394\_at |  | 361344 | ENSRNOG00000018648 | ENSRNOT00000025164 | NP\_001101905.1 | metallophosphoesterase 1 [Source:RefSeq\_peptide;Acc:NP\_001101905] | 1 |
| 1388497\_at |  | 291135 | ENSRNOG00000018415 | ENSRNOT00000024878 | NP\_001099581.1 | thioesterase superfamily member 2 [Source:RefSeq\_peptide;Acc:NP\_001099581] | 1 |
| 1388529\_at |  | 295395 | ENSRNOG00000014575 | ENSRNOT00000020149 | Rtcd1 | RNA terminal phosphate cyclase domain 1 [Source:RefSeq\_peptide;Acc:NP\_001004227] | 1 |
| 1371475\_at | O55004 | 56759 | ENSRNOG00000025625 | ENSRNOT00000041495 | Rnase4 | Ribonuclease 4 precursor (EC 3.1.27.-) (RNase 4) (RL3). [Source:UniProtKB/Swiss-Prot;Acc:O55004] | 1 |
| 1371418\_at | Q5XIM9 | 299809 | ENSRNOG00000021317 | ENSRNOT00000037946 | Cct2 | T-complex protein 1 subunit beta (TCP-1-beta) (CCT-beta). [Source:UniProtKB/Swiss-Prot;Acc:Q5XIM9] | 1 |
| 1386953\_at | P16232 | 25116 | ENSRNOG00000005861 | ENSRNOT00000007870 | Hsd11b1 | Corticosteroid 11-beta-dehydrogenase isozyme 1 (EC 1.1.1.146) (11-DH) (11-beta-hydroxysteroid dehydrogenase 1) (11-beta-HSD1). [Source:UniProtKB/Swiss-Prot;Acc:P16232] | 1 |
| 1384526\_at |  | 290803 | ENSRNOG00000007748 | ENSRNOT00000034325 | LOC689179 | testis expressed gene 15 [Source:RefSeq\_peptide;Acc:NP\_001099557] | 1 |
| 1385391\_at | Q66HB6 | 306872 | ENSRNOG00000024111 | ENSRNOT00000038349 | Ctag3 | Cancer-associated gene 1 protein homolog (CAGE-1) (Cancer/testis antigen 3 homolog) (CT3 homolog). [Source:UniProtKB/Swiss-Prot;Acc:Q66HB6] | 1 |
| 1375185\_at |  | 308939 | ENSRNOG00000010427 | ENSRNOT00000014002 | NP\_001101015.1 | importin 7 [Source:RefSeq\_peptide;Acc:NP\_001101015] | 1 |
| 1395508\_at | Q68FQ0 | 294864 | ENSRNOG00000011632 | ENSRNOT00000015886 | Cct5 | T-complex protein 1 subunit epsilon (TCP-1-epsilon) (CCT-epsilon). [Source:UniProtKB/Swiss-Prot;Acc:Q68FQ0] | 1 |
| 1393335\_at |  |  | ENSRNOG00000004275 | ENSRNOT00000005968 | Egfl6 | EGF-like-domain, multiple 6 Gene [Source:MGI (curated);Acc:Egfl6-001] | 1 |
| 1393800\_at |  |  | ENSRNOG00000003187 | ENSRNOT00000004252 | Armcx5 | armadillo repeat containing, X-linked 5 Gene [Source:MGI (curated);Acc:Armcx5-001] | 1 |
| 1372184\_at |  | 315463 | ENSRNOG00000009255 | ENSRNOT00000012269 | NP\_001101599.1 |  | 1 |
| 1376261\_at | Q5QJC9 | 366734 | ENSRNOG00000011527 | ENSRNOT00000015333 | Bag5 | BAG family molecular chaperone regulator 5 (Bcl-2-associated athanogene 5) (BAG-5). [Source:UniProtKB/Swiss-Prot;Acc:Q5QJC9] | 1 |
| 1374892\_at |  | 360547 | ENSRNOG00000011714 | ENSRNOT00000015769 | NP\_001101748.1 | spermidine/spermine N1-acetyl transferase 2 [Source:RefSeq\_peptide;Acc:NP\_001101748] | 1 |
| 1371443\_at | Q5M951 | 362671 | ENSRNOG00000025034 | ENSRNOT00000032859 | RGD1304567 | Uncharacterized protein C1orf174 homolog. [Source:UniProtKB/Swiss-Prot;Acc:Q5M951] | 1 |
| 1374418\_at |  | 291914 | ENSRNOG00000015534 | ENSRNOT00000020816 | NP\_001099643.1 | similar to CG8009-PA (predicted) (RGD1308816\_predicted), mRNA [Source:RefSeq\_dna;Acc:NM\_001106173] | 1 |
| 1389970\_at |  |  | ENSRNOG00000001852 | ENSRNOT00000002531 | Ergic2 | ERGIC and golgi 2 Gene [Source:MGI (curated);Acc:Ergic2-001] | 1 |
| 1373448\_at |  | 299203 | ENSRNOG00000006744 | ENSRNOT00000009086 | NP\_001100216.1 | acylphosphatase 1, erythrocyte (common) type [Source:RefSeq\_peptide;Acc:NP\_001100216] | 1 |
| 1385663\_at |  | 310306 | ENSRNOG00000030639 | ENSRNOT00000048030  ENSRNOT00000041426  ENSRNOT00000042873 | NP\_001101135.1 | ubiquitin specific protease 13 (isopeptidase T-3) [Source:RefSeq\_peptide;Acc:NP\_001101135] | 1 |
| 1373846\_at |  | 305284  683767  366434 | ENSRNOG00000024829  ENSRNOG00000008512 | ENSRNOT00000039626  ENSRNOT00000011272 | RGD1561589\_predicted  RGD1311774\_predicted |  | 1 |
| 1389510\_at | Q6AYK5 | 289707 | ENSRNOG00000005374 | ENSRNOT00000007154 | Lyar | Cell growth-regulating nucleolar protein. [Source:UniProtKB/Swiss-Prot;Acc:Q6AYK5] | 1 |
| 1377014\_at |  | 310376 | ENSRNOG00000016353 | ENSRNOT00000021964 | RGD1308116\_predicted |  | 1 |
| 1379471\_at |  | 305268 | ENSRNOG00000021931 | ENSRNOT00000037509 | NP\_001100683.1 | similar to RIKEN cDNA 5730469D23 (predicted) (RGD1308324\_predicted), mRNA [Source:RefSeq\_dna;Acc:NM\_001107213] | 1 |
| 1382646\_at |  | 291078 | ENSRNOG00000016705 | ENSRNOT00000022902 | Prpf4b | PRP4 pre-mRNA processing factor 4 homolog B [Source:RefSeq\_peptide;Acc:NP\_001011923] | 1 |
| 1398886\_at |  |  | ENSRNOG00000037534 | ENSRNOT00000056857 | 2700094K13Rik | RIKEN cDNA 2700094K13 gene Gene [Source:MGI Symbol;Acc:MGI:1919907] | 1 |
| 1393101\_at |  |  | ENSRNOG00000025702 | ENSRNOT00000044071 | Fbxl10 | Fbxl10 protein (Fragment). [Source:UniProtKB/TrEMBL;Acc:Q641Z3] | 1 |
| 1386904\_a\_at | P00173 | 64001 | ENSRNOG00000015205 | ENSRNOT00000020446 | Cyb5 | Cytochrome b5. [Source:UniProtKB/Swiss-Prot;Acc:P00173] | 1 |
| 1386691\_at |  | 363268 | ENSRNOG00000017053 | ENSRNOT00000022901 | NP\_001102274.1 | F-box only protein 36 [Source:RefSeq\_peptide;Acc:NP\_001102274] | 1 |
| 1387497\_at | Q63634 | 25340 | ENSRNOG00000014172 | ENSRNOT00000018976 | Npy5r | Neuropeptide Y receptor type 5 (NPY5-R) (NPY-Y5 receptor) (Y5 receptor). [Source:UniProtKB/Swiss-Prot;Acc:Q63634] | 1 |
| 1388170\_at | Q8R4G8 | 291772 | ENSRNOG00000016467 | ENSRNOT00000022320  ENSRNOT00000061504 | Kctd1 | BTB/POZ domain-containing protein KCTD1 (Vitamin A-deficient testicular protein 6). [Source:UniProtKB/Swiss-Prot;Acc:Q8R4G8] | 1 |
| 1372873\_at |  | 307390 | ENSRNOG00000019063 | ENSRNOT00000025790 | NP\_001100855.1 | F-box protein 38 [Source:RefSeq\_peptide;Acc:NP\_001100855] | 1 |
| 1391456\_at | Q68FQ2 | 315509 | ENSRNOG00000009149 | ENSRNOT00000012247 | Jam3 | Junctional adhesion molecule C precursor (JAM-C) (Junctional adhesion molecule 3) (JAM-3). [Source:UniProtKB/Swiss-Prot;Acc:Q68FQ2] | 1 |
| 1372557\_at |  |  | ENSRNOG00000001689 | ENSRNOT00000043337 | NP\_001102312.1 | ADP-ribosylation factor-like 6 [Source:RefSeq\_peptide;Acc:NP\_001102312] | 1 |
| 1371845\_at | Q5M882 | 292831 | ENSRNOG00000027646 | ENSRNOT00000020474 | Pop4 | Ribonuclease P protein subunit p29 (EC 3.1.26.5). [Source:UniProtKB/Swiss-Prot;Acc:Q5M882] | 1 |
| 1389323\_at | Q4V7A0 | 363064 | ENSRNOG00000012803 | ENSRNOT00000017239 | Wdr61 | WD repeat-containing protein 61. [Source:UniProtKB/Swiss-Prot;Acc:Q4V7A0] | 1 |
| 1371464\_at | Q6DGF4 | 293067 | ENSRNOG00000013506 | ENSRNOT00000018098 | Za20d3 | AN1-type zinc finger protein 6 (Zinc finger A20 domain-containing protein 3). [Source:UniProtKB/Swiss-Prot;Acc:Q6DGF4] | 1 |
| 1376592\_at |  | 293829 | ENSRNOG00000016327 | ENSRNOT00000021884 | NP\_001099811.1 | methylmalonyl CoA epimerase [Source:RefSeq\_peptide;Acc:NP\_001099811] | 1 |
| 1391653\_at |  |  | ENSRNOG00000003241 | ENSRNOT00000004658 | Gabrg2 | Gamma-aminobutyric acid receptor subunit gamma-2 precursor (GABA(A) receptor subunit gamma-2). [Source:UniProtKB/Swiss-Prot;Acc:P18508] | 1 |
| 1373484\_at |  | 313445  313348 | ENSRNOG00000014029 | ENSRNOT00000018788 | Klhl13 | kelch-like 9 [Source:RefSeq\_peptide;Acc:NP\_001101414] | 1 |
| 1374481\_at |  | 364183 | ENSRNOG00000006207 | ENSRNOT00000008127 | Cno | Cno protein (Fragment). [Source:UniProtKB/TrEMBL;Acc:Q5BJZ8] | 1 |
| 1393148\_at |  |  | ENSRNOG00000002480 | ENSRNOT00000003503 | NP\_001099448.1 | G protein-coupled receptor 137B [Source:RefSeq\_peptide;Acc:NP\_001099448] | 1 |
| 1382469\_at |  | 317423 | ENSRNOG00000002654 | ENSRNOT00000003565 | RGD1564253\_predicted |  | 1 |
| 1368132\_at | Q8R5K6 | 170842 | ENSRNOG00000002828 | ENSRNOT00000003780 | Tob1 | Protein Tob1 (Transducer of erbB-2 1). [Source:UniProtKB/Swiss-Prot;Acc:Q8R5K6] | 1 |
| 1388700\_at |  | 314374 | ENSRNOG00000004709 | ENSRNOT00000006604 | NP\_001101517.1 | checkpoint suppressor 1 [Source:RefSeq\_peptide;Acc:NP\_001101517] | 1 |
| 1371695\_at |  | 304862 | ENSRNOG00000002394 | ENSRNOT00000003318 | Tpr | translocated promoter region [Source:RefSeq\_peptide;Acc:NP\_001100655] | 1 |
| 1398473\_at | Q32WR5 | 293938 | ENSRNOG00000012684 | ENSRNOT00000017006 | Bloc1s2 | Biogenesis of lysosome-related organelles complex-1 subunit 2 (Spinal cord-expressed protein 1). [Source:UniProtKB/Swiss-Prot;Acc:Q32WR5] | 1 |
| 1372817\_at | Q62981 | 53982 | ENSRNOG00000020762 | ENSRNOT00000049877 | Zfp260 | Zinc finger protein 260 (Zfp-260) (POZF-1) (Pancreas-only zinc finger protein 1). [Source:UniProtKB/Swiss-Prot;Acc:Q62981] | 1 |
| 1383090\_at |  | 289143 | ENSRNOG00000002569 | ENSRNOT00000003460 | NP\_001099433.1 | mitochondrial ribosomal protein S14 [Source:RefSeq\_peptide;Acc:NP\_001099433] | 1 |
| 1376028\_at |  | 303514 | ENSRNOG00000009643 | ENSRNOT00000055330 | NP\_001100518.1 | similar to RIKEN cDNA 4121402D02 (predicted) (RGD1564778\_predicted), mRNA [Source:RefSeq\_dna;Acc:NM\_001107048] | 1 |
| 1392474\_at |  | 303206 | ENSRNOG00000002763 | ENSRNOT00000003792 | Ulk2 | Unc-51 like kinase 2 (C. elegans) Gene [Source:MGI (curated);Acc:Ulk2-001] | 1 |
| 1375638\_at | Q66H98 | 316384 | ENSRNOG00000025895 | ENSRNOT00000028907 | Sdpr | Serum deprivation-response protein (Phosphatidylserine-binding protein). [Source:UniProtKB/Swiss-Prot;Acc:Q66H98] | 1 |
| 1398941\_at |  |  | ENSRNOG00000006713  ENSRNOG00000012044  ENSRNOG00000037078 | ENSRNOT00000031596  ENSRNOT00000045980  ENSRNOT00000016081  ENSRNOT00000058670  ENSRNOT00000037460 | LOC498647  LOC367191 |  | 1 |
| 1398244\_at | Q4QRB2 | 170933 | ENSRNOG00000017742 | ENSRNOT00000024271 | Syf2 | Pre-mRNA-splicing factor SYF2. [Source:UniProtKB/Swiss-Prot;Acc:Q4QRB2] | 1 |
| 1396215\_at | Q5U1Z8 | 502782 | ENSRNOG00000006858 | ENSRNOT00000008973 | LOC502782 | Protein preY, mitochondrial precursor. [Source:UniProtKB/Swiss-Prot;Acc:Q5U1Z8] | 1 |
| 1372659\_at |  | 684322 | ENSRNOG00000015097 | ENSRNOT00000020671 | LOC684322 |  | 1 |
| 1372324\_at |  |  | ENSRNOG00000027818 | ENSRNOT00000039481 | Znhit3 | zinc finger, HIT type 3 Gene [Source:MGI (curated);Acc:Znhit3-001] | 1 |
| 1376861\_at |  | 686087 | ENSRNOG00000008840 | ENSRNOT00000011995 | LOC686087 | similar to RIKEN cDNA 1810018L05 (LOC317312), mRNA [Source:RefSeq\_dna;Acc:NM\_001014107] | 1 |
| 1370807\_at | Q91ZQ0 | 192129 | ENSRNOG00000003967 | ENSRNOT00000005658 | Tmem49 | Transmembrane protein 49 (Vacuole membrane protein 1). [Source:UniProtKB/Swiss-Prot;Acc:Q91ZQ0] | 1 |
| 1386861\_at | P0C0S7 | 58940 | ENSRNOG00000038375  ENSRNOG00000010306 | ENSRNOT00000058436  ENSRNOT00000013919 | H2afz | Histone H2A.Z (H2A/z). [Source:UniProtKB/Swiss-Prot;Acc:P0C0S7] | 1 |
| 1385839\_x\_at |  | 362545 | ENSRNOG00000007527 | ENSRNOT00000009897 | NP\_001102140.1 | TM2 domain containing 1 [Source:RefSeq\_peptide;Acc:NP\_001102140] | 1 |
| 1372538\_at |  | 300083 | ENSRNOG00000018920 | ENSRNOT00000025758 | Slc25a17\_predicted |  | 1 |
| 1373868\_at |  |  | ENSRNOG00000013052 | ENSRNOT00000017929 | Q7TP73\_RAT | Aa2-041. [Source:UniProtKB/TrEMBL;Acc:Q7TP73] | 1 |
| 1378100\_at |  | 299810 | ENSRNOG00000005689 | ENSRNOT00000007580 | Yeats4\_predicted |  | 1 |
| 1380182\_at |  |  | ENSRNOG00000008666 | ENSRNOT00000057655 | RGD1563437\_predicted |  | 1 |
| 1371752\_at |  | 287419 | ENSRNOG00000004980 | ENSRNOT00000006883 | RGD1563527\_predicted | similar to Ran-interacting protein MOG1 (predicted) (RGD1563195\_predicted), mRNA [Source:RefSeq\_dna;Acc:NM\_001105790] | 1 |
| 1370384\_a\_at |  |  | ENSRNOG00000017727 | ENSRNOT00000059975  ENSRNOT00000059973  ENSRNOT00000059970  ENSRNOT00000023941 | Prlr | prolactin receptor Gene [Source:MGI Symbol;Acc:MGI:97763] | 1 |
| 1385296\_at | Q4V8B8 | 314999 | ENSRNOG00000008978 | ENSRNOT00000011876 | Trmt12 | tRNA wybutosine-synthesizing protein 2 homolog (EC 2.1.1.-) (tRNA-yW- synthesizing protein 2) (tRNA methyltransferase 12). [Source:UniProtKB/Swiss-Prot;Acc:Q4V8B8] | 1 |
| 1371404\_at |  |  | ENSRNOG00000010103 | ENSRNOT00000014033 | Eif4b | eukaryotic translation initiation factor 4B [Source:RefSeq\_peptide;Acc:NP\_001008325] | 1 |
| 1368208\_at | Q9JIY6 | 171084 | ENSRNOG00000015851 | ENSRNOT00000021243 | RGD621605 | Probable N-acetyltransferase CML6 (EC 2.3.1.-) (Camello-like protein 6) (Camello-like protein 1). [Source:UniProtKB/Swiss-Prot;Acc:Q9JIY6] | 1 |
| 1374111\_at |  | 368070 | ENSRNOG00000004061 | ENSRNOT00000005439 | NP\_001102493.1 | similar to Pinin (LOC368070), mRNA [Source:RefSeq\_dna;Acc:NM\_001109023] | 1 |
| 1371639\_at |  | 288666 | ENSRNOG00000001274 | ENSRNOT00000001717 | NP\_001099402.1 | vacuolar protein sorting 29 [Source:RefSeq\_peptide;Acc:NP\_001099402] | 1 |
| 1389551\_at | Q561R9 | 297768 | ENSRNOG00000007829 | ENSRNOT00000010369 | Lactb2 | Beta-lactamase-like protein 2 (EC 3.-.-.-). [Source:UniProtKB/Swiss-Prot;Acc:Q561R9] | 1 |
| 1378191\_at | Q56A27 | 298075 | ENSRNOG00000023605 | ENSRNOT00000030825 | Ncbp1 | Nuclear cap-binding protein subunit 1. [Source:UniProtKB/Swiss-Prot;Acc:Q56A27] | 1 |
| 1372834\_at |  | 360834 | ENSRNOG00000002705 | ENSRNOT00000003715 | Vps4b | vacuolar protein sorting 4b [Source:RefSeq\_peptide;Acc:NP\_001020887] | 1 |
| 1386571\_at |  |  | ENSRNOG00000002474 | ENSRNOT00000003369 | Tom1l1 | target of myb1-like 1 (chicken) Gene [Source:MGI (curated);Acc:Tom1l1-008] | 1 |
| 1390937\_at | Q68FW6 | 299153 | ENSRNOG00000038480 | ENSRNOT00000058642 | RGD1309051 | Uncharacterized protein C14orf50 homolog. [Source:UniProtKB/Swiss-Prot;Acc:Q68FW6] | 1 |
| 1375851\_at |  | 289457 | ENSRNOG00000002214 | ENSRNOT00000003026 | NP\_001099465.1 | kelch-like 8 [Source:RefSeq\_peptide;Acc:NP\_001099465] | 1 |
| 1372289\_at |  | 311122 | ENSRNOG00000022922 | ENSRNOT00000036728 | Slc25a12 | solute carrier family 25 (mitochondrial carrier, Aralar), member 12 Gene [Source:MGI (curated);Acc:Slc25a12-001] | 1 |
| 1382186\_a\_at | Q566R3 | 295228 | ENSRNOG00000018969 | ENSRNOT00000056923  ENSRNOT00000039340  ENSRNOT00000025940 | RGD1311086 | G patch domain-containing protein 4. [Source:UniProtKB/Swiss-Prot;Acc:Q566R3] | 1 |
| 1383824\_at |  | 289664 | ENSRNOG00000003205 | ENSRNOT00000058803 | NP\_001099479.1 | LIM domain binding 2 [Source:RefSeq\_peptide;Acc:NP\_001099479] | 1 |
| 1398879\_at | Q6AYN2 | 290796 | ENSRNOG00000012329 | ENSRNOT00000016792 | Tmem66 | Transmembrane protein 66 precursor. [Source:UniProtKB/Swiss-Prot;Acc:Q6AYN2] | 1 |
| 1394093\_at |  | 307766 | ENSRNOG00000018149 | ENSRNOT00000024568 | NP\_001100889.1 | SWI/SNF related, matrix associated, actin dependent regulator of chromatin, subfamily a, member 5 [Source:RefSeq\_peptide;Acc:NP\_001100889] | 1 |
| 1383354\_a\_at |  |  | ENSRNOG00000005285 | ENSRNOT00000007221 | Fbxo33 | F-box protein 33 Gene [Source:MGI Symbol;Acc:MGI:1917861] | 1 |
| 1372248\_at |  |  | ENSRNOG00000000302 | ENSRNOT00000000330 | Sesn1 | sestrin 1 Gene [Source:MGI (curated);Acc:Sesn1-001] | 1 |
| 1376418\_a\_at |  | 306804 | ENSRNOG00000014616 | ENSRNOT00000019862 | Iars\_predicted | Iars\_predicted protein (Fragment). [Source:UniProtKB/TrEMBL;Acc:Q5BJR3] | 1 |
| 1388555\_at |  | 287474 | ENSRNOG00000014072 | ENSRNOT00000019574 | NP\_001099275.1 | thioredoxin-like 5 [Source:RefSeq\_peptide;Acc:NP\_001099275] | 1 |
| 1367729\_at | P04182 | 64313 | ENSRNOG00000016807 | ENSRNOT00000022628 | Oat | Ornithine aminotransferase, mitochondrial precursor (EC 2.6.1.13) (Ornithine--oxo-acid aminotransferase). [Source:UniProtKB/Swiss-Prot;Acc:P04182] | 1 |
| 1372201\_at |  | 360584 | ENSRNOG00000027860 | ENSRNOT00000029883 | Zfp403 | Gametogenetin-binding protein 2 (Protein ZNF403). [Source:UniProtKB/Swiss-Prot;Acc:Q6GVH5] | 1 |
| 1375848\_at | Q6AXM8 | 296851 | ENSRNOG00000009112 | ENSRNOT00000036460 | Pon2 | Serum paraoxonase/arylesterase 2 (EC 3.1.1.2) (EC 3.1.8.1) (PON 2) (Serum aryldialkylphosphatase 2) (A-esterase 2) (Aromatic esterase 2). [Source:UniProtKB/Swiss-Prot;Acc:Q6AXM8] | 1 |
| 1370706\_a\_at |  |  | ENSRNOG00000031004 | ENSRNOT00000013087 | Cyp2j3 | Cytochrome P450 2J3 (EC 1.14.14.1) (CYPIIJ3). [Source:UniProtKB/Swiss-Prot;Acc:P51590] | 1 |
| 1384040\_at |  |  | ENSRNOG00000006822 | ENSRNOT00000038631 | RGD1311640\_predicted |  | 2 |
| 1372829\_at |  | 295284 | ENSRNOG00000021215 | ENSRNOT00000028807 | Rbm8\_predicted | RNA-binding motif protein 8 (Fragment). [Source:UniProtKB/TrEMBL;Acc:Q27W01] | 2 |
| 1372764\_at |  |  | ENSRNOG00000001106 | ENSRNOT00000001467 | Denr | density-regulated protein Gene [Source:MGI Symbol;Acc:MGI:1915434] | 2 |
| 1383336\_at |  | 368070 | ENSRNOG00000004061 | ENSRNOT00000005439 | NP\_001102493.1 | similar to Pinin (LOC368070), mRNA [Source:RefSeq\_dna;Acc:NM\_001109023] | 2 |
| 1389454\_at |  | 292814 | ENSRNOG00000013250 | ENSRNOT00000017708 | NP\_001099717.1 | programmed cell death 5 [Source:RefSeq\_peptide;Acc:NP\_001099717] | 2 |
| 1371832\_at | Q641X2 | 300837 | ENSRNOG00000010116 | ENSRNOT00000013512 | Leo1 | RNA polymerase-associated protein LEO1. [Source:UniProtKB/Swiss-Prot;Acc:Q641X2] | 2 |
| 1375450\_at |  |  | ENSRNOG00000004716 | ENSRNOT00000041995 | Sdccag1 | serologically defined colon cancer antigen 1 Gene [Source:MGI Symbol;Acc:MGI:1918305] | 2 |
| 1367979\_s\_at | Q64654 | 25427 | ENSRNOG00000007234 | ENSRNOT00000009985 | Cyp51 | Cytochrome P450 51A1 (EC 1.14.13.70) (CYPLI) (P450LI) (Sterol 14-alpha demethylase) (Lanosterol 14-alpha demethylase) (LDM) (P450-14DM). [Source:UniProtKB/Swiss-Prot;Acc:Q64654] | 2 |
| 1389446\_at |  |  | ENSRNOG00000011932 | ENSRNOT00000016758 | Snrpa1 | small nuclear ribonucleoprotein polypeptide A' Gene [Source:MGI (curated);Acc:Snrpa1-001] | 2 |
| 1372793\_at | P28042 | 54304 | ENSRNOG00000012100 | ENSRNOT00000016217 | Ssbp1 | Single-stranded DNA-binding protein, mitochondrial precursor (Mt-SSB) (MtSSB) (P16). [Source:UniProtKB/Swiss-Prot;Acc:P28042] | 2 |
| 1373806\_at | Q4V8E4 | 289859 | ENSRNOG00000003901 | ENSRNOT00000005306 | RGD1311732 | Coiled-coil domain-containing protein 104. [Source:UniProtKB/Swiss-Prot;Acc:Q4V8E4] | 2 |
| 1385291\_a\_at |  |  | ENSRNOG00000015787 | ENSRNOT00000021277 | Sc65 | Synaptonemal complex protein SC65. [Source:UniProtKB/Swiss-Prot;Acc:Q64375] | 2 |
| 1368034\_at | O35314 | 24259 | ENSRNOG00000021269 | ENSRNOT00000028892  ENSRNOT00000055699 | Chgb | Secretogranin-1 precursor (Secretogranin-I) (SgI) (Chromogranin-B) (CgB) (Glucagonoma peptide) [Contains: GAWK peptide; CCB peptide]. [Source:UniProtKB/Swiss-Prot;Acc:O35314] | 2 |
| 1372207\_at |  |  | ENSRNOG00000020340 | ENSRNOT00000027582  ENSRNOT00000060835 | Brd8 | bromodomain containing 8 [Source:RefSeq\_peptide;Acc:NP\_001008509] | 2 |
| 1378638\_a\_at |  | 290706 | ENSRNOG00000011293 | ENSRNOT00000015130 | RGD1311747\_predicted |  | 2 |
| 1372627\_at |  | 290408 | ENSRNOG00000012594 | ENSRNOT00000017086  ENSRNOT00000042606 | Sugt1 | Aa1114. [Source:UniProtKB/TrEMBL;Acc:Q7TQ12] | 2 |
| 1379909\_at | Q5XIG5 | 361202 | ENSRNOG00000019272 | ENSRNOT00000026070 | Gkap1 | G kinase-anchoring protein 1. [Source:UniProtKB/Swiss-Prot;Acc:Q5XIG5] | 2 |
| 1395779\_at |  | 305605 | ENSRNOG00000004057 | ENSRNOT00000005388 | RGD1306694\_predicted |  | 2 |
| 1388965\_at |  | 299147 | ENSRNOG00000005045 | ENSRNOT00000006755 | NP\_001100210.1 | protein phosphatase 2, regulatory subunit B (B56), epsilon isoform [Source:RefSeq\_peptide;Acc:NP\_001100210] | 2 |
| 1371512\_at |  |  | ENSRNOG00000025388 | ENSRNOT00000061133  ENSRNOT00000034712 | Mrpl33 | mitochondrial ribosomal protein L33 Gene [Source:MGI (curated);Acc:Mrpl33-001] | 2 |
| 1373080\_at |  | 314417 | ENSRNOG00000004827 | ENSRNOT00000006982  ENSRNOT00000047832  ENSRNOT00000057215 | NP\_001101526.1 | poly (A) polymerase alpha [Source:RefSeq\_peptide;Acc:NP\_001101526] | 2 |
| 1371730\_at | Q3MIB4 |  | ENSRNOG00000015162 | ENSRNOT00000020770 | RGD1305466 | Peroxisomal Lon protease homolog 2 (EC 3.4.21.-) (Lon protease 2) (Lon protease-like protein 2). [Source:UniProtKB/Swiss-Prot;Acc:Q3MIB4] | 2 |
| 1374323\_at |  |  | ENSRNOG00000018066 | ENSRNOT00000024355 | Bccip | BRCA2 and CDKN1A interacting protein Gene [Source:MGI (curated);Acc:Bccip-001] | 2 |
| 1368229\_at | Q9QZP1 | 84404 | ENSRNOG00000004360 | ENSRNOT00000006137 | Sip1 | Survival of motor neuron protein-interacting protein 1 (SMN- interacting protein 1) (Component of gems 2) (Gemin-2). [Source:UniProtKB/Swiss-Prot;Acc:Q9QZP1] | 2 |
| 1373899\_at |  | 296190 | ENSRNOG00000004784 | ENSRNOT00000006412 | RGD1309829\_predicted |  | 2 |
| 1373135\_at | Q5XI97 | 619440 | ENSRNOG00000020658 | ENSRNOT00000028047 | LOC619440 | Alanyl-tRNA synthetase domain-containing protein 1. [Source:UniProtKB/Swiss-Prot;Acc:Q5XI97] | 2 |
| 1374804\_at |  | 362593 | ENSRNOG00000009430 | ENSRNOT00000012702 | Gnl2 | guanine nucleotide binding protein-like 2 (nucleolar) [Source:RefSeq\_peptide;Acc:NP\_001020907] | 2 |
| 1377576\_at |  | 310856 | ENSRNOG00000012091 | ENSRNOT00000016195 | Ppa2\_predicted |  | 2 |
| 1384323\_at |  | 289990 | ENSRNOG00000007203 | ENSRNOT00000009649 | Psmc6 | Psmc6 protein (Fragment). [Source:UniProtKB/TrEMBL;Acc:Q32PW9] | 2 |
| 1374605\_at |  | 309953 | ENSRNOG00000032414 | ENSRNOT00000045851 | RGD1309660\_predicted |  | 2 |
| 1369043\_at | P15385 | 25469 | ENSRNOG00000004918 | ENSRNOT00000006524 | Kcna4 | Potassium voltage-gated channel subfamily A member 4 (Voltage-gated potassium channel subunit Kv1.4) (RCK4) (RHK1) (RK3). [Source:UniProtKB/Swiss-Prot;Acc:P15385] | 2 |
| 1377659\_at |  | 310483 | ENSRNOG00000012827 | ENSRNOT00000017151  ENSRNOT00000017143 | NP\_001101150.1 | myeloid leukemia factor 1 [Source:RefSeq\_peptide;Acc:NP\_001101150] | 2 |
| 1370934\_at | P49791 | 25281 | ENSRNOG00000001456 | ENSRNOT00000001979 | Nup153 | Nuclear pore complex protein Nup153 (Nucleoporin Nup153) (153 kDa nucleoporin). [Source:UniProtKB/Swiss-Prot;Acc:P49791] | 2 |
| 1398367\_at |  | 287643 | ENSRNOG00000004686 | ENSRNOT00000006230 | Spop | Spop protein (Fragment). [Source:UniProtKB/TrEMBL;Acc:Q5BJL3] | 2 |
| 1387872\_at | P04256 | 29578 | ENSRNOG00000036839 | ENSRNOT00000055285  ENSRNOT00000055283 | Hnrpa1 | Heterogeneous nuclear ribonucleoprotein A1 (Helix-destabilizing protein) (Single-strand RNA-binding protein) (hnRNP core protein A1) (HDP). [Source:UniProtKB/Swiss-Prot;Acc:P04256] | 2 |
| 1367596\_at | P62856 | 27139  689919  682354  303862  691434  682348  683863  688948 | ENSRNOG00000005517  ENSRNOG00000040080  ENSRNOG00000029512  ENSRNOG00000031450  ENSRNOG00000037921  ENSRNOG00000031958 | ENSRNOT00000007304  ENSRNOT00000061643  ENSRNOT00000051134  ENSRNOT00000042292  ENSRNOT00000057635  ENSRNOT00000041748 | RS26\_RAT  LOC689919    RGD1562415\_predicted  LOC688948 | 40S ribosomal protein S26. [Source:UniProtKB/Swiss-Prot;Acc:P62856]    ribosomal protein S26 (Rps26), mRNA [Source:RefSeq\_dna;Acc:NM\_013224] | 2 |
| 1369085\_s\_at | Q9WU11 | 688682  683118  113938 | ENSRNOG00000018685  ENSRNOG00000022595 | ENSRNOT00000025224  ENSRNOT00000034599 | LOC688682  Snurf | Rat snRNP-associated polypeptide N, complete cds. [Source:UniProtKB/TrEMBL;Acc:Q63747]  SNRPN upstream reading frame protein. [Source:UniProtKB/Swiss-Prot;Acc:Q9WU11] | 2 |
| 1389288\_at |  | 291660 | ENSRNOG00000017571 | ENSRNOT00000023811 | NP\_001099623.1 | NADH dehydrogenase (ubiquinone) 1 alpha subcomplex, 2 [Source:RefSeq\_peptide;Acc:NP\_001099623] | 2 |
| 1388613\_at | Q80W96 | 685052  681980  290985 | ENSRNOG00000018343 | ENSRNOT00000024781 | Hbld2 | Iron-sulfur cluster assembly 1 homolog, mitochondrial precursor (HESB- like domain-containing protein 2) (Iron sulfur assembly protein IscA). [Source:UniProtKB/Swiss-Prot;Acc:Q80W96] | 2 |
| 1377263\_at |  |  | ENSRNOG00000007053 | ENSRNOT00000009238 | Med7 | mediator complex subunit 7 Gene [Source:MGI (curated);Acc:Med7-004] | 2 |
| 1382179\_at |  | 500629 | ENSRNOG00000039551 | ENSRNOT00000006020 | NP\_001102746.1 | similar to alcohol dehydrogenase PAN2 (predicted) (RGD1565196\_predicted), mRNA [Source:RefSeq\_dna;Acc:NM\_001109276] | 2 |
| 1374423\_at |  | 100134827 | ENSRNOG00000015502 | ENSRNOT00000020907 | NP\_001099937.1 | hippocampus abundant gene transcript 1 [Source:RefSeq\_peptide;Acc:NP\_001099937] | 2 |
| 1379538\_at |  |  | ENSRNOG00000022597 | ENSRNOT00000035152 | Cenpj | centromere protein J Gene [Source:MGI (curated);Acc:Cenpj-001] | 2 |
| 1389728\_at |  |  | ENSRNOG00000004730 | ENSRNOT00000006696  ENSRNOT00000041095 | NP\_001101228.1 | Meis1, myeloid ecotropic viral integration site 1 homolog 2 [Source:RefSeq\_peptide;Acc:NP\_001101228] | 2 |
| 1371967\_at | Q5M818 | 293754 | ENSRNOG00000021005 | ENSRNOT00000028517 | Mrpl16 | 39S ribosomal protein L16, mitochondrial precursor (L16mt) (MRP-L16). [Source:UniProtKB/Swiss-Prot;Acc:Q5M818] | 2 |
| 1393779\_x\_at |  |  | ENSRNOG00000015787 | ENSRNOT00000021277 | Sc65 | Synaptonemal complex protein SC65. [Source:UniProtKB/Swiss-Prot;Acc:Q64375] | 2 |
| 1378194\_a\_at | Q5FVJ0 | 360921 | ENSRNOG00000003428 | ENSRNOT00000060349  ENSRNOT00000020608  ENSRNOT00000004776 | RUFY3\_RAT | Protein RUFY3 (Rap2-interacting protein x) (RIPx) (Single axon- regulated protein) (Singar). [Source:UniProtKB/Swiss-Prot;Acc:Q5FVJ0] | 2 |
| 1367893\_a\_at | Q9WU61 | 170927 | ENSRNOG00000020360 | ENSRNOT00000051701  ENSRNOT00000055859 | Clcc1 | Chloride channel CLIC-like protein 1 precursor (Mid-1-related chloride channel protein 1). [Source:UniProtKB/Swiss-Prot;Acc:Q9WU61] | 2 |
| 1388397\_at |  | 114021 | ENSRNOG00000007087 | ENSRNOT00000009314 | Ebna1bp2 | EBNA1 binding protein 2 [Source:RefSeq\_peptide;Acc:NP\_001008721] | 2 |
| 1377644\_at |  | 291925 | ENSRNOG00000017090 | ENSRNOT00000023169 | RGD1308706\_predicted |  | 2 |
| 1386982\_at | Q09326 | 94273 | ENSRNOG00000004234 | ENSRNOT00000005608 | Mgat2 | Alpha-1,6-mannosyl-glycoprotein 2-beta-N-acetylglucosaminyltransferase (EC 2.4.1.143) (Mannoside acetylglucosaminyltransferase 2) (N- glycosyl-oligosaccharide-glycoprotein N-acetylglucosaminyltransferase II) (Beta-1,2-N-acetylglucosaminyltransferase II) ( [Source:UniProtKB/Swiss-Prot;Acc:Q09326] | 2 |
| 1373004\_at | Q7TQ20 | 116456 | ENSRNOG00000012392 | ENSRNOT00000016909 | Dnajc2 | DnaJ homolog subfamily C member 2 (Zuotin-related factor 1) (Gliosarcoma-related antigen MIDA1). [Source:UniProtKB/Swiss-Prot;Acc:Q7TQ20] | 2 |
| 1383181\_at |  | 364240 | ENSRNOG00000006619 | ENSRNOT00000009009 | NP\_001102335.1 | DnaJ (Hsp40) homolog, subfamily C, member 9 [Source:RefSeq\_peptide;Acc:NP\_001102335] | 2 |
| 1388737\_at |  |  | ENSRNOG00000028227 | ENSRNOT00000017492 | Pb1 | polybromo 1 Gene [Source:MGI (curated);Acc:Pb1-001] | 2 |
| 1384472\_at |  | 362683 | ENSRNOG00000025926 | ENSRNOT00000036037  ENSRNOT00000035905 | LOC362683 | LOC362683 protein (Fragment). [Source:UniProtKB/TrEMBL;Acc:Q6AXS6] | 2 |
| 1394459\_at |  | 317423 | ENSRNOG00000002654 | ENSRNOT00000003565 | RGD1564253\_predicted |  | 2 |
| 1398757\_at | P13084 | 300303  25498 | ENSRNOG00000008352  ENSRNOG00000008250  ENSRNOG00000039997  ENSRNOG00000039307  ENSRNOG00000039285  ENSRNOG00000004616  ENSRNOG00000023726  ENSRNOG00000018923 | ENSRNOT00000011063  ENSRNOT00000061529  ENSRNOT00000061482  ENSRNOT00000048926  ENSRNOT00000039902  ENSRNOT00000010899  ENSRNOT00000006591  ENSRNOT00000059926  ENSRNOT00000034260  ENSRNOT00000025575 | Npm1 | Nucleophosmin (NPM) (Nucleolar phosphoprotein B23) (Numatrin) (Nucleolar protein NO38). [Source:UniProtKB/Swiss-Prot;Acc:P13084] | 2 |
| 1376202\_at |  | 293667 | ENSRNOG00000020110 | ENSRNOT00000027233 | NP\_001099794.1 | UDP-GlcNAc:betaGal beta-1,3-N-acetylglucosaminyltransferase 6 [Source:RefSeq\_peptide;Acc:NP\_001099794] | 2 |
| 1388975\_at |  | 361071 | ENSRNOG00000017481 | ENSRNOT00000023516 | Sucla2\_predicted | succinate-Coenzyme A ligase, ADP-forming, beta subunit [Source:RefSeq\_peptide;Acc:NP\_001101857] | 2 |
| 1387262\_at | P38656 | 81783  680385  679080 | ENSRNOG00000007998  ENSRNOG00000011606 | ENSRNOT00000011174  ENSRNOT00000010625  ENSRNOT00000015427 | Ssb  LOC680385 | Lupus La protein homolog (La ribonucleoprotein) (La autoantigen homolog). [Source:UniProtKB/Swiss-Prot;Acc:P38656] | 2 |
| 1370073\_at | Q9R0T3 | 63880 | ENSRNOG00000010352 | ENSRNOT00000014182 | Dnajc3 | DnaJ homolog subfamily C member 3 (Interferon-induced, double-stranded RNA-activated protein kinase inhibitor) (Protein kinase inhibitor p58) (Protein kinase inhibitor of 58 kDa). [Source:UniProtKB/Swiss-Prot;Acc:Q9R0T3] | 2 |
| 1373052\_at | Q4KLJ8 | 316348 | ENSRNOG00000013286 | ENSRNOT00000017882 | Pdcl3 | Phosducin-like protein 3. [Source:UniProtKB/Swiss-Prot;Acc:Q4KLJ8] | 2 |
| 1393086\_at |  | 305268 | ENSRNOG00000021931 | ENSRNOT00000037509 | NP\_001100683.1 | similar to RIKEN cDNA 5730469D23 (predicted) (RGD1308324\_predicted), mRNA [Source:RefSeq\_dna;Acc:NM\_001107213] | 2 |
| 1389126\_at |  | 361005 | ENSRNOG00000009297 | ENSRNOT00000012372 | NP\_001101839.1 | coiled-coil-helix-coiled-coil-helix domain containing 1 [Source:RefSeq\_peptide;Acc:NP\_001101839] | 2 |
| 1383283\_at |  | 317576 | ENSRNOG00000003875 | ENSRNOT00000005267 | NP\_001101726.1 | oculocerebrorenal syndrome of Lowe [Source:RefSeq\_peptide;Acc:NP\_001101726] | 2 |
| 1373764\_at |  |  | ENSRNOG00000014501 | ENSRNOT00000019617  ENSRNOT00000057590  ENSRNOT00000057589  ENSRNOT00000045467  ENSRNOT00000057588 | Zfml | zinc finger, matrin-like Gene [Source:MGI Symbol;Acc:MGI:1203484] | 2 |
| 1392045\_at | Q5M7A3 | 315957 | ENSRNOG00000015370 | ENSRNOT00000020610 | Tmem22 | Transmembrane protein 22. [Source:UniProtKB/Swiss-Prot;Acc:Q5M7A3] | 2 |
| 1399094\_at |  | 299154 | ENSRNOG00000007336 | ENSRNOT00000009621  ENSRNOT00000058592 | NP\_001100211.1 | churchill domain containing 1 [Source:RefSeq\_peptide;Acc:NP\_001100211] | 2 |
| 1398871\_at | P24049 | 365613  302479  679399  291434  292539  690772  691392  687168  689891  312703  360649 | ENSRNOG00000030605  ENSRNOG00000034271  ENSRNOG00000010271  ENSRNOG00000022022  ENSRNOG00000018680  ENSRNOG00000029262  ENSRNOG00000010407  ENSRNOG00000027271 | ENSRNOT00000050309  ENSRNOT00000046252  ENSRNOT00000010282  ENSRNOT00000030173  ENSRNOT00000025217  ENSRNOT00000049695  ENSRNOT00000014028  ENSRNOT00000029782 | RGD1563903\_predicted    Rpl17  Pex5\_predicted  RGD1359290 | 60S ribosomal protein L17 (L23) (Amino acid starvation-induced protein) (ASI). [Source:UniProtKB/Swiss-Prot;Acc:P24049]  Pex5\_predicted protein (Fragment). [Source:UniProtKB/TrEMBL;Acc:Q2M2R8]  Ribosomal\_L22 domain containing protein RGD1359290 [Source:RefSeq\_peptide;Acc:NP\_001041363] | 2 |
| 1373675\_at | Q6AXW1 | 114022 | ENSRNOG00000003385 | ENSRNOT00000004558  ENSRNOT00000060062 | Glrx2 | Glutaredoxin-2, mitochondrial precursor. [Source:UniProtKB/Swiss-Prot;Acc:Q6AXW1] | 2 |
| 1383341\_at |  | 290291 | ENSRNOG00000011603 | ENSRNOT00000059446  ENSRNOT00000016162 | Cab39l | calcium binding protein 39-like [Source:RefSeq\_peptide;Acc:NP\_001011917] | 2 |
| 1393647\_at | Q5M939 | 296501 | ENSRNOG00000001524 | ENSRNOT00000002085 | Hat1 | Histone acetyltransferase type B catalytic subunit (EC 2.3.1.48). [Source:UniProtKB/Swiss-Prot;Acc:Q5M939] | 2 |
| 1392734\_at |  |  | ENSRNOG00000004067 | ENSRNOT00000044233 | Nrcam | Neuronal cell adhesion molecule precursor (Nr-CAM) (NgCAM-related cell adhesion molecule) (Ng-CAM-related) (rBravo) (Ankyrin-binding cell adhesion molecule NrCAM). [Source:UniProtKB/Swiss-Prot;Acc:P97686] | 2 |
| 1374426\_at |  | 687741  690049  362897  683232  685596 | ENSRNOG00000024967  ENSRNOG00000032204  ENSRNOG00000030679 | ENSRNOT00000037030  ENSRNOT00000057660  ENSRNOT00000046035  ENSRNOT00000048584 | LOC690049 |  | 2 |
| 1371295\_at | P60868 | 500559  122772  500817  500451 | ENSRNOG00000030345  ENSRNOG00000008555  ENSRNOG00000030206  ENSRNOG00000029627  ENSRNOG00000031205  ENSRNOG00000029341  ENSRNOG00000033541  ENSRNOG00000030596 | ENSRNOT00000051449  ENSRNOT00000011314  ENSRNOT00000048346  ENSRNOT00000041813  ENSRNOT00000047587  ENSRNOT00000042264  ENSRNOT00000045982  ENSRNOT00000040481 | Rps20    RGD1562259\_predicted | 40S ribosomal protein S20. [Source:UniProtKB/Swiss-Prot;Acc:P60868] | 2 |
| 1367597\_at | P62243 | 297756  291353  65136 | ENSRNOG00000007238  ENSRNOG00000001318  ENSRNOG00000005017  ENSRNOG00000018768 | ENSRNOT00000009490  ENSRNOT00000001785  ENSRNOT00000006662  ENSRNOT00000025796 | LOC297756  RGD1562569\_predicted  RGD1566369\_predicted  Rps8 | ribosomal protein S8-like [Source:RefSeq\_peptide;Acc:NP\_001013950]    40S ribosomal protein S8. [Source:UniProtKB/Swiss-Prot;Acc:P62243] | 2 |
| 1369984\_at |  | 89786 | ENSRNOG00000038951 | ENSRNOT00000059541 | Cox17 | COX17 homolog, cytochrome c oxidase assembly protein [Source:RefSeq\_peptide;Acc:NP\_445992] | 2 |
| 1374519\_at |  |  | ENSRNOG00000008298 | ENSRNOT00000011405  ENSRNOT00000057906 | Q45N69\_RAT | Dedicator of cytokinesis 7 (Fragment). [Source:UniProtKB/TrEMBL;Acc:Q45N69] | 2 |
| 1392669\_at |  |  | ENSRNOG00000009836 | ENSRNOT00000013297 | Rbm26 | RNA binding motif protein 26 Gene [Source:MGI Symbol;Acc:MGI:1921463] | 2 |
| 1376690\_at |  | 312849 | ENSRNOG00000001820 | ENSRNOT00000002500 | NP\_001101365.1 | SRB7 [Source:RefSeq\_peptide;Acc:NP\_001101365] | 2 |
| 1377664\_at |  |  | ENSRNOG00000004127 | ENSRNOT00000005467 | Q91Y79\_RAT | KARP-1-binding protein 1. [Source:UniProtKB/TrEMBL;Acc:Q91Y79] | 2 |
| 1381174\_at | P60924 | 307480 | ENSRNOG00000019276 | ENSRNOT00000026273 | RGD735029 | Uncharacterized protein KIAA0141 homolog. [Source:UniProtKB/Swiss-Prot;Acc:P60924] | 2 |
| 1391130\_at |  | 308318 | ENSRNOG00000015598 | ENSRNOT00000020897 | RGD1308782 | Similar to Zinc finger protein OZF (POZF-1). [Source:UniProtKB/TrEMBL;Acc:Q4V8J3] | 2 |
| 1376072\_at | Q5FVQ7 | 301634 | ENSRNOG00000019087 | ENSRNOT00000050494 | RGD1309973 | Transmembrane protein 157 precursor. [Source:UniProtKB/Swiss-Prot;Acc:Q5FVQ7] | 2 |
| 1392983\_at |  | 287772 | ENSRNOG00000003117 | ENSRNOT00000004283 | Psmd12 | proteasome 26S non-ATPase subunit 12 [Source:RefSeq\_peptide;Acc:NP\_001005875] | 2 |
| 1388882\_at |  | 299104 | ENSRNOG00000004629 | ENSRNOT00000006531 | NP\_001100206.1 | FK506 binding protein 3 [Source:RefSeq\_peptide;Acc:NP\_001100206] | 2 |
| 1393034\_at |  | 293112 | ENSRNOG00000018987 | ENSRNOT00000025653 | RGD1565584\_predicted |  | 2 |
| 1373177\_x\_at |  | 691744  363336  501362  690250  689326  690429  689664  686018  501306  690024  690943  501361  685760  689678  688964  296780  501477  685786  690395  689957  683992  501386  680758  689418  501377  690048  680166  685735  501329  501220  690782  287921  689827  691762  498323  501469  501384  690659  679745  501483  689215  690832  302199  691406  691395  690855  691595  689410  690974  501421  501488  689846  689001  685044  679792  690061  690577  681391  686053  685805  691773  689497  316919  688909  301977  689762  688644  501383  501234  302278  363313  501334  689878  690631  501342  685698  690828  684652  689797  501372  685779  689681  689094  501302  680812  689501  685285  688572  689084  501315  689347  685344  691793  688946  680453  501482  363363  689545  363354  691692  688175  363337  684824  690929  501296  363424  679726  691586  681322  684084  685300  689666  685424  689329  685609  685488  681405  689241  685053  681346  683963  367390  679608  685435  684492  689058  685369  687310  689091  686041  683944  312014  363351 | ENSRNOG00000026588  ENSRNOG00000039950  ENSRNOG00000032397  ENSRNOG00000029942  ENSRNOG00000029196  ENSRNOG00000034204  ENSRNOG00000039509 | ENSRNOT00000061383  ENSRNOT00000041051  ENSRNOT00000044993  ENSRNOT00000061382  ENSRNOT00000050943  ENSRNOT00000050521  ENSRNOT00000061379  ENSRNOT00000061378  ENSRNOT00000061377  ENSRNOT00000046396  ENSRNOT00000040473  ENSRNOT00000041121  ENSRNOT00000040545  ENSRNOT00000050888  ENSRNOT00000051707  ENSRNOT00000047525  ENSRNOT00000042030 | LOC680812    LOC679726  LOC684084  LOC685424 | similar to RIKEN cDNA 1700081O22 (LOC363337), mRNA [Source:RefSeq\_dna;Acc:NM\_001014221] | 2 |
| 1383324\_at |  | 362359 | ENSRNOG00000021579 | ENSRNOT00000029332  ENSRNOT00000059060  ENSRNOT00000059059  ENSRNOT00000029354 | Mpp6\_predicted |  | 2 |
| 1391163\_at |  | 308789 | ENSRNOG00000032723 | ENSRNOT00000047408 | NP\_001101004.1 | elongation factor Tu GTP binding domain containing 1 [Source:RefSeq\_peptide;Acc:NP\_001101004] | 2 |
| 1390382\_at |  | 311359 | ENSRNOG00000015703 | ENSRNOT00000021091 | RGD1311457\_predicted |  | 2 |
| 1390514\_at | Q66H65 | 288416 | ENSRNOG00000001108 | ENSRNOT00000001468 | MGC94223 | Phosphonoformate immuno-associated protein 5 homolog. [Source:UniProtKB/Swiss-Prot;Acc:Q66H65] | 2 |
| 1367832\_at | P70470 | 25514 | ENSRNOG00000008320 | ENSRNOT00000011312 | Lypla1 | Acyl-protein thioesterase 1 (EC 3.1.2.-) (Lysophospholipase 1) (Lysophospholipase I). [Source:UniProtKB/Swiss-Prot;Acc:P70470] | 2 |
| 1387271\_at | P57093 | 114209 | ENSRNOG00000018044 | ENSRNOT00000024362 | Phyh | Phytanoyl-CoA dioxygenase, peroxisomal precursor (EC 1.14.11.18) (Phytanoyl-CoA alpha-hydroxylase) (PhyH) (Phytanic acid oxidase). [Source:UniProtKB/Swiss-Prot;Acc:P57093] | 2 |
| 1393583\_at |  | 316820  498420 | ENSRNOG00000006185 | ENSRNOT00000008135 | LOC316820 | similar to RIKEN cDNA 5730466H23 (predicted) (RGD1560957\_predicted), mRNA [Source:RefSeq\_dna;Acc:NM\_001109094] | 2 |
| 1377846\_a\_at |  |  | ENSRNOG00000016134 | ENSRNOT00000021923 | Msh6 | mutS homolog 6 (E. coli) Gene [Source:MGI Symbol;Acc:MGI:1343961] | 2 |
| 1370638\_at |  | 361833 | ENSRNOG00000024011 | ENSRNOT00000031134  ENSRNOT00000045622 | Ank3 | ankyrin 3, epithelial isoform 1 [Source:RefSeq\_peptide;Acc:NP\_113993] | 2 |
| 1370238\_at | Q9JJW3 | 171069 | ENSRNOG00000020473 | ENSRNOT00000027754 | Usmg5 | Up-regulated during skeletal muscle growth protein 5 (Diabetes- associated protein in insulin-sensitive tissues). [Source:UniProtKB/Swiss-Prot;Acc:Q9JJW3] | 2 |
| 1371344\_at | P18445 | 315642  499321  682417  500123  363817  499133  689935  688118  679196  293418 | ENSRNOG00000031736  ENSRNOG00000033274  ENSRNOG00000031273  ENSRNOG00000031859  ENSRNOG00000021055  ENSRNOG00000018338  ENSRNOG00000014214 | ENSRNOT00000041555  ENSRNOT00000041621  ENSRNOT00000041326  ENSRNOT00000045301  ENSRNOT00000028590  ENSRNOT00000013741  ENSRNOT00000055350  ENSRNOT00000019247 | RGD1565415\_predicted  RGD1559972\_predicted  Vwa1  RL27A\_RAT | ribosomal protein L27a (predicted) (Rpl27a\_predicted), mRNA [Source:RefSeq\_dna;Acc:NM\_001106290]  von Willebrand factor A domain-containing protein 1 precursor. [Source:UniProtKB/Swiss-Prot;Acc:Q642A6]  60S ribosomal protein L27a. [Source:UniProtKB/Swiss-Prot;Acc:P18445] | 2 |
| 1370253\_at | P47198 | 81768 | ENSRNOG00000028747  ENSRNOG00000037185  ENSRNOG00000011104 | ENSRNOT00000034810  ENSRNOT00000056134  ENSRNOT00000014904 | RL22\_RAT    Rpl22 | 60S ribosomal protein L22. [Source:UniProtKB/Swiss-Prot;Acc:P47198]    ribosomal protein L22 (Rpl22), mRNA [Source:RefSeq\_dna;Acc:NM\_031104] | 2 |
| 1389729\_at |  | 288652 | ENSRNOG00000001061 | ENSRNOT00000001403 | MGC94142 | similar to cDNA sequence BC003324 (MGC94142), mRNA [Source:RefSeq\_dna;Acc:NM\_001004205] | 2 |
| 1381989\_at |  |  | ENSRNOG00000005292 | ENSRNOT00000048536 | LOC314393 |  | 2 |
| 1375634\_at |  | 299707 | ENSRNOG00000005102 | ENSRNOT00000006903 | NP\_001100246.1 | coiled-coil domain containing 53 [Source:RefSeq\_peptide;Acc:NP\_001100246] | 2 |
| 1375938\_at |  | 363151 | ENSRNOG00000020946 | ENSRNOT00000028426 | NP\_001102253.1 | coiled-coil domain containing 12 [Source:RefSeq\_peptide;Acc:NP\_001102253] | 2 |
| 1393589\_at |  | 317196 | ENSRNOG00000005215 | ENSRNOT00000006992 | NP\_001101714.1 |  | 2 |
| 1368403\_at | O55081 | 81758 | ENSRNOG00000012153 | ENSRNOT00000017361 | Rbl2 | Retinoblastoma-like protein 2 (130 kDa retinoblastoma-associated protein) (PRB2) (P130) (RBR-2) (PPAR-alpha-interacting complex protein 128) (PRIC128). [Source:UniProtKB/Swiss-Prot;Acc:O55081] | 2 |
| 1398770\_at | P83883 | 292964  682278  680395  687717  81769  499622  363636  691991 | ENSRNOG00000031022 | ENSRNOT00000048198 | Rpl36a | 60S ribosomal protein L36a (60S ribosomal protein L44). [Source:UniProtKB/Swiss-Prot;Acc:P83883] | 2 |
| 1388851\_at | P48721 | 291671 | ENSRNOG00000019525 | ENSRNOT00000026696 | LOC500372 | Stress-70 protein, mitochondrial precursor (75 kDa glucose-regulated protein) (GRP 75) (Heat shock 70 kDa protein 9) (Peptide-binding protein 74) (PBP74) (mtHSP70) (Mortalin). [Source:UniProtKB/Swiss-Prot;Acc:P48721] | 2 |
| 1382741\_at |  | 361585 | ENSRNOG00000015734 | ENSRNOT00000021366 | Ube3a\_predicted |  | 2 |
| 1374004\_at | Q5HZA6 | 298771 | ENSRNOG00000007326 | ENSRNOT00000061826  ENSRNOT00000009660 | RGD1310143 | Prolyl endopeptidase-like (EC 3.4.21.-) (Prolylendopeptidase-like). [Source:UniProtKB/Swiss-Prot;Acc:Q5HZA6] | 2 |
| 1369966\_a\_at | P62850 | 681601  680393  503183  686418  690475  81776  366411  315035 | ENSRNOG00000010189  ENSRNOG00000033186  ENSRNOG00000009695  ENSRNOG00000031579 | ENSRNOT00000013588  ENSRNOT00000052060  ENSRNOT00000012849  ENSRNOT00000043005 | LOC681601  RGD1562937\_predicted  RGD1566241\_predicted  Rps24 | 40S ribosomal protein S24. [Source:UniProtKB/Swiss-Prot;Acc:P62850] | 2 |
| 1399158\_a\_at | P13084 | 300303  25498 | ENSRNOG00000008352  ENSRNOG00000039997  ENSRNOG00000039307  ENSRNOG00000039285  ENSRNOG00000004616  ENSRNOG00000005913  ENSRNOG00000023726  ENSRNOG00000018923 | ENSRNOT00000011063  ENSRNOT00000061482  ENSRNOT00000048926  ENSRNOT00000039902  ENSRNOT00000010899  ENSRNOT00000006591  ENSRNOT00000007811  ENSRNOT00000034260  ENSRNOT00000025575 | Npm1 | Nucleophosmin (NPM) (Nucleolar phosphoprotein B23) (Numatrin) (Nucleolar protein NO38). [Source:UniProtKB/Swiss-Prot;Acc:P13084] | 2 |
| 1392023\_at | Q5PQN7 | 366507 | ENSRNOG00000016200 | ENSRNOT00000021822 | Lzic | Protein LZIC (Leucine zipper and ICAT homologous domain-containing protein) (Leucine zipper and CTNNBIP1 domain-containing protein). [Source:UniProtKB/Swiss-Prot;Acc:Q5PQN7] | 2 |
| 1384314\_at |  | 305606 | ENSRNOG00000004161 | ENSRNOT00000005834 | Mtif2 | mitochondrial translational initiation factor 2 [Source:RefSeq\_peptide;Acc:NP\_001004254] | 2 |
| 1384246\_at |  | 307766 | ENSRNOG00000018149 | ENSRNOT00000024568 | NP\_001100889.1 | SWI/SNF related, matrix associated, actin dependent regulator of chromatin, subfamily a, member 5 [Source:RefSeq\_peptide;Acc:NP\_001100889] | 2 |
| 1398852\_at | P05765 | 81775 | ENSRNOG00000038864  ENSRNOG00000037867  ENSRNOG00000033916  ENSRNOG00000006325 | ENSRNOT00000059383  ENSRNOT00000057533  ENSRNOT00000051693  ENSRNOT00000008458 | Rps21 | 40S ribosomal protein S21. [Source:UniProtKB/Swiss-Prot;Acc:P05765] | 2 |
| 1374063\_at |  | 361814  680129  682323 | ENSRNOG00000000520 | ENSRNOT00000000627 | Sfrs3\_predicted | splicing factor, arginine/serine-rich 3 (SRp20) [Source:RefSeq\_peptide;Acc:NP\_001041372] | 2 |
| 1383422\_at |  | 362564 | ENSRNOG00000008025 | ENSRNOT00000010628 | NP\_001102142.1 |  | 2 |
| 1387300\_at | P63155 | 116481 | ENSRNOG00000040045  ENSRNOG00000010587 | ENSRNOT00000061588  ENSRNOT00000014632 | CRNL1\_RAT  Crnkl1 | Crooked neck-like protein 1 (Crooked neck homolog) (Crooked neck protein). [Source:UniProtKB/Swiss-Prot;Acc:P63155]  Crn, crooked neck-like 1 (Drosophila) (Crnkl1), mRNA [Source:RefSeq\_dna;Acc:NM\_053797] | 2 |
| 1388303\_at | P12749 | 307636  498998  687415  688981  287417  685704  686722 | ENSRNOG00000033258  ENSRNOG00000039783  ENSRNOG00000032000  ENSRNOG00000004214  ENSRNOG00000032777 | ENSRNOT00000041004  ENSRNOT00000061041  ENSRNOT00000046585  ENSRNOT00000005588  ENSRNOT00000046460 | RGD1563840\_predicted    LOC688981  RGD1565215\_predicted  LOC686722 | 60S ribosomal protein L26. [Source:UniProtKB/Swiss-Prot;Acc:P12749] | 2 |
| 1384011\_a\_at | Q68FN9 | 290946 | ENSRNOG00000027422 | ENSRNOT00000061802  ENSRNOT00000034410 | RGD1309729 | FAST kinase domain-containing protein 3. [Source:UniProtKB/Swiss-Prot;Acc:Q68FN9] | 2 |
| 1389026\_at |  |  | ENSRNOG00000009664 | ENSRNOT00000013095 | Ankrd42 | ankyrin repeat domain 42 Gene [Source:MGI (curated);Acc:Ankrd42-004] | 2 |
| 1380548\_at |  | 303968 | ENSRNOG00000001958 | ENSRNOT00000002684 | NP\_001100563.1 | intraflagellar transport 57 homolog [Source:RefSeq\_peptide;Acc:NP\_001100563] | 2 |
| 1371605\_at |  | 299739 | ENSRNOG00000007407 | ENSRNOT00000010117 | NP\_001100251.2 | NADH dehydrogenase (ubiquinone) 1 alpha subcomplex, 12 [Source:RefSeq\_peptide;Acc:NP\_001100251] | 2 |
| 1379542\_at |  | 680737  687845 | ENSRNOG00000001685  ENSRNOG00000005556 | ENSRNOT00000002290  ENSRNOT00000007351 | AC123665.9  Snrpf | small nuclear ribonucleoprotein polypeptide F Gene [Source:MGI (curated);Acc:Snrpf-001] | 2 |
| 1399025\_at |  |  | ENSRNOG00000007175 | ENSRNOT00000009438 | RGD1562337\_predicted |  | 2 |
| 1389125\_at |  | 289491 | ENSRNOG00000002070 | ENSRNOT00000039083  ENSRNOT00000002834  ENSRNOT00000060834 | NP\_001099467.1 | mitochondrial ribosomal protein L1 [Source:RefSeq\_peptide;Acc:NP\_001099467] | 2 |
| 1370190\_at |  |  | ENSRNOG00000006532 | ENSRNOT00000050223 | H3f3b | H3 histone, family 3B (H3f3b), mRNA [Source:RefSeq\_dna;Acc:NM\_053985] | 2 |
| 1370202\_at | P53817 | 24913 | ENSRNOG00000021206 | ENSRNOT00000028796  ENSRNOT00000032879 | Hrasls3 | HRAS-like suppressor 3 (H-rev 107 protein). [Source:UniProtKB/Swiss-Prot;Acc:P53817] | 2 |
| 1387064\_at | P24392 | 29534 | ENSRNOG00000008748 | ENSRNOT00000011579  ENSRNOT00000049852 | Pxmp3 | Peroxisome assembly factor 1 (PAF-1) (Peroxin-2) (Peroxisomal membrane protein 3). [Source:UniProtKB/Swiss-Prot;Acc:P24392] | 2 |
| 1396013\_at | Q5XIN6 | 305457 | ENSRNOG00000016427 | ENSRNOT00000022540 | Letm1 | Leucine zipper-EF-hand-containing transmembrane protein 1, mitochondrial precursor. [Source:UniProtKB/Swiss-Prot;Acc:Q5XIN6] | 2 |
| 1377937\_at |  | 289143 | ENSRNOG00000002569 | ENSRNOT00000003460 | NP\_001099433.1 | mitochondrial ribosomal protein S14 [Source:RefSeq\_peptide;Acc:NP\_001099433] | 2 |
| 1372581\_at | Q5RK19 | 287645 | ENSRNOG00000006500 | ENSRNOT00000008964 | Snf8 | Vacuolar-sorting protein SNF8 (ELL-associated protein of 30 kDa). [Source:UniProtKB/Swiss-Prot;Acc:Q5RK19] | 2 |
| 1369996\_at | O88828 | 83503 | ENSRNOG00000011214 | ENSRNOT00000015009 | Polr2f | DNA-directed RNA polymerases I, II, and III subunit RPABC2 (RNA polymerases I, II, and III subunit ABC2) (DNA-directed RNA polymerase II subunit F) (RPB6). [Source:UniProtKB/Swiss-Prot;Acc:O88828] | 2 |
| 1393587\_a\_at |  | 291320 | ENSRNOG00000016955 | ENSRNOT00000022986 | NP\_001099592.1 |  | 2 |
| 1379221\_at |  | 291860 | ENSRNOG00000017471 | ENSRNOT00000023988  ENSRNOT00000061227 | RGD1308847\_predicted | similar to SPla/RYanodine receptor SPRY (1J970) (predicted) (RGD1308847\_predicted), mRNA [Source:RefSeq\_dna;Acc:NM\_001100945] | 2 |
| 1379652\_at |  | 360886 | ENSRNOG00000023541 | ENSRNOT00000035875 | Ahctf1\_predicted |  | 2 |
| 1383825\_at |  | 315655 | ENSRNOG00000012237 | ENSRNOT00000034924  ENSRNOT00000016402 | Rdx | radixin [Source:RefSeq\_peptide;Acc:NP\_001005889] | 2 |
| 1372172\_at |  | 296136 | ENSRNOG00000015756 | ENSRNOT00000021198 | NP\_001099976.1 | nephronophthisis 1 (juvenile) homolog [Source:RefSeq\_peptide;Acc:NP\_001099976] | 2 |
| 1386770\_x\_at | P63161 | 171138 | ENSRNOG00000029811 | ENSRNOT00000040844 | Kcne2 | Potassium voltage-gated channel subfamily E member 2 (Minimum potassium ion channel-related peptide 1) (Potassium channel subunit beta MiRP1) (MinK-related peptide 1). [Source:UniProtKB/Swiss-Prot;Acc:P63161] | 2 |
| 1391426\_a\_at | Q7TP40 | 288165 | ENSRNOG00000028411 | ENSRNOT00000035666  ENSRNOT00000039997 | LOC288165 | PEST proteolytic signal-containing nuclear protein (PEST-containing nuclear protein) (PCNP) (Liver regeneration-related protein LRRG084). [Source:UniProtKB/Swiss-Prot;Acc:Q7TP40] | 2 |
| 1380513\_at | Q5I0E6 | 305120 | ENSRNOG00000023484 | ENSRNOT00000002815 | RGD1309034 | RNA polymerase II-associated protein 2. [Source:UniProtKB/Swiss-Prot;Acc:Q5I0E6] | 2 |
| 1373768\_at |  | 362426 | ENSRNOG00000006576 | ENSRNOT00000008741 | Bms1 | BMS1 homolog, ribosome assembly protein (yeast) Gene [Source:MGI Symbol;Acc:MGI:2446132] | 2 |
| 1388766\_at |  | 288150 | ENSRNOG00000001559 | ENSRNOT00000002134 | Mtx2 | metaxin 2 [Source:RefSeq\_peptide;Acc:NP\_001008287] | 2 |
| 1379420\_at |  | 299135 | ENSRNOG00000005589 | ENSRNOT00000007645 | RGD1565002\_predicted |  | 2 |
| 1371804\_at |  | 361052 | ENSRNOG00000012209 | ENSRNOT00000016416  ENSRNOT00000051316 | Cdadc1 | cytidine and dCMP deaminase domain containing 1 [Source:RefSeq\_peptide;Acc:NP\_001012156] | 2 |
| 1398373\_at | Q6AY39 | 310508 | ENSRNOG00000012019 | ENSRNOT00000016012 | B3galt3 | UDP-GalNAc:beta-1,3-N-acetylgalactosaminyltransferase 1 (EC 2.4.1.79) (Beta-3-GalNAc-T1) (Beta-1,3-galactosyltransferase 3) (Beta-1,3- GalTase 3) (Beta3Gal-T3) (b3Gal-T3) (Galactosylgalactosylglucosylceramide beta-D-acetyl- galactosaminyltransferase) (UDP [Source:UniProtKB/Swiss-Prot;Acc:Q6AY39] | 2 |
| 1384039\_at |  |  | ENSRNOG00000008741 | ENSRNOT00000060410 | RGD1310950\_predicted |  | 2 |
| 1371969\_at |  |  | ENSRNOG00000010233 | ENSRNOT00000041264 | Cald1 | Non-muscle caldesmon (CDM) (L-caldesmon). [Source:UniProtKB/Swiss-Prot;Acc:Q62736] | 2 |
| 1399057\_at | Q6AYU1 | 300891 | ENSRNOG00000014410 | ENSRNOT00000019365  ENSRNOT00000019471 | LOC366923 | Mortality factor 4-like protein 1 (MORF-related gene 15 protein) (Transcription factor-like protein MRG15). [Source:UniProtKB/Swiss-Prot;Acc:Q6AYU1] | 2 |
| 1383782\_at |  | 362700 | ENSRNOG00000027136 | ENSRNOT00000039375 | NP\_001102172.1 | spastin [Source:RefSeq\_peptide;Acc:NP\_001102172] | 2 |
| 1367457\_at |  | 114558 | ENSRNOG00000020513 | ENSRNOT00000027868 | Becn1 | beclin 1 [Source:RefSeq\_peptide;Acc:NP\_446191] | 2 |
| 1372744\_at |  | 295625 | ENSRNOG00000005504 | ENSRNOT00000059246 | NP\_001099952.1 | plakophilin 4 [Source:RefSeq\_peptide;Acc:NP\_001099952] | 2 |
| 1370464\_at |  | 170913 | ENSRNOG00000008012 | ENSRNOT00000011166  ENSRNOT00000061306 | Abcb1a | ATP-binding cassette, sub-family B (MDR/TAP), member 1A [Source:RefSeq\_peptide;Acc:NP\_596892] | 2 |
| 1367977\_at | P37377 | 29219 | ENSRNOG00000008656 | ENSRNOT00000039247  ENSRNOT00000042485 | Snca | Alpha-synuclein. [Source:UniProtKB/Swiss-Prot;Acc:P37377] | 2 |
| 1384564\_at |  | 499520 | ENSRNOG00000013372 | ENSRNOT00000017870 | RGD1564672\_predicted |  | 2 |
| 1395445\_at |  | 499617 | ENSRNOG00000028382 | ENSRNOT00000000101 | LOC499617 | similar to regulatory factor X-associated protein (LOC499617), mRNA [Source:RefSeq\_dna;Acc:NM\_001044263] | 2 |
| 1388449\_at |  | 363241 | ENSRNOG00000024186  ENSRNOG00000030934  ENSRNOG00000031628 | ENSRNOT00000034740  ENSRNOT00000048735  ENSRNOT00000042893  ENSRNOT00000047024 | LOC365574    Q7TPK5\_RAT | eukaryotic translation elongation factor 1 beta 2 [Source:RefSeq\_peptide;Acc:NP\_001102269]    Ac2-067. [Source:UniProtKB/TrEMBL;Acc:Q7TPK5] | 2 |
| 1388805\_at | P63331 | 24672 | ENSRNOG00000005389 | ENSRNOT00000007621 | Ppp2ca | Serine/threonine-protein phosphatase 2A catalytic subunit alpha isoform (EC 3.1.3.16) (PP2A-alpha). [Source:UniProtKB/Swiss-Prot;Acc:P63331] | 2 |
| 1395875\_at |  |  | ENSRNOG00000040297 | ENSRNOT00000062017 | RGD1306062\_predicted |  | 2 |
| 1373475\_at |  | 288065 | ENSRNOG00000031653 | ENSRNOT00000042208 | NP\_001099345.1 | coiled-coil domain containing 58 [Source:RefSeq\_peptide;Acc:NP\_001099345] | 2 |
| 1398761\_at | P09895 | 81763  501206 | ENSRNOG00000023529  ENSRNOG00000037220 | ENSRNOT00000030428  ENSRNOT00000056238 | Rpl5  RGD1564051\_predicted | 60S ribosomal protein L5. [Source:UniProtKB/Swiss-Prot;Acc:P09895] | 2 |
| 1381955\_at |  | 287346 | ENSRNOG00000002873 | ENSRNOT00000003841  ENSRNOT00000058484 | RGD1561932\_predicted |  | 2 |
| 1373885\_at |  | 300266 | ENSRNOG00000036841 | ENSRNOT00000055289 | NP\_001100267.1 | chromobox homolog 5 [Source:RefSeq\_peptide;Acc:NP\_001100267] | 2 |
| 1367768\_at | Q64361 | 59073 | ENSRNOG00000013572 | ENSRNOT00000018402 | Lxn | Latexin (Endogenous carboxypeptidase inhibitor) (ECI) (Tissue carboxypeptidase inhibitor) (TCI). [Source:UniProtKB/Swiss-Prot;Acc:Q64361] | 2 |
| 1377667\_at |  |  | ENSRNOG00000005183 | ENSRNOT00000006996 | NP\_001102428.1 | Rtf1, Paf1/RNA polymerase II complex component, homolog [Source:RefSeq\_peptide;Acc:NP\_001102428] | 2 |
| 1388391\_at |  | 363441 | ENSRNOG00000040005 | ENSRNOT00000061493 | NP\_001102283.1 | NADH dehydrogenase (ubiquinone) 1 alpha subcomplex, 1 [Source:RefSeq\_peptide;Acc:NP\_001102283] | 2 |
| 1373026\_at |  | 363028 | ENSRNOG00000029862 | ENSRNOT00000045384  ENSRNOT00000044611 | Spbc24\_predicted |  | 2 |
| 1391126\_at |  | 299739 | ENSRNOG00000007407 | ENSRNOT00000010117 | NP\_001100251.2 | NADH dehydrogenase (ubiquinone) 1 alpha subcomplex, 12 [Source:RefSeq\_peptide;Acc:NP\_001100251] | 2 |
| 1373365\_at | Q4KM73 | 298410 | ENSRNOG00000007775 | ENSRNOT00000010318 | Cmpk | UMP-CMP kinase (EC 2.7.4.14) (Cytidylate kinase) (Deoxycytidylate kinase) (Cytidine monophosphate kinase) (Uridine monophosphate/cytidine monophosphate kinase) (UMP/CMP kinase) (UMP/CMPK) (Uridine monophosphate kinase). [Source:UniProtKB/Swiss-Prot;Acc:Q4KM73] | 2 |
| 1389340\_at |  | 683524 | ENSRNOG00000018741 | ENSRNOT00000025335 | LOC683524 |  | 2 |
| 1382144\_at |  | 294963 | ENSRNOG00000011639 | ENSRNOT00000015864 | Mrpl47 | mitochondrial ribosomal protein L47 [Source:RefSeq\_peptide;Acc:NP\_001032260] | 2 |
| 1371868\_at |  | 295334 | ENSRNOG00000018783 | ENSRNOT00000025592 | NP\_001099928.1 | breast carcinoma amplified sequence 2 [Source:RefSeq\_peptide;Acc:NP\_001099928] | 2 |
| 1373440\_at |  | 690354  682893 | ENSRNOG00000024319 | ENSRNOT00000009271 | LOC690354 | Mdn1 protein (Fragment). [Source:UniProtKB/TrEMBL;Acc:Q5RJZ0] | 2 |
| 1367502\_at |  | 309140 | ENSRNOG00000013845 | ENSRNOT00000018848 | NP\_001101037.1 | mitochondrial ribosomal protein L21 [Source:RefSeq\_peptide;Acc:NP\_001101037] | 2 |
| 1388310\_at |  | 681962  680027  500402  294748  689031  691877  678808  287703  686076 | ENSRNOG00000033125  ENSRNOG00000033979  ENSRNOG00000039577  ENSRNOG00000039575  ENSRNOG00000029118  ENSRNOG00000033765 | ENSRNOT00000045349  ENSRNOT00000049520  ENSRNOT00000060664  ENSRNOT00000060661  ENSRNOT00000050501  ENSRNOT00000041033 | RGD1560994\_predicted  LOC681962 | suppressor of initiator codon mutations, related sequence 1 [Source:RefSeq\_peptide;Acc:NP\_001099307] | 2 |
| 1392864\_at |  | 299012 | ENSRNOG00000004696 | ENSRNOT00000006241  ENSRNOT00000041373 | Arhgap5 | Rho GTPase activating protein 5 [Source:RefSeq\_peptide;Acc:NP\_001041334] | 2 |
| 1378816\_a\_at |  | 365410 | ENSRNOG00000021057 | ENSRNOT00000028595 | NP\_001102397.1 | oxysterol binding protein [Source:RefSeq\_peptide;Acc:NP\_001102397] | 2 |
| 1396403\_at |  |  | ENSRNOG00000036607 | ENSRNOT00000054763 | AC120159.9 | ENSMUSG00000048936 protein Fragment [Source:UniProtKB/TrEMBL;Acc:Q05CL5] | 2 |
| 1388528\_at | P22509 | 292747 | ENSRNOG00000019229  ENSRNOG00000029138 | ENSRNOT00000026021  ENSRNOT00000035739  ENSRNOT00000052291 | Fbl | rRNA 2'-O-methyltransferase fibrillarin (EC 2.1.1.-) (Nucleolar protein 1). [Source:UniProtKB/Swiss-Prot;Acc:P22509] | 2 |
| 1398870\_at | Q62760 | 266601 | ENSRNOG00000019980 | ENSRNOT00000027088 | Tomm20 | Mitochondrial import receptor subunit TOM20 homolog (Mitochondrial 20 kDa outer membrane protein) (Outer mitochondrial membrane receptor Tom20). [Source:UniProtKB/Swiss-Prot;Acc:Q62760] | 2 |
| 1373913\_at |  | 360992 | ENSRNOG00000003600 | ENSRNOT00000004919  ENSRNOT00000056791 | Pnpt1 | Pnpt1 protein (Fragment). [Source:UniProtKB/TrEMBL;Acc:Q562B9] | 2 |
| 1375867\_at |  | 287441 | ENSRNOG00000014689 | ENSRNOT00000019687 | Zbtb4\_predicted |  | 2 |
| 1398353\_at |  | 361842 | ENSRNOG00000000557 | ENSRNOT00000000673 | Sar1a | SAR1a gene homolog 1 [Source:RefSeq\_peptide;Acc:NP\_001007740] | 2 |
| 1385292\_at |  |  | ENSRNOG00000015787 | ENSRNOT00000021277 | Sc65 | Synaptonemal complex protein SC65. [Source:UniProtKB/Swiss-Prot;Acc:Q64375] | 2 |
| 1368158\_at | Q62991 | 54350 | ENSRNOG00000031203 | ENSRNOT00000040548 | Scfd1 | Sec1 family domain-containing protein 1 (Syntaxin-binding protein 1- like 2) (Vesicle transport-related protein Ra410) (Sly1p). [Source:UniProtKB/Swiss-Prot;Acc:Q62991] | 2 |
| 1387780\_at | O35824 | 84026 | ENSRNOG00000016251 | ENSRNOT00000022573 | Dnaja2 | DnaJ homolog subfamily A member 2 (RDJ2). [Source:UniProtKB/Swiss-Prot;Acc:O35824] | 2 |
| 1387696\_a\_at | P22771 | 24397 | ENSRNOG00000003313 | ENSRNOT00000004443  ENSRNOT00000004426 | Glra2 | Glycine receptor subunit alpha-2 precursor (Glycine receptor strychnine-binding subunit). [Source:UniProtKB/Swiss-Prot;Acc:P22771] | 2 |
| 1395616\_at |  | 290270 | ENSRNOG00000032888 | ENSRNOT00000049420  ENSRNOT00000028145 | RGD1305133 | M-phase phosphoprotein, mpp8 [Source:RefSeq\_peptide;Acc:NP\_001017375] | 2 |
| 1395699\_at |  | 361293 | ENSRNOG00000023376 | ENSRNOT00000039377 | NP\_001101893.1 | RIO kinase 3 [Source:RefSeq\_peptide;Acc:NP\_001101893] | 2 |
| 1368154\_at | P19686 | 497757 | ENSRNOG00000012302 | ENSRNOT00000017190 | Gucy1a3 | Guanylate cyclase soluble subunit alpha-3 (EC 4.6.1.2) (GCS-alpha-3) (Soluble guanylate cyclase large subunit) (GCS-alpha-1). [Source:UniProtKB/Swiss-Prot;Acc:P19686] | 2 |
| 1373500\_at | Q5SGE0 | 313867 | ENSRNOG00000005877 | ENSRNOT00000008200 | Lrpprc | Leucine-rich PPR motif-containing protein, mitochondrial precursor (130 kDa leucine-rich protein) (LRP 130) (Leucine rich protein 157) (rLRP157). [Source:UniProtKB/Swiss-Prot;Acc:Q5SGE0] | 2 |
| 1384857\_at |  | 246150 | ENSRNOG00000026319 | ENSRNOT00000010118  ENSRNOT00000045617 | Akap9 | A kinase (PRKA) anchor protein (yotiao) 9 [Source:RefSeq\_peptide;Acc:NP\_001032170] | 2 |
| 1389712\_at |  | 361668 | ENSRNOG00000016102 | ENSRNOT00000021702 | NP\_001101976.1 | early B-cell factor 3 [Source:RefSeq\_peptide;Acc:NP\_001101976] | 2 |
| 1382452\_at | Q66H98 | 316384 | ENSRNOG00000025895 | ENSRNOT00000028907 | Sdpr | Serum deprivation-response protein (Phosphatidylserine-binding protein). [Source:UniProtKB/Swiss-Prot;Acc:Q66H98] | 2 |
| 1373744\_at |  | 311412 | ENSRNOG00000016965 | ENSRNOT00000023235  ENSRNOT00000055875 | NP\_001101241.1 | anaphase promoting complex subunit 1 [Source:RefSeq\_peptide;Acc:NP\_001101241] | 2 |
| 1370465\_at |  | 170913 | ENSRNOG00000008012 | ENSRNOT00000011166  ENSRNOT00000061306 | Abcb1a | ATP-binding cassette, sub-family B (MDR/TAP), member 1A [Source:RefSeq\_peptide;Acc:NP\_596892] | 2 |
| 1377338\_at |  | 294800 | ENSRNOG00000018063 | ENSRNOT00000024557 | NP\_001099889.1 | RAD1 homolog [Source:RefSeq\_peptide;Acc:NP\_001099889] | 2 |
| 1372329\_at |  |  | ENSRNOG00000024492 | ENSRNOT00000055607 | RGD1311435 | RGD1311435 protein (Fragment). [Source:UniProtKB/TrEMBL;Acc:Q5BJT8] | 2 |
| 1368419\_at |  |  | ENSRNOG00000011913 | ENSRNOT00000016914 | Cp | Ceruloplasmin precursor (EC 1.16.3.1) (Ferroxidase). [Source:UniProtKB/Swiss-Prot;Acc:P13635] | 2 |
| 1370911\_at | Q63014 | 116633 | ENSRNOG00000006559 | ENSRNOT00000008827 | Akap8 | A-kinase anchor protein 8 (A-kinase anchor protein 95 kDa) (AKAP 95). [Source:UniProtKB/Swiss-Prot;Acc:Q63014] | 2 |
| 1373632\_at | Q5EB68 | 373541 | ENSRNOG00000039848  ENSRNOG00000026813 | ENSRNOT00000061166  ENSRNOT00000031053 | Taf9  4921516M08Rik | Adenylate kinase isoenzyme 6 (EC 2.7.4.3) (ATP-AMP transphosphorylase 6). [Source:UniProtKB/Swiss-Prot;Acc:Q5EB68]  Adenylate kinase isoenzyme 6 (EC 2.7.4.3)(ATP-AMP transphosphorylase 6) [Source:UniProtKB/Swiss-Prot;Acc:Q8VCP8] | 2 |
| 1389485\_at | Q5PQN4 |  | ENSRNOG00000007286 | ENSRNOT00000048765 | LOC314859 | Nuclear protein MDM1. [Source:UniProtKB/Swiss-Prot;Acc:Q5PQN4] | 2 |
| 1389820\_at |  |  | ENSRNOG00000030237 | ENSRNOT00000051895 |  |  | 2 |
| 1378318\_at |  | 498097 | ENSRNOG00000001754 | ENSRNOT00000002392 | NP\_001102525.1 | similar to WD repeat domain 53 (predicted) (RGD1559546\_predicted), mRNA [Source:RefSeq\_dna;Acc:NM\_001109055] | 2 |
| 1386525\_at |  | 679894  683195  313308 | ENSRNOG00000007315 | ENSRNOT00000061664  ENSRNOT00000009755 | Thoc2\_predicted |  | 2 |
| 1373716\_at | Q5PQP9 | 361653 | ENSRNOG00000019935 | ENSRNOT00000027032 | Armc5 | Armadillo repeat-containing protein 5. [Source:UniProtKB/Swiss-Prot;Acc:Q5PQP9] | 2 |
| 1389117\_at | Q9WVS2 | 290028 | ENSRNOG00000009333 | ENSRNOT00000012790 | Osgep | Probable O-sialoglycoprotein endopeptidase (EC 3.4.24.57) (Pasteurella haemolytica metalloprotease homolog with glycoprotein substrates/gpc- like protein 1) (Prsmg1/Gcpl1) (Fragment). [Source:UniProtKB/Swiss-Prot;Acc:Q9WVS2] | 2 |
| 1373621\_at |  | 192357 | ENSRNOG00000020216 | ENSRNOT00000027429 | Gmpr2 | guanosine monophosphate reductase 2 [Source:RefSeq\_peptide;Acc:NP\_001013054] | 2 |
| 1370242\_at | P62268 | 124323  501058  498360 | ENSRNOG00000016580  ENSRNOG00000029461 | ENSRNOT00000022348  ENSRNOT00000046342 | RS23\_RAT | 40S ribosomal protein S23. [Source:UniProtKB/Swiss-Prot;Acc:P62268] | 2 |
| 1382643\_at | P57769 | 64088 | ENSRNOG00000009953 | ENSRNOT00000050987 | Snx16 | Sorting nexin-16. [Source:UniProtKB/Swiss-Prot;Acc:P57769] | 2 |
| 1398661\_at |  |  | ENSRNOG00000012058 | ENSRNOT00000050143 | NP\_001102408.1 | similar to Agrin (predicted) (RGD1306592\_predicted), mRNA [Source:RefSeq\_dna;Acc:NM\_001108938] | 2 |
| 1384205\_at | Q5XI55 | 361014 | ENSRNOG00000006143 | ENSRNOT00000008289 | Ngly1 | Peptide-N(4)-(N-acetyl-beta-glucosaminyl)asparagine amidase (EC 3.5.1.52) (PNGase) (Peptide:N-glycanase) (N-glycanase 1). [Source:UniProtKB/Swiss-Prot;Acc:Q5XI55] | 2 |
| 1372497\_at | Q501R9 | 303554  498369 | ENSRNOG00000020730 | ENSRNOT00000055198  ENSRNOT00000028149 | Nbr1 | Next to BRCA1 gene 1 protein (Neighbor of BRCA1 gene 1 protein). [Source:UniProtKB/Swiss-Prot;Acc:Q501R9] | 2 |
| 1388154\_at | Q62814 | 116651 | ENSRNOG00000010760 | ENSRNOT00000014361 | E2F5\_RAT | Transcription factor E2F5 (E2F-5) (Fragment). [Source:UniProtKB/Swiss-Prot;Acc:Q62814] | 2 |
| 1388531\_at | Q5XIU9 | 361940 | ENSRNOG00000014051 | ENSRNOT00000018796 | Pgrmc2 | Membrane-associated progesterone receptor component 2. [Source:UniProtKB/Swiss-Prot;Acc:Q5XIU9] | 2 |
| 1370170\_at |  | 117280 | ENSRNOG00000033790 | ENSRNOT00000044477  ENSRNOT00000046781 | Hnrpu | heterogeneous nuclear ribonucleoprotein U [Source:RefSeq\_peptide;Acc:NP\_476480] | 2 |
| 1389182\_at |  | 301419 | ENSRNOG00000010100 | ENSRNOT00000013502 | RGD1311269\_predicted |  | 2 |
| 1384553\_at | Q6QI44 | 360811 | ENSRNOG00000001350 | ENSRNOT00000048984  ENSRNOT00000001823 | RGD1305685 | TPR repeat-containing protein C12orf30 homolog (Liver regeneration- related protein LRRGT00164). [Source:UniProtKB/Swiss-Prot;Acc:Q6QI44] | 2 |
| 1383021\_at | Q6UE39 | 311039 | ENSRNOG00000005335 | ENSRNOT00000007108  ENSRNOT00000048804 | Galnt13 | Polypeptide N-acetylgalactosaminyltransferase 13 (EC 2.4.1.41) (Protein-UDP acetylgalactosaminyltransferase 13) (UDP- GalNAc:polypeptide N-acetylgalactosaminyltransferase 13) (Polypeptide GalNAc transferase 13) (GalNAc-T13) (pp-GaNTase 13). [Source:UniProtKB/Swiss-Prot;Acc:Q6UE39] | 2 |
| 1393922\_at |  | 362549 | ENSRNOG00000009782 | ENSRNOT00000013089 | RGD1561825\_predicted |  | 2 |
| 1374284\_at | Q566C5 | 362423 | ENSRNOG00000013526 | ENSRNOT00000018101 | Rassf4 | Ras association domain-containing protein 4. [Source:UniProtKB/Swiss-Prot;Acc:Q566C5] | 2 |
| 1390901\_at | Q6WRH9 | 310448 | ENSRNOG00000013917 | ENSRNOT00000018628 | Igsf10 | Immunoglobulin superfamily member 10 precursor (Calvaria mechanical force protein 608) (CMF608). [Source:UniProtKB/Swiss-Prot;Acc:Q6WRH9] | 2 |
| 1393004\_at |  |  | ENSRNOG00000000129 | ENSRNOT00000047893 | RGD1559864\_predicted |  | 2 |
| 1390876\_at | P0C644 | 311355 | ENSRNOG00000014436 | ENSRNOT00000019482 | RGD1311552\_predicted | Inositol hexakisphosphate and diphosphoinositol-pentakisphosphate kinase 1 (EC 2.7.4.21) (EC 2.7.4.24) (InsP6 and PP-IP5 kinase 1) (PP- IP5 kinase 1) (Histidine acid phosphatase domain-containing protein 2A). [Source:UniProtKB/Swiss-Prot;Acc:P0C644] | 2 |
| 1398983\_at | P0C2C1 | 502223  301352 | ENSRNOG00000018511 | ENSRNOT00000024990 | LOC502223 | 39S ribosomal protein L30, mitochondrial precursor (L30mt) (MRP-L30). [Source:UniProtKB/Swiss-Prot;Acc:P0C2C1] | 2 |
| 1382556\_a\_at |  |  | ENSRNOG00000013574 | ENSRNOT00000018413 | RGD1309388\_predicted |  | 2 |
| 1389492\_at | Q62848 | 246310 | ENSRNOG00000010326 | ENSRNOT00000055039  ENSRNOT00000041309  ENSRNOT00000040270 | Arfgap1 | ADP-ribosylation factor GTPase-activating protein 1 (ADP-ribosylation factor 1 GTPase-activating protein) (ARF1 GAP) (ARF1-directed GTPase- activating protein) (GAP protein). [Source:UniProtKB/Swiss-Prot;Acc:Q62848] | 2 |
| 1397552\_at |  | 313861 | ENSRNOG00000030294 | ENSRNOT00000061931  ENSRNOT00000048972  ENSRNOT00000042307 | NP\_001101478.1 | echinoderm microtubule associated protein like 4 [Source:RefSeq\_peptide;Acc:NP\_001101478] | 2 |
| 1389621\_at | Q5U317 | 289582 | ENSRNOG00000002275 | ENSRNOT00000003093  ENSRNOT00000003096  ENSRNOT00000045050 | Fip1l1 | Pre-mRNA 3'-end-processing factor FIP1 (FIP1-like 1). [Source:UniProtKB/Swiss-Prot;Acc:Q5U317] | 2 |
| 1377959\_at |  | 498600 | ENSRNOG00000039528 | ENSRNOT00000019556 | RGD1561287\_predicted |  | 2 |
| 1372713\_at |  |  | ENSRNOG00000006587 | ENSRNOT00000057179  ENSRNOT00000044312 | RGD1309550 | RGD1309550 protein (Fragment). [Source:UniProtKB/TrEMBL;Acc:Q5FWY6] | 2 |
| 1379481\_at |  | 116697 | ENSRNOG00000015732 | ENSRNOT00000021096 | Bcl2l2 | Bcl2-like 2 [Source:RefSeq\_peptide;Acc:NP\_068622] | 2 |
| 1373824\_at | Q75UQ2 | 313982  292027 | ENSRNOG00000019326 | ENSRNOT00000026249 | Cfdp1 | Craniofacial development protein 1 (Bucentaur). [Source:UniProtKB/Swiss-Prot;Acc:Q75UQ2] | 2 |
| 1370830\_at |  | 24329 | ENSRNOG00000004332 | ENSRNOT00000006087 | Egfr | epidermal growth factor receptor [Source:RefSeq\_peptide;Acc:NP\_113695] | 2 |
| 1385458\_a\_at |  | 361624 | ENSRNOG00000013744 | ENSRNOT00000018497 | NP\_001101967.1 |  | 2 |
| 1383554\_at |  | 315911 | ENSRNOG00000011120 | ENSRNOT00000016100 | LOC679381 |  | 2 |
| 1368992\_a\_at | Q09167 | 29667 | ENSRNOG00000005513 | ENSRNOT00000007583  ENSRNOT00000058290 | Sfrs5 | Splicing factor, arginine/serine-rich 5 (Pre-mRNA-splicing factor SRP40) (Insulin-induced growth response protein CL-4) (Delayed-early protein HRS). [Source:UniProtKB/Swiss-Prot;Acc:Q09167] | 2 |
| 1370506\_at | Q63619 | 25249 | ENSRNOG00000017012 | ENSRNOT00000022988 | Coq7 | Ubiquinone biosynthesis protein COQ7 homolog (Coenzyme Q biosynthesis protein 7 homolog) (Timing protein clk-1 homolog) (Fragment). [Source:UniProtKB/Swiss-Prot;Acc:Q63619] | 2 |
| 1370283\_at | P06761 | 25617 | ENSRNOG00000018294 | ENSRNOT00000025067 | Hspa5 | 78 kDa glucose-regulated protein precursor (GRP 78) (Heat shock 70 kDa protein 5) (Immunoglobulin heavy chain-binding protein) (BiP) (Steroidogenesis-activator polypeptide). [Source:UniProtKB/Swiss-Prot;Acc:P06761] | 2 |
| 1388669\_at |  | 313583  503168 | ENSRNOG00000007629 | ENSRNOT00000010373 | Sf3a3 | splicing factor 3a, subunit 3 [Source:RefSeq\_peptide;Acc:NP\_001020869] | 2 |
| 1376195\_at | Q4V8J7 | 361217  682571 | ENSRNOG00000031378  ENSRNOG00000011119  ENSRNOG00000034120 | ENSRNOT00000049885  ENSRNOT00000014787  ENSRNOT00000045802 | Spin | Spindlin-1. [Source:UniProtKB/Swiss-Prot;Acc:Q4V8J7] | 2 |
| 1370398\_at | Q9R0A3 | 192225 | ENSRNOG00000003955 | ENSRNOT00000005297  ENSRNOT00000057654  ENSRNOT00000057652 | Spata7 | Spermatogenesis-associated protein 7 homolog (Rat sperm DNA no.3) (RSD-3) (Fertility-related protein WMP1). [Source:UniProtKB/Swiss-Prot;Acc:Q9R0A3] | 2 |
| 1392469\_at |  | 311881 | ENSRNOG00000018687 | ENSRNOT00000025399 | NP\_001101305.1 | F-box and WD-40 domain protein 2 [Source:RefSeq\_peptide;Acc:NP\_001101305] | 2 |
| 1392320\_s\_at |  | 289437 | ENSRNOG00000002054 | ENSRNOT00000002811  ENSRNOT00000061583  ENSRNOT00000061579 | NP\_001099463.1 | glomulin, FKBP associated protein [Source:RefSeq\_peptide;Acc:NP\_001099463] | 3 |
| 1376641\_at | P59924 | 291797 | ENSRNOG00000015332  ENSRNOG00000032739 | ENSRNOT00000021087  ENSRNOT00000046718  ENSRNOT00000045976 | Thoc1  THOC1\_RAT | THO complex 1 [Source:RefSeq\_peptide;Acc:NP\_001041315]  THO complex subunit 1 (Tho1) (Nuclear matrix protein p84) (Liver regeneration-related protein LRRG175). [Source:UniProtKB/Swiss-Prot;Acc:P59924] | 3 |
| 1392629\_a\_at | Q499V6 | 362990 | ENSRNOG00000004996 | ENSRNOT00000006780 | Zcrb1 | Zinc finger CCHC-type and RNA-binding motif-containing protein 1 (U11/U12 small nuclear ribonucleoprotein 31 kDa protein) (U11/U12 snRNP 31 kDa protein). [Source:UniProtKB/Swiss-Prot;Acc:Q499V6] | 3 |
| 1374188\_at |  | 294912 | ENSRNOG00000009057 | ENSRNOT00000012240 | Tloc1 | translocation protein 1 [Source:RefSeq\_peptide;Acc:NP\_001029301] | 3 |
| 1385999\_at | Q925S8 | 114217 | ENSRNOG00000017100 | ENSRNOT00000023395 | Yme1l1 | ATP-dependent metalloprotease YME1L1 (EC 3.4.24.-) (YME1-like protein 1) (ATP-dependent metalloprotease FtsH1) (Meg-4). [Source:UniProtKB/Swiss-Prot;Acc:Q925S8] | 3 |
| 1387875\_at | O35346 | 25614 | ENSRNOG00000007916 | ENSRNOT00000011219  ENSRNOT00000046362 | Ptk2 | Focal adhesion kinase 1 (EC 2.7.10.2) (FADK 1) (pp125FAK). [Source:UniProtKB/Swiss-Prot;Acc:O35346] | 3 |
| 1370455\_a\_at | P63057 | 252920 | ENSRNOG00000017969 | ENSRNOT00000024243 | Olfm3 | Noelin-3 precursor (Olfactomedin-3) (Optimedin). [Source:UniProtKB/Swiss-Prot;Acc:P63057] | 3 |
| 1377998\_at | Q3B7D0 | 304024 | ENSRNOG00000001654 | ENSRNOT00000002257 | Cpox | Coproporphyrinogen III oxidase, mitochondrial precursor (EC 1.3.3.3) (Coproporphyrinogenase) (Coprogen oxidase) (COX). [Source:UniProtKB/Swiss-Prot;Acc:Q3B7D0] | 3 |
| 1382161\_at |  | 293828 | ENSRNOG00000016266 | ENSRNOT00000038511 | NP\_001099810.1 | M-phase phosphoprotein 10 (U3 small nucleolar ribonucleoprotein) [Source:RefSeq\_peptide;Acc:NP\_001099810] | 3 |
| 1391427\_at |  | 294718 | ENSRNOG00000013596 | ENSRNOT00000018527 | NP\_001099878.1 | similar to RIKEN cDNA 1500031M22 (predicted) (RGD1311752\_predicted), mRNA [Source:RefSeq\_dna;Acc:NM\_001106408] | 3 |
| 1385830\_at |  | 292078 | ENSRNOG00000038239 | ENSRNOT00000058216 | NP\_001099665.1 |  | 3 |
| 1392211\_at |  | 295461 | ENSRNOG00000023373 | ENSRNOT00000038509 | NP\_001099944.1 | SEC24 related gene family, member B [Source:RefSeq\_peptide;Acc:NP\_001099944] | 3 |
| 1377970\_at |  | 294762 | ENSRNOG00000010824 | ENSRNOT00000014944  ENSRNOT00000060595 | LOC294762 |  | 3 |
| 1367619\_at | P70580 |  | ENSRNOG00000012786 | ENSRNOT00000017101 | Pgrmc1 | Membrane-associated progesterone receptor component 1 (Acidic 25 kDa protein) (25-DX) (Ventral midline antigen) (VEMA). [Source:UniProtKB/Swiss-Prot;Acc:P70580] | 3 |
| 1372720\_at |  | 293060 | ENSRNOG00000019529 | ENSRNOT00000026595 | Btbd1 | BTB (POZ) domain containing 1 [Source:RefSeq\_peptide;Acc:NP\_001011932] | 3 |
| 1378022\_at |  | 313216 | ENSRNOG00000006087 | ENSRNOT00000059869 | NP\_001101399.2 | ring finger protein 20 [Source:RefSeq\_peptide;Acc:NP\_001101399] | 3 |
| 1394059\_s\_at | Q925S8 | 114217 | ENSRNOG00000017100 | ENSRNOT00000023395 | Yme1l1 | ATP-dependent metalloprotease YME1L1 (EC 3.4.24.-) (YME1-like protein 1) (ATP-dependent metalloprotease FtsH1) (Meg-4). [Source:UniProtKB/Swiss-Prot;Acc:Q925S8] | 3 |
| 1390579\_at |  | 290686 | ENSRNOG00000014188 | ENSRNOT00000019363 | RGD1305222\_predicted |  | 3 |
| 1379636\_at | Q498D5 | 313840 | ENSRNOG00000006082 | ENSRNOT00000008045 | LOC313840 | Protein FAM82A. [Source:UniProtKB/Swiss-Prot;Acc:Q498D5] | 3 |
| 1377820\_a\_at |  |  | ENSRNOG00000024414 | ENSRNOT00000054950 | NP\_001099770.1 | arginine-tRNA-protein transferase 1 [Source:RefSeq\_peptide;Acc:NP\_001099770] | 3 |
| 1377642\_at |  | 363425 | ENSRNOG00000006411 | ENSRNOT00000008722 | Cav2 | caveolin 2 [Source:RefSeq\_peptide;Acc:NP\_571989] | 3 |
| 1383089\_at | Q6AXT5 | 299799 | ENSRNOG00000003923 | ENSRNOT00000005258 | Rab21 | Ras-related protein Rab-21. [Source:UniProtKB/Swiss-Prot;Acc:Q6AXT5] | 3 |
| 1395488\_at |  | 499505 | ENSRNOG00000013673 | ENSRNOT00000045570 | Msh3 | Msh3 protein (Fragment). [Source:UniProtKB/TrEMBL;Acc:Q5BJY1] | 3 |
| 1398764\_at | P20280 | 683497  681195  366248  691444  79449  499845  684853  682478  690841  679852  682788  682211  680974  684656  294700  684216  691195  365429  683546  680639  310365  300731  296502  366689  295941  364560  366724  682872  680650  295472  679634  679310  293642 | ENSRNOG00000029410  ENSRNOG00000000957  ENSRNOG00000033461  ENSRNOG00000032803  ENSRNOG00000031717  ENSRNOG00000029384  ENSRNOG00000034137  ENSRNOG00000029395  ENSRNOG00000029498  ENSRNOG00000031062  ENSRNOG00000033115  ENSRNOG00000031735  ENSRNOG00000033779  ENSRNOG00000028867  ENSRNOG00000034135  ENSRNOG00000031560  ENSRNOG00000030594  ENSRNOG00000033855  ENSRNOG00000036643 | ENSRNOT00000046791  ENSRNOT00000001265  ENSRNOT00000041479  ENSRNOT00000047936  ENSRNOT00000048123  ENSRNOT00000049363  ENSRNOT00000047523  ENSRNOT00000048115  ENSRNOT00000048356  ENSRNOT00000049571  ENSRNOT00000051772  ENSRNOT00000041159  ENSRNOT00000044723  ENSRNOT00000051901  ENSRNOT00000040249  ENSRNOT00000048373  ENSRNOT00000050384  ENSRNOT00000042619  ENSRNOT00000054860 | LOC682478    LOC690364  RGD1565159\_predicted  AC115846.7  RGD1562469\_predicted  RGD1566326\_predicted  LOC682872 | 60S ribosomal protein L21. [Source:UniProtKB/Swiss-Prot;Acc:P20280] | 3 |
| 1391212\_at | Q5PPP3 | 302593 | ENSRNOG00000002387 | ENSRNOT00000044372 | Tceal1 | Transcription elongation factor A protein-like 1 (TCEA-like protein 1) (Transcription elongation factor S-II protein-like 1). [Source:UniProtKB/Swiss-Prot;Acc:Q5PPP3] | 3 |
| 1389294\_at |  | 308666 | ENSRNOG00000011945 | ENSRNOT00000016376 | NP\_001100987.1 | cytoplasmic FMR1 interacting protein 1 [Source:RefSeq\_peptide;Acc:NP\_001100987] | 3 |
| 1372999\_at | Q5PPL2 | 315405 | ENSRNOG00000008390 | ENSRNOT00000061916  ENSRNOT00000011186 | Dcun1d5 | DCN1-like protein 5 (Defective in cullin neddylation protein 1-like protein 5) (DCUN1 domain-containing protein 5). [Source:UniProtKB/Swiss-Prot;Acc:Q5PPL2] | 3 |
| 1379580\_at |  | 311607 | ENSRNOG00000016744 | ENSRNOT00000022861 | NP\_001101267.1 | chromodomain helicase DNA binding protein 6 [Source:RefSeq\_peptide;Acc:NP\_001101267] | 3 |
| 1390168\_a\_at |  | 362184 | ENSRNOG00000004842 | ENSRNOT00000006431 | Dph4 | DnaJ (Hsp40) homolog, subfamily C, member 24 Gene [Source:MGI (curated);Acc:Dph4-001] | 3 |
| 1373201\_at |  | 29611 | ENSRNOG00000015029 | ENSRNOT00000020267 | Dbt | dihydrolipoamide branched chain transacylase E2 [Source:RefSeq\_peptide;Acc:NP\_445764] | 3 |
| 1374518\_at | Q5BK09 | 362011 | ENSRNOG00000017744 | ENSRNOT00000023903  ENSRNOT00000023910 | Tmem77 | Transmembrane protein 77. [Source:UniProtKB/Swiss-Prot;Acc:Q5BK09] | 3 |
| 1380443\_at |  | 362856 | ENSRNOG00000005350 | ENSRNOT00000007549 | Pwp1\_predicted |  | 3 |
| 1371899\_at | Q4V8C7 | 311130 | ENSRNOG00000011195 | ENSRNOT00000015624 | Prkra | Interferon-inducible double stranded RNA-dependent protein kinase activator A (Protein kinase, interferon-inducible double stranded RNA- dependent activator) (Protein activator of the interferon-induced protein kinase). [Source:UniProtKB/Swiss-Prot;Acc:Q4V8C7] | 3 |
| 1379388\_at |  |  | ENSRNOG00000004186 | ENSRNOT00000005637 | Snx13 | sorting nexin 13 Gene [Source:MGI Symbol;Acc:MGI:2661416] | 3 |
| 1380447\_a\_at |  | 289469 | ENSRNOG00000002178 | ENSRNOT00000002962 | NP\_001099466.1 | mitochondrial ribosomal protein S18C [Source:RefSeq\_peptide;Acc:NP\_001099466] | 3 |
| 1389204\_at |  | 361269 | ENSRNOG00000018011 | ENSRNOT00000048933 | RGD1564456\_predicted |  | 3 |
| 1378741\_at | Q6AXZ3 | 362739 | ENSRNOG00000023591 | ENSRNOT00000033533 | RGD1309207 | Serine/threonine-protein phosphatase 2A regulatory subunit B'' subunit gamma. [Source:UniProtKB/Swiss-Prot;Acc:Q6AXZ3] | 3 |
| 1376687\_at | Q569C3 | 313387 | ENSRNOG00000007890 | ENSRNOT00000010933 | Usp1 | Ubiquitin carboxyl-terminal hydrolase 1 (EC 3.1.2.15) (Ubiquitin thioesterase 1) (Ubiquitin-specific-processing protease 1) (Deubiquitinating enzyme 1). [Source:UniProtKB/Swiss-Prot;Acc:Q569C3] | 3 |
| 1389980\_at |  | 289324 | ENSRNOG00000003717 | ENSRNOT00000004939 | NP\_001099451.1 | similar to Protein HSPC163 (predicted) (RGD1559740\_predicted), mRNA [Source:RefSeq\_dna;Acc:NM\_001105981] | 3 |
| 1376067\_at |  | 299805 | ENSRNOG00000004909 | ENSRNOT00000006779 | Cnot2 | CCR4-NOT transcription complex, subunit 2 [Source:RefSeq\_peptide;Acc:NP\_001011988] | 3 |
| 1377172\_at |  | 362021 | ENSRNOG00000012149 | ENSRNOT00000016250 | RGD1560967\_predicted |  | 3 |
| 1393043\_at |  | 303456 | ENSRNOG00000002644 | ENSRNOT00000003582 | Wdr50\_predicted |  | 3 |
| 1391236\_at |  | 305268 | ENSRNOG00000021931 | ENSRNOT00000037509 | NP\_001100683.1 | similar to RIKEN cDNA 5730469D23 (predicted) (RGD1308324\_predicted), mRNA [Source:RefSeq\_dna;Acc:NM\_001107213] | 3 |
| 1384734\_at |  | 288280 | ENSRNOG00000002126 | ENSRNOT00000002895 | Ncam2 | neural cell adhesion molecule 2 [Source:RefSeq\_peptide;Acc:NP\_981954] | 3 |
| 1385007\_at |  | 309986 | ENSRNOG00000014927 | ENSRNOT00000020111 | Zcchc9 | zinc finger, CCHC domain containing 9 [Source:RefSeq\_peptide;Acc:NP\_001013174] | 3 |
| 1389162\_at |  | 297416 | ENSRNOG00000018410 | ENSRNOT00000024884 | NP\_001100076.1 | histone cell cycle regulation defective interacting protein 5 [Source:RefSeq\_peptide;Acc:NP\_001100076] | 3 |
| 1373861\_at |  | 361089 | ENSRNOG00000024022 | ENSRNOT00000039423 | NP\_001101860.1 | Nedd4 family interacting protein 2 [Source:RefSeq\_peptide;Acc:NP\_001101860] | 3 |
| 1383953\_at |  |  | ENSRNOG00000022812 | ENSRNOT00000059349  ENSRNOT00000059348 | Ercc5 | excision repair cross-complementing rodent repair deficiency, complementation group 5 Gene [Source:MGI (curated);Acc:Ercc5-001] | 3 |
| 1390800\_a\_at |  | 363425 | ENSRNOG00000006411 | ENSRNOT00000008722 | Cav2 | caveolin 2 [Source:RefSeq\_peptide;Acc:NP\_571989] | 3 |
| 1382045\_at |  | 366896 | ENSRNOG00000003889 | ENSRNOT00000005207 | Tbc1d15 | Tbc1d15 protein (Fragment). [Source:UniProtKB/TrEMBL;Acc:Q4FZT5] | 3 |
| 1372776\_at |  |  | ENSRNOG00000005261 | ENSRNOT00000007016 | Fbxl5 | F-box and leucine-rich repeat protein 5 Gene [Source:MGI (curated);Acc:Fbxl5-002] | 3 |
| 1368060\_at | P52759 | 65151 | ENSRNOG00000005437 | ENSRNOT00000007430 | Hrsp12 | Ribonuclease UK114 (EC 3.1.-.-) (14.5 kDa translational inhibitor protein) (Perchloric acid soluble protein). [Source:UniProtKB/Swiss-Prot;Acc:P52759] | 3 |
| 1371856\_at | Q66HE1 | 100125373 | ENSRNOG00000009248 | ENSRNOT00000012258 | Pnrc2 | Proline-rich nuclear receptor coactivator 2. [Source:UniProtKB/Swiss-Prot;Acc:Q66HE1] | 3 |
| 1375425\_at |  | 287541 | ENSRNOG00000008891 | ENSRNOT00000011795 | NP\_001099285.1 |  | 3 |
| 1379850\_at |  | 289990 | ENSRNOG00000007203 | ENSRNOT00000009649 | Psmc6 | Psmc6 protein (Fragment). [Source:UniProtKB/TrEMBL;Acc:Q32PW9] | 3 |
| 1391415\_at | Q6VBQ8 | 289809 | ENSRNOG00000005524 | ENSRNOT00000007824 | LOC289809 | RNA-binding protein PNO1. [Source:UniProtKB/Swiss-Prot;Acc:Q6VBQ8] | 3 |
| 1383429\_at |  | 311078 | ENSRNOG00000004911 | ENSRNOT00000006605 | LOC311078 | Similar to T-Brain-1. [Source:UniProtKB/TrEMBL;Acc:Q4V8E2] | 3 |
| 1383011\_at |  | 502531 | ENSRNOG00000013393 | ENSRNOT00000018022 | NP\_001102809.1 | eukaryotic translation initiation factor 2A [Source:RefSeq\_peptide;Acc:NP\_001102809] | 3 |
| 1398789\_at | P61928 | 498744  682878  690840  679570  683675  360956  289715  81770  289384 | ENSRNOG00000012538  ENSRNOG00000032887  ENSRNOG00000031640  ENSRNOG00000033384  ENSRNOG00000006400  ENSRNOG00000033803  ENSRNOG00000031609  ENSRNOG00000031098  ENSRNOG00000034180 | ENSRNOT00000017052  ENSRNOT00000048997  ENSRNOT00000049975  ENSRNOT00000043523  ENSRNOT00000058414  ENSRNOT00000008579  ENSRNOT00000045156  ENSRNOT00000047422  ENSRNOT00000049634  ENSRNOT00000046519 | RGD1561310\_predicted  LOC690840  LOC683675  Tbc1d14  RGD1562796\_predicted  RGD1560186\_predicted  Rpl37 | TBC1 domain family, member 14 isoform 2 [Source:RefSeq\_peptide;Acc:NP\_001029193]  60S ribosomal protein L37. [Source:UniProtKB/Swiss-Prot;Acc:P61928] | 3 |
| 1368175\_at | Q8R515 | 171159 | ENSRNOG00000006412 | ENSRNOT00000008454 | Zhx1 | Zinc fingers and homeoboxes protein 1. [Source:UniProtKB/Swiss-Prot;Acc:Q8R515] | 3 |
| 1390628\_at |  | 360949 | ENSRNOG00000005043 | ENSRNOT00000006727 | NP\_001101831.1 | cytoplasmic polyadenylation element binding protein 2 [Source:RefSeq\_peptide;Acc:NP\_001101831] | 3 |
| 1379327\_at |  | 307766 | ENSRNOG00000018149 | ENSRNOT00000024568 | NP\_001100889.1 | SWI/SNF related, matrix associated, actin dependent regulator of chromatin, subfamily a, member 5 [Source:RefSeq\_peptide;Acc:NP\_001100889] | 3 |
| 1373060\_at |  |  | ENSRNOG00000040257 | ENSRNOT00000000904 | Chmp2b | chromatin modifying protein 2B Gene [Source:MGI Symbol;Acc:MGI:1916192] | 3 |
| 1369777\_a\_at | Q9QX74 | 171093 | ENSRNOG00000029931 | ENSRNOT00000040150  ENSRNOT00000041825 | Shank2 | SH3 and multiple ankyrin repeat domains protein 2 (Shank2) (Cortactin- binding protein 1) (CortBP1) (Proline-rich synapse-associated protein 1) (ProSAP1) (GKAP/SAPAP-interacting protein) (SPANK-3). [Source:UniProtKB/Swiss-Prot;Acc:Q9QX74] | 3 |
| 1398276\_at | Q63622 | 64053 | ENSRNOG00000022635 | ENSRNOT00000055403  ENSRNOT00000055402  ENSRNOT00000055401  ENSRNOT00000055400  ENSRNOT00000034416  ENSRNOT00000055399  ENSRNOT00000055397 | Dlgh2 | Disks large homolog 2 (Postsynaptic density protein PSD-93) (Channel- associated protein of synapse-110) (Chapsyn-110). [Source:UniProtKB/Swiss-Prot;Acc:Q63622] | 3 |
| 1374699\_at |  | 313969 | ENSRNOG00000004084 | ENSRNOT00000005424 | RGD1305779\_predicted |  | 3 |
| 1368045\_at | Q9JK41 | 171135 | ENSRNOG00000014475 | ENSRNOT00000019544 | Slc31a1 | High affinity copper uptake protein 1 (rCTR1) (Copper transporter 1) (Solute carrier family 31 member 1) (Liver regeneration-related protein LRRGT00200). [Source:UniProtKB/Swiss-Prot;Acc:Q9JK41] | 3 |
| 1377855\_at | Q66H89 | 366872 | ENSRNOG00000007859 | ENSRNOT00000034639 | RGD1359593 | Coiled-coil domain-containing protein 41. [Source:UniProtKB/Swiss-Prot;Acc:Q66H89] | 3 |
| 1392541\_at |  | 685702  362368 | ENSRNOG00000034084  ENSRNOG00000028843 | ENSRNOT00000043752  ENSRNOT00000045562 | LOC685702 | similar to RIKEN cDNA A030007L17; EST AA673177 (predicted) (RGD1304876\_predicted), mRNA [Source:RefSeq\_dna;Acc:NM\_001108629] | 3 |
| 1392997\_at |  |  | ENSRNOG00000024647 | ENSRNOT00000029115 | Ppp4r2 | protein phosphatase 4, regulatory subunit 2 Gene [Source:MGI Symbol;Acc:MGI:3027896] | 3 |
| 1399018\_at |  | 360602 | ENSRNOG00000002835 | ENSRNOT00000003914 | NP\_001101761.1 | similar to cisplatin resistance-associated overexpressed protein (predicted) (RGD1307981\_predicted), mRNA [Source:RefSeq\_dna;Acc:NM\_001108291] | 3 |
| 1390788\_a\_at |  | 361305  688637 | ENSRNOG00000027355 | ENSRNOT00000031530 | Wdr36\_predicted |  | 3 |
| 1391416\_at |  |  | ENSRNOG00000002940 | ENSRNOT00000035592  ENSRNOT00000004068 | Q99MD2\_RAT | Serologically defined breast cancer antigen NY-BR-16-like protein (Fragment). [Source:UniProtKB/TrEMBL;Acc:Q99MD2] | 3 |
| 1393459\_at |  |  | ENSRNOG00000011900 | ENSRNOT00000056544 | Fmr1 | fragile X mental retardation syndrome 1 homolog [Source:RefSeq\_peptide;Acc:NP\_434691] | 3 |
| 1398760\_at | P04646 | 686786  57809  685887  691650  287558 | ENSRNOG00000031694  ENSRNOG00000029454  ENSRNOG00000033625  ENSRNOG00000031411  ENSRNOG00000038678  ENSRNOG00000033058  ENSRNOG00000032348  ENSRNOG00000030217  ENSRNOG00000031641  ENSRNOG00000033399  ENSRNOG00000032303 | ENSRNOT00000041291  ENSRNOT00000050438  ENSRNOT00000043796  ENSRNOT00000045599  ENSRNOT00000059047  ENSRNOT00000043587  ENSRNOT00000043508  ENSRNOT00000049614  ENSRNOT00000042285  ENSRNOT00000050968  ENSRNOT00000040261 | RL35A\_RAT  Rpl35a | 60S ribosomal protein L35a. [Source:UniProtKB/Swiss-Prot;Acc:P04646] | 3 |
| 1371313\_at | P62752 | 308263  686564  679285  683904  688776  498523  289656  679137  680441  499523  360572 | ENSRNOG00000029255  ENSRNOG00000029670  ENSRNOG00000030614  ENSRNOG00000029859  ENSRNOG00000032148  ENSRNOG00000028963  ENSRNOG00000032771  ENSRNOG00000010132  ENSRNOG00000033439  ENSRNOG00000023344 | ENSRNOT00000045791  ENSRNOT00000048598  ENSRNOT00000040647  ENSRNOT00000049703  ENSRNOT00000043836  ENSRNOT00000051559  ENSRNOT00000051599  ENSRNOT00000050217  ENSRNOT00000013436  ENSRNOT00000051510  ENSRNOT00000035657 | CAAA01202578.1.1.17880  LOC679285  RGD1565170\_predicted  RGD1562755\_predicted  AL772308.4  LOC365203  LOC686564 | 60S ribosomal protein L23a. [Source:UniProtKB/Swiss-Prot;Acc:P62752] | 3 |
| 1383396\_at |  | 306022 | ENSRNOG00000014478 | ENSRNOT00000020135 | NP\_001100748.1 | fibronectin type III domain containing 3a [Source:RefSeq\_peptide;Acc:NP\_001100748] | 3 |
| 1388344\_at | Q498U4 | 362819 | ENSRNOG00000030520 | ENSRNOT00000009155 | RGD1305692 | Nuclear protein Hcc-1. [Source:UniProtKB/Swiss-Prot;Acc:Q498U4] | 3 |
| 1377718\_at |  | 501552 | ENSRNOG00000003346 | ENSRNOT00000004456  ENSRNOT00000059133 | RGD1561555\_predicted |  | 3 |
| 1368223\_at | Q9WUQ1 | 79252 | ENSRNOG00000001607 | ENSRNOT00000002187 | Adamts1 | ADAMTS-1 precursor (EC 3.4.24.-) (A disintegrin and metalloproteinase with thrombospondin motifs 1) (ADAM-TS 1) (ADAM-TS1). [Source:UniProtKB/Swiss-Prot;Acc:Q9WUQ1] | 3 |
| 1372711\_at |  | 498750 | ENSRNOG00000018456 | ENSRNOT00000059564 | LOC498750 | similar to cDNA sequence BC005537 (LOC498750), mRNA [Source:RefSeq\_dna;Acc:NM\_001017510] | 3 |
| 1379853\_at |  |  | ENSRNOG00000010241 | ENSRNOT00000013615  ENSRNOT00000057362 | Hspb11 | heat shock protein family B (small), member 11 Gene [Source:MGI Symbol;Acc:MGI:1920188] | 3 |
| 1372400\_at |  | 361181 | ENSRNOG00000019649 | ENSRNOT00000026653 | RGD1563853\_predicted |  | 3 |
| 1370932\_at | Q9QYP1 | 83469 | ENSRNOG00000015285 | ENSRNOT00000021353 | Lrp4 | Low-density lipoprotein receptor-related protein 4 precursor (Multiple epidermal growth factor-like domains 7). [Source:UniProtKB/Swiss-Prot;Acc:Q9QYP1] | 3 |
| 1368177\_at | Q63151 | 114024 | ENSRNOG00000014718 | ENSRNOT00000020161 | Acsl3 | Long-chain-fatty-acid--CoA ligase 3 (EC 6.2.1.3) (Long-chain acyl-CoA synthetase 3) (LACS 3) (Brain acyl-CoA synthetase II). [Source:UniProtKB/Swiss-Prot;Acc:Q63151] | 3 |
| 1393373\_at |  | 498289 | ENSRNOG00000003778 | ENSRNOT00000005072 | LOC498289 |  | 3 |
| 1398854\_at | P83732 | 64307  367865 | ENSRNOG00000001611  ENSRNOG00000023733 | ENSRNOT00000002194  ENSRNOT00000034329 | Rpl24  RGD1560821\_predicted | 60S ribosomal protein L24 (L30). [Source:UniProtKB/Swiss-Prot;Acc:P83732] | 3 |
| 1377281\_at |  | 305851 | ENSRNOG00000011953 | ENSRNOT00000016288 | NP\_001100731.1 | suppressor of Ty 16 homolog [Source:RefSeq\_peptide;Acc:NP\_001100731] | 3 |
| 1370585\_a\_at | P68403 |  | ENSRNOG00000012061 | ENSRNOT00000016442 | Prkcb1 | Protein kinase C beta type (EC 2.7.11.13) (PKC-beta) (PKC-B). [Source:UniProtKB/Swiss-Prot;Acc:P68403] | 3 |
| 1373214\_at |  | 316370 | ENSRNOG00000011740 | ENSRNOT00000016285 | NP\_001101687.1 | KDEL (Lys-Asp-Glu-Leu) containing 1 [Source:RefSeq\_peptide;Acc:NP\_001101687] | 3 |
| 1388389\_at | Q91Y81 | 117515 | ENSRNOG00000017952 | ENSRNOT00000024261 | Sept2 | Septin-2 (Vascular endothelial cell specific protein 11). [Source:UniProtKB/Swiss-Prot;Acc:Q91Y81] | 3 |
| 1393780\_at |  | 310375 | ENSRNOG00000015925 | ENSRNOT00000021608  ENSRNOT00000021484 | Zfp131 | Zfp131 protein (Fragment). [Source:UniProtKB/TrEMBL;Acc:Q4G013] | 3 |
| 1375337\_at |  | 290834 | ENSRNOG00000017231 | ENSRNOT00000041330 | Adam9 | ADAM metallopeptidase domain 9 [Source:RefSeq\_peptide;Acc:NP\_001014772] | 3 |
| 1371081\_at | Q9Z1C7 |  | ENSRNOG00000001516 | ENSRNOT00000048797 | Rapgef4 | Rap guanine nucleotide exchange factor 4 (cAMP-regulated guanine nucleotide exchange factor II) (cAMP-GEFII) (Exchange factor directly activated by cAMP 2) (Epac 2) (Fragment). [Source:UniProtKB/Swiss-Prot;Acc:Q9Z1C7] | 3 |
| 1389981\_at |  | 361317 | ENSRNOG00000027250 | ENSRNOT00000060199 | NP\_001101899.1 | RNA binding motif protein 27 [Source:RefSeq\_peptide;Acc:NP\_001101899] | 3 |
| 1383822\_at |  | 306809 | ENSRNOG00000016031 | ENSRNOT00000021701  ENSRNOT00000048653 | Bicd2 | bicaudal D homolog 2 isoform 1 [Source:RefSeq\_peptide;Acc:NP\_001028846] | 3 |
| 1399022\_at |  | 301434 | ENSRNOG00000025768 | ENSRNOT00000030934 | NP\_001100383.1 | CDC-like kinase 1 [Source:RefSeq\_peptide;Acc:NP\_001100383] | 3 |
| 1368199\_at | O08658 | 113929 | ENSRNOG00000006126 | ENSRNOT00000056587  ENSRNOT00000056586  ENSRNOT00000009015 | Nup88 | Nuclear pore complex protein Nup88 (Nucleoporin Nup88) (88 kDa nuclear pore complex protein) (Nucleoporin Nup84). [Source:UniProtKB/Swiss-Prot;Acc:O08658] | 3 |
| 1377102\_at |  | 289318 | ENSRNOG00000003310 | ENSRNOT00000004519 | Tmem63a\_predicted |  | 3 |
| 1398980\_at |  | 305851 | ENSRNOG00000011953 | ENSRNOT00000016288 | NP\_001100731.1 | suppressor of Ty 16 homolog [Source:RefSeq\_peptide;Acc:NP\_001100731] | 3 |
| 1368726\_a\_at |  | 170902 | ENSRNOG00000031216 | ENSRNOT00000044241 | Zfp347 | zinc finger protein 347 [Source:RefSeq\_peptide;Acc:NP\_596881] | 3 |
| 1399154\_at |  |  | ENSRNOG00000019145 | ENSRNOT00000054850 | NP\_001101985.1 | F-box and leucine-rich repeat protein 11 [Source:RefSeq\_peptide;Acc:NP\_001101985] | 3 |
| 1380393\_at | Q6AYT0 | 362061 | ENSRNOG00000028319 | ENSRNOT00000032808 | Cryz | Quinone oxidoreductase (EC 1.6.5.5) (NADPH:quinone reductase) (Zeta- crystallin). [Source:UniProtKB/Swiss-Prot;Acc:Q6AYT0] | 3 |
| 1384411\_at |  | 679270  680817 | ENSRNOG00000008433 | ENSRNOT00000011138 | LOC680817 |  | 3 |
| 1398308\_at |  | 296883 | ENSRNOG00000008309  ENSRNOG00000037481 | ENSRNOT00000011123  ENSRNOT00000056733 | NP\_001100054.1 | replication protein A3 [Source:RefSeq\_peptide;Acc:NP\_001100054] | 3 |
| 1388865\_at |  |  | ENSRNOG00000024647 | ENSRNOT00000029115 | Ppp4r2 | protein phosphatase 4, regulatory subunit 2 Gene [Source:MGI Symbol;Acc:MGI:3027896] | 3 |
| 1373387\_at |  | 681987  688717 | ENSRNOG00000012314 | ENSRNOT00000016479 | LOC681987 | similar to CG33714-PB, isoform B (LOC688717), mRNA [Source:RefSeq\_dna;Acc:NM\_001109507] | 3 |
| 1372934\_at |  | 299209 | ENSRNOG00000010194 | ENSRNOT00000013556  ENSRNOT00000013560 | RGD1307392\_predicted |  | 3 |
| 1367666\_at |  |  | ENSRNOG00000003399 | ENSRNOT00000004572 | Hnrph1 | Heterogeneous nuclear ribonucleoprotein H (hnRNP H) (Ratsg1). [Source:UniProtKB/Swiss-Prot;Acc:Q8VHV7] | 3 |
| 1375632\_at | P63174 | 681221  685963  690468  681502  682969  689671  689284  690833  686435  682793  682065  688248  687648  686066  502741  678816  680512  679405  680353 | ENSRNOG00000031798  ENSRNOG00000031434  ENSRNOG00000032847  ENSRNOG00000033808  ENSRNOG00000029377  ENSRNOG00000033484  ENSRNOG00000033686  ENSRNOG00000030747  ENSRNOG00000031332  ENSRNOG00000036729 | ENSRNOT00000043427  ENSRNOT00000040570  ENSRNOT00000049811  ENSRNOT00000043108  ENSRNOT00000046518  ENSRNOT00000048963  ENSRNOT00000050489  ENSRNOT00000042661  ENSRNOT00000044141  ENSRNOT00000030800 | Rpl38  LOC687648  RGD1561636\_predicted  LOC680512  LOC680353 | 60S ribosomal protein L38. [Source:UniProtKB/Swiss-Prot;Acc:P63174] | 3 |
| 1398998\_at |  | 302913 | ENSRNOG00000002545 | ENSRNOT00000003439 | NP\_001100442.1 | similar to CG4768-PA (predicted) (RGD1309748\_predicted), mRNA [Source:RefSeq\_dna;Acc:NM\_001106972] | 3 |
| 1374308\_at | Q99MS0 | 116486 | ENSRNOG00000004672 | ENSRNOT00000006542 | Sec14l2 | SEC14-like protein 2 (Alpha-tocopherol-associated protein) (TAP) (Supernatant protein factor) (SPF) (Squalene transfer protein). [Source:UniProtKB/Swiss-Prot;Acc:Q99MS0] | 3 |
| 1392502\_at |  | 360886 | ENSRNOG00000023541 | ENSRNOT00000035875 | Ahctf1\_predicted |  | 3 |
| 1383410\_at | Q6AYB5 | 116650 | ENSRNOG00000032776 | ENSRNOT00000049937 | Srp54 | Signal recognition particle 54 kDa protein (SRP54). [Source:UniProtKB/Swiss-Prot;Acc:Q6AYB5] | 3 |
| 1394086\_at |  | 288167 | ENSRNOG00000001616 | ENSRNOT00000044177 | NP\_001099358.1 | SUMO1/sentrin specific protease 7 [Source:RefSeq\_peptide;Acc:NP\_001099358] | 3 |
| 1387077\_at | Q712U5 | 60336 | ENSRNOG00000023086  ENSRNOG00000020977 | ENSRNOT00000030221  ENSRNOT00000057268  ENSRNOT00000028470 | Arpp19 | cAMP-regulated phosphoprotein 19 (ARPP-19). [Source:UniProtKB/Swiss-Prot;Acc:Q712U5] | 3 |
| 1377728\_at |  | 499567 | ENSRNOG00000038902 | ENSRNOT00000059467 | NP\_001102649.1 |  | 3 |
| 1388739\_at |  | 314399 | ENSRNOG00000008108 | ENSRNOT00000010739 | RGD1359144 | similar to chromosome 14 open reading frame 130 (RGD1359144), mRNA [Source:RefSeq\_dna;Acc:NM\_001007705] | 3 |
| 1387775\_at | O08950 | 83828 | ENSRNOG00000011062 | ENSRNOT00000014706 | Gtf2a2 | Transcription initiation factor IIA gamma chain (TFIIA P12 subunit) (TFIIA-12) (TFIIAS) (TFIIA-gamma). [Source:UniProtKB/Swiss-Prot;Acc:O08950] | 3 |
| 1371837\_at |  | 287765 | ENSRNOG00000030680 | ENSRNOT00000048490 | Ddx5 | ddx5 [Source:RefSeq\_peptide;Acc:NP\_001007614] | 3 |
| 1393317\_at |  | 500288 | ENSRNOG00000006941 | ENSRNOT00000009464 | RGD1560852\_predicted |  | 3 |
| 1380035\_at |  | 500501 | ENSRNOG00000007299 | ENSRNOT00000010173 | MGC125002 | similar to RIKEN cDNA 5830433M19 (MGC125002), mRNA [Source:RefSeq\_dna;Acc:NM\_001034154] | 3 |
| 1376714\_at | Q5U201 | 302032 | ENSRNOG00000036917 | ENSRNOT00000055441 | RGD1308119 | antagonist of mitotic exit network 1 homolog [Source:RefSeq\_peptide;Acc:NP\_001008334] | 3 |
| 1374028\_at |  |  | ENSRNOG00000009950 | ENSRNOT00000013370 | Q5BJL6\_RAT | LOC500974 protein (Fragment). [Source:UniProtKB/TrEMBL;Acc:Q5BJL6] | 3 |
| 1368323\_at | Q02445 | 29436 | ENSRNOG00000005039 | ENSRNOT00000006787 | Tfpi | Tissue factor pathway inhibitor precursor (TFPI) (Lipoprotein- associated coagulation inhibitor) (LACI) (Extrinsic pathway inhibitor) (EPI). [Source:UniProtKB/Swiss-Prot;Acc:Q02445] | 3 |
| 1391560\_at |  |  | ENSRNOG00000014460 | ENSRNOT00000019389 | Hivep1 | human immunodeficiency virus type I enhancer binding protein 1 Gene [Source:MGI Symbol;Acc:MGI:96100] | 3 |
| 1374704\_at | Q566E5 | 315664 | ENSRNOG00000007177 | ENSRNOT00000009497 | Kdelc2 | KDEL motif-containing protein 2 precursor. [Source:UniProtKB/Swiss-Prot;Acc:Q566E5] | 3 |
| 1391412\_at |  | 362229 | ENSRNOG00000011785 | ENSRNOT00000016351 | NP\_001102066.1 | 5'-3' exoribonuclease 2 [Source:RefSeq\_peptide;Acc:NP\_001102066] | 3 |
| 1376606\_a\_at |  | 682575  685258 | ENSRNOG00000004681 | ENSRNOT00000006402 |  |  | 3 |
| 1394095\_at |  | 316687 | ENSRNOG00000011962 | ENSRNOT00000016028 | RGD1307449 |  | 3 |
| 1382614\_at |  | 499508 | ENSRNOG00000013055 | ENSRNOT00000017705 | RGD1564784\_predicted |  | 3 |
| 1383256\_at |  |  | ENSRNOG00000025025 | ENSRNOT00000036931 | Dnttip2 | deoxynucleotidyltransferase, terminal, interacting protein 2 Gene [Source:MGI Symbol;Acc:MGI:1923173] | 3 |
| 1389968\_at |  | 292148 | ENSRNOG00000010117 | ENSRNOT00000013559 | Eif3s10 | eukaryotic translation initiation factor 3, subunit 10 (theta) [Source:RefSeq\_peptide;Acc:NP\_001040552] | 3 |
| 1390933\_a\_at | Q5RJK3 | 298081 | ENSRNOG00000012454 | ENSRNOT00000016720 | Rg9mtd3 | RNA (guanine-9-)-methyltransferase domain-containing protein 3 (EC 2.1.1.-). [Source:UniProtKB/Swiss-Prot;Acc:Q5RJK3] | 3 |
| 1382739\_at | P70585 | 312787 | ENSRNOG00000007126 | ENSRNOT00000009341 | Gpr19 | Probable G-protein coupled receptor 19. [Source:UniProtKB/Swiss-Prot;Acc:P70585] | 3 |
| 1381349\_a\_at |  | 363188 | ENSRNOG00000015813 | ENSRNOT00000021158  ENSRNOT00000061531  ENSRNOT00000021540 | LOC363188 |  | 3 |
| 1392646\_at |  |  | ENSRNOG00000006841 | ENSRNOT00000009084 | Ano4 | anoctamin 4 Gene [Source:MGI Symbol;Acc:MGI:2443344] | 3 |
| 1373492\_at | Q5RJQ7 | 361726 | ENSRNOG00000020646 | ENSRNOT00000028028 | RGD1309216 | Protein EMI5 homolog, mitochondrial precursor. [Source:UniProtKB/Swiss-Prot;Acc:Q5RJQ7] | 3 |
| 1383736\_at | Q8CH84 | 286973 | ENSRNOG00000006853 | ENSRNOT00000009035 | Elavl2 | ELAV-like protein 2 (Hu-antigen B) (HuB). [Source:UniProtKB/Swiss-Prot;Acc:Q8CH84] | 3 |
| 1392273\_at |  |  | ENSRNOG00000026180 | ENSRNOT00000030489 | Letm2 | leucine zipper-EF-hand containing transmembrane protein 2 Gene [Source:MGI Symbol;Acc:MGI:2444979] | 3 |
| 1368136\_at | Q62733 | 25359 | ENSRNOG00000008797 | ENSRNOT00000044146  ENSRNOT00000012715 | Tmpo | Lamina-associated polypeptide 2 isoform beta (Thymopoietin isoform beta) (TP beta). [Source:UniProtKB/Swiss-Prot;Acc:Q62733] | 3 |
| 1373381\_at | Q5PQN1 | 309758 | ENSRNOG00000000381 | ENSRNOT00000051367  ENSRNOT00000000428 | Herc4 | Probable E3 ubiquitin-protein ligase HERC4 (EC 6.3.2.-) (HECT domain and RCC1-like domain-containing protein 4). [Source:UniProtKB/Swiss-Prot;Acc:Q5PQN1] | 3 |
| 1384437\_at |  | 317575 | ENSRNOG00000003762 | ENSRNOT00000005111 | Smarca1 | SWI/SNF related, matrix associated, actin dependent regulator of chromatin, subfamily a, member 1 Gene [Source:MGI (curated);Acc:Smarca1-002] | 3 |
| 1395375\_at | Q6P5P3 | 309196 | ENSRNOG00000019464 | ENSRNOT00000026376 | Ttc9c | Tetratricopeptide repeat protein 9C (TPR repeat protein 9C). [Source:UniProtKB/Swiss-Prot;Acc:Q6P5P3] | 3 |
| 1391356\_at |  |  | ENSRNOG00000014630 | ENSRNOT00000020142 | Iws1 | IWS1 homolog (IWS1-like protein). [Source:UniProtKB/Swiss-Prot;Acc:Q3SWT4] | 3 |
| 1376249\_at | Q6AYS4 | 292485 | ENSRNOG00000015551 | ENSRNOT00000020946 | Fuca2 | Plasma alpha-L-fucosidase precursor (EC 3.2.1.51) (Alpha-L-fucosidase 2) (Alpha-L-fucoside fucohydrolase 2). [Source:UniProtKB/Swiss-Prot;Acc:Q6AYS4] | 3 |
| 1370827\_at | Q68EJ0 | 171015 | ENSRNOG00000010024 | ENSRNOT00000048595  ENSRNOT00000013908 | Cyb5r4 | Cytochrome b5 reductase 4 (EC 1.6.2.2) (Flavohemoprotein b5/b5R) (b5+b5R) (cb5/cb5R) (N-terminal cytochrome b5 and cytochrome b5 oxidoreductase domain-containing protein). [Source:UniProtKB/Swiss-Prot;Acc:Q68EJ0] | 3 |
| 1392488\_at |  | 307526 | ENSRNOG00000016250 | ENSRNOT00000061074 | NP\_001100869.1 |  | 3 |
| 1373164\_at | Q91XS8 | 170904 | ENSRNOG00000012502 | ENSRNOT00000016856 | Stk17b | Serine/threonine-protein kinase 17B (EC 2.7.11.1) (DAP kinase-related apoptosis-inducing protein kinase 2). [Source:UniProtKB/Swiss-Prot;Acc:Q91XS8] | 3 |
| 1387186\_at | Q99P75 | 84589 | ENSRNOG00000030443 | ENSRNOT00000050018 | Rab9 | Ras-related protein Rab-9A (Rab-9A). [Source:UniProtKB/Swiss-Prot;Acc:Q99P75] | 3 |
| 1374170\_at |  | 498749 | ENSRNOG00000018246 | ENSRNOT00000059570 | LOC498749 | similar to putative TRAF and TNF receptor associated protein (LOC498749), mRNA [Source:RefSeq\_dna;Acc:NM\_001034947] | 3 |
| 1377503\_at |  | 308201 | ENSRNOG00000012692 | ENSRNOT00000017165 | Riok2 | RIO kinase 2 [Source:RefSeq\_peptide;Acc:NP\_001009687] | 3 |
| 1375853\_at |  | 314690 | ENSRNOG00000008331 | ENSRNOT00000011326 | RGD1309995\_predicted |  | 3 |
| 1382923\_at |  |  | ENSRNOG00000000204 | ENSRNOT00000056804  ENSRNOT00000000222  ENSRNOT00000056803 | Syncrip | Heterogeneous nuclear ribonucleoprotein Q (hnRNP Q) (hnRNP-Q) (Synaptotagmin-binding, cytoplasmic RNA-interacting protein) (Liver regeneration-related protein LRRG077). [Source:UniProtKB/Swiss-Prot;Acc:Q7TP47] | 3 |
| 1377532\_at |  | 311575 | ENSRNOG00000019948 | ENSRNOT00000045551  ENSRNOT00000040939  ENSRNOT00000027067 | NP\_001101265.1 | similar to Hepatocellular carcinoma-associated antigen 58 homolog (predicted) (RGD1305020\_predicted), mRNA [Source:RefSeq\_dna;Acc:NM\_001107795] | 3 |
| 1387977\_at | Q9JIL9 | 85482 | ENSRNOG00000008580 | ENSRNOT00000012377 | Nbn | Nibrin (Nijmegen breakage syndrome protein 1 homolog). [Source:UniProtKB/Swiss-Prot;Acc:Q9JIL9] | 3 |
| 1388936\_at |  | 84407 | ENSRNOG00000013481 | ENSRNOT00000018535 | Cdh11 | Cadherin-11 (Fragment). [Source:UniProtKB/TrEMBL;Acc:Q9JIW2] | 3 |
| 1383160\_at |  | 315447 | ENSRNOG00000026643 | ENSRNOT00000030569 | NP\_001101598.1 | cysteine and histidine-rich domain (CHORD)-containing, zinc-binding protein 1 [Source:RefSeq\_peptide;Acc:NP\_001101598] | 3 |
| 1380320\_at |  | 305171 | ENSRNOG00000002203 | ENSRNOT00000003006 | RGD1311361 | RGD1311361 protein (Fragment). [Source:UniProtKB/TrEMBL;Acc:Q641Z1] | 3 |
| 1397853\_s\_at |  |  | ENSRNOG00000007733 | ENSRNOT00000037863 | Arhgef9 | Rho guanine nucleotide exchange factor 9 (Rac/Cdc42 guanine nucleotide exchange factor 9) (Collybistin). [Source:UniProtKB/Swiss-Prot;Acc:Q9QX73] | 3 |
| 1380101\_at |  | 305586 | ENSRNOG00000006261 | ENSRNOT00000008919 | NP\_001100714.1 | poly(A) polymerase gamma [Source:RefSeq\_peptide;Acc:NP\_001100714] | 3 |
| 1387179\_at | P40146 | 29241 | ENSRNOG00000004890 | ENSRNOT00000006789 | Adcy8 | Adenylate cyclase type 8 (EC 4.6.1.1) (Adenylate cyclase type VIII) (ATP pyrophosphate-lyase 8) (Adenylyl cyclase 8) (Ca(2+)/calmodulin- activated adenylyl cyclase). [Source:UniProtKB/Swiss-Prot;Acc:P40146] | 3 |
| 1376050\_at |  | 294018 | ENSRNOG00000020274 | ENSRNOT00000027482 | NP\_001099835.1 | TAF5 RNA polymerase II, TATA box binding protein (TBP)-associated factor [Source:RefSeq\_peptide;Acc:NP\_001099835] | 3 |
| 1370531\_a\_at | P70496 | 25096 | ENSRNOG00000028156 | ENSRNOT00000039296  ENSRNOT00000039308 | Pld1 | Phospholipase D1 (EC 3.1.4.4) (PLD 1) (Choline phosphatase 1) (Phosphatidylcholine-hydrolyzing phospholipase D1) (rPLD1). [Source:UniProtKB/Swiss-Prot;Acc:P70496] | 3 |
| 1392599\_at |  | 302678 | ENSRNOG00000004304 | ENSRNOT00000006584 | Syap1 | synapse associated protein 1 [Source:RefSeq\_peptide;Acc:NP\_001004253] | 3 |
| 1372804\_at |  | 289562 | ENSRNOG00000002055 | ENSRNOT00000002810  ENSRNOT00000002832 | RGD1359460 | MMR\_HSR1 domain containing protein RGD1359460 [Source:RefSeq\_peptide;Acc:NP\_001006960] | 3 |
| 1372575\_at | Q5PQQ2 | 297695 | ENSRNOG00000005505 | ENSRNOT00000007402 | Wbp11 | WW domain-binding protein 11 (Wbp-11). [Source:UniProtKB/Swiss-Prot;Acc:Q5PQQ2] | 3 |
| 1373186\_at |  |  | ENSRNOG00000002271 | ENSRNOT00000042382 | Slain2 | SLAIN motif family, member 2 Gene [Source:MGI Symbol;Acc:MGI:1923241] | 3 |
| 1374858\_at |  |  | ENSRNOG00000010369 | ENSRNOT00000013854 | Dhx29 | DEAH (Asp-Glu-Ala-His) box polypeptide 29 Gene [Source:MGI Symbol;Acc:MGI:2145374] | 3 |
| 1373190\_at |  | 312227 | ENSRNOG00000010795 | ENSRNOT00000040969 | Cnot4 | CCR4-NOT transcription complex, subunit 4 [Source:RefSeq\_peptide;Acc:NP\_001032871] | 3 |
| 1382294\_at | O35112 | 79559 | ENSRNOG00000001989 | ENSRNOT00000002738 | Alcam | CD166 antigen precursor (Activated leukocyte cell adhesion molecule) (Protein MEMD) (HB2) (SB-10 antigen) (KG-CAM). [Source:UniProtKB/Swiss-Prot;Acc:O35112] | 3 |
| 1398795\_at | P15178 | 116483 | ENSRNOG00000003743 | ENSRNOT00000005127 | Dars | Aspartyl-tRNA synthetase, cytoplasmic (EC 6.1.1.12) (Aspartate--tRNA ligase) (AspRS). [Source:UniProtKB/Swiss-Prot;Acc:P15178] | 3 |
| 1372202\_at | Q5RJT0 | 301374 | ENSRNOG00000016447 | ENSRNOT00000022601 | RGD1310553 | Ashwin. [Source:UniProtKB/Swiss-Prot;Acc:Q5RJT0] | 3 |
| 1390653\_at |  | 314243 | ENSRNOG00000021628 | ENSRNOT00000006860 | RGD1307393 | Putative uncharacterized protein RGD1307393. [Source:UniProtKB/TrEMBL;Acc:Q5FVP5] | 3 |
| 1372339\_at | Q6YDN7 | 366381 | ENSRNOG00000029785 | ENSRNOT00000040894 | Cdc26 | Anaphase-promoting complex subunit CDC26 (Cell division cycle protein 26) (Protein BWK-2). [Source:UniProtKB/Swiss-Prot;Acc:Q6YDN7] | 3 |
| 1370244\_at | P07154 | 25697 | ENSRNOG00000018566 | ENSRNOT00000025462 | Ctsl | Cathepsin L1 precursor (EC 3.4.22.15) (Major excreted protein) (MEP) (Cyclic protein 2) (CP-2) [Contains: Procathepsin L; Cathepsin L1 heavy chain; Cathepsin L1 light chain]. [Source:UniProtKB/Swiss-Prot;Acc:P07154] | 3 |
| 1393276\_at |  | 287475 | ENSRNOG00000014618 | ENSRNOT00000019919 | Med31\_predicted |  | 3 |
| 1395840\_at | Q6VV72 | 317163  302697 | ENSRNOG00000031421 | ENSRNOT00000041237 | Eif1a | Eukaryotic translation initiation factor 1A (eIF-1A) (eIF-4C) (Liver regeneration-related protein LRRG048). [Source:UniProtKB/Swiss-Prot;Acc:Q6VV72] | 3 |
| 1383742\_at |  | 310815 | ENSRNOG00000017077 | ENSRNOT00000022947 | Snx7 | sorting nexin 7 [Source:RefSeq\_peptide;Acc:NP\_001012083] | 3 |
| 1373441\_at |  | 308961 | ENSRNOG00000018048 | ENSRNOT00000024367 | Dctn5 | dynactin 5 [Source:RefSeq\_peptide;Acc:NP\_001032867] | 3 |
| 1399127\_at |  |  | ENSRNOG00000015230 | ENSRNOT00000036303 | RGD1562376\_predicted |  | 3 |
| 1387081\_at | Q62703 | 29218 | ENSRNOG00000015780 | ENSRNOT00000021817 | Rcn2 | Reticulocalbin-2 precursor (Calcium-binding protein ERC-55) (Taipoxin- associated calcium-binding protein 49) (TCBP-49). [Source:UniProtKB/Swiss-Prot;Acc:Q62703] | 3 |
| 1391805\_at |  | 313111 | ENSRNOG00000007441 | ENSRNOT00000050653 | Klhl32 | kelch-like 32 (Drosophila) Gene [Source:MGI (curated);Acc:Klhl32-001] | 3 |
| 1388373\_at |  | 289036 | ENSRNOG00000004143 | ENSRNOT00000005551 | Adipor1 | adiponectin receptor 1 [Source:RefSeq\_peptide;Acc:NP\_997470] | 3 |
| 1373238\_at | Q5BJQ7 | 360874 | ENSRNOG00000003816 | ENSRNOT00000058689 | Tada1l | Transcriptional adapter 1-like protein. [Source:UniProtKB/Swiss-Prot;Acc:Q5BJQ7] | 3 |
| 1373267\_at |  | 362724 | ENSRNOG00000005522 | ENSRNOT00000007330 | NP\_001102175.1 | Sh3 domain YSC-like 1 [Source:RefSeq\_peptide;Acc:NP\_001102175] | 3 |
| 1383826\_at |  | 303754 | ENSRNOG00000036661 | ENSRNOT00000054917 | NP\_001100546.1 | Rab40b, member RAS oncogene family [Source:RefSeq\_peptide;Acc:NP\_001100546] | 3 |
| 1382874\_at | Q66H59 | 304860 | ENSRNOG00000002775 | ENSRNOT00000003713 | LOC304860 | N-acetylneuraminate lyase (EC 4.1.3.3) (NALase) (N-acetylneuraminic acid aldolase) (N-acetylneuraminate pyruvate-lyase) (Sialic acid lyase) (Sialate lyase) (Sialate-pyruvate lyase) (Sialic acid aldolase). [Source:UniProtKB/Swiss-Prot;Acc:Q66H59] | 3 |
| 1368342\_at | O09178 | 25095 | ENSRNOG00000018262 | ENSRNOT00000024933 | Ampd3 | AMP deaminase 3 (EC 3.5.4.6) (AMP deaminase isoform E). [Source:UniProtKB/Swiss-Prot;Acc:O09178] | 3 |
| 1386078\_at |  | 313139 | ENSRNOG00000008908 | ENSRNOT00000011969 | NP\_001101394.1 | solute carrier family 35 (CMP-sialic acid transporter), member 1 [Source:RefSeq\_peptide;Acc:NP\_001101394] | 3 |
| 1368356\_a\_at | Q9JJ22 | 80897 | ENSRNOG00000009997 | ENSRNOT00000013625 | Arts1 | Endoplasmic reticulum aminopeptidase 1 (EC 3.4.11.-) (Adipocyte- derived leucine aminopeptidase) (A-LAP) (ARTS-1) (Aminopeptidase PILS) (Puromycin-insensitive leucyl-specific aminopeptidase) (PILS-AP). [Source:UniProtKB/Swiss-Prot;Acc:Q9JJ22] | 3 |
| 1378027\_at |  |  | ENSRNOG00000002176 | ENSRNOT00000002970 | NP\_001099353.1 | poliovirus receptor-related 3 [Source:RefSeq\_peptide;Acc:NP\_001099353] | 3 |
| 1398774\_at | P62890 | 64640  364060 | ENSRNOG00000021406  ENSRNOG00000032825  ENSRNOG00000033265  ENSRNOG00000005975 | ENSRNOT00000029708  ENSRNOT00000042688  ENSRNOT00000045014  ENSRNOT00000007925  ENSRNOT00000057587 | AC139241.4  RL30\_RAT  RGD1562397\_predicted  Rpl30 | 60S ribosomal protein L30. [Source:UniProtKB/Swiss-Prot;Acc:P62890] | 3 |
| 1390989\_at |  | 363463 | ENSRNOG00000003350 | ENSRNOT00000004494 | RGD1563952\_predicted |  | 3 |
| 1370010\_at | P17046 | 24944 | ENSRNOG00000000164 | ENSRNOT00000000177 | Lamp2 | Lysosome-associated membrane glycoprotein 2 precursor (LAMP-2) (Lysosomal membrane glycoprotein-type B) (LGP-B) (LGP-96) (LGP-110) (CD107b antigen). [Source:UniProtKB/Swiss-Prot;Acc:P17046] | 3 |
| 1368588\_at | Q99PT0 | 85432 | ENSRNOG00000002612 | ENSRNOT00000003600 | Ddx52 | Probable ATP-dependent RNA helicase DDX52 (EC 3.6.1.-) (DEAD box protein 52) (ATP-dependent RNA helicase ROK1-like) (rROK1L). [Source:UniProtKB/Swiss-Prot;Acc:Q99PT0] | 3 |
| 1371963\_at | P14882 | 687008 | ENSRNOG00000014146 | ENSRNOT00000019144  ENSRNOT00000057427 | PCCA\_RAT | Propionyl-CoA carboxylase alpha chain, mitochondrial precursor (EC 6.4.1.3) (PCCase subunit alpha) (Propanoyl-CoA:carbon dioxide ligase subunit alpha) (Fragment). [Source:UniProtKB/Swiss-Prot;Acc:P14882] | 3 |
| 1367515\_at |  | 306492 | ENSRNOG00000012263 | ENSRNOT00000016882 | NP\_001100783.1 | CCR4-NOT transcription complex, subunit 7 [Source:RefSeq\_peptide;Acc:NP\_001100783] | 3 |
| 1398441\_at |  | 316369 | ENSRNOG00000017155 | ENSRNOT00000023029 | NP\_001101686.1 | non-catalytic region of tyrosine kinase adaptor protein 2 [Source:RefSeq\_peptide;Acc:NP\_001101686] | 3 |
| 1382642\_at |  | 305083 | ENSRNOG00000009099 | ENSRNOT00000012085 | Zfp281 | zinc finger protein 281 [Source:RefSeq\_peptide;Acc:NP\_001012030] | 3 |
| 1398888\_at |  |  | ENSRNOG00000006532 | ENSRNOT00000050223 | H3f3b | H3 histone, family 3B (H3f3b), mRNA [Source:RefSeq\_dna;Acc:NM\_053985] | 3 |
| 1374061\_at | Q5FVR3 | 295629 | ENSRNOG00000006623 | ENSRNOT00000008987  ENSRNOT00000009160 | Cd302 | CD302 antigen precursor (C-type lectin domain family 13 member A). [Source:UniProtKB/Swiss-Prot;Acc:Q5FVR3] | 3 |
| 1376102\_at |  | 316516 | ENSRNOG00000014797 | ENSRNOT00000019848 | Tmbim1 | transmembrane BAX inhibitor motif containing 1 [Source:RefSeq\_peptide;Acc:NP\_001007714] | 3 |
| 1390419\_a\_at |  | 290783 | ENSRNOG00000013061 | ENSRNOT00000017452 | Tusc3 | tumor suppressor candidate 3 [Source:RefSeq\_peptide;Acc:NP\_001004212] | 3 |
| 1370030\_at | P48508 | 29739 | ENSRNOG00000013409 | ENSRNOT00000018343 | Gclm | Glutamate--cysteine ligase regulatory subunit (EC 6.3.2.2) (Gamma- glutamylcysteine synthetase regulatory subunit) (Gamma-ECS regulatory subunit) (GCS light chain) (Glutamate--cysteine ligase modifier subunit). [Source:UniProtKB/Swiss-Prot;Acc:P48508] | 3 |
| 1395516\_at | Q5BJT0 | 290912 | ENSRNOG00000024142 | ENSRNOT00000046755 | RGD1310061 | Arginine and glutamate-rich protein 1. [Source:UniProtKB/Swiss-Prot;Acc:Q5BJT0] | 3 |
| 1367713\_at | P68101 | 54318  364984 | ENSRNOG00000009432 | ENSRNOT00000013375 | Eif2s1 | Eukaryotic translation initiation factor 2 subunit 1 (Eukaryotic translation initiation factor 2 subunit alpha) (eIF-2-alpha) (EIF- 2alpha) (EIF-2A). [Source:UniProtKB/Swiss-Prot;Acc:P68101] | 3 |
| 1398455\_at |  | 365252 | ENSRNOG00000014277 | ENSRNOT00000019175 | Zdhhc13 | zinc finger, DHHC domain containing 13 [Source:RefSeq\_peptide;Acc:NP\_001034126] | 3 |
| 1382059\_at | Q5XI67 | 308283 | ENSRNOG00000014852 | ENSRNOT00000019918 | Fbxo30 | F-box only protein 30. [Source:UniProtKB/Swiss-Prot;Acc:Q5XI67] | 3 |
| 1390875\_a\_at | Q66H89 | 366872 | ENSRNOG00000007859 | ENSRNOT00000034639 | RGD1359593 | Coiled-coil domain-containing protein 41. [Source:UniProtKB/Swiss-Prot;Acc:Q66H89] | 3 |
| 1393283\_at |  | 499506 | ENSRNOG00000040166 | ENSRNOT00000061812 | NP\_001102644.1 | similar to cytosolic phosphoprotein DP58 (LOC499506), mRNA [Source:RefSeq\_dna;Acc:NM\_001109174] | 3 |
| 1388752\_at |  |  | ENSRNOG00000013052 | ENSRNOT00000049492 | Q7TP73\_RAT | Aa2-041. [Source:UniProtKB/TrEMBL;Acc:Q7TP73] | 3 |
| 1388886\_at |  | 497865 | ENSRNOG00000002874 | ENSRNOT00000003834 | RGD1565486\_predicted |  | 3 |
| 1370243\_a\_at | P06302 | 29222  500448 | ENSRNOG00000025731  ENSRNOG00000006571  ENSRNOG00000018584 | ENSRNOT00000036412  ENSRNOT00000008592  ENSRNOT00000025093 | Ptma | Prothymosin alpha [Contains: Thymosin alpha]. [Source:UniProtKB/Swiss-Prot;Acc:P06302] | 3 |
| 1376681\_at | Q5U4F0 | 308911 | ENSRNOG00000018766 | ENSRNOT00000025651 | RGD1308302 | Cerebral protein 1 homolog. [Source:UniProtKB/Swiss-Prot;Acc:Q5U4F0] | 3 |
| 1392943\_at | Q3T1J2 |  | ENSRNOG00000006777 | ENSRNOT00000009018 | MBOA2\_RAT | Membrane-bound O-acyltransferase domain-containing protein 2 (EC 2.3.-.-) (O-acyltransferase domain-containing protein 2). [Source:UniProtKB/Swiss-Prot;Acc:Q3T1J2] | 3 |
| 1372372\_at | Q7TP52 | 310201 | ENSRNOG00000011260 | ENSRNOT00000015448 | RGD1306952 | Carboxymethylenebutenolidase homolog (EC 3.1.-.-) (Liver regeneration- related protein LRRG072). [Source:UniProtKB/Swiss-Prot;Acc:Q7TP52] | 3 |
| 1397892\_at | Q6VV72 | 317163  302697 | ENSRNOG00000031421 | ENSRNOT00000041237 | Eif1a | Eukaryotic translation initiation factor 1A (eIF-1A) (eIF-4C) (Liver regeneration-related protein LRRG048). [Source:UniProtKB/Swiss-Prot;Acc:Q6VV72] | 3 |
| 1370828\_at | Q9JKR5 | 246326 | ENSRNOG00000022686 | ENSRNOT00000036092 | Zdhhc2 | Palmitoyltransferase ZDHHC2 (EC 2.3.1.-) (Zinc finger DHHC domain- containing protein 2) (DHHC-2). [Source:UniProtKB/Swiss-Prot;Acc:Q9JKR5] | 3 |
| 1367826\_at | O54968 | 83619 | ENSRNOG00000001548 | ENSRNOT00000002114 | Nfe2l2 | Nuclear factor erythroid 2-related factor 2 (NF-E2-related factor 2) (NFE2-related factor 2) (Nuclear factor, erythroid derived 2, like 2). [Source:UniProtKB/Swiss-Prot;Acc:O54968] | 3 |
| 1373015\_at |  | 683844  316552  683019  689577 | ENSRNOG00000016205 | ENSRNOT00000021681 | Rnf11\_predicted |  | 3 |
| 1371384\_at |  | 683456  294680  498293  500914 | ENSRNOG00000016912  ENSRNOG00000031864  ENSRNOG00000029243 | ENSRNOT00000022864  ENSRNOT00000042547  ENSRNOT00000049445 | RGD1564126\_predicted  RGD1563812\_predicted | basic transcription factor 3 [Source:RefSeq\_peptide;Acc:NP\_001008310]  basic transcription factor 3 (Btf3), mRNA [Source:RefSeq\_dna;Acc:NM\_001008309] | 3 |
| 1379343\_at | Q6AY04 | 301418 | ENSRNOG00000015983 | ENSRNOT00000021533 | MGC94335 | Uncharacterized protein C2orf47 homolog, mitochondrial precursor. [Source:UniProtKB/Swiss-Prot;Acc:Q6AY04] | 3 |
| 1371715\_at | Q4G019 | 500651 | ENSRNOG00000004292 | ENSRNOT00000005825 | MGC112883 | UPF0445 protein C14orf147 homolog. [Source:UniProtKB/Swiss-Prot;Acc:Q4G019] | 3 |
| 1390022\_at | Q4KLF8 | 360854 | ENSRNOG00000028062 | ENSRNOT00000036716 | Arpc5 | Actin-related protein 2/3 complex subunit 5 (Arp2/3 complex 16 kDa subunit) (p16-ARC). [Source:UniProtKB/Swiss-Prot;Acc:Q4KLF8] | 3 |
| 1390692\_at |  | 313560  299504 | ENSRNOG00000009963 | ENSRNOT00000013899 | Ctps\_predicted |  | 3 |
| 1374232\_at |  | 685605  685590  685626  170911  685653 | ENSRNOG00000024392  ENSRNOG00000010458 | ENSRNOT00000033390  ENSRNOT00000058875  ENSRNOT00000058874  ENSRNOT00000014527 | Pik3ca  LOC685653 | Phosphatidylinositol 3-kinase alpha catalytic subunit (EC 2.7.1.137) (Fragment). [Source:UniProtKB/TrEMBL;Acc:Q91XL6] | 3 |
| 1390893\_at |  | 619582 | ENSRNOG00000029152 | ENSRNOT00000050434 | Tmem69 | transmembrane protein 69 [Source:RefSeq\_peptide;Acc:NP\_001030173] | 3 |
| 1393051\_at | Q5U310 | 501619 | ENSRNOG00000037709 | ENSRNOT00000052422  ENSRNOT00000029833 | LOC501619 | Armadillo repeat-containing X-linked protein 1. [Source:UniProtKB/Swiss-Prot;Acc:Q5U310] | 3 |
| 1376366\_at | P60192 | 295217 | ENSRNOG00000013356 | ENSRNOT00000018830 | Snapap | SNARE-associated protein Snapin (Synaptosomal-associated protein 25- binding protein) (SNAP-associated protein). [Source:UniProtKB/Swiss-Prot;Acc:P60192] | 3 |
| 1374909\_at |  | 682955  679725 | ENSRNOG00000030247 | ENSRNOT00000033556  ENSRNOT00000024825  ENSRNOT00000024789 | LOC679725 | LOC679725 protein (Fragment). [Source:UniProtKB/TrEMBL;Acc:Q5XI12] | 3 |
| 1372303\_at |  | 299909 | ENSRNOG00000005145 | ENSRNOT00000007528 | RGD1307697\_predicted |  | 3 |
| 1375529\_at |  | 359725 | ENSRNOG00000024411 | ENSRNOT00000032199 | Cbr4 | carbonic reductase 4 [Source:RefSeq\_peptide;Acc:NP\_872613] | 3 |
| 1390298\_at |  |  | ENSRNOG00000010971 | ENSRNOT00000014594 | Snx18 | sorting nexin 18 Gene [Source:MGI Symbol;Acc:MGI:2137642] | 3 |
| 1398848\_at | P50503 | 81800  297828 | ENSRNOG00000019070 | ENSRNOT00000025925 | St13 | Hsc70-interacting protein (Hip) (Protein ST13 homolog) (Protein FAM10A1). [Source:UniProtKB/Swiss-Prot;Acc:P50503] | 3 |
| 1383424\_at |  | 314004 | ENSRNOG00000007690 | ENSRNOT00000052003 | NP\_001101487.1 | thymidylate kinase family LPS-inducible member [Source:RefSeq\_peptide;Acc:NP\_001101487] | 3 |
| 1372455\_at | Q569A2 | 362326 | ENSRNOG00000005272 | ENSRNOT00000007020 | Tm4sf12 | Tetraspanin-12 (Tspan-12) (Transmembrane 4 superfamily member 12). [Source:UniProtKB/Swiss-Prot;Acc:Q569A2] | 3 |
| 1387227\_at | Q6IN36 | 117538 | ENSRNOG00000018406 | ENSRNOT00000024922 | Waspip | WAS/WASL-interacting protein family member 1 (Wiskott-Aldrich syndrome protein-interacting protein) (WASP-interacting protein). [Source:UniProtKB/Swiss-Prot;Acc:Q6IN36] | 3 |
| 1374401\_at |  | 291464 | ENSRNOG00000017832 | ENSRNOT00000050631  ENSRNOT00000024541 | Snx2\_predicted | sorting nexin 2 [Source:RefSeq\_peptide;Acc:NP\_001099605] | 3 |
| 1372815\_at |  | 684298  298385  690303 | ENSRNOG00000012778 | ENSRNOT00000017422 | Magoh\_predicted | Mago-nashi-like proliferation-associated protein (Fragment). [Source:UniProtKB/TrEMBL;Acc:Q27W02] | 3 |
| 1376010\_at |  | 291078 | ENSRNOG00000016705 | ENSRNOT00000022902 | Prpf4b | PRP4 pre-mRNA processing factor 4 homolog B [Source:RefSeq\_peptide;Acc:NP\_001011923] | 3 |
| 1374487\_at |  | 300797 | ENSRNOG00000017119 | ENSRNOT00000022999 | RGD1307481 | family with sequence similarity 96, member A [Source:RefSeq\_peptide;Acc:NP\_001008328] | 3 |
| 1376964\_at |  | 302661 | ENSRNOG00000004574 | ENSRNOT00000006074 | NP\_001100431.1 | similar to Ofd1 protein (predicted) (RGD1562231\_predicted), mRNA [Source:RefSeq\_dna;Acc:NM\_001106961] | 3 |
| 1394992\_at | Q9Z142 | 59303 | ENSRNOG00000002254 | ENSRNOT00000003128 | Tmem33 | Transmembrane protein 33 (DB83 protein). [Source:UniProtKB/Swiss-Prot;Acc:Q9Z142] | 3 |
| 1372151\_at |  | 307474 | ENSRNOG00000018849 | ENSRNOT00000025467 | NP\_001100860.1 | transcription elongation regulator 1 (CA150) [Source:RefSeq\_peptide;Acc:NP\_001100860] | 3 |
| 1391169\_at |  | 500921 | ENSRNOG00000006068 | ENSRNOT00000008159 | RGD1562562\_predicted |  | 3 |
| 1367583\_at | P63029 | 683302  685718  116646  289930  500923 | ENSRNOG00000033517  ENSRNOG00000030299  ENSRNOG00000001049  ENSRNOG00000000896  ENSRNOG00000002475 | ENSRNOT00000044222  ENSRNOT00000048912  ENSRNOT00000001383  ENSRNOT00000001195  ENSRNOT00000003348 | AC160125.2  LOC685718  RGD1562068\_predicted    RGD1565798\_predicted | Translationally-controlled tumor protein (TCTP) (Lens epithelial protein). [Source:UniProtKB/Swiss-Prot;Acc:P63029] | 3 |
| 1382540\_at |  | 295607 | ENSRNOG00000004864 | ENSRNOT00000006549 | NP\_001099950.1 | pre-mRNA processing factor 40 homolog A [Source:RefSeq\_peptide;Acc:NP\_001099950] | 3 |
| 1369669\_at | P42676 | 117041 | ENSRNOG00000011561 | ENSRNOT00000048090  ENSRNOT00000015853 | Nln | Neurolysin, mitochondrial precursor (EC 3.4.24.16) (Neurotensin endopeptidase) (Mitochondrial oligopeptidase M) (Microsomal endopeptidase) (MEP). [Source:UniProtKB/Swiss-Prot;Acc:P42676] | 3 |
| 1387327\_at | Q920F3 | 170843 | ENSRNOG00000012284 | ENSRNOT00000016578 | Khdrbs2 | KH domain-containing, RNA-binding, signal transduction-associated protein 2 (Sam68-like mammalian protein 1) (SLM-1) (rSLM-1). [Source:UniProtKB/Swiss-Prot;Acc:Q920F3] | 3 |
| 1388884\_at |  | 291076 | ENSRNOG00000029549 | ENSRNOT00000048923 | RGD1310224 | similar to RIKEN cDNA 1810022C23 (RGD1310224), mRNA [Source:RefSeq\_dna;Acc:NM\_001009275] | 3 |
| 1386154\_at |  | 367235 | ENSRNOG00000013587 | ENSRNOT00000018234 | RGD1304927\_predicted |  | 3 |
| 1385346\_at | Q5HZF2 | 114765 | ENSRNOG00000011678 | ENSRNOT00000015605 | Wbp4 | WW domain-binding protein 4 (WBP-4) (WW domain-containing-binding protein 4) (Formin-binding protein 21). [Source:UniProtKB/Swiss-Prot;Acc:Q5HZF2] | 3 |
| 1394943\_at |  | 293112 | ENSRNOG00000018987 | ENSRNOT00000025653 | RGD1565584\_predicted |  | 3 |
| 1368516\_at | Q03348 | 25167 | ENSRNOG00000021223 | ENSRNOT00000055801  ENSRNOT00000028825  ENSRNOT00000028826 | Ptpra | Receptor-type tyrosine-protein phosphatase alpha precursor (EC 3.1.3.48) (Protein-tyrosine phosphatase alpha) (R-PTP-alpha). [Source:UniProtKB/Swiss-Prot;Acc:Q03348] | 3 |
| 1395139\_at |  | 311478 | ENSRNOG00000004951 | ENSRNOT00000036273 | NP\_001101253.1 | kinesin family member 16B [Source:RefSeq\_peptide;Acc:NP\_001101253] | 3 |
| 1373875\_at |  | 299818 | ENSRNOG00000004316 | ENSRNOT00000005794 | RGD1308261\_predicted |  | 3 |
| 1393458\_s\_at |  | 500030 | ENSRNOG00000005775 | ENSRNOT00000007707 | NP\_001103962.1 | similar to PHD finger protein 14 isoform 1 (predicted) (RGD1563764\_predicted), mRNA [Source:RefSeq\_dna;Acc:NM\_001110492] | 3 |
| 1398811\_at | O88823 | 29439 | ENSRNOG00000016379 | ENSRNOT00000022405 | Jtb | Protein JTB precursor. [Source:UniProtKB/Swiss-Prot;Acc:O88823] | 3 |
| 1390450\_a\_at |  | 291015 | ENSRNOG00000029792 | ENSRNOT00000020532 | NP\_001099573.1 | osteoglycin [Source:RefSeq\_peptide;Acc:NP\_001099573] | 3 |
| 1380265\_at |  | 314228 | ENSRNOG00000009296 | ENSRNOT00000012361 | NP\_001101503.1 | small nuclear RNA activating complex, polypeptide 1 [Source:RefSeq\_peptide;Acc:NP\_001101503] | 3 |
| 1390820\_at |  | 313481 | ENSRNOG00000009720 | ENSRNOT00000033886 | NP\_001101423.1 | zinc finger, CCHC domain containing 11 [Source:RefSeq\_peptide;Acc:NP\_001101423] | 3 |
| 1370895\_at |  | 85250 | ENSRNOG00000003736 | ENSRNOT00000005073 | Col5a2 | Collagen alpha 2 type V (Fragment). [Source:UniProtKB/TrEMBL;Acc:O70598] | 3 |
| 1387126\_at | Q64566 | 170699 | ENSRNOG00000013305 | ENSRNOT00000045087 | Atp2c1 | Calcium-transporting ATPase type 2C member 1 (EC 3.6.3.8) (ATPase 2C1) (ATP-dependent Ca(2+) pump PMR1). [Source:UniProtKB/Swiss-Prot;Acc:Q64566] | 3 |
| 1382446\_at | Q5XIM4 | 362749 | ENSRNOG00000004893 | ENSRNOT00000006509 | Atp5s | ATP synthase subunit s, mitochondrial precursor (ATP synthase-coupling factor B) (Mitochondrial ATP synthase regulatory component factor B). [Source:UniProtKB/Swiss-Prot;Acc:Q5XIM4] | 3 |
| 1373200\_at |  | 291057 | ENSRNOG00000016390 | ENSRNOT00000022065 | NP\_001099576.1 | eukaryotic translation elongation factor 1 epsilon 1 [Source:RefSeq\_peptide;Acc:NP\_001099576] | 3 |
| 1392885\_at |  | 291439 | ENSRNOG00000024104 | ENSRNOT00000058777  ENSRNOT00000032202 | Mbd1 | methyl-CpG binding domain protein 1 [Source:RefSeq\_peptide;Acc:NP\_001011924] | 3 |
| 1394414\_at | Q5M7T4 | 362699 | ENSRNOG00000005610 | ENSRNOT00000007607 | Yipf4 | Protein YIPF4 (YIP1 family member 4). [Source:UniProtKB/Swiss-Prot;Acc:Q5M7T4] | 3 |
| 1367613\_at | Q63716 | 683813  117254 | ENSRNOG00000003941  ENSRNOG00000017194  ENSRNOG00000017695 | ENSRNOT00000038075  ENSRNOT00000023132  ENSRNOT00000023972 | Prdx1 | Peroxiredoxin-1 (EC 1.11.1.15) (Thioredoxin peroxidase 2) (Thioredoxin-dependent peroxide reductase 2) (Heme-binding 23 kDa protein) (HBP23). [Source:UniProtKB/Swiss-Prot;Acc:Q63716] | 3 |
| 1376760\_at |  | 681004  683880 | ENSRNOG00000002036 | ENSRNOT00000002800 | 1810007M14Rik | RIKEN cDNA 1810007M14 gene Gene [Source:MGI Symbol;Acc:MGI:1914617] | 3 |
| 1384112\_at | P21588 | 58813 | ENSRNOG00000011071 | ENSRNOT00000015057 | Nt5e | 5'-nucleotidase precursor (EC 3.1.3.5) (Ecto-5'-nucleotidase) (5'-NT) (CD73 antigen). [Source:UniProtKB/Swiss-Prot;Acc:P21588] | 3 |
| 1372500\_at |  | 300838 | ENSRNOG00000032436 | ENSRNOT00000013852 | Tmod3 | tropomodulin 3 [Source:RefSeq\_peptide;Acc:NP\_001011997] | 3 |
| 1391536\_at |  | 362324 | ENSRNOG00000007924 | ENSRNOT00000010952 | RGD1308432\_predicted |  | 3 |
| 1383309\_at | P61943 | 304023 | ENSRNOG00000001653 | ENSRNOT00000002255 | St3gal6 | Type 2 lactosamine alpha-2,3-sialyltransferase (EC 2.4.99.-) (CMP- NeuAc:beta-galactoside alpha-2,3-sialyltransferase VI) (ST3Gal VI) (Sialyltransferase 10). [Source:UniProtKB/Swiss-Prot;Acc:P61943] | 3 |
| 1367634\_at | P62902 | 287029  680384  678952  690384  686767  289401  683629  64298  688416  291561  299740  298126  681338  682798  502887 | ENSRNOG00000030214  ENSRNOG00000029278  ENSRNOG00000039487  ENSRNOG00000030278  ENSRNOG00000039249  ENSRNOG00000013508  ENSRNOG00000031313  ENSRNOG00000033024  ENSRNOG00000033428  ENSRNOG00000029926  ENSRNOG00000030383  ENSRNOG00000032947  ENSRNOG00000025536  ENSRNOG00000030296  ENSRNOG00000030657 | ENSRNOT00000041674  ENSRNOT00000047850  ENSRNOT00000060519  ENSRNOT00000043590  ENSRNOT00000045401  ENSRNOT00000033733  ENSRNOT00000042660  ENSRNOT00000043610  ENSRNOT00000015408  ENSRNOT00000049211  ENSRNOT00000046725  ENSRNOT00000047517  ENSRNOT00000039731  ENSRNOT00000042885  ENSRNOT00000047507 | RGD1562055\_predicted  LOC680384  LOC690384  RGD1564839\_predicted  RGD1564021\_predicted  LOC683629    RGD1565894\_predicted  RGD1562547\_predicted  RGD1561195\_predicted  LOC682798  RGD1563551\_predicted | 60S ribosomal protein L31. [Source:UniProtKB/Swiss-Prot;Acc:P62902] | 3 |
| 1388182\_at |  | 246327 | ENSRNOG00000031993 | ENSRNOT00000045511 | Prim1 | DNA primase, p49 subunit [Source:RefSeq\_peptide;Acc:NP\_001008768] | 3 |
| 1388917\_at | Q63357 | 25485 | ENSRNOG00000003276 | ENSRNOT00000004609 | Myo1d | Myosin-Id (Myosin heavy chain myr 4). [Source:UniProtKB/Swiss-Prot;Acc:Q63357] | 3 |
| 1388126\_at | O35217 | 499084  29688 | ENSRNOG00000011287 | ENSRNOT00000015017 | Minpp1 | Multiple inositol polyphosphate phosphatase 1 precursor (EC 3.1.3.62) (Inositol (1,3,4,5)-tetrakisphosphate 3-phosphatase) (Ins(1,3,4,5)P(4) 3-phosphatase). [Source:UniProtKB/Swiss-Prot;Acc:O35217] | 3 |
| 1399062\_at |  |  | ENSRNOG00000031995 | ENSRNOT00000047282 | 2810008M24Rik | RIKEN cDNA 2810008M24 gene Gene [Source:MGI Symbol;Acc:MGI:1922866] | 3 |
| 1387459\_at | P27775 | 24678 | ENSRNOG00000000811 | ENSRNOT00000001075  ENSRNOT00000057364  ENSRNOT00000057363 | Pkib | cAMP-dependent protein kinase inhibitor beta (PKI-beta) (cAMP- dependent protein kinase inhibitor, testis isoform). [Source:UniProtKB/Swiss-Prot;Acc:P27775] | 3 |
| 1373194\_at |  | 690354  682893 | ENSRNOG00000024319 | ENSRNOT00000009271 | LOC690354 | Mdn1 protein (Fragment). [Source:UniProtKB/TrEMBL;Acc:Q5RJZ0] | 3 |
| 1377745\_at |  | 310946 | ENSRNOG00000011555 | ENSRNOT00000015434 | LOC310946 | Putative uncharacterized protein LOC310946. [Source:UniProtKB/TrEMBL;Acc:Q3KR61] | 3 |
| 1376788\_at |  | 306722 | ENSRNOG00000018198 | ENSRNOT00000024675 | NP\_001100805.1 | death associated protein kinase 1 [Source:RefSeq\_peptide;Acc:NP\_001100805] | 3 |
| 1390234\_at |  | 84486 | ENSRNOG00000013516 | ENSRNOT00000019126  ENSRNOT00000018478 | Sf3b1 | Spliceosomal protein SAP155 (Fragment). [Source:UniProtKB/TrEMBL;Acc:Q9ET34] | 3 |
| 1389220\_at |  | 682679 | ENSRNOG00000036577 | ENSRNOT00000054682 | LOC682679 |  | 3 |
| 1380553\_at |  | 313085 | ENSRNOG00000009358 | ENSRNOT00000012394 | RGD1306323\_predicted |  | 3 |
| 1372267\_at |  | 296651 | ENSRNOG00000018809 | ENSRNOT00000025433 | NP\_001100039.1 | proteasome (prosome, macropain) 26S subunit, non-ATPase, 5 [Source:RefSeq\_peptide;Acc:NP\_001100039] | 3 |
| 1388372\_at | P17078 | 681918  686074  296709  301725 | ENSRNOG00000030364  ENSRNOG00000014272  ENSRNOG00000038279 | ENSRNOT00000051911  ENSRNOT00000019162  ENSRNOT00000058267 | LOC686074  LOC301725 | 60S ribosomal protein L35. [Source:UniProtKB/Swiss-Prot;Acc:P17078] | 3 |
| 1371761\_at | P11250 | 679499  680170  684829  362041  689061 | ENSRNOG00000038045  ENSRNOG00000016387 | ENSRNOT00000057895  ENSRNOT00000009046 | LOC684829  RGD1561086\_predicted | ribosomal protein L34 [Source:RefSeq\_peptide;Acc:NP\_001102037]  60S ribosomal protein L34. [Source:UniProtKB/Swiss-Prot;Acc:P11250] | 3 |
| 1396413\_at | Q9ERU2 | 360389 | ENSRNOG00000013379 | ENSRNOT00000018050 | Zfp422 | Zinc finger protein 22 (Krueppel-type zinc finger protein Krox-25). [Source:UniProtKB/Swiss-Prot;Acc:Q9ERU2] | 3 |
| 1371774\_at |  | 302642 | ENSRNOG00000003809 | ENSRNOT00000043241 | Sat | spermidine/spermine N1-acetyl transferase [Source:RefSeq\_peptide;Acc:NP\_001007668] | 3 |
| 1370333\_a\_at | P08025 | 24482 | ENSRNOG00000004517 | ENSRNOT00000005995  ENSRNOT00000004136  ENSRNOT00000006103 | Igf1 | Insulin-like growth factor I precursor (IGF-I) (Somatomedin). [Source:UniProtKB/Swiss-Prot;Acc:P08025] | 3 |
| 1374581\_at |  | 689741  362912 | ENSRNOG00000005551 | ENSRNOT00000007484 | RGD1311835 | similar to RIKEN cDNA 1110021N07 (RGD1311835), mRNA [Source:RefSeq\_dna;Acc:NM\_001014202] | 3 |
| 1396116\_at |  |  | ENSRNOG00000004716 | ENSRNOT00000041995  ENSRNOT00000029880 | Sdccag1 | serologically defined colon cancer antigen 1 Gene [Source:MGI Symbol;Acc:MGI:1918305] | 3 |
| 1389231\_at |  |  | ENSRNOG00000006357 | ENSRNOT00000050999 | RGD1564957\_predicted |  | 3 |
| 1387071\_a\_at | P19332 | 29477 | ENSRNOG00000005133 | ENSRNOT00000042984  ENSRNOT00000045127  ENSRNOT00000006856  ENSRNOT00000050070  ENSRNOT00000043604  ENSRNOT00000045134  ENSRNOT00000006947 | Mapt | Microtubule-associated protein tau (Neurofibrillary tangle protein) (Paired helical filament-tau) (PHF-tau). [Source:UniProtKB/Swiss-Prot;Acc:P19332] | 3 |
| 1378405\_at | Q5XIU2 | 361627 | ENSRNOG00000010087 | ENSRNOT00000013733 | Zfp143 | Zinc finger protein 143 (Zfp-143). [Source:UniProtKB/Swiss-Prot;Acc:Q5XIU2] | 3 |
| 1378524\_at |  | 362900 | ENSRNOG00000009658 | ENSRNOT00000013344 | Rnf19\_predicted |  | 3 |
| 1378096\_at | Q2PQA9 | 117550 | ENSRNOG00000017466 | ENSRNOT00000023861 | Kif5b | Kinesin heavy chain (Ubiquitous kinesin heavy chain) (UKHC). [Source:UniProtKB/Swiss-Prot;Acc:Q2PQA9] | 3 |
| 1373138\_at | Q6AY63 | 361274 | ENSRNOG00000017741 | ENSRNOT00000023892 | Nudt5 | ADP-sugar pyrophosphatase (EC 3.6.1.13) (EC 3.6.1.-) (Nucleoside diphosphate-linked moiety X motif 5) (Nudix motif 5). [Source:UniProtKB/Swiss-Prot;Acc:Q6AY63] | 3 |
| 1390201\_at | P62836 | 295347 | ENSRNOG00000032463 | ENSRNOT00000047836 | Rap1a | Ras-related protein Rap-1A precursor (Ras-related protein Krev-1). [Source:UniProtKB/Swiss-Prot;Acc:P62836] | 3 |
| 1374178\_at | P51646 | 117050 | ENSRNOG00000006839 | ENSRNOT00000009181 | Arl5a | ADP-ribosylation factor-like protein 5A. [Source:UniProtKB/Swiss-Prot;Acc:P51646] | 3 |
| 1367631\_at | Q9R1E9 | 64032 | ENSRNOG00000015036 | ENSRNOT00000020528 | Ctgf | Connective tissue growth factor precursor (Connective tissue growth- related protein). [Source:UniProtKB/Swiss-Prot;Acc:Q9R1E9] | 3 |
| 1370939\_at | P18163 | 25288 | ENSRNOG00000010633 | ENSRNOT00000014235 | Acsl1 | Long-chain-fatty-acid--CoA ligase 1 (EC 6.2.1.3) (Long-chain acyl-CoA synthetase 1) (LACS 1) (Long-chain-fatty-acid--CoA ligase, liver isozyme). [Source:UniProtKB/Swiss-Prot;Acc:P18163] | 3 |
| 1384465\_at | Q4KLH4 | 305910 | ENSRNOG00000020782 | ENSRNOT00000028219 | Pspc1 | Paraspeckle component 1. [Source:UniProtKB/Swiss-Prot;Acc:Q4KLH4] | 3 |
| 1385470\_at |  |  | ENSRNOG00000005292 | ENSRNOT00000048536 | LOC314393 |  | 3 |
| 1377662\_at | Q5M827 | 363465 | ENSRNOG00000003674 | ENSRNOT00000004884 | Pir | Pirin. [Source:UniProtKB/Swiss-Prot;Acc:Q5M827] | 3 |
| 1385024\_at |  | 361760 | ENSRNOG00000017298 | ENSRNOT00000023396 | NP\_001101995.1 | cutC copper transporter homolog [Source:RefSeq\_peptide;Acc:NP\_001101995] | 3 |
| 1379298\_at |  | 288264 | ENSRNOG00000028594 | ENSRNOT00000029985 | NP\_001099363.1 | interferon (alpha and beta) receptor 1 [Source:RefSeq\_peptide;Acc:NP\_001099363] | 3 |
| 1384282\_at |  | 304342 | ENSRNOG00000039234 | ENSRNOT00000001810 | Zipro1 | zinc finger proliferation 1 [Source:RefSeq\_peptide;Acc:NP\_001012021] | 3 |
| 1390802\_at |  | 293888 | ENSRNOG00000015932 | ENSRNOT00000021544 | NP\_001099817.1 |  | 3 |
| 1396696\_at | P19493 | 29629 | ENSRNOG00000006957 | ENSRNOT00000009542 | Gria4 | Glutamate receptor 4 precursor (GluR-4) (GluR4) (GluR-D) (Glutamate receptor ionotropic, AMPA 4) (AMPA-selective glutamate receptor 4). [Source:UniProtKB/Swiss-Prot;Acc:P19493] | 3 |
| 1374842\_at |  | 362686 | ENSRNOG00000005087 | ENSRNOT00000006824 | NP\_001102171.1 | CCAAT/enhancer binding protein zeta [Source:RefSeq\_peptide;Acc:NP\_001102171] | 3 |
| 1376026\_at |  |  | ENSRNOG00000002012 | ENSRNOT00000002756 | Donson | downstream neighbor of SON [Source:RefSeq\_peptide;Acc:NP\_001008288] | 3 |
| 1383911\_at |  |  | ENSRNOG00000006644 | ENSRNOT00000009083 | Jmjd2c | jumonji domain containing 2C Gene [Source:MGI (curated);Acc:Jmjd2c-006] | 3 |
| 1377701\_at |  | 362602 | ENSRNOG00000013865 | ENSRNOT00000019135 | NP\_001102151.1 | similar to RIKEN cDNA 9330177P20 (predicted) (RGD1561833\_predicted), mRNA [Source:RefSeq\_dna;Acc:NM\_001108681] | 3 |
| 1369662\_at | P04775 | 24766 | ENSRNOG00000005018 | ENSRNOT00000007069  ENSRNOT00000045323 | Scn2a1 | Sodium channel protein type 2 subunit alpha (Sodium channel protein type II subunit alpha) (Voltage-gated sodium channel subunit alpha Nav1.2) (Sodium channel protein, brain II subunit alpha). [Source:UniProtKB/Swiss-Prot;Acc:P04775] | 3 |
| 1374397\_at |  | 296302 | ENSRNOG00000017447 | ENSRNOT00000023786 | Eif2s2 | eukaryotic translation initiation factor 2, subunit 2 beta, 38kDa [Source:RefSeq\_peptide;Acc:NP\_955412] | 3 |
| 1373961\_at | Q66H33 | 304176 | ENSRNOG00000000720  ENSRNOG00000038965 | ENSRNOT00000000911  ENSRNOT00000015146 | CC038\_RAT  MGC95208 | Uncharacterized protein C3orf38 homolog. [Source:UniProtKB/Swiss-Prot;Acc:Q66H33]  similar to 4930453N24Rik protein (MGC95208), mRNA [Source:RefSeq\_dna;Acc:NM\_001005552] | 3 |
| 1389184\_at |  | 685332  687772 | ENSRNOG00000018718 | ENSRNOT00000025412 | LOC687772 |  | 3 |
| 1374067\_at |  |  | ENSRNOG00000009954 | ENSRNOT00000046582  ENSRNOT00000013289 | RGD1308127 | Uncharacterized protein C10orf46 homolog. [Source:UniProtKB/Swiss-Prot;Acc:Q5XI53] | 3 |
| 1367637\_a\_at | P63088 | 24669 | ENSRNOG00000001269 | ENSRNOT00000048851  ENSRNOT00000001711 | Ppp1cc | Serine/threonine-protein phosphatase PP1-gamma catalytic subunit (EC 3.1.3.16) (PP-1G) (Protein phosphatase 1C catalytic subunit). [Source:UniProtKB/Swiss-Prot;Acc:P63088] | 3 |
| 1385595\_at |  | 683353  689197 | ENSRNOG00000026690 | ENSRNOT00000039689 | LOC683353 | similar to antigenic determinant of rec-A protein (LOC689197), mRNA [Source:RefSeq\_dna;Acc:NM\_001109529] | 3 |
| 1388132\_at |  | 252855 | ENSRNOG00000013154 | ENSRNOT00000018049 | Sfpq | NonO/p54nrb homolog [Source:RefSeq\_peptide;Acc:NP\_001020442] | 3 |
| 1398906\_at |  | 501550 | ENSRNOG00000004419 | ENSRNOT00000006006 | RGD1306925 | trafficking protein particle complex protein 2 [Source:RefSeq\_peptide;Acc:NP\_001020136] | 3 |
| 1389302\_at | Q5BJY3 | 291737 | ENSRNOG00000016532 | ENSRNOT00000022169 | RGD1310199 | Uncharacterized protein C18orf37 homolog. [Source:UniProtKB/Swiss-Prot;Acc:Q5BJY3] | 3 |
| 1381299\_at |  | 680591 | ENSRNOG00000029582 | ENSRNOT00000045463 | LOC680591 |  | 3 |
| 1379075\_at | Q3T1J2 |  | ENSRNOG00000006777 | ENSRNOT00000009018 | MBOA2\_RAT | Membrane-bound O-acyltransferase domain-containing protein 2 (EC 2.3.-.-) (O-acyltransferase domain-containing protein 2). [Source:UniProtKB/Swiss-Prot;Acc:Q3T1J2] | 3 |
| 1368330\_at | Q9QYW0 | 114512 | ENSRNOG00000002778 | ENSRNOT00000000258 | Aatf | Protein AATF (Apoptosis-antagonizing transcription factor). [Source:UniProtKB/Swiss-Prot;Acc:Q9QYW0] | 3 |
| 1369954\_at | P41562 | 24479 | ENSRNOG00000015020 | ENSRNOT00000020322  ENSRNOT00000044302 | Idh1 | Isocitrate dehydrogenase [NADP] cytoplasmic (EC 1.1.1.42) (Cytosolic NADP-isocitrate dehydrogenase) (Oxalosuccinate decarboxylase) (IDH) (NADP(+)-specific ICDH) (IDP). [Source:UniProtKB/Swiss-Prot;Acc:P41562] | 3 |
| 1371398\_at |  | 690441  684567  687430  689271 | ENSRNOG00000027049  ENSRNOG00000000943 | ENSRNOT00000033537  ENSRNOT00000060939  ENSRNOT00000001251 | LOC690441  LOC689271 |  | 3 |
| 1382290\_at |  | 361664 | ENSRNOG00000017137 | ENSRNOT00000023121 | NP\_001101974.1 | similar to CG9643-PA (predicted) (RGD1306300\_predicted), mRNA [Source:RefSeq\_dna;Acc:NM\_001108504] | 3 |
| 1399157\_at |  | 308537 | ENSRNOG00000014463 | ENSRNOT00000019463 | NP\_001100977.1 | similar to NNX3 (predicted) (RGD1310358\_predicted), mRNA [Source:RefSeq\_dna;Acc:NM\_001107507] | 3 |
| 1388725\_at | Q9JLS8 | 56766 | ENSRNOG00000037992 | ENSRNOT00000007859 | Leprot | Leptin receptor gene-related protein (OB-R gene-related protein) (OB- RGRP). [Source:UniProtKB/Swiss-Prot;Acc:Q9JLS8] | 3 |
| 1368279\_at |  | 114510 | ENSRNOG00000011280 | ENSRNOT00000015094  ENSRNOT00000015388 | Mllt3 | myeloid/lymphoid or mixed-lineage leukemia, translocated to, 3 [Source:RefSeq\_peptide;Acc:NP\_446170] | 3 |
| 1374812\_at |  | 498331 | ENSRNOG00000002061 | ENSRNOT00000061162 | LOC498331 | Protein tyrosine phosphatase (Fragment). [Source:UniProtKB/TrEMBL;Acc:Q99ND7] | 3 |
| 1385497\_x\_at |  | 360926 | ENSRNOG00000002272 | ENSRNOT00000003088 | NP\_001101828.1 | ligand of numb-protein X 1 [Source:RefSeq\_peptide;Acc:NP\_001101828] | 3 |
| 1392399\_at |  | 362902 | ENSRNOG00000004301 | ENSRNOT00000005803 | Wdsof1\_predicted |  | 3 |
| 1391393\_at |  | 361585 | ENSRNOG00000015734 | ENSRNOT00000021366 | Ube3a\_predicted |  | 3 |
| 1368211\_at | P13471 | 29284  687947  679683  691255  687373 | ENSRNOG00000018774  ENSRNOG00000031585  ENSRNOG00000025122 | ENSRNOT00000025655  ENSRNOT00000059501  ENSRNOT00000042949  ENSRNOT00000036330 | Rps14  LOC687947  LOC691255 | 40S ribosomal protein S14. [Source:UniProtKB/Swiss-Prot;Acc:P13471] | 3 |
| 1396385\_at |  | 595134 | ENSRNOG00000026610 | ENSRNOT00000034944 | LOC595134 |  | 3 |
| 1372571\_at | Q5I0I2 | 362849 | ENSRNOG00000007769 | ENSRNOT00000010399  ENSRNOT00000059979 | March2 | E3 ubiquitin-protein ligase MARCH2 (EC 6.3.2.-) (Membrane-associated RING finger protein 2) (Membrane-associated RING-CH protein II) (MARCH-II). [Source:UniProtKB/Swiss-Prot;Acc:Q5I0I2] | 3 |
| 1368888\_a\_at | Q9JK11 | 83765 | ENSRNOG00000004621 | ENSRNOT00000006443  ENSRNOT00000041638  ENSRNOT00000006957 | Rtn4 | Reticulon-4 (Neurite outgrowth inhibitor) (Nogo protein) (Foocen) (Glut4 vesicle 20 kDa protein). [Source:UniProtKB/Swiss-Prot;Acc:Q9JK11] | 3 |
| 1372101\_at | P97544 | 192270 | ENSRNOG00000008116 | ENSRNOT00000011237 | Ppap2b | Lipid phosphate phosphohydrolase 3 (EC 3.1.3.4) (Phosphatidic acid phosphatase 2b) (Phosphatidate phosphohydrolase type 2b) (PAP2b) (PAP- 2b) (PAP2-beta) (Differentially expressed in rat intestine 42) (Dri42). [Source:UniProtKB/Swiss-Prot;Acc:P97544] | 3 |
| 1388813\_at | P84082 | 79119 | ENSRNOG00000004807 | ENSRNOT00000006483 | Arf2 | ADP-ribosylation factor 2. [Source:UniProtKB/Swiss-Prot;Acc:P84082] | 3 |
| 1382192\_at |  | 293186 | ENSRNOG00000026902 | ENSRNOT00000030474 | NP\_001099756.1 | extracellular link domain-containing 1 [Source:RefSeq\_peptide;Acc:NP\_001099756] | 3 |
| 1394200\_at | P14659 | 60460 | ENSRNOG00000006472 | ENSRNOT00000008504 | Hspa2 | Heat shock-related 70 kDa protein 2 (Heat shock protein 70.2) (Testis- specific heat shock protein-related) (HST). [Source:UniProtKB/Swiss-Prot;Acc:P14659] | 3 |
| 1371429\_at |  | 114489 | ENSRNOG00000019400 | ENSRNOT00000026327 | Dag1 | Dystroglycan 1 (Fragment). [Source:UniProtKB/TrEMBL;Acc:Q91XP6] | 3 |
| 1371489\_at | O88846 | 29274 | ENSRNOG00000013930 | ENSRNOT00000019555 | Rnf4 | RING finger protein 4 (SNURF). [Source:UniProtKB/Swiss-Prot;Acc:O88846] | 3 |
| 1373064\_at |  | 290370 | ENSRNOG00000009063 | ENSRNOT00000012530 | NP\_001099520.1 | DnaJ (Hsp40) homolog, subfamily C, member 15 [Source:RefSeq\_peptide;Acc:NP\_001099520] | 3 |
| 1393256\_at |  | 291314 | ENSRNOG00000018125 | ENSRNOT00000024697 | Sephs1 | selenophosphate synthetase 1 [Source:RefSeq\_peptide;Acc:NP\_001098100] | 3 |
| 1390130\_at | Q9WVC7 | 64553 | ENSRNOG00000004841 | ENSRNOT00000006562 | Akap6 | A-kinase anchor protein 6 (Protein kinase A-anchoring protein 6) (PRKA6) (mAKAP). [Source:UniProtKB/Swiss-Prot;Acc:Q9WVC7] | 3 |
| 1389229\_at | Q66H78 | 315939 | ENSRNOG00000012480 | ENSRNOT00000017085 | ACPL2\_RAT | Acid phosphatase-like protein 2 precursor (EC 3.1.3.2). [Source:UniProtKB/Swiss-Prot;Acc:Q66H78] | 3 |
| 1388788\_at |  | 364975 | ENSRNOG00000003307 | ENSRNOT00000004570 | NP\_001102366.1 | glutaryl-Coenzyme A dehydrogenase [Source:RefSeq\_peptide;Acc:NP\_001102366] | 3 |
| 1372619\_at |  | 309176 | ENSRNOG00000020975 | ENSRNOT00000028475 | Mrpl49 | mitochondrial ribosomal protein L49 [Source:RefSeq\_peptide;Acc:NP\_001041348] | 3 |
| 1372065\_at |  | 305235 | ENSRNOG00000002256 | ENSRNOT00000052035  ENSRNOT00000060712  ENSRNOT00000003071 | Art3 | ADP-ribosyltransferase 3 [Source:RefSeq\_peptide;Acc:NP\_001012034] | 3 |
| 1373143\_at |  | 316982 | ENSRNOG00000036913 | ENSRNOT00000055437 | RGD1309621 | RGD1309621 protein (Fragment). [Source:UniProtKB/TrEMBL;Acc:Q5RJS5] | 3 |
| 1382417\_at |  | 304798 | ENSRNOG00000009696 | ENSRNOT00000012984 | Mdm4 | mouse double minute 4 homolog [Source:RefSeq\_peptide;Acc:NP\_001012026] | 3 |
| 1378007\_at |  |  | ENSRNOG00000025076 | ENSRNOT00000019959 | RGD1306148\_predicted |  | 3 |
| 1373206\_at |  | 294925 | ENSRNOG00000024089 | ENSRNOT00000034096 | Fndc3b\_predicted |  | 3 |
| 1378536\_at |  | 313370 | ENSRNOG00000026226 | ENSRNOT00000012396 | NP\_001101416.1 | hook homolog 1 [Source:RefSeq\_peptide;Acc:NP\_001101416] | 3 |
| 1385226\_at |  | 363634 | ENSRNOG00000015669 | ENSRNOT00000020970 | NP\_001102301.1 | potassium channel tetramerisation domain containing 11 [Source:RefSeq\_peptide;Acc:NP\_001102301] | 3 |
| 1381924\_at |  | 292078 | ENSRNOG00000038239 | ENSRNOT00000058216 | NP\_001099665.1 |  | 3 |
| 1386952\_a\_at | Q62871 | 116659 | ENSRNOG00000009781 | ENSRNOT00000047243 | Dncic2 | Cytoplasmic dynein 1 intermediate chain 2 (Dynein intermediate chain 2, cytosolic) (DH IC-2) (Cytoplasmic dynein intermediate chain 2). [Source:UniProtKB/Swiss-Prot;Acc:Q62871] | 3 |
| 1388180\_at | Q63068 | 286917 | ENSRNOG00000014459 | ENSRNOT00000019627 | Phax | RNA U small nuclear RNA export adapter protein (Phosphorylated adapter RNA export protein) (Resiniferatoxin-binding protein 2) (RBP-2) (26 kDa resiniferatoxin-binding protein) (RBP-26) (RTX-42). [Source:UniProtKB/Swiss-Prot;Acc:Q63068] | 3 |
| 1384846\_at |  | 682900  679610 | ENSRNOG00000006114 | ENSRNOT00000009264 | A330021E22Rik | RIKEN cDNA A330021E22 gene Gene [Source:MGI Symbol;Acc:MGI:2443778] | 3 |
| 1367568\_a\_at | P08494 | 25333 | ENSRNOG00000005695 | ENSRNOT00000007577 | Mgp | Matrix Gla protein precursor (MGP). [Source:UniProtKB/Swiss-Prot;Acc:P08494] | 3 |
| 1374224\_at |  | 114859 | ENSRNOG00000006027 | ENSRNOT00000009222  ENSRNOT00000056469 | NP\_001099214.1 | eukaryotic translation initiation factor 2 alpha kinase 4 (predicted) [Source:RefSeq\_peptide;Acc:NP\_001099214] | 3 |
| 1385492\_at |  | 307206 | ENSRNOG00000014006 | ENSRNOT00000019384 | NP\_001100841.1 | similar to Neuropilin- and tolloid-like protein 1 (predicted) (RGD1566269\_predicted), mRNA [Source:RefSeq\_dna;Acc:NM\_001107371] | 3 |
| 1388142\_at |  | 114122 | ENSRNOG00000029212 | ENSRNOT00000045532 | Cspg2 | Versican core protein precursor (Large fibroblast proteoglycan) (Chondroitin sulfate proteoglycan core protein 2) (PG-M) (Glial hyaluronate-binding protein) (GHAP) (Fragments). [Source:UniProtKB/Swiss-Prot;Acc:Q9ERB4] | 3 |
| 1391040\_at |  | 498249  291787 | ENSRNOG00000012899 | ENSRNOT00000017291 | RGD1308872\_predicted |  | 3 |
| 1368225\_at |  |  | ENSRNOG00000033643 | ENSRNOT00000040371 | Exoc2 | Exocyst complex component 2 (Exocyst complex component Sec5) (rSec5). [Source:UniProtKB/Swiss-Prot;Acc:O54921] | 3 |
| 1372453\_at | Q5XI68 | 289881 | ENSRNOG00000000070 | ENSRNOT00000000080 | Dr1 | TATA-binding protein-associated phosphoprotein (Down-regulator of transcription 1) (Negative cofactor 2 beta) (NC2 beta). [Source:UniProtKB/Swiss-Prot;Acc:Q5XI68] | 3 |
| 1377118\_at | Q3B7T8 | 290722 | ENSRNOG00000010566 | ENSRNOT00000014083 | RGD1308517 | Uncharacterized protein KIAA1712 homolog. [Source:UniProtKB/Swiss-Prot;Acc:Q3B7T8] | 3 |
| 1388709\_at |  | 362703 | ENSRNOG00000026316 | ENSRNOT00000035320 | LOC362703 | LOC362703 protein. [Source:UniProtKB/TrEMBL;Acc:Q4KLJ6] | 3 |
| 1391628\_at |  | 361295 | ENSRNOG00000016800 | ENSRNOT00000041836  ENSRNOT00000032454 | Ss18 | Ss18 protein (Fragment). [Source:UniProtKB/TrEMBL;Acc:Q5XI66] | 3 |
| 1376664\_at |  | 246187 | ENSRNOG00000020049 | ENSRNOT00000027200 | LOC246187 | Metalloproteinase (Fragment). [Source:UniProtKB/TrEMBL;Acc:Q8CIV0] | 3 |
| 1389299\_at | P19836 | 140544 | ENSRNOG00000001762 | ENSRNOT00000002403 | Pcyt1a | Choline-phosphate cytidylyltransferase A (EC 2.7.7.15) (Phosphorylcholine transferase A) (CTP:phosphocholine cytidylyltransferase A) (CT A) (CCT A) (CCT-alpha). [Source:UniProtKB/Swiss-Prot;Acc:P19836] | 3 |
| 1394347\_at |  | 685004  687154  501069 | ENSRNOG00000029910 | ENSRNOT00000015113  ENSRNOT00000051290 | LOC501069 | LOC501069 protein (Fragment). [Source:UniProtKB/TrEMBL;Acc:Q5U4E6] | 3 |
| 1374105\_at | Q8VH49 | 140937 | ENSRNOG00000019428 | ENSRNOT00000026328 | Higd1a | HIG1 domain family member 1A (Hypoxia-inducible gene 1 protein). [Source:UniProtKB/Swiss-Prot;Acc:Q8VH49] | 3 |
| 1398395\_at |  | 298914 | ENSRNOG00000006358 | ENSRNOT00000009401 | NP\_001100189.1 | integrin beta 1 binding protein 1 [Source:RefSeq\_peptide;Acc:NP\_001100189] | 3 |
| 1378606\_at |  | 291694 | ENSRNOG00000020384 | ENSRNOT00000027663 | NP\_001099628.1 |  | 3 |
| 1393559\_at | P97574 | 81801 | ENSRNOG00000015075 | ENSRNOT00000020728 | Stc1 | Stanniocalcin-1 precursor (STC-1). [Source:UniProtKB/Swiss-Prot;Acc:P97574] | 3 |
| 1388947\_at |  |  | ENSRNOG00000023356 | ENSRNOT00000035785 | Eif5b | eukaryotic translation initiation factor 5B [Source:RefSeq\_peptide;Acc:NP\_001103611] | 3 |
| 1386915\_at | Q9EST6 | 170724 | ENSRNOG00000009266 | ENSRNOT00000060043 | Anp32b | Acidic leucine-rich nuclear phosphoprotein 32 family member B (Proliferation-related acidic leucine-rich protein PAL31). [Source:UniProtKB/Swiss-Prot;Acc:Q9EST6] | 3 |
| 1374154\_at |  |  | ENSRNOG00000005263 | ENSRNOT00000034989 | LOC312030 |  | 3 |
| 1372333\_at |  | 360844  685587  687315 | ENSRNOG00000034027  ENSRNOG00000031127  ENSRNOG00000034206 | ENSRNOT00000043934  ENSRNOT00000042614  ENSRNOT00000040164 | Snrpe\_predicted  LOC687315 |  | 3 |
| 1377622\_at |  | 308839 | ENSRNOG00000024194 | ENSRNOT00000037050 | Hbxap\_predicted |  | 3 |
| 1375932\_at | P09330 | 24689 | ENSRNOG00000004160 | ENSRNOT00000005615 | Prps2 | Ribose-phosphate pyrophosphokinase 2 (EC 2.7.6.1) (Phosphoribosyl pyrophosphate synthetase II) (PRS-II). [Source:UniProtKB/Swiss-Prot;Acc:P09330] | 3 |
| 1370166\_at | P34900 | 25615 | ENSRNOG00000004936 | ENSRNOT00000007255 | Sdc2 | Syndecan-2 precursor (SYND2) (Fibroglycan) (Heparan sulfate proteoglycan core protein) (HSPG). [Source:UniProtKB/Swiss-Prot;Acc:P34900] | 3 |
| 1372865\_at |  | 362002 | ENSRNOG00000000098 | ENSRNOT00000000110 | NP\_001102030.1 | zinc finger protein 364 [Source:RefSeq\_peptide;Acc:NP\_001102030] | 3 |
| 1389384\_at |  | 362495 | ENSRNOG00000007506 | ENSRNOT00000010073 | Hrpap20 | hormone-regulated proliferation associated protein 20 [Source:RefSeq\_peptide;Acc:NP\_942078] | 3 |
| 1380815\_at |  | 304923 | ENSRNOG00000003861 | ENSRNOT00000005148 | LOC304923 | LOC304923 protein (Fragment). [Source:UniProtKB/TrEMBL;Acc:Q5RK15] | 3 |
| 1384548\_at | P62912 | 315521  680959  682072  683110  688684  28298 | ENSRNOG00000028869  ENSRNOG00000030346  ENSRNOG00000008516  ENSRNOG00000032605  ENSRNOG00000010746  ENSRNOG00000026921 | ENSRNOT00000048460  ENSRNOT00000045382  ENSRNOT00000011244  ENSRNOT00000046799  ENSRNOT00000014493  ENSRNOT00000036031 | RGD1563958\_predicted  LOC682072  LOC688684  Rpl32 | 60S ribosomal protein L32. [Source:UniProtKB/Swiss-Prot;Acc:P62912] | 3 |
| 1379967\_at | Q5U2Z0 | 306695 | ENSRNOG00000027234 | ENSRNOT00000036287 | Zfp367 | Zinc finger protein 367. [Source:UniProtKB/Swiss-Prot;Acc:Q5U2Z0] | 3 |
| 1373090\_at |  | 361233 | ENSRNOG00000014165 | ENSRNOT00000060226  ENSRNOT00000019040 | Ssr1 | Translocon-associated protein subunit alpha precursor (TRAP-alpha) (Signal sequence receptor subunit alpha) (SSR-alpha) (Liver regeneration-related protein LRRG137). [Source:UniProtKB/Swiss-Prot;Acc:Q7TPJ0] | 3 |
| 1393689\_at |  | 296086 | ENSRNOG00000005006 | ENSRNOT00000006874 | NP\_001099970.1 | NADH dehydrogenase (ubiquinone) 1 alpha subcomplex, assembly factor 1 [Source:RefSeq\_peptide;Acc:NP\_001099970] | 3 |
| 1388941\_at | Q400C7 | 360650 | ENSRNOG00000000246 | ENSRNOT00000000263 | RGD1304846 | Archaemetzincin-2 (EC 3.-.-.-) (Archeobacterial metalloproteinase-like protein 2). [Source:UniProtKB/Swiss-Prot;Acc:Q400C7] | 3 |
| 1382353\_at |  | 686562  690450 | ENSRNOG00000004506 | ENSRNOT00000006187 | LOC690450 |  | 3 |
| 1386261\_x\_at |  | 363425 | ENSRNOG00000006411 | ENSRNOT00000008722 | Cav2 | caveolin 2 [Source:RefSeq\_peptide;Acc:NP\_571989] | 3 |
| 1368487\_at | P29524 | 60325 | ENSRNOG00000002460 | ENSRNOT00000003409 | Serpinb2 | Plasminogen activator inhibitor 2 type A (PAI2A) (PAI-2). [Source:UniProtKB/Swiss-Prot;Acc:P29524] | 3 |
| 1384944\_at |  |  | ENSRNOG00000005776 | ENSRNOT00000008589 | NP\_001101527.1 | B-cell leukemia/lymphoma 11B [Source:RefSeq\_peptide;Acc:NP\_001101527] | 3 |
| 1377902\_a\_at |  | 297561 | ENSRNOG00000009742 | ENSRNOT00000013070 | NP\_001100087.2 | RAD52 homolog [Source:RefSeq\_peptide;Acc:NP\_001100087] | 3 |
| 1395142\_at |  |  | ENSRNOG00000021589 | ENSRNOT00000057605 | RGD1561931\_predicted |  | 3 |
| 1385804\_x\_at |  | 361305  688637 | ENSRNOG00000027355 | ENSRNOT00000031530 | Wdr36\_predicted |  | 3 |
| 1392033\_a\_at |  |  | ENSRNOG00000025286 | ENSRNOT00000033554  ENSRNOT00000055736 | Pank2 | pantothenate kinase 2 (Hallervorden-Spatz syndrome) Gene [Source:MGI (curated);Acc:Pank2-001] | 3 |
| 1371380\_at | P26284 | 29554 | ENSRNOG00000025383 | ENSRNOT00000038352 | Pdha1 | Pyruvate dehydrogenase E1 component subunit alpha, somatic form, mitochondrial precursor (EC 1.2.4.1) (PDHE1-A type I). [Source:UniProtKB/Swiss-Prot;Acc:P26284] | 3 |
| 1390786\_at |  | 310635 | ENSRNOG00000020027 | ENSRNOT00000027182 | Arhgef2 | rho/rac guanine nucleotide exchange factor (GEF) 2 [Source:RefSeq\_peptide;Acc:NP\_001012079] | 3 |
| 1399043\_at | Q3T1K5 | 493810 | ENSRNOG00000028549 | ENSRNOT00000039017 | Capza2 | F-actin-capping protein subunit alpha-2 (CapZ alpha-2). [Source:UniProtKB/Swiss-Prot;Acc:Q3T1K5] | 3 |
| 1394591\_at |  |  | ENSRNOG00000000236 | ENSRNOT00000000252 | Zfp207 | zinc finger protein 207 [Source:RefSeq\_peptide;Acc:NP\_001034109] | 3 |
| 1399160\_a\_at | P62839  P61078 | 81920  641452 | ENSRNOG00000013741 | ENSRNOT00000051335 | Ube2d3 | Ubiquitin-conjugating enzyme E2 D3 (EC 6.3.2.19) (Ubiquitin-protein ligase D3) (Ubiquitin carrier protein D3) (Ubiquitin-conjugating enzyme E2-17 kDa 3) (E2(17)KB 3) (Phosphoarginine phosphatase) (PAPase). [Source:UniProtKB/Swiss-Prot;Acc:P61078] | 3 |
| 1374947\_at |  | 310838 | ENSRNOG00000013737 | ENSRNOT00000018417 | NP\_001101192.1 | breast cancer anti-estrogen resistance 3 [Source:RefSeq\_peptide;Acc:NP\_001101192] | 3 |
| 1369105\_a\_at | P27775 | 24678 | ENSRNOG00000000811 | ENSRNOT00000001075  ENSRNOT00000057364  ENSRNOT00000057363 | Pkib | cAMP-dependent protein kinase inhibitor beta (PKI-beta) (cAMP- dependent protein kinase inhibitor, testis isoform). [Source:UniProtKB/Swiss-Prot;Acc:P27775] | 3 |
| 1367612\_at | P08011 | 171341 | ENSRNOG00000007743 | ENSRNOT00000010579 | Mgst1 | Microsomal glutathione S-transferase 1 (EC 2.5.1.18) (Microsomal GST- 1) (Microsomal GST-I). [Source:UniProtKB/Swiss-Prot;Acc:P08011] | 3 |
| 1372458\_at |  |  | ENSRNOG00000001062 | ENSRNOT00000001407 |  |  | 3 |
| 1372812\_at |  | 296115 | ENSRNOG00000008629 | ENSRNOT00000011551 | RGD1559930\_predicted |  | 3 |
| 1373266\_at |  |  | ENSRNOG00000033261 | ENSRNOT00000061158 | RGD1306327 | similar to downregulated in renal cell carcinoma [Source:RefSeq\_peptide;Acc:NP\_001020300] | 3 |
| 1379242\_at |  | 678905  684996 | ENSRNOG00000013806 | ENSRNOT00000018746 | LOC684996 |  | 3 |
| 1371573\_at | P83883 | 308353  365560  501876  292964  682278  680395  687717  81769  499622  363636  365800  691991  365368  687721  687726 | ENSRNOG00000031844  ENSRNOG00000032062  ENSRNOG00000031315  ENSRNOG00000029944  ENSRNOG00000011494  ENSRNOG00000032408  ENSRNOG00000019170 | ENSRNOT00000039948  ENSRNOT00000043603  ENSRNOT00000043171  ENSRNOT00000052106  ENSRNOT00000015293  ENSRNOT00000057222  ENSRNOT00000047773  ENSRNOT00000025950 | AC102235.10  RGD1564617\_predicted    RGD1563431\_predicted  LOC682278  RGD1561420\_predicted | 60S ribosomal protein L36a (60S ribosomal protein L44). [Source:UniProtKB/Swiss-Prot;Acc:P83883] | 3 |
| 1372223\_at |  | 303010  682617  685957 | ENSRNOG00000033169 | ENSRNOT00000043415 | LOC685957 | cytoplasmic polyadenylation element binding protein 4 [Source:RefSeq\_peptide;Acc:NP\_001100462] | 3 |
| 1374647\_at |  | 310749 | ENSRNOG00000019671 | ENSRNOT00000026631 | Rsbn1\_predicted |  | 3 |
| 1367749\_at | P51886 | 81682 | ENSRNOG00000004610 | ENSRNOT00000006109 | Lum | Lumican precursor (Keratan sulfate proteoglycan lumican) (KSPG lumican). [Source:UniProtKB/Swiss-Prot;Acc:P51886] | 3 |
| 1379556\_a\_at | Q6F596 | 291211 | ENSRNOG00000016767 | ENSRNOT00000022483 | Ggps1 | Geranylgeranyl pyrophosphate synthetase (GGPP synthetase) (GGPPSase) (Geranylgeranyl diphosphate synthase) [Includes: Dimethylallyltranstransferase (EC 2.5.1.1); Geranyltranstransferase (EC 2.5.1.10); Farnesyltranstransferase (EC 2.5.1.29)]. [Source:UniProtKB/Swiss-Prot;Acc:Q6F596] | 3 |
| 1385765\_at |  | 687456  689523  685380  360888  687459  685393 | ENSRNOG00000023304 | ENSRNOT00000057706 | LOC360888 |  | 3 |
| 1386908\_at | Q9ESH6 | 64045 | ENSRNOG00000012183 | ENSRNOT00000016372 | Glrx1 | Glutaredoxin-1 (Thioltransferase-1) (TTase-1). [Source:UniProtKB/Swiss-Prot;Acc:Q9ESH6] | 3 |
| 1391042\_at |  |  | ENSRNOG00000000075 | ENSRNOT00000000086 | Mtf2 | Mtf2 protein (Fragment). [Source:UniProtKB/TrEMBL;Acc:Q566Q3] | 3 |
| 1393257\_at | Q4QQT3 | 362160 | ENSRNOG00000010379 | ENSRNOT00000014484 | Cugbp1 | CUG-BP- and ETR-3-like factor 1 (CELF-1) (Bruno-like protein 2) (RNA- binding protein BRUNOL-2) (CUG triplet repeat RNA-binding protein 1) (CUG-BP1). [Source:UniProtKB/Swiss-Prot;Acc:Q4QQT3] | 3 |
| 1387926\_at |  | 114100 | ENSRNOG00000008305 | ENSRNOT00000011284 | Sc5d | sterol-C5-desaturase (fungal ERG3, delta-5-desaturase) homolog [Source:RefSeq\_peptide;Acc:NP\_446094] | 3 |
| 1384500\_at |  |  | ENSRNOG00000008560 | ENSRNOT00000011325 | 1700041C02Rik | RIKEN cDNA 1700041C02 gene Gene [Source:MGI Symbol;Acc:MGI:1920582] | 3 |
| 1390977\_at |  | 295344 | ENSRNOG00000014002 | ENSRNOT00000019129  ENSRNOT00000056003  ENSRNOT00000056001 | St7l | suppression of tumorigenicity 7-like [Source:RefSeq\_peptide;Acc:NP\_001007640] | 3 |
| 1393118\_at |  | 294948 | ENSRNOG00000013253 | ENSRNOT00000018548 | NP\_001099895.1 | armadillo repeat containing 1 [Source:RefSeq\_peptide;Acc:NP\_001099895] | 3 |
| 1376260\_at |  | 295428 | ENSRNOG00000015250 | ENSRNOT00000020574 | NP\_001099940.1 |  | 3 |
| 1376749\_at |  | 291015 | ENSRNOG00000029792 | ENSRNOT00000020532 | NP\_001099573.1 | osteoglycin [Source:RefSeq\_peptide;Acc:NP\_001099573] | 3 |
| 1371253\_at | P13803 | 300726 | ENSRNOG00000015233 | ENSRNOT00000020544 | Etfa | Electron transfer flavoprotein subunit alpha, mitochondrial precursor (Alpha-ETF). [Source:UniProtKB/Swiss-Prot;Acc:P13803] | 3 |
| 1393149\_at |  | 393088  393089  393090  116742  393091  393092 | ENSRNOG00000020119 | ENSRNOT00000027393  ENSRNOT00000027383  ENSRNOT00000027377  ENSRNOT00000027372  ENSRNOT00000027349  ENSRNOT00000048626  ENSRNOT00000027343  ENSRNOT00000027340 | Pcdhac2 | protocadherin alpha 13 [Source:RefSeq\_peptide;Acc:NP\_446386] | 3 |
| 1372274\_at |  |  | ENSRNOG00000021614 | ENSRNOT00000014652 | Mll5 | Mll5 protein (Fragment). [Source:UniProtKB/TrEMBL;Acc:Q5BJY5] | 3 |
| 1368467\_at | P33274 | 56266 | ENSRNOG00000004786 | ENSRNOT00000006533 | Cyp4f1 | Cytochrome P450 4F1 (EC 1.14.14.1) (CYPIVF1) (P450-A3). [Source:UniProtKB/Swiss-Prot;Acc:P33274] | 3 |
| 1371988\_at |  | 294410 | ENSRNOG00000000800 | ENSRNOT00000001048  ENSRNOT00000057422 | Man1a\_predicted | mannosidase, alpha, class 1A, member 1 [Source:RefSeq\_peptide;Acc:NP\_001028828] | 3 |
| 1384149\_at |  | 362685 | ENSRNOG00000025317 | ENSRNOT00000034919 | NP\_001102170.1 |  | 3 |
| 1372111\_at | P41350 | 25404 | ENSRNOG00000006694 | ENSRNOT00000009253 | Cav1 | Caveolin-1. [Source:UniProtKB/Swiss-Prot;Acc:P41350] | 3 |
| 1379814\_at |  |  | ENSRNOG00000022637 | ENSRNOT00000032008 | Q7TN00\_RAT | Cardiac titin N2BA isoform (Fragment). [Source:UniProtKB/TrEMBL;Acc:Q7TN00] | 3 |
| 1373837\_at |  | 291906 | ENSRNOG00000011956 | ENSRNOT00000060865 | Fts | AKT interacting protein [Source:RefSeq\_peptide;Acc:NP\_001011926] | 3 |
| 1383705\_at |  |  | ENSRNOG00000031995 | ENSRNOT00000047282 | 2810008M24Rik | RIKEN cDNA 2810008M24 gene Gene [Source:MGI Symbol;Acc:MGI:1922866] | 3 |
| 1372298\_at |  | 298066 | ENSRNOG00000005841 | ENSRNOT00000007711 | Txndc4 | thioredoxin domain containing 4 (endoplasmic reticulum) [Source:RefSeq\_peptide;Acc:NP\_001008318] | 3 |
| 1398710\_at | Q4V8D1 | 310848 | ENSRNOG00000011053 | ENSRNOT00000055570 | Cyp2u1 | Cytochrome P450 2U1 (EC 1.14.14.1). [Source:UniProtKB/Swiss-Prot;Acc:Q4V8D1] | 3 |
| 1393561\_at |  |  | ENSRNOG00000030213 | ENSRNOT00000029132 | Vps13c | vacuolar protein sorting 13C (yeast) Gene [Source:MGI Symbol;Acc:MGI:2444207] | 3 |
| 1371647\_at |  | 309475 | ENSRNOG00000013443 | ENSRNOT00000018043 | RGD1564625\_predicted |  | 3 |
| 1367671\_at | P04961 | 25737 | ENSRNOG00000021264 | ENSRNOT00000028887 | Pcna | Proliferating cell nuclear antigen (PCNA) (Cyclin). [Source:UniProtKB/Swiss-Prot;Acc:P04961] | 3 |
| 1377703\_at | Q6AYT4 | 309420 | ENSRNOG00000015369 | ENSRNOT00000020613 | RGD1310316 | Protein FAM122A. [Source:UniProtKB/Swiss-Prot;Acc:Q6AYT4] | 3 |
| 1379245\_at | Q8K581 | 280671 | ENSRNOG00000018593 | ENSRNOT00000025098 | Txndc9 | Thioredoxin domain-containing protein 9 (ES cell-related protein). [Source:UniProtKB/Swiss-Prot;Acc:Q8K581] | 3 |
| 1390973\_at |  | 295323 | ENSRNOG00000015347 | ENSRNOT00000056150  ENSRNOT00000020615  ENSRNOT00000043146 | NP\_001099923.1 | tripartite motif protein 45 [Source:RefSeq\_peptide;Acc:NP\_001099923] | 3 |
| 1368426\_at | P11466 | 83842 | ENSRNOG00000006779 | ENSRNOT00000010060 | Crot | Peroxisomal carnitine O-octanoyltransferase (EC 2.3.1.137) (COT). [Source:UniProtKB/Swiss-Prot;Acc:P11466] | 3 |
| 1387208\_at | Q99JA8 | 85382 | ENSRNOG00000011719 | ENSRNOT00000015590  ENSRNOT00000016057 | Ngb | Neuroglobin. [Source:UniProtKB/Swiss-Prot;Acc:Q99JA8] | 3 |
| 1387259\_at | Q9Z1Y3 | 83501 | ENSRNOG00000015602 | ENSRNOT00000021170 | Cdh2 | Cadherin-2 precursor (Neural cadherin) (N-cadherin) (CD325 antigen). [Source:UniProtKB/Swiss-Prot;Acc:Q9Z1Y3] | 3 |
| 1374183\_at |  | 299043 | ENSRNOG00000004509 | ENSRNOT00000006030  ENSRNOT00000006101 | NP\_001100199.1 | similar to RIKEN cDNA 1810011O16 (predicted) (RGD1309624\_predicted), mRNA [Source:RefSeq\_dna;Acc:NM\_001106729] | 3 |
| 1374465\_at |  | 299313 | ENSRNOG00000009893 | ENSRNOT00000013476 | Uxt | ubiquitously expressed transcript [Source:RefSeq\_peptide;Acc:NP\_001006983] | 3 |
| 1390118\_at |  | 311166 | ENSRNOG00000007416 | ENSRNOT00000009726 | RGD1307679 | ATP/GTP-binding protein [Source:RefSeq\_peptide;Acc:NP\_001009599] | 3 |
| 1382902\_at |  | 362376 | ENSRNOG00000023969 | ENSRNOT00000031023  ENSRNOT00000058626 | Q5U2P8\_RAT | Herc6 protein (Fragment). [Source:UniProtKB/TrEMBL;Acc:Q5U2P8] | 3 |
| 1395689\_at |  | 310013 | ENSRNOG00000016505 | ENSRNOT00000022120 | LOC310013 |  | 3 |
| 1368393\_at |  |  | ENSRNOG00000024806 | ENSRNOT00000034279 |  |  | 3 |
| 1387266\_at | Q920M9 | 140941 | ENSRNOG00000015143 | ENSRNOT00000020329 | Siah1a | E3 ubiquitin-protein ligase SIAH1 (EC 6.3.2.-) (Seven in absentia homolog 1) (Siah-1) (Siah-1a). [Source:UniProtKB/Swiss-Prot;Acc:Q920M9] | 3 |
| 1374588\_at |  | 362409 | ENSRNOG00000006813 | ENSRNOT00000009008 | NP\_001102109.1 | sulfatase modifying factor 1 [Source:RefSeq\_peptide;Acc:NP\_001102109] | 3 |
| 1371527\_at | P54848 | 25314 | ENSRNOG00000008676 | ENSRNOT00000011580 | Emp1 | Epithelial membrane protein 1 (EMP-1) (Tumor-associated membrane protein). [Source:UniProtKB/Swiss-Prot;Acc:P54848] | 3 |
| 1372266\_at |  | 309812 | ENSRNOG00000000593 | ENSRNOT00000000725 | Rev3l | REV3-like, catalytic subunit of DNA polymerase zeta RAD54 like [Source:RefSeq\_peptide;Acc:NP\_001077435] | 3 |
| 1373905\_at |  |  | ENSRNOG00000011910 | ENSRNOT00000015971 | Hnrpr | heterogeneous nuclear ribonucleoprotein R [Source:RefSeq\_peptide;Acc:NP\_783193] | 3 |
| 1375975\_at |  |  | ENSRNOG00000006717 | ENSRNOT00000056301 | RGD1310736\_predicted |  | 3 |
| 1383250\_at |  |  | ENSRNOG00000005012 | ENSRNOT00000048022  ENSRNOT00000006726 | Q5M811\_RAT | Utp14a protein. [Source:UniProtKB/TrEMBL;Acc:Q5M811] | 3 |
| 1390058\_at | Q498T2 | 361990  500378 | ENSRNOG00000012760 | ENSRNOT00000017561  ENSRNOT00000010455 | LOC500378 | Uncharacterized protein C1orf77 homolog. [Source:UniProtKB/Swiss-Prot;Acc:Q498T2] | 3 |
| 1375870\_a\_at | Q5PQP1 | 362138 | ENSRNOG00000008482 | ENSRNOT00000011682 | Rbms1 | RNA-binding motif, single-stranded-interacting protein 1. [Source:UniProtKB/Swiss-Prot;Acc:Q5PQP1] | 3 |
| 1398431\_at | Q5PPN4 | 297814 | ENSRNOG00000005669 | ENSRNOT00000007802 | Car8 | Carbonic anhydrase-related protein (CARP) (CA-VIII). [Source:UniProtKB/Swiss-Prot;Acc:Q5PPN4] | 3 |
| 1372438\_at |  | 288174 | ENSRNOG00000027797 | ENSRNOT00000029420 | Nit2 | nitrilase family, member 2 [Source:RefSeq\_peptide;Acc:NP\_001029298] | 3 |
| 1372533\_at |  | 297504 | ENSRNOG00000007944 | ENSRNOT00000010810 | RGD1563633\_predicted |  | 3 |
| 1384920\_at |  | 362171 | ENSRNOG00000004666 | ENSRNOT00000006291 | RGD1309969 | similar to RIKEN cDNA 2600010E01 (RGD1309969), mRNA [Source:RefSeq\_dna;Acc:NM\_001080150] | 3 |
| 1382853\_at |  | 683844  316552  683019  689577 | ENSRNOG00000016205 | ENSRNOT00000021681 | Rnf11\_predicted |  | 3 |
| 1368650\_at | O08876 | 81813 | ENSRNOG00000006118 | ENSRNOT00000008350 | Klf10 | Krueppel-like factor 10 (Transforming growth factor-beta-inducible early growth response protein 1) (TGFB-inducible early growth response protein 1) (TIEG-1) (Zinc finger transcription factor homolog CPG20). [Source:UniProtKB/Swiss-Prot;Acc:O08876] | 3 |
| 1388796\_at | Q62931 | 94189 | ENSRNOG00000003971 | ENSRNOT00000005283 | Gosr1 | Golgi SNAP receptor complex member 1 (28 kDa Golgi SNARE protein) (28 kDa cis-Golgi SNARE p28) (GOS-28). [Source:UniProtKB/Swiss-Prot;Acc:Q62931] | 3 |
| 1368642\_at | Q9Z1Y3 | 83501 | ENSRNOG00000015602 | ENSRNOT00000021170 | Cdh2 | Cadherin-2 precursor (Neural cadherin) (N-cadherin) (CD325 antigen). [Source:UniProtKB/Swiss-Prot;Acc:Q9Z1Y3] | 3 |
| 1370376\_a\_at | Q62764 | 83807 | ENSRNOG00000005480 | ENSRNOT00000007427 | Csda | DNA-binding protein A (Cold shock domain-containing protein A) (Muscle Y-box protein YB2) (Y-box-binding protein-A) (RYB-A). [Source:UniProtKB/Swiss-Prot;Acc:Q62764] | 3 |
| 1368525\_at | Q9ET09 | 79032 | ENSRNOG00000017545 | ENSRNOT00000023787 | Mrs2l | Magnesium transporter MRS2L, mitochondrial precursor (MRS2-like protein). [Source:UniProtKB/Swiss-Prot;Acc:Q9ET09] | 3 |
| 1388150\_at | Q80U96 | 85252 | ENSRNOG00000009935 | ENSRNOT00000014062 | Xpo1 | Exportin-1 (Exp1) (Chromosome region maintenance 1 protein homolog). [Source:UniProtKB/Swiss-Prot;Acc:Q80U96] | 3 |
| 1382478\_at |  | 311462 | ENSRNOG00000008088 | ENSRNOT00000010835 | NP\_001101252.1 | BTB (POZ) domain containing 3 [Source:RefSeq\_peptide;Acc:NP\_001101252] | 3 |
| 1385327\_at |  | 307489 | ENSRNOG00000020062 | ENSRNOT00000027172 | Pcdhb13 | Protocadherin-T4 (Fragment). [Source:UniProtKB/TrEMBL;Acc:Q9JIU1] | 3 |
| 1388893\_at | Q6AYF6 | 306253 | ENSRNOG00000018179 | ENSRNOT00000013291  ENSRNOT00000024624 | Glt8d1 | Glycosyltransferase 8 domain-containing protein 1 (EC 2.4.1.-). [Source:UniProtKB/Swiss-Prot;Acc:Q6AYF6] | 3 |
| 1372107\_at | Q9WUH4 | 25177 | ENSRNOG00000000875 | ENSRNOT00000043820  ENSRNOT00000001169 | Fhl1 | Four and a half LIM domains protein 1 (FHL-1). [Source:UniProtKB/Swiss-Prot;Acc:Q9WUH4] | 3 |
| 1382218\_at |  |  | ENSRNOG00000010497 | ENSRNOT00000048511 | RGD1305807 |  | 3 |
| 1388185\_at | P33568 | 24708 | ENSRNOG00000016029 | ENSRNOT00000021752 | Rb1 | Retinoblastoma-associated protein (PP105) (RB) (Fragment). [Source:UniProtKB/Swiss-Prot;Acc:P33568] | 3 |
| 1383629\_a\_at | Q4G055 | 691538  686263 | ENSRNOG00000017310 | ENSRNOT00000023366 | LOC691538 | RNA-binding protein 40 (RNA-binding motif protein 40) (RNA-binding region-containing protein 3). [Source:UniProtKB/Swiss-Prot;Acc:Q4G055] | 3 |
| 1387944\_at | Q9R0A8 | 192228 | ENSRNOG00000017067 | ENSRNOT00000023101 | Ccdc5 | Coiled-coil domain-containing protein 5. [Source:UniProtKB/Swiss-Prot;Acc:Q9R0A8] | 3 |
| 1373430\_at |  |  | ENSRNOG00000025148 | ENSRNOT00000046559 | NP\_001101730.1 | bromodomain adjacent to zinc finger domain, 2B [Source:RefSeq\_peptide;Acc:NP\_001101730] | 3 |
| 1374636\_at |  | 310352 | ENSRNOG00000014066 | ENSRNOT00000018872 | NP\_001101140.1 | PHD finger protein 17 [Source:RefSeq\_peptide;Acc:NP\_001101140] | 3 |
| 1374484\_at | Q5U2V9 | 288092 | ENSRNOG00000003075 | ENSRNOT00000048738  ENSRNOT00000004116 | Tmem39a | Transmembrane protein 39A. [Source:UniProtKB/Swiss-Prot;Acc:Q5U2V9] | 3 |
| 1388318\_at | P16617 | 24644 | ENSRNOG00000002467 | ENSRNOT00000003390 | Pgk1 | Phosphoglycerate kinase 1 (EC 2.7.2.3). [Source:UniProtKB/Swiss-Prot;Acc:P16617] | 3 |
| 1383908\_at |  | 307148 | ENSRNOG00000018520 | ENSRNOT00000025046 | NP\_001100837.1 | NOL1/NOP2/Sun domain family, member 6 [Source:RefSeq\_peptide;Acc:NP\_001100837] | 3 |
| 1390048\_at |  | 302969 | ENSRNOG00000004478 | ENSRNOT00000060907  ENSRNOT00000006370 | Srrm2\_predicted |  | 3 |
| 1368173\_at | Q9QZ86 | 60373 | ENSRNOG00000016486 | ENSRNOT00000022676 | Nol5 | Nucleolar protein 5 (Nucleolar protein NOP5) (Nopp140-associated protein of 65 kDa). [Source:UniProtKB/Swiss-Prot;Acc:Q9QZ86] | 4 |
| 1398523\_a\_at |  | 288264 | ENSRNOG00000028594 | ENSRNOT00000029985 | NP\_001099363.1 | interferon (alpha and beta) receptor 1 [Source:RefSeq\_peptide;Acc:NP\_001099363] | 4 |
| 1382238\_at |  | 361849 | ENSRNOG00000000397 | ENSRNOT00000000447 | NP\_001102005.1 |  | 4 |
| 1379274\_at | Q63085 | 29516 | ENSRNOG00000011417 | ENSRNOT00000015498 | Pde3b | cGMP-inhibited 3',5'-cyclic phosphodiesterase B (EC 3.1.4.17) (Cyclic GMP-inhibited phosphodiesterase B) (CGI-PDE B) (CGIPDE1). [Source:UniProtKB/Swiss-Prot;Acc:Q63085] | 4 |
| 1384147\_at | Q6VV72 | 317163  302697 | ENSRNOG00000031421 | ENSRNOT00000041237 | Eif1a | Eukaryotic translation initiation factor 1A (eIF-1A) (eIF-4C) (Liver regeneration-related protein LRRG048). [Source:UniProtKB/Swiss-Prot;Acc:Q6VV72] | 4 |
| 1379582\_a\_at |  | 114494 | ENSRNOG00000015423 | ENSRNOT00000021156  ENSRNOT00000041849 | Ccna2 | cyclin A2 [Source:RefSeq\_peptide;Acc:NP\_446154] | 4 |
| 1374879\_x\_at |  | 307070 | ENSRNOG00000015888 | ENSRNOT00000058548 | NP\_001100831.1 | La ribonucleoprotein domain family, member 5 [Source:RefSeq\_peptide;Acc:NP\_001100831] | 4 |
| 1376579\_at | Q68FS4 | 289668 | ENSRNOG00000003289 | ENSRNOT00000004770 | Lap3 | Cytosol aminopeptidase (EC 3.4.11.1) (Leucine aminopeptidase) (LAP) (Leucyl aminopeptidase) (Leucine aminopeptidase 3) (Proline aminopeptidase) (EC 3.4.11.5) (Prolyl aminopeptidase). [Source:UniProtKB/Swiss-Prot;Acc:Q68FS4] | 4 |
| 1388110\_at | P62630 | 171361 | ENSRNOG00000027390  ENSRNOG00000015106  ENSRNOG00000004012  ENSRNOG00000009439  ENSRNOG00000012863  ENSRNOG00000029996 | ENSRNOT00000031981  ENSRNOT00000020291  ENSRNOT00000005341  ENSRNOT00000013608  ENSRNOT00000017180  ENSRNOT00000036217 | Eef1a1  LOC364172 | Elongation factor 1-alpha 1 (EF-1-alpha-1) (Elongation factor 1 A-1) (eEF1A-1) (Elongation factor Tu) (EF-Tu). [Source:UniProtKB/Swiss-Prot;Acc:P62630] | 4 |
| 1384456\_at |  | 313427 | ENSRNOG00000005802 | ENSRNOT00000029228  ENSRNOT00000007778 | Usp24\_predicted | Putative uncharacterized protein (Fragment). [Source:UniProtKB/TrEMBL;Acc:Q4G007] | 4 |
| 1382139\_at |  |  | ENSRNOG00000006614 | ENSRNOT00000030629 | RGD1308795\_predicted |  | 4 |
| 1384352\_at |  | 361878 | ENSRNOG00000012099 | ENSRNOT00000016135 | Papd4 | PAP associated domain containing 4 [Source:RefSeq\_peptide;Acc:NP\_001008373] | 4 |
| 1388867\_at |  | 361178 | ENSRNOG00000019222 | ENSRNOT00000026150  ENSRNOT00000026023 | MGC112830 | similar to transcription factor (MGC112830), mRNA [Source:RefSeq\_dna;Acc:NM\_001025718] | 4 |
| 1395098\_at |  |  | ENSRNOG00000002884 | ENSRNOT00000051763 | LOC498266 | similar to Golgin 45 (Basic leucine zipper nuclear factor 1) (LOC498266), mRNA [Source:RefSeq\_dna;Acc:NM\_001017494] | 4 |
| 1382957\_at |  | 360602 | ENSRNOG00000002835 | ENSRNOT00000003914 | NP\_001101761.1 | similar to cisplatin resistance-associated overexpressed protein (predicted) (RGD1307981\_predicted), mRNA [Source:RefSeq\_dna;Acc:NM\_001108291] | 4 |
| 1385269\_s\_at | Q5BJT7 | 304743 | ENSRNOG00000002514 | ENSRNOT00000003435 | LOC304743 | Coiled-coil domain-containing protein 93. [Source:UniProtKB/Swiss-Prot;Acc:Q5BJT7] | 4 |
| 1382007\_at | Q6AYE2 | 292156 | ENSRNOG00000012957 | ENSRNOT00000050684  ENSRNOT00000017770 | Sh3glb1 | Endophilin-B1 (SH3 domain-containing GRB2-like protein B1). [Source:UniProtKB/Swiss-Prot;Acc:Q6AYE2] | 4 |
| 1392223\_at |  | 60443 | ENSRNOG00000002523 | ENSRNOT00000043052 | Epn2 | Epsin-2 (EPS-15-interacting protein 2). [Source:UniProtKB/Swiss-Prot;Acc:Q9Z1Z3] | 4 |
| 1382521\_at |  | 24398 | ENSRNOG00000013595 | ENSRNOT00000032525 | Gls | glutaminase (Gls), transcript variant 2, mRNA [Source:RefSeq\_dna;Acc:NM\_001109968] | 4 |
| 1383965\_at |  | 684506  689116 | ENSRNOG00000001746 | ENSRNOT00000002381 | NP\_001102995.1 | similar to nuclear cap binding protein subunit 2 (LOC689116), mRNA [Source:RefSeq\_dna;Acc:NM\_001109525] | 4 |
| 1383454\_a\_at |  |  | ENSRNOG00000003767 | ENSRNOT00000005032 | Rps6kc1 | ribosomal protein S6 kinase polypeptide 1 Gene [Source:MGI Symbol;Acc:MGI:2443419] | 4 |
| 1378962\_at | Q5XI03 | 287766 | ENSRNOG00000014354 | ENSRNOT00000019380 | RGD1309655 | Coiled-coil domain-containing protein 45. [Source:UniProtKB/Swiss-Prot;Acc:Q5XI03] | 4 |
| 1388355\_at |  | 291295 | ENSRNOG00000018767 | ENSRNOT00000025550 | Rbm17 | RNA binding motif protein 17 [Source:RefSeq\_peptide;Acc:NP\_001013076] | 4 |
| 1377723\_at |  | 303403 | ENSRNOG00000003679 | ENSRNOT00000035280 | NP\_001100505.1 | thyroid hormone receptor associated protein 1 [Source:RefSeq\_peptide;Acc:NP\_001100505] | 4 |
| 1389760\_at | Q5XI70 | 309457 | ENSRNOG00000020250 | ENSRNOT00000027450 | Pcgf6 | Polycomb group RING finger protein 6 (RING finger protein 134). [Source:UniProtKB/Swiss-Prot;Acc:Q5XI70] | 4 |
| 1389130\_at |  | 361944 | ENSRNOG00000010815 | ENSRNOT00000047635 | Elf2 | E74-like factor 2 isoform 1 [Source:RefSeq\_peptide;Acc:NP\_001029081] | 4 |
| 1376704\_a\_at |  | 309259 | ENSRNOG00000016489 | ENSRNOT00000022069 | Ndnl2 | Ndnl2 protein (Fragment). [Source:UniProtKB/TrEMBL;Acc:Q4KM72] | 4 |
| 1378902\_at |  | 304423 | ENSRNOG00000024352 | ENSRNOT00000038092 | NP\_001100607.1 | radical S-adenosyl methionine and flavodoxin domains 1 [Source:RefSeq\_peptide;Acc:NP\_001100607] | 4 |
| 1388313\_at | P62853 | 691710  686507  366605  122799  691532  687806  686426  685085  501042 | ENSRNOG00000031703  ENSRNOG00000027503  ENSRNOG00000038966  ENSRNOG00000032288  ENSRNOG00000029737 | ENSRNOT00000040710  ENSRNOT00000029546  ENSRNOT00000059561  ENSRNOT00000048883  ENSRNOT00000044779 | RGD1563613\_predicted  Rps25    LOC686426  RGD1564597\_predicted | 40S ribosomal protein S25. [Source:UniProtKB/Swiss-Prot;Acc:P62853] | 4 |
| 1378551\_at |  | 316435 | ENSRNOG00000017631 | ENSRNOT00000023796 | Cyp20a1 | cytochrome P450, family 20, subfamily A, polypeptide 1 [Source:RefSeq\_peptide;Acc:NP\_955433] | 4 |
| 1372811\_at |  |  | ENSRNOG00000012255 | ENSRNOT00000016449 | Ktn1 | kinectin 1 Gene [Source:MGI Symbol;Acc:MGI:109153] | 4 |
| 1389253\_at |  | 29142 | ENSRNOG00000016219 | ENSRNOT00000021694 | Vnn1 | vanin 1 [Source:RefSeq\_peptide;Acc:NP\_001020794] | 4 |
| 1380066\_at |  | 288562 | ENSRNOG00000001407 | ENSRNOT00000001905 | Trfr2\_predicted | transferrin receptor 2 [Source:RefSeq\_peptide;Acc:NP\_001099386] | 4 |
| 1389202\_at |  |  | ENSRNOG00000038085 | ENSRNOT00000050635 | MGC124653 | Similar to Ribulose-5-phosphate-3-epimerase. [Source:UniProtKB/TrEMBL;Acc:Q3MIF0] | 4 |
| 1382489\_at |  | 315843 | ENSRNOG00000008652 | ENSRNOT00000011864  ENSRNOT00000036864 | RGD1564964\_predicted |  | 4 |
| 1368057\_at | P16970 | 25270 | ENSRNOG00000011929 | ENSRNOT00000016739 | Abcd3 | ATP-binding cassette sub-family D member 3 (70 kDa peroxisomal membrane protein) (PMP70). [Source:UniProtKB/Swiss-Prot;Acc:P16970] | 4 |
| 1378629\_at |  | 316164 | ENSRNOG00000012942 | ENSRNOT00000017556 | Satb1 | special AT-rich sequence binding protein 1 [Source:RefSeq\_peptide;Acc:NP\_001012129] | 4 |
| 1384262\_at |  | 192280 | ENSRNOG00000011474 | ENSRNOT00000051720 | Ppp1r3b | protein phosphatase 1, regulatory (inhibitor) subunit 3B [Source:RefSeq\_peptide;Acc:NP\_620267] | 4 |
| 1387951\_at |  | 64036 | ENSRNOG00000003927 | ENSRNOT00000005318 | Daf1 | decay accelarating factor 1 [Source:RefSeq\_peptide;Acc:NP\_071605] | 4 |
| 1370355\_at | P07308 | 246074  681458 | ENSRNOG00000013552 | ENSRNOT00000018447 | Scd1 | Acyl-CoA desaturase 1 (EC 1.14.19.1) (Stearoyl-CoA desaturase 1) (Fatty acid desaturase 1) (Delta(9)-desaturase 1). [Source:UniProtKB/Swiss-Prot;Acc:P07308] | 4 |
| 1387101\_at | O35547 | 113976 | ENSRNOG00000019180 | ENSRNOT00000026057 | Acsl4 | Long-chain-fatty-acid--CoA ligase 4 (EC 6.2.1.3) (Long-chain acyl-CoA synthetase 4) (LACS 4). [Source:UniProtKB/Swiss-Prot;Acc:O35547] | 4 |
| 1395399\_at |  | 500555 | ENSRNOG00000012266 | ENSRNOT00000017156 | NP\_001102737.1 | similar to PS1D protein (predicted) (RGD1565267\_predicted), mRNA [Source:RefSeq\_dna;Acc:NM\_001109267] | 4 |
| 1367767\_at | P97519 | 79238 | ENSRNOG00000009422 | ENSRNOT00000012853 | Hmgcl | Hydroxymethylglutaryl-CoA lyase, mitochondrial precursor (EC 4.1.3.4) (HMG-CoA lyase) (HL) (3-hydroxy-3-methylglutarate-CoA lyase). [Source:UniProtKB/Swiss-Prot;Acc:P97519] | 4 |
| 1392523\_at |  | 304092 | ENSRNOG00000002021 | ENSRNOT00000002769 | Son | Son protein (Fragment). [Source:UniProtKB/TrEMBL;Acc:Q6PDU3] | 4 |
| 1385428\_at |  | 290280 | ENSRNOG00000010137 | ENSRNOT00000013901 | NP\_001099512.1 | exportin 4 [Source:RefSeq\_peptide;Acc:NP\_001099512] | 4 |
| 1377670\_at |  |  | ENSRNOG00000009891 | ENSRNOT00000013552 | Pcf11 | cleavage and polyadenylation factor subunit homolog (S. cerevisiae) Gene [Source:MGI (curated);Acc:Pcf11-001] | 4 |
| 1368118\_at | Q9QYN5 | 83477 | ENSRNOG00000014613 | ENSRNOT00000019911 | Bcl10 | B-cell lymphoma/leukemia 10 (B-cell CLL/lymphoma 10) (Bcl-10) (R-RCD1) (RCD). [Source:UniProtKB/Swiss-Prot;Acc:Q9QYN5] | 4 |
| 1387973\_at | P51869 | 286904 | ENSRNOG00000032895 | ENSRNOT00000041034 | Cyp4f4 | Cytochrome P450 4F4 (EC 1.14.14.1) (CYPIVF4). [Source:UniProtKB/Swiss-Prot;Acc:P51869] | 4 |
| 1379550\_a\_at |  | 246770 | ENSRNOG00000001478 | ENSRNOT00000049866  ENSRNOT00000002010  ENSRNOT00000002013  ENSRNOT00000002007 | Gtf2ird1 | general transcription factor II I repeat domain-containing 1 [Source:RefSeq\_peptide;Acc:NP\_001001504] | 4 |
| 1385592\_at |  |  | ENSRNOG00000034240 | ENSRNOT00000041003 | Bcor\_predicted |  | 4 |
| 1373574\_at |  | 363886 | ENSRNOG00000027175 | ENSRNOT00000033288 | NP\_001102322.1 |  | 4 |
| 1377676\_at | Q9EPJ0 | 64709 | ENSRNOG00000004262 | ENSRNOT00000005634 | Nucks | Nuclear ubiquitous casein and cyclin-dependent kinases substrate. [Source:UniProtKB/Swiss-Prot;Acc:Q9EPJ0] | 4 |
| 1388964\_at | Q66HC7 | 361946 | ENSRNOG00000013100 | ENSRNOT00000058026  ENSRNOT00000017923 | RGD1307812 | Protein FAM48A. [Source:UniProtKB/Swiss-Prot;Acc:Q66HC7] | 4 |
| 1370173\_at | P07895 | 24787 | ENSRNOG00000019048 | ENSRNOT00000025794 | Sod2 | Superoxide dismutase [Mn], mitochondrial precursor (EC 1.15.1.1). [Source:UniProtKB/Swiss-Prot;Acc:P07895] | 4 |
| 1367500\_at |  | 679014  680532 | ENSRNOG00000010420 | ENSRNOT00000013841 | LOC680532 |  | 4 |
| 1381881\_at | P60570 | 315435 | ENSRNOG00000019894  ENSRNOG00000010060 | ENSRNOT00000049453  ENSRNOT00000013577  ENSRNOT00000045203 | LOC686478  Panx1 | Pannexin-1. [Source:UniProtKB/Swiss-Prot;Acc:P60570] | 4 |
| 1381575\_at |  |  | ENSRNOG00000006783 | ENSRNOT00000008425 | NP\_001101201.1 | nebulin [Source:RefSeq\_peptide;Acc:NP\_001101201] | 4 |
| 1388076\_at | Q6AXS5 | 246303 | ENSRNOG00000005890 | ENSRNOT00000009503 | Serbp1 | Plasminogen activator inhibitor 1 RNA-binding protein (PAI1 RNA- binding protein 1) (PAI-RBP1) (SERPINE1 mRNA-binding protein 1) (RDA288). [Source:UniProtKB/Swiss-Prot;Acc:Q6AXS5] | 4 |
| 1393248\_at |  | 310512 | ENSRNOG00000009310 | ENSRNOT00000012417 | NP\_001101152.1 | NMD3 homolog [Source:RefSeq\_peptide;Acc:NP\_001101152] | 4 |
| 1372653\_at |  | 300211 | ENSRNOG00000014566 | ENSRNOT00000019527 | Fkbp11 | FK506 binding protein 11 [Source:RefSeq\_peptide;Acc:NP\_001013123] | 4 |
| 1381130\_at |  | 296178 | ENSRNOG00000021272 | ENSRNOT00000028898  ENSRNOT00000028897 | NP\_001099984.1 | minichromosome maintenance deficient 8 [Source:RefSeq\_peptide;Acc:NP\_001099984] | 4 |
| 1385265\_a\_at |  | 686480  690728 | ENSRNOG00000001808 | ENSRNOT00000002473 | LOC686480 | similar to Protein C12orf11 (Sarcoma antigen NY-SAR-95) (LOC690728), mRNA [Source:RefSeq\_dna;Acc:NM\_001109608] | 4 |
| 1394136\_at |  | 307050 | ENSRNOG00000016578 | ENSRNOT00000022462 | NP\_001100829.1 | PAP associated domain containing 1 [Source:RefSeq\_peptide;Acc:NP\_001100829] | 4 |
| 1381878\_at |  | 302935  687555  679694 | ENSRNOG00000003019 | ENSRNOT00000061236  ENSRNOT00000004088 | LOC687555 | ubinuclein 1 [Source:RefSeq\_peptide;Acc:NP\_001100447] | 4 |
| 1389655\_at |  | 287593 | ENSRNOG00000004288 | ENSRNOT00000005661 | RGD1306819 | Bcl-2 inhibitor of transcription [Source:RefSeq\_peptide;Acc:NP\_001013882] | 4 |
| 1392092\_at |  | 317580 | ENSRNOG00000006698 | ENSRNOT00000008868 | NP\_001101727.1 | RAB33A, member of RAS oncogene family [Source:RefSeq\_peptide;Acc:NP\_001101727] | 4 |
| 1388550\_at | Q4KMA2 | 298012 | ENSRNOG00000016137 | ENSRNOT00000021629 | Rad23b | UV excision repair protein RAD23 homolog B. [Source:UniProtKB/Swiss-Prot;Acc:Q4KMA2] | 4 |
| 1370823\_at | Q91XN4 | 83837 | ENSRNOG00000016066 | ENSRNOT00000021801 | Bambi | BMP and activin membrane-bound inhibitor homolog precursor (Kinase- deficient TGFbeta superfamily receptor subunit). [Source:UniProtKB/Swiss-Prot;Acc:Q91XN4] | 4 |
| 1371952\_at |  | 311902 | ENSRNOG00000006763 | ENSRNOT00000009706 | NP\_001101308.1 | RNA binding motif protein 18 [Source:RefSeq\_peptide;Acc:NP\_001101308] | 4 |
| 1391462\_at |  | 361054 | ENSRNOG00000014659 | ENSRNOT00000020036 | Ebpl\_predicted |  | 4 |
| 1385268\_at | Q5BJT7 | 304743 | ENSRNOG00000002514 | ENSRNOT00000003435 | LOC304743 | Coiled-coil domain-containing protein 93. [Source:UniProtKB/Swiss-Prot;Acc:Q5BJT7] | 4 |
| 1368331\_at | Q01460 | 81652 | ENSRNOG00000015573 | ENSRNOT00000020972  ENSRNOT00000020899 | Ctbs | Di-N-acetylchitobiase precursor (EC 3.2.1.-). [Source:UniProtKB/Swiss-Prot;Acc:Q01460] | 4 |
| 1383902\_at |  | 304007 | ENSRNOG00000001609 | ENSRNOT00000002191 | NP\_001100566.1 | leucine-rich repeats and IQ motif containing 2 [Source:RefSeq\_peptide;Acc:NP\_001100566] | 4 |
| 1370948\_a\_at | P30009 | 681252  294446 | ENSRNOG00000000579 | ENSRNOT00000000707 | LOC294446 | Myristoylated alanine-rich C-kinase substrate (MARCKS) (Protein kinase C substrate 80 kDa protein). [Source:UniProtKB/Swiss-Prot;Acc:P30009] | 4 |
| 1381019\_x\_at | Q1AAU6 | 314961 | ENSRNOG00000005739 | ENSRNOT00000045503 | Ddef1\_predicted | 130 kDa phosphatidylinositol 4,5-biphosphate-dependent ARF1 GTPase- activating protein (PIP2-dependent ARF1 GAP) (ADP-ribosylation factor- directed GTPase-activating protein 1) (ARF GTPase-activating protein 1) (Development and differentiation-enhancing f [Source:UniProtKB/Swiss-Prot;Acc:Q1AAU6] | 4 |
| 1371074\_a\_at | Q62724 | 29685 | ENSRNOG00000003703 | ENSRNOT00000004969 | Mcm6 | DNA replication licensing factor MCM6 (Intestinal DNA replication protein) (Fragment). [Source:UniProtKB/Swiss-Prot;Acc:Q62724] | 4 |
| 1393161\_at | Q5BK68 | 362537 | ENSRNOG00000010825 | ENSRNOT00000015254 | Snapc3 | snRNA-activating protein complex subunit 3 (SNAPc subunit 3) (Small nuclear RNA-activating complex polypeptide 3). [Source:UniProtKB/Swiss-Prot;Acc:Q5BK68] | 4 |
| 1373994\_at |  |  | ENSRNOG00000009757 | ENSRNOT00000041234 | RGD1565557\_predicted | RGD1565557\_predicted protein. [Source:UniProtKB/TrEMBL;Acc:Q4KLI5] | 4 |
| 1373552\_at |  | 296865 | ENSRNOG00000021748 | ENSRNOT00000016042 | Las1l\_predicted |  | 4 |
| 1376602\_a\_at |  | 300724 | ENSRNOG00000022702 | ENSRNOT00000020309 | Fbxo22 | F-box only protein 22 [Source:RefSeq\_peptide;Acc:NP\_001032859] | 4 |
| 1377842\_at |  | 362728 | ENSRNOG00000010750 | ENSRNOT00000059991  ENSRNOT00000014373 | NP\_001102177.1 | TWIST neighbor [Source:RefSeq\_peptide;Acc:NP\_001102177] | 4 |
| 1383448\_at |  |  | ENSRNOG00000019478 | ENSRNOT00000050853 | Isgf3g | interferon dependent positive acting transcription factor 3 gamma [Source:RefSeq\_peptide;Acc:NP\_001012041] | 4 |
| 1374425\_at |  |  | ENSRNOG00000005882 | ENSRNOT00000058906 | Tle1\_predicted |  | 4 |
| 1393088\_at | Q5U2R4 | 304012 | ENSRNOG00000039567 | ENSRNOT00000002198 | Rg9mtd1 | RNA (guanine-9-)-methyltransferase domain-containing protein 1, mitochondrial precursor (EC 2.1.1.-). [Source:UniProtKB/Swiss-Prot;Acc:Q5U2R4] | 4 |
| 1377100\_at |  |  | ENSRNOG00000001098 | ENSRNOT00000001464 | Pds5b | PDS5, regulator of cohesion maintenance, homolog B (S. cerevisiae) Gene [Source:MGI (curated);Acc:Pds5b-001] | 4 |
| 1388649\_at |  |  | ENSRNOG00000023210 | ENSRNOT00000032410 | Tmem1\_predicted |  | 4 |
| 1385035\_at |  | 360763  690287  684318  501659 | ENSRNOG00000033411  ENSRNOG00000029046  ENSRNOG00000012086 | ENSRNOT00000044038  ENSRNOT00000061131  ENSRNOT00000042095  ENSRNOT00000016098  ENSRNOT00000056504 | Usp12\_predicted |  | 4 |
| 1367588\_a\_at | P35427 | 317646 | ENSRNOG00000025179  ENSRNOG00000020618 | ENSRNOT00000038531  ENSRNOT00000056277  ENSRNOT00000027976 | Rpl13a | 60S ribosomal protein L13a. [Source:UniProtKB/Swiss-Prot;Acc:P35427] | 4 |
| 1368540\_at | Q5PQV5 | 83684 | ENSRNOG00000010694 | ENSRNOT00000014326  ENSRNOT00000056928 | Tpbg | Trophoblast glycoprotein precursor (5T4 oncofetal trophoblast glycoprotein) (5T4 oncotrophoblast glycoprotein). [Source:UniProtKB/Swiss-Prot;Acc:Q5PQV5] | 4 |
| 1381018\_a\_at | Q1AAU6 | 314961 | ENSRNOG00000005739 | ENSRNOT00000045503 | Ddef1\_predicted | 130 kDa phosphatidylinositol 4,5-biphosphate-dependent ARF1 GTPase- activating protein (PIP2-dependent ARF1 GAP) (ADP-ribosylation factor- directed GTPase-activating protein 1) (ARF GTPase-activating protein 1) (Development and differentiation-enhancing f [Source:UniProtKB/Swiss-Prot;Acc:Q1AAU6] | 4 |
| 1393643\_at |  | 362182 | ENSRNOG00000013452 | ENSRNOT00000018074 | NP\_001102056.1 | reticulocalbin 1 [Source:RefSeq\_peptide;Acc:NP\_001102056] | 4 |
| 1376384\_at |  | 311151 | ENSRNOG00000008868 | ENSRNOT00000057826  ENSRNOT00000012290 | NM\_001107739.1 | dual specificity phosphatase 19 (predicted) (Dusp19\_predicted), mRNA [Source:RefSeq\_dna;Acc:NM\_001107739] | 4 |
| 1372697\_at |  | 298517 | ENSRNOG00000008279 | ENSRNOT00000011565 | Mrps15 | mitochondrial ribosomal protein S15 [Source:RefSeq\_peptide;Acc:NP\_001007654] | 4 |
| 1385673\_at | Q4G2Y1 | 287463 | ENSRNOG00000006913 | ENSRNOT00000009082  ENSRNOT00000044363 | RGD1308492 | RPA-interacting protein. [Source:UniProtKB/Swiss-Prot;Acc:Q4G2Y1] | 4 |
| 1374547\_at |  | 502663 | ENSRNOG00000015277 | ENSRNOT00000020756 | LOC502663 | small EDRK-rich factor 2 [Source:RefSeq\_peptide;Acc:NP\_001092252] | 4 |
| 1393581\_at |  | 289054 | ENSRNOG00000012318 | ENSRNOT00000016797  ENSRNOT00000060206 | NP\_001099425.1 | asp (abnormal spindle)-like, microcephaly associated [Source:RefSeq\_peptide;Acc:NP\_001099425] | 4 |
| 1389857\_at |  | 680354 | ENSRNOG00000034198 | ENSRNOT00000050302 | LOC680354 |  | 4 |
| 1376094\_at |  | 246769 | ENSRNOG00000014190 | ENSRNOT00000019047 | Hint3 | Histidine triad protein 4 (Fragment). [Source:UniProtKB/TrEMBL;Acc:Q8K3P7] | 4 |
| 1384381\_at |  | 313210 | ENSRNOG00000018126 | ENSRNOT00000024564 | Abca1 | ATP-binding cassette, sub-family A (ABC1), member 1 [Source:RefSeq\_peptide;Acc:NP\_835196] | 4 |
| 1398588\_at | Q80ZG5 | 303057  317672 | ENSRNOG00000003822 | ENSRNOT00000059597  ENSRNOT00000005132  ENSRNOT00000059596 | Slu7 | Pre-mRNA-splicing factor SLU7. [Source:UniProtKB/Swiss-Prot;Acc:Q80ZG5] | 4 |
| 1384154\_at | Q5HZF2 | 114765 | ENSRNOG00000011678 | ENSRNOT00000015605 | Wbp4 | WW domain-binding protein 4 (WBP-4) (WW domain-containing-binding protein 4) (Formin-binding protein 21). [Source:UniProtKB/Swiss-Prot;Acc:Q5HZF2] | 4 |
| 1379044\_at |  | 310348 | ENSRNOG00000038366 | ENSRNOT00000044804 | RGD1307509 | similar to RIKEN cDNA 1700108L22 (RGD1307509), mRNA [Source:RefSeq\_dna;Acc:NM\_001014036] | 4 |
| 1374906\_at |  | 313450 | ENSRNOG00000034040 | ENSRNOT00000051474 | LOC313450 | similar to RIKEN cDNA 2810428C21 (LOC313450), mRNA [Source:RefSeq\_dna;Acc:NM\_001014791] | 4 |
| 1375595\_at |  | 300756 | ENSRNOG00000009887 | ENSRNOT00000013581  ENSRNOT00000046564 | Arih1 | ariadne ubiquitin-conjugating enzyme E2 binding protein homolog 1 [Source:RefSeq\_peptide;Acc:NP\_001013126] | 4 |
| 1370908\_at |  | 500195  84577 | ENSRNOG00000000604 | ENSRNOT00000000742 | Hdac2 | Histone deacetylase 2 (Fragment). [Source:UniProtKB/TrEMBL;Acc:Q99PA1] | 4 |
| 1377730\_at |  | 299207 | ENSRNOG00000008781 | ENSRNOT00000012327 | NP\_001100219.1 | similar to HSPC288 (predicted) (RGD1310769\_predicted), mRNA [Source:RefSeq\_dna;Acc:NM\_001106749] | 4 |
| 1385194\_at |  | 312927 | ENSRNOG00000006833 | ENSRNOT00000010014 | NP\_001101371.1 | RB1-inducible coiled-coil 1 [Source:RefSeq\_peptide;Acc:NP\_001101371] | 4 |
| 1385154\_at |  | 314405 | ENSRNOG00000009117 | ENSRNOT00000012124 | NP\_001101523.1 | OTU domain, ubiquitin aldehyde binding 2 [Source:RefSeq\_peptide;Acc:NP\_001101523] | 4 |
| 1372072\_at | Q6AYR6 | 361351 | ENSRNOG00000018006 | ENSRNOT00000051857  ENSRNOT00000024204 | RGD1308579 | Immediate early response 3-interacting protein 1. [Source:UniProtKB/Swiss-Prot;Acc:P85007] | 4 |
| 1389496\_at |  | 361458 | ENSRNOG00000013202 | ENSRNOT00000017617 | Akap7 | A-kinase anchoring protein 18 ,isoform delta [Source:RefSeq\_peptide;Acc:NP\_001001801] | 4 |
| 1375108\_at | P05506  P05507 | 26199  26200 | ENSRNOG00000033615  ENSRNOG00000031053 | ENSRNOT00000041241  ENSRNOT00000044582 | NU3M\_RAT  NU4LM\_RAT | NADH-ubiquinone oxidoreductase chain 3 (EC 1.6.5.3) (NADH dehydrogenase subunit 3). [Source:UniProtKB/Swiss-Prot;Acc:P05506]  NADH-ubiquinone oxidoreductase chain 4L (EC 1.6.5.3) (NADH dehydrogenase subunit 4L). [Source:UniProtKB/Swiss-Prot;Acc:P05507] | 4 |
| 1390146\_at |  | 360916 | ENSRNOG00000002258 | ENSRNOT00000003076 | NP\_001101824.1 | similar to RIKEN cDNA 2610318G18 (predicted) (RGD1306105\_predicted), mRNA [Source:RefSeq\_dna;Acc:NM\_001108354] | 4 |
| 1367732\_at | O35353  P54311 | 24400  294962 | ENSRNOG00000016638 | ENSRNOT00000041789  ENSRNOT00000022649 | Gnb1 | Guanine nucleotide-binding protein G(I)/G(S)/G(T) subunit beta-1 (Transducin beta chain 1). [Source:UniProtKB/Swiss-Prot;Acc:P54311] | 4 |
| 1382721\_at | Q6GVH5 | 360584 | ENSRNOG00000027860 | ENSRNOT00000029883  ENSRNOT00000042506 | Zfp403 | Gametogenetin-binding protein 2 (Protein ZNF403). [Source:UniProtKB/Swiss-Prot;Acc:Q6GVH5] | 4 |
| 1382219\_at |  | 310348 | ENSRNOG00000038366 | ENSRNOT00000044804 | RGD1307509 | similar to RIKEN cDNA 1700108L22 (RGD1307509), mRNA [Source:RefSeq\_dna;Acc:NM\_001014036] | 4 |
| 1376941\_at |  | 360796 | ENSRNOG00000001483 | ENSRNOT00000002032 | NP\_001101802.1 | Williams-Beuren syndrome chromosome region 16 homolog [Source:RefSeq\_peptide;Acc:NP\_001101802] | 4 |
| 1385519\_at |  | 362489 | ENSRNOG00000005673 | ENSRNOT00000007970 | NP\_001102127.1 | acute myelogenous leukemia 1 translocation 1 protein [Source:RefSeq\_peptide;Acc:NP\_001102127] | 4 |
| 1373386\_at | P21994 | 394266 | ENSRNOG00000008855 | ENSRNOT00000011711 | Gjb2 | Gap junction beta-2 protein (Connexin-26) (Cx26). [Source:UniProtKB/Swiss-Prot;Acc:P21994] | 4 |
| 1385856\_at |  | 291061 | ENSRNOG00000014049 | ENSRNOT00000018936 | Riok1 | Riok1 protein (Fragment). [Source:UniProtKB/TrEMBL;Acc:Q6AY66] | 4 |
| 1372451\_at | Q5XIE9 | 316209  501098 | ENSRNOG00000013852 | ENSRNOT00000018523 | Trfp | Mediator of RNA polymerase II transcription subunit 20 (Mediator complex subunit 20) (TRF-proximal protein homolog). [Source:UniProtKB/Swiss-Prot;Acc:Q5XIE9] | 4 |
| 1382143\_at |  |  | ENSRNOG00000011203 | ENSRNOT00000057523 | NP\_001100757.1 | FERM, RhoGEF (Arhgef) and pleckstrin domain protein 1 (chondrocyte-derived) [Source:RefSeq\_peptide;Acc:NP\_001100757] | 4 |
| 1397447\_at |  | 500108 | ENSRNOG00000006703 | ENSRNOT00000008743 | NP\_001102700.1 | similar to zinc finger protein 398 [Source:RefSeq\_peptide;Acc:NP\_001102700] | 4 |
| 1373290\_at |  | 312299 | ENSRNOG00000006048 | ENSRNOT00000008149  ENSRNOT00000045557 | LOC312299 |  | 4 |
| 1388188\_at | Q63688 | 25429 | ENSRNOG00000009730 | ENSRNOT00000013116 | Cyp7b1 | Cytochrome P450 7B1 (EC 1.14.13.100) (25-hydroxycholesterol 7-alpha- hydroxylase) (Oxysterol 7-alpha-hydroxylase) (HCT-1) (Fragment). [Source:UniProtKB/Swiss-Prot;Acc:Q63688] | 4 |
| 1383508\_at |  |  | ENSRNOG00000011006 | ENSRNOT00000014765 | Stoml3 | stomatin (Epb7.2)-like 3 Gene [Source:MGI (curated);Acc:Stoml3-001] | 4 |
| 1383245\_at |  | 362740 | ENSRNOG00000008610 | ENSRNOT00000011419 | NP\_001102182.1 | MAP3K12 binding inhibitory protein 1 [Source:RefSeq\_peptide;Acc:NP\_001102182] | 4 |
| 1370109\_s\_at | P62630 | 171361 | ENSRNOG00000015106  ENSRNOG00000009439  ENSRNOG00000012863  ENSRNOG00000029996 | ENSRNOT00000020291  ENSRNOT00000013608  ENSRNOT00000017180  ENSRNOT00000036217 | Eef1a1  LOC364172 | Elongation factor 1-alpha 1 (EF-1-alpha-1) (Elongation factor 1 A-1) (eEF1A-1) (Elongation factor Tu) (EF-Tu). [Source:UniProtKB/Swiss-Prot;Acc:P62630] | 4 |
| 1371691\_at |  | 297073 | ENSRNOG00000024705 | ENSRNOT00000033918 | Rarres2 | retinoic acid receptor responder (tazarotene induced) 2 [Source:RefSeq\_peptide;Acc:NP\_001013445] | 4 |
| 1371011\_at |  | 266778  295090  295091 | ENSRNOG00000010268  ENSRNOG00000028443  ENSRNOG00000028345 | ENSRNOT00000013777  ENSRNOT00000057659  ENSRNOT00000057658  ENSRNOT00000057657  ENSRNOT00000057656  ENSRNOT00000057638  ENSRNOT00000057637  ENSRNOT00000057636  ENSRNOT00000031015 | Casrl1  RGD1306812\_predicted  LOC295091 | calcium-sensing receptor like 1 [Source:RefSeq\_peptide;Acc:NP\_001092968]  Putative pheromone receptor V2R2c (Fragment). [Source:UniProtKB/TrEMBL;Acc:Q99PC0]  putative pheromone receptor V2R2B (LOC295091), mRNA [Source:RefSeq\_dna;Acc:NM\_001099502] | 4 |
| 1384886\_at |  | 500057 | ENSRNOG00000007662 | ENSRNOT00000010223 | NP\_001102695.1 |  | 4 |
| 1384407\_at |  | 287063 | ENSRNOG00000003794 | ENSRNOT00000005109  ENSRNOT00000041699 | RGD1311451\_predicted |  | 4 |
| 1373273\_at |  | 298374 | ENSRNOG00000009451 | ENSRNOT00000012635 | NP\_001100142.1 | PRP38 pre-mRNA processing factor 38 (yeast) domain containing A [Source:RefSeq\_peptide;Acc:NP\_001100142] | 4 |
| 1374770\_at |  | 84431 | ENSRNOG00000010034 | ENSRNOT00000013463 | Asah1 | N-acylsphingosine amidohydrolase 1 [Source:RefSeq\_peptide;Acc:NP\_445859] | 4 |
| 1367857\_at | Q920R3 | 84575 | ENSRNOG00000020480 | ENSRNOT00000027834 | Fads1 | Fatty acid desaturase 1 (EC 1.14.19.-) (Delta(5) fatty acid desaturase) (Delta(5) desaturase) (D5D). [Source:UniProtKB/Swiss-Prot;Acc:Q920R3] | 4 |
| 1374567\_at |  |  | ENSRNOG00000004106 | ENSRNOT00000005541  ENSRNOT00000058440 | Q5U3X2\_RAT | Psrc2 protein (Fragment). [Source:UniProtKB/TrEMBL;Acc:Q5U3X2] | 4 |
| 1389191\_at | P0C0A2 | 290851 | ENSRNOG00000012654 | ENSRNOT00000016995 | VPS36\_RAT | Vacuolar protein-sorting-associated protein 36 (ELL-associated protein of 45 kDa). [Source:UniProtKB/Swiss-Prot;Acc:P0C0A2] | 4 |
| 1383962\_at | P59692 | 362791 | ENSRNOG00000028640 | ENSRNOT00000031788  ENSRNOT00000051681 | SIVA\_RAT | Apoptosis regulatory protein Siva (CD27-binding protein) (CD27BP). [Source:UniProtKB/Swiss-Prot;Acc:P59692] | 4 |
| 1372462\_at | Q5XI22 | 308100  364149 | ENSRNOG00000019189 | ENSRNOT00000026003 | Acat2 | Acetyl-CoA acetyltransferase, cytosolic (EC 2.3.1.9) (Cytosolic acetoacetyl-CoA thiolase). [Source:UniProtKB/Swiss-Prot;Acc:Q5XI22] | 4 |
| 1378875\_a\_at | Q6MG12 | 361789  294231 | ENSRNOG00000000809  ENSRNOG00000000812 | ENSRNOT00000001065  ENSRNOT00000001067  ENSRNOT00000001077 | RGD1303066  RGD1302996 | similar to RIKEN cDNA 2610110G12 (RGD1303066), mRNA [Source:RefSeq\_dna;Acc:NM\_212498]  Uncharacterized protein C6orf136 homolog. [Source:UniProtKB/Swiss-Prot;Acc:Q6MG12] | 4 |
| 1389314\_at |  | 289323 | ENSRNOG00000003629 | ENSRNOT00000004927 | NP\_001099450.1 | nuclear VCP-like [Source:RefSeq\_peptide;Acc:NP\_001099450] | 4 |
| 1395108\_at |  | 171494 | ENSRNOG00000015390 | ENSRNOT00000051859 | Ap1g1 | Gamma-adaptin (Fragment). [Source:UniProtKB/TrEMBL;Acc:Q8R525] | 4 |
| 1371875\_at | Q4FZV0 | 310864 | ENSRNOG00000013476 | ENSRNOT00000018202 | Manba | Beta-mannosidase precursor (EC 3.2.1.25) (Lysosomal beta A mannosidase) (Mannanase) (Mannase). [Source:UniProtKB/Swiss-Prot;Acc:Q4FZV0] | 4 |
| 1382466\_at |  | 291060 | ENSRNOG00000013756 | ENSRNOT00000018508 | NP\_001099577.1 | similar to RIKEN cDNA 6530403A03 (RGD1309020), mRNA [Source:RefSeq\_dna;Acc:NM\_001106107] | 4 |
| 1388480\_at |  | 288707 | ENSRNOG00000001192 | ENSRNOT00000001581 | Gltp\_predicted |  | 4 |
| 1392902\_at | Q76MT4 | 366203 | ENSRNOG00000004777 | ENSRNOT00000034487  ENSRNOT00000006333 | RGD1306067 | ESF1 homolog (ABT1-associated protein). [Source:UniProtKB/Swiss-Prot;Acc:Q76MT4] | 4 |
| 1370712\_at | Q5J3K5 | 286957 | ENSRNOG00000032036 | ENSRNOT00000052101 | Vnr2 | Vomeronasal type-1 receptor A15 (Vomeronasal receptor 2) (Pheromone receptor VN2). [Source:UniProtKB/Swiss-Prot;Acc:Q5J3K5] | 4 |
| 1367800\_at | P19637 | 25692 | ENSRNOG00000019018 | ENSRNOT00000025763 | Plat | Tissue-type plasminogen activator precursor (EC 3.4.21.68) (tPA) (t- PA) (t-plasminogen activator) [Contains: Tissue-type plasminogen activator chain A; Tissue-type plasminogen activator chain B]. [Source:UniProtKB/Swiss-Prot;Acc:P19637] | 4 |
| 1386967\_at | Q9JJL4 | 85428 | ENSRNOG00000015415 | ENSRNOT00000020822 | Rhoq | Rho-related GTP-binding protein RhoQ precursor (Ras-related GTP- binding protein TC10). [Source:UniProtKB/Swiss-Prot;Acc:Q9JJL4] | 4 |
| 1393990\_at |  | 305687 | ENSRNOG00000014237 | ENSRNOT00000019644 | NP\_001100720.1 | zinc finger protein 503 [Source:RefSeq\_peptide;Acc:NP\_001100720] | 4 |
| 1389077\_at |  | 362460 | ENSRNOG00000012695 | ENSRNOT00000016937 | Golt1b | golgi transport 1 homolog B (S. cerevisiae) Gene [Source:MGI (curated);Acc:Golt1b-001] | 4 |
| 1390415\_at | Q5XHZ9 | 292206 | ENSRNOG00000015810 | ENSRNOT00000060956 | Trip13 | Thyroid receptor-interacting protein 13 (TRIP-13) (Thyroid hormone receptor interactor 13). [Source:UniProtKB/Swiss-Prot;Acc:Q5XHZ9] | 4 |
| 1396521\_at |  | 291699 | ENSRNOG00000020468 | ENSRNOT00000027755 | NP\_001099629.1 | StAR-related lipid transfer (START) domain containing 4 [Source:RefSeq\_peptide;Acc:NP\_001099629] | 4 |
| 1371039\_at |  | 58942 | ENSRNOG00000007666 | ENSRNOT00000010160 | Cacnb4 | calcium channel, voltage-dependent, beta 4 subunit [Source:RefSeq\_peptide;Acc:NP\_001099203] | 4 |
| 1370928\_at | P0C0T0 | 65161 | ENSRNOG00000002520 | ENSRNOT00000003412 | Litaf | Lipopolysaccharide-induced tumor necrosis factor-alpha factor homolog (LPS-induced TNF-alpha factor homolog) (Estrogen-enhanced transcript protein 1) (Eet-1). [Source:UniProtKB/Swiss-Prot;Acc:P0C0T0] | 4 |
| 1383005\_at | Q91ZQ0 | 192129 | ENSRNOG00000003967 | ENSRNOT00000005658 | Tmem49 | Transmembrane protein 49 (Vacuole membrane protein 1). [Source:UniProtKB/Swiss-Prot;Acc:Q91ZQ0] | 4 |
| 1373418\_at |  | 289352 | ENSRNOG00000002393 | ENSRNOT00000038264  ENSRNOT00000003252 | Eprs | glutamyl-prolyl-tRNA synthetase [Source:RefSeq\_peptide;Acc:NP\_001019409] | 4 |
| 1370062\_at | Q8VH49 | 140937 | ENSRNOG00000019428 | ENSRNOT00000026328 | Higd1a | HIG1 domain family member 1A (Hypoxia-inducible gene 1 protein). [Source:UniProtKB/Swiss-Prot;Acc:Q8VH49] | 4 |
| 1370024\_at | P55051 | 80841 | ENSRNOG00000000814 | ENSRNOT00000001079 | Fabp7 | Fatty acid-binding protein, brain (B-FABP) (Brain lipid-binding protein) (BLBP). [Source:UniProtKB/Swiss-Prot;Acc:P55051] | 4 |
| 1381814\_at |  |  | ENSRNOG00000006733 | ENSRNOT00000031674 | RGD1566016\_predicted |  | 4 |
| 1367582\_at | P25886 | 364828  500019  364985  500645  687044  687209  692035  306926  690662  687178  367874  686728  315051  684399  690690  685714  686807  683273  681881  29283  688309  691477  679255  367175 | ENSRNOG00000031963  ENSRNOG00000028402  ENSRNOG00000030439  ENSRNOG00000033695  ENSRNOG00000025794  ENSRNOG00000028320  ENSRNOG00000029594  ENSRNOG00000025630  ENSRNOG00000007024  ENSRNOG00000025853  ENSRNOG00000038719  ENSRNOG00000033224  ENSRNOG00000027347  ENSRNOG00000021762  ENSRNOG00000029856  ENSRNOG00000037401  ENSRNOG00000011138  ENSRNOG00000032822  ENSRNOG00000037172 | ENSRNOT00000040921  ENSRNOT00000040047  ENSRNOT00000036458  ENSRNOT00000049689  ENSRNOT00000050331  ENSRNOT00000033201  ENSRNOT00000034681  ENSRNOT00000047001  ENSRNOT00000032995  ENSRNOT00000009205  ENSRNOT00000039250  ENSRNOT00000048860  ENSRNOT00000049230  ENSRNOT00000000433  ENSRNOT00000037416  ENSRNOT00000057412  ENSRNOT00000049555  ENSRNOT00000014849  ENSRNOT00000056379  ENSRNOT00000056378  ENSRNOT00000050117 | RGD1559877\_predicted  RGD1562425\_predicted  RGD1566073\_predicted  RGD1563300\_predicted  LOC306476  RGD1566409\_predicted  LOC690662  RGD1564473\_predicted  RGD1563579\_predicted  Rpl29  RGD1564138\_predicted | 60S ribosomal protein L29 (P23). [Source:UniProtKB/Swiss-Prot;Acc:P25886] | 4 |
| 1376450\_at |  | 299841 | ENSRNOG00000004593  ENSRNOG00000004421 | ENSRNOT00000058102  ENSRNOT00000006051 | Tmem5 | transmembrane protein 5 [Source:RefSeq\_peptide;Acc:NP\_001019930] | 4 |
| 1374227\_at |  | 298782 | ENSRNOG00000027191 | ENSRNOT00000006505 | Birc6\_predicted |  | 4 |
| 1376636\_at | P80204 | 29591 | ENSRNOG00000007036 | ENSRNOT00000009452 | Tgfbr1 | TGF-beta receptor type-1 precursor (EC 2.7.11.30) (TGF-beta receptor type I) (TGFR-1) (TGF-beta type I receptor) (Transforming growth factor-beta receptor type I) (TbetaR-I) (Serine/threonine-protein kinase receptor R4) (SKR4). [Source:UniProtKB/Swiss-Prot;Acc:P80204] | 4 |
| 1369157\_at | Q63085 | 29516 | ENSRNOG00000011417 | ENSRNOT00000015498 | Pde3b | cGMP-inhibited 3',5'-cyclic phosphodiesterase B (EC 3.1.4.17) (Cyclic GMP-inhibited phosphodiesterase B) (CGI-PDE B) (CGIPDE1). [Source:UniProtKB/Swiss-Prot;Acc:Q63085] | 4 |
| 1371913\_at |  |  | ENSRNOG00000012216 | ENSRNOT00000016390 | Tgfbi | transforming growth factor, beta induced Gene [Source:MGI Symbol;Acc:MGI:99959] | 4 |
| 1393983\_at |  |  | ENSRNOG00000007088 | ENSRNOT00000009645 | NP\_001101572.1 | exportin, tRNA (nuclear export receptor for tRNAs) [Source:RefSeq\_peptide;Acc:NP\_001101572] | 4 |
| 1384729\_at |  | 304799 | ENSRNOG00000028493  ENSRNOG00000037277 | ENSRNOT00000037039  ENSRNOT00000056360 | NP\_001100645.1 | protein phosphatase 1, regulatory (inhibitor) subunit 15b [Source:RefSeq\_peptide;Acc:NP\_001100645] | 4 |
| 1370821\_at |  | 690050 | ENSRNOG00000016468 | ENSRNOT00000022085 | Tpmt | thiopurine methyltransferase [Source:RefSeq\_peptide;Acc:NP\_001072999] | 4 |
| 1370000\_at | Q9JI85 | 59295 | ENSRNOG00000020456 | ENSRNOT00000027752 | Nucb2 | Nucleobindin-2 precursor (DNA-binding protein NEFA). [Source:UniProtKB/Swiss-Prot;Acc:Q9JI85] | 4 |
| 1383954\_at |  |  | ENSRNOG00000016813 | ENSRNOT00000041366 | Tia1 | cytotoxic granule-associated RNA binding protein 1 [Source:RefSeq\_peptide;Acc:NP\_001012096] | 4 |
| 1378243\_at | Q5HZE5 | 360985 | ENSRNOG00000009220 | ENSRNOT00000012224 | Tmem17 | Transmembrane protein 17. [Source:UniProtKB/Swiss-Prot;Acc:Q5HZE5] | 4 |
| 1374113\_at |  | 314126 | ENSRNOG00000006828 | ENSRNOT00000009133 | Baz1a\_predicted |  | 4 |
| 1381355\_at | Q499Q5 | 287612 | ENSRNOG00000008635 | ENSRNOT00000011500 | Mks1 | Meckel syndrome type 1 protein homolog. [Source:UniProtKB/Swiss-Prot;Acc:Q499Q5] | 4 |
| 1391612\_at | Q7TNI4 | 444986 | ENSRNOG00000012259 | ENSRNOT00000016354 | Il22ra2 | Interleukin-22 receptor alpha-2 chain precursor (IL-22R-alpha-2) (Interleukin-22-binding protein) (IL22BP) (Cytokine receptor family type 2, soluble 1) (CRF2-S1). [Source:UniProtKB/Swiss-Prot;Acc:Q7TNI4] | 4 |
| 1376307\_a\_at | Q8CFD1 | 266631 | ENSRNOG00000010595 | ENSRNOT00000042968 | Drb1 | RNA-binding protein 45 (RNA-binding motif protein 45) (Developmentally-regulated RNA-binding protein 1). [Source:UniProtKB/Swiss-Prot;Acc:Q8CFD1] | 4 |
| 1375969\_at | Q6P752 | 304881 | ENSRNOG00000024849 | ENSRNOT00000050734 | Tor1aip2 | Torsin-1A-interacting protein 2. [Source:UniProtKB/Swiss-Prot;Acc:Q6P752] | 4 |
| 1372522\_at | Q5BJT0 | 290912 | ENSRNOG00000024142 | ENSRNOT00000046755 | RGD1310061 | Arginine and glutamate-rich protein 1. [Source:UniProtKB/Swiss-Prot;Acc:Q5BJT0] | 4 |
| 1379959\_at | Q6P6S5 | 288233 | ENSRNOG00000001629 | ENSRNOT00000002222 | Wrb | Tryptophan-rich protein. [Source:UniProtKB/Swiss-Prot;Acc:Q6P6S5] | 4 |
| 1381925\_x\_at |  | 282546  497729 | ENSRNOG00000017030 | ENSRNOT00000022939 | Arid1b | transcription factor 1 [Source:RefSeq\_peptide;Acc:NP\_750843] | 4 |
| 1386296\_at |  | 303135 | ENSRNOG00000007462 | ENSRNOT00000009841 | NP\_001100472.1 | septin 8 [Source:RefSeq\_peptide;Acc:NP\_001100472] | 4 |
| 1392936\_at |  | 366693  497865 | ENSRNOG00000002871 | ENSRNOT00000003832 | NP\_001102454.1 | RNA binding motif protein 25 [Source:RefSeq\_peptide;Acc:NP\_001102454] | 4 |
| 1378305\_at |  | 295061 | ENSRNOG00000015812 | ENSRNOT00000021535 | NP\_001099904.1 | transmembrane 4 superfamily member 1 [Source:RefSeq\_peptide;Acc:NP\_001099904] | 4 |
| 1378753\_at | Q6P6T5 | 83497 | ENSRNOG00000018297 | ENSRNOT00000024674 | Ocln | Occludin. [Source:UniProtKB/Swiss-Prot;Acc:Q6P6T5] | 4 |
| 1394953\_at |  | 296072 | ENSRNOG00000005209 | ENSRNOT00000007028 | Spred1 | sprouty protein with EVH-1 domain 1, related sequence [Source:RefSeq\_peptide;Acc:NP\_001040554] | 4 |
| 1395986\_at | Q9WVC1 | 360272 | ENSRNOG00000003840 | ENSRNOT00000005477 | Slit2 | Slit homolog 2 protein precursor (Slit-2) (Fragment). [Source:UniProtKB/Swiss-Prot;Acc:Q9WVC1] | 4 |
| 1377810\_at |  | 304887 | ENSRNOG00000004736 | ENSRNOT00000006511 | NP\_001094150.1 | Ral GEF with PH domain and SH3 binding motif 2 [Source:RefSeq\_peptide;Acc:NP\_001094150] | 4 |
| 1390401\_at |  | 85262 | ENSRNOG00000010592 | ENSRNOT00000052068 | Slc25a27 | solute carrier family 25, member 27 [Source:RefSeq\_peptide;Acc:NP\_445952] | 4 |
| 1371354\_at |  | 290561 | ENSRNOG00000018943 | ENSRNOT00000025606 | Tnnc1 | troponin C, cardiac/slow skeletal [Source:RefSeq\_peptide;Acc:NP\_001029277] | 4 |
| 1388924\_at | Q6TMA8 | 362850 | ENSRNOG00000007545 | ENSRNOT00000010031 | Angptl4 | Angiopoietin-related protein 4 precursor (Angiopoietin-like 4) (Hepatic fibrinogen/angiopoietin-related protein) (HFARP). [Source:UniProtKB/Swiss-Prot;Acc:Q6TMA8] | 4 |
| 1379525\_at | Q5U2V5 | 366196 | ENSRNOG00000021273 | ENSRNOT00000028900 | RGD1311037 | Cardiolipin synthetase (EC 2.7.8.-) (Cardiolipin synthase) (CLS). [Source:UniProtKB/Swiss-Prot;Acc:Q5U2V5] | 4 |
| 1388635\_at |  | 311405  498067 | ENSRNOG00000022727 | ENSRNOT00000031577 | RGD1309744\_predicted | Putative uncharacterized protein RGD1309744\_predicted. [Source:UniProtKB/TrEMBL;Acc:A2RRU2] | 4 |
| 1393795\_at |  | 311071 | ENSRNOG00000004677 | ENSRNOT00000006350 | Zfhx1b | zinc finger homeobox 1b [Source:RefSeq\_peptide;Acc:NP\_001028873] | 4 |
| 1369393\_at | Q63562 | 116596 | ENSRNOG00000016378 | ENSRNOT00000022190 | Map3k8 | Mitogen-activated protein kinase kinase kinase 8 (EC 2.7.11.25) (Tumor progression locus 2) (TPL-2). [Source:UniProtKB/Swiss-Prot;Acc:Q63562] | 4 |
| 1368829\_at |  | 83727 | ENSRNOG00000007302 | ENSRNOT00000010659 | Fbn1 | fibrillin 1 [Source:RefSeq\_peptide;Acc:NP\_114013] | 4 |
| 1393843\_at |  | 315745 | ENSRNOG00000023653  ENSRNOG00000007077 | ENSRNOT00000028945  ENSRNOT00000009368 | NP\_001101627.1 | feminization 1 homolog b [Source:RefSeq\_peptide;Acc:NP\_001101627] | 4 |
| 1379829\_at |  |  | ENSRNOG00000017166 | ENSRNOT00000023354 | Mycbp | c-myc binding protein Gene [Source:MGI (curated);Acc:Mycbp-001] | 4 |
| 1371133\_a\_at | P12369 | 24679 | ENSRNOG00000009079 | ENSRNOT00000012415 | Prkar2b | cAMP-dependent protein kinase type II-beta regulatory subunit. [Source:UniProtKB/Swiss-Prot;Acc:P12369] | 4 |
| 1367803\_at | P70582 | 53372 | ENSRNOG00000002247 | ENSRNOT00000003070 | Nup54 | Nucleoporin p54 (54 kDa nucleoporin). [Source:UniProtKB/Swiss-Prot;Acc:P70582] | 4 |
| 1383402\_at |  |  | ENSRNOG00000018830 | ENSRNOT00000025474 | Aff3\_predicted |  | 4 |
| 1385403\_at |  | 308871 | ENSRNOG00000020175 | ENSRNOT00000027321 | NP\_001101010.1 | ring finger protein 121 [Source:RefSeq\_peptide;Acc:NP\_001101010] | 4 |
| 1378912\_at |  |  | ENSRNOG00000016965 | ENSRNOT00000023235 | NP\_001101241.1 | anaphase promoting complex subunit 1 [Source:RefSeq\_peptide;Acc:NP\_001101241] | 4 |
| 1373293\_at |  | 288029 | ENSRNOG00000001727 | ENSRNOT00000002354  ENSRNOT00000058812  ENSRNOT00000058811 | Lsg1 | large subunit GTPase 1 homolog [Source:RefSeq\_peptide;Acc:NP\_001013439] | 4 |
| 1369788\_s\_at |  | 298247 | ENSRNOG00000026299 | ENSRNOT00000039554 | Mysm1\_predicted |  | 4 |
| 1376810\_at |  | 361046 | ENSRNOG00000032888 | ENSRNOT00000028112 | RGD1305133 | M-phase phosphoprotein, mpp8 [Source:RefSeq\_peptide;Acc:NP\_001017375] | 4 |
| 1383631\_at |  |  | ENSRNOG00000011922 | ENSRNOT00000017243 | Q7TQ74\_RAT | Ac1573. [Source:UniProtKB/TrEMBL;Acc:Q7TQ74] | 4 |
| 1392446\_at |  | 290692 | ENSRNOG00000029441 | ENSRNOT00000047223 | Klhl2\_predicted |  | 4 |
| 1384240\_at | P25095 | 24180 | ENSRNOG00000018346 | ENSRNOT00000038532 | Agtr1a | Type-1A angiotensin II receptor (AT1) (AT1A). [Source:UniProtKB/Swiss-Prot;Acc:P25095] | 4 |
| 1383119\_at | Q4KLH3 | 316290 | ENSRNOG00000014142 | ENSRNOT00000019062 | Ogfrl1 | Opioid growth factor receptor-like protein 1. [Source:UniProtKB/Swiss-Prot;Acc:Q4KLH3] | 4 |
| 1387350\_at |  | 114206 | ENSRNOG00000005851 | ENSRNOT00000008143 | Tmf1 | TATA element modulatory factor 1 [Source:RefSeq\_peptide;Acc:NP\_446123] | 4 |
| 1382396\_at |  | 293454 | ENSRNOG00000018243 | ENSRNOT00000025072 | RGD1306614 | ubiquitin-binding protein homolog [Source:RefSeq\_peptide;Acc:NP\_001030083] | 4 |
| 1388911\_at | O89044 | 301323 | ENSRNOG00000012486 | ENSRNOT00000060452  ENSRNOT00000016828 | Prim2 | DNA primase large subunit (EC 2.7.7.-) (DNA primase 58 kDa subunit) (p58). [Source:UniProtKB/Swiss-Prot;Acc:O89044] | 4 |
| 1388659\_at | Q9WU49 | 260416 | ENSRNOG00000002610 | ENSRNOT00000003514 | Carhsp1 | Calcium-regulated heat stable protein 1 (Calcium-regulated heat-stable protein of 24 kDa) (CRHSP-24). [Source:UniProtKB/Swiss-Prot;Acc:Q9WU49] | 4 |
| 1377049\_at |  | 246246 | ENSRNOG00000008190 | ENSRNOT00000042164 | Ntel1 | Patatin-like phospholipase domain-containing protein 7 (EC 3.1.1.-) (NTE-related esterase) (Liver NTE-related protein 1). [Source:UniProtKB/Swiss-Prot;Acc:Q5BK26] | 4 |
| 1375633\_at |  | 406864 | ENSRNOG00000029682 | ENSRNOT00000042972 | Clic1 | chloride intracellular channel 1 [Source:RefSeq\_peptide;Acc:NP\_001002807] | 4 |
| 1384766\_a\_at |  | 500030 | ENSRNOG00000005775 | ENSRNOT00000047029  ENSRNOT00000007707 | NP\_001103962.1 | similar to PHD finger protein 14 isoform 1 (predicted) (RGD1563764\_predicted), mRNA [Source:RefSeq\_dna;Acc:NM\_001110492] | 4 |
| 1376135\_at | Q3KRD0 | 304919 | ENSRNOG00000002813 | ENSRNOT00000003828 | Dars2 | Aspartyl-tRNA synthetase, mitochondrial precursor (EC 6.1.1.12) (Aspartate--tRNA ligase) (AspRS). [Source:UniProtKB/Swiss-Prot;Acc:Q3KRD0] | 4 |
| 1389222\_at |  |  | ENSRNOG00000011895 | ENSRNOT00000015923 | LOC691143 | Bq135360. [Source:UniProtKB/TrEMBL;Acc:Q7TP01] | 4 |
| 1391070\_at |  | 500692 | ENSRNOG00000038183 | ENSRNOT00000058127 | RGD1566220\_predicted |  | 4 |
| 1379261\_at | Q5I0J4 | 691962  287598  687897 | ENSRNOG00000025981 | ENSRNOT00000036098 | RGD1307084 | Spindle and kinetochore-associated protein 2 (Protein FAM33A). [Source:UniProtKB/Swiss-Prot;Acc:Q5I0J4] | 4 |
| 1370536\_at | O70467 | 89820 | ENSRNOG00000014829 | ENSRNOT00000050731  ENSRNOT00000020853 | Hrmt1l3 | Protein arginine N-methyltransferase 3 (EC 2.1.1.-) (Heterogeneous nuclear ribonucleoprotein methyltransferase-like protein 3). [Source:UniProtKB/Swiss-Prot;Acc:O70467] | 4 |
| 1379810\_at |  | 114632 | ENSRNOG00000011384 | ENSRNOT00000015213 | Scye1 | small inducible cytokine subfamily E, member 1 [Source:RefSeq\_peptide;Acc:NP\_446209] | 4 |
| 1373203\_at |  | 361213 | ENSRNOG00000010882 | ENSRNOT00000014546 | NP\_001101876.1 | serine palmitoyltransferase, long chain base subunit 1 [Source:RefSeq\_peptide;Acc:NP\_001101876] | 4 |
| 1372004\_at |  | 362454 | ENSRNOG00000000024 | ENSRNOT00000000025 | NP\_001102121.1 | heme binding protein 1 [Source:RefSeq\_peptide;Acc:NP\_001102121] | 4 |
| 1376368\_at | A1L131 | 294009 | ENSRNOG00000019574 | ENSRNOT00000026609 | Cuedc2\_predicted | CUE domain-containing protein 2. [Source:UniProtKB/Swiss-Prot;Acc:A1L131] | 4 |
| 1398356\_at | Q4KM65 | 291877 | ENSRNOG00000019444 | ENSRNOT00000026297 | Cpsf5 | Cleavage and polyadenylation specificity factor subunit 5 (Nucleoside diphosphate-linked moiety X motif 21) (Nudix motif 21). [Source:UniProtKB/Swiss-Prot;Acc:Q4KM65] | 4 |
| 1377608\_a\_at |  |  | ENSRNOG00000012455 | ENSRNOT00000049822  ENSRNOT00000017922 | Tardbp | TAR DNA binding protein [Source:RefSeq\_peptide;Acc:NP\_001011979] | 4 |
| 1372909\_at |  | 307302 | ENSRNOG00000017035 | ENSRNOT00000023236 | RGD1565619\_predicted |  | 4 |
| 1367602\_at |  | 114490 | ENSRNOG00000012193 | ENSRNOT00000016244 | Cited2 | Cbp/p300-interacting transactivator, with Glu/Asp-rich carboxy-terminal domain, 2 [Source:RefSeq\_peptide;Acc:NP\_446150] | 4 |
| 1367554\_at | Q9JHB9  P02780 | 25010  361725 | ENSRNOG00000020305  ENSRNOG00000023151 | ENSRNOT00000027502  ENSRNOT00000031612 | Scgb2a1  Scgb2a2 | Secretoglobin family 2A member 1 precursor (Prostatic steroid-binding protein C3.2). [Source:UniProtKB/Swiss-Prot;Acc:Q9JHB9]  Secretoglobin family 2A member 2 precursor (Prostatic steroid-binding protein C3) (C3.1) (Prostatein peptide C3). [Source:UniProtKB/Swiss-Prot;Acc:P02780] | 4 |
| 1373708\_at | Q3MHT4  Q68FR6 | 499314  293725 | ENSRNOG00000020047  ENSRNOG00000020075 | ENSRNOT00000027187  ENSRNOT00000027305 | MGC125034  Eef1g | U6 snRNA-specific terminal uridylyltransferase 1 (EC 2.7.7.52) (U6- TUTase) (RNA-binding protein 21) (RNA-binding motif protein 21). [Source:UniProtKB/Swiss-Prot;Acc:Q3MHT4]  Elongation factor 1-gamma (EF-1-gamma) (eEF-1B gamma). [Source:UniProtKB/Swiss-Prot;Acc:Q68FR6] | 4 |
| 1387786\_at | P62775 | 79215 | ENSRNOG00000011857 | ENSRNOT00000015808 | Mtpn | Myotrophin (Protein V-1) (Granule cell differentiation protein). [Source:UniProtKB/Swiss-Prot;Acc:P62775] | 4 |
| 1389315\_at |  | 304546 | ENSRNOG00000001190 | ENSRNOT00000045946 | Git2 | G protein-coupled receptor kinase-interactor 2 [Source:RefSeq\_peptide;Acc:NP\_001005553] | 4 |
| 1384110\_at |  |  | ENSRNOG00000004823 | ENSRNOT00000006709 | Dock4 | dedicator of cytokinesis 4 Gene [Source:MGI Symbol;Acc:MGI:1918006] | 4 |
| 1391413\_at |  |  | ENSRNOG00000020179 | ENSRNOT00000027351 | LOC365090 |  | 4 |
| 1371024\_at | P53565 | 116639 | ENSRNOG00000001424 | ENSRNOT00000001928 | LOC681658 | Homeobox protein cut-like 1 (CCAAT displacement protein) (CDP) (CDP2) (Fragment). [Source:UniProtKB/Swiss-Prot;Acc:P53565] | 4 |
| 1371644\_at | Q5RJR2 | 315265 | ENSRNOG00000022507 | ENSRNOT00000038032 | Ptk9 | Twinfilin-1. [Source:UniProtKB/Swiss-Prot;Acc:Q5RJR2] | 4 |
| 1369036\_at | P42260 | 54257 | ENSRNOG00000000368 | ENSRNOT00000000415 | Grik2 | Glutamate receptor, ionotropic kainate 2 precursor (Glutamate receptor 6) (GluR-6) (GluR6). [Source:UniProtKB/Swiss-Prot;Acc:P42260] | 4 |
| 1371044\_at | O08593 | 81744 | ENSRNOG00000013048 | ENSRNOT00000043859 | Pde7a | High affinity cAMP-specific 3',5'-cyclic phosphodiesterase 7A (EC 3.1.4.17) (Rolipram-insensitive phosphodiesterase type 7) (Fragment). [Source:UniProtKB/Swiss-Prot;Acc:O08593] | 4 |
| 1371172\_at |  |  | ENSRNOG00000017798 | ENSRNOT00000042813 | Atp2b3 | Plasma membrane calcium-transporting ATPase 3 (EC 3.6.3.8) (PMCA3) (Plasma membrane calcium pump isoform 3) (Plasma membrane calcium ATPase isoform 3). [Source:UniProtKB/Swiss-Prot;Acc:Q64568] | 4 |
| 1384125\_at |  | 311968 | ENSRNOG00000021614 | ENSRNOT00000014652  ENSRNOT00000038978 | Mll5 | Mll5 protein (Fragment). [Source:UniProtKB/TrEMBL;Acc:Q5BJY5] | 4 |
| 1376963\_at |  | 314862 | ENSRNOG00000007821 | ENSRNOT00000010283 | NP\_001101570.1 | dual-specificity tyrosine-(Y)-phosphorylation regulated kinase 2 [Source:RefSeq\_peptide;Acc:NP\_001101570] | 4 |
| 1367683\_at |  | 85245 | ENSRNOG00000040190  ENSRNOG00000030058  ENSRNOG00000002034  ENSRNOG00000037940  ENSRNOG00000015329 | ENSRNOT00000061856  ENSRNOT00000040329  ENSRNOT00000041274  ENSRNOT00000002781  ENSRNOT00000020795 | Kpna2 | karyopherin (importin) alpha 2 [Source:RefSeq\_peptide;Acc:NP\_445935] | 4 |
| 1368079\_at | Q63065 | 116551 | ENSRNOG00000001517 | ENSRNOT00000002072 | Pdk1 | [Pyruvate dehydrogenase [lipoamide]] kinase isozyme 1, mitochondrial precursor (EC 2.7.11.2) (Pyruvate dehydrogenase kinase isoform 1) (PDK p48). [Source:UniProtKB/Swiss-Prot;Acc:Q63065] | 4 |
| 1383870\_at |  | 497986 | ENSRNOG00000007823 | ENSRNOT00000010527 | RGD1562142\_predicted |  | 4 |
| 1396035\_at |  | 297757 | ENSRNOG00000007258 | ENSRNOT00000009530 | RGD1559717\_predicted |  | 4 |
| 1389133\_at |  | 298786 | ENSRNOG00000005856 | ENSRNOT00000007919 | NP\_001100178.1 | solute carrier family 30 (zinc transporter), member 6 [Source:RefSeq\_peptide;Acc:NP\_001100178] | 4 |
| 1367648\_at | P12843 | 25662 | ENSRNOG00000016957 | ENSRNOT00000023068  ENSRNOT00000023088 | Igfbp2 | Insulin-like growth factor-binding protein 2 precursor (IGFBP-2) (IBP- 2) (IGF-binding protein 2) (BRL-BP). [Source:UniProtKB/Swiss-Prot;Acc:P12843] | 4 |
| 1370650\_s\_at |  | 25245 | ENSRNOG00000004454 | ENSRNOT00000005900 | RGD1308470 | B2 bradykinin receptor (BK-2 receptor) (B2R). [Source:UniProtKB/Swiss-Prot;Acc:P25023] | 4 |
| 1388626\_at |  | 296162 | ENSRNOG00000021232 | ENSRNOT00000030192 | NP\_001099982.1 |  | 4 |
| 1390147\_at |  |  | ENSRNOG00000014664 | ENSRNOT00000061385  ENSRNOT00000061384 | 1110037F02Rik | RIKEN cDNA 1110037F02 gene Gene [Source:MGI Symbol;Acc:MGI:1913435] | 4 |
| 1390021\_at | Q00715 | 691488  684797  64647  306945  687023  682558  680403  295278 | ENSRNOG00000021198 | ENSRNOT00000028779 | H2B1\_RAT | Histone H2B type 1. [Source:UniProtKB/Swiss-Prot;Acc:Q00715] | 4 |
| 1391564\_at |  | 363022 | ENSRNOG00000020356 | ENSRNOT00000027587 | RGD1564241\_predicted |  | 4 |
| 1393890\_at |  | 499818 | ENSRNOG00000024990 | ENSRNOT00000034769 | NP\_001102670.1 | similar to protein phosphatase 1, regulatory (inhibitor) subunit 1C (predicted) (RGD1562095\_predicted), mRNA [Source:RefSeq\_dna;Acc:NM\_001109200] | 4 |
| 1395146\_at |  | 312670 | ENSRNOG00000007990 | ENSRNOT00000010556 | Adipor2 | adiponectin receptor 2 [Source:RefSeq\_peptide;Acc:NP\_001033068] | 4 |
| 1388131\_at | Q3KRE8 | 291081 | ENSRNOG00000017445 | ENSRNOT00000023582 | Tubb2b | Tubulin beta-2B chain (T beta-15). [Source:UniProtKB/Swiss-Prot;Acc:Q3KRE8] | 5 |
| 1367676\_at | P52925 | 682545  498988  680704  29395  498072  498388 | ENSRNOG00000033321  ENSRNOG00000030313  ENSRNOG00000013167  ENSRNOG00000029107 | ENSRNOT00000046874  ENSRNOT00000040944  ENSRNOT00000017635  ENSRNOT00000040676 | RGD1559962\_predicted  Hmgb2  RGD1561694\_predicted | High mobility group protein B2 (High mobility group protein 2) (HMG- 2). [Source:UniProtKB/Swiss-Prot;Acc:P52925] | 5 |
| 1367986\_at | Q62786 | 29602 | ENSRNOG00000015655 | ENSRNOT00000021162 | Ptgfrn | Prostaglandin F2 receptor negative regulator precursor (Prostaglandin F2-alpha receptor regulatory protein) (Prostaglandin F2-alpha receptor-associated protein) (CD315 antigen). [Source:UniProtKB/Swiss-Prot;Acc:Q62786] | 5 |
| 1372693\_at |  |  | ENSRNOG00000036839 | ENSRNOT00000055284 | Hnrpa1 | Heterogeneous nuclear ribonucleoprotein A1 (Helix-destabilizing protein) (Single-strand RNA-binding protein) (hnRNP core protein A1) (HDP). [Source:UniProtKB/Swiss-Prot;Acc:P04256] | 5 |
| 1399004\_at |  | 679572  683563 | ENSRNOG00000019823 | ENSRNOT00000026856 | LOC683563 |  | 5 |
| 1372685\_at |  |  | ENSRNOG00000009785 | ENSRNOT00000012985 | Cdkn3 | cyclin-dependent kinase inhibitor 3 Gene [Source:MGI Symbol;Acc:MGI:1919641] | 5 |
| 1368412\_a\_at |  | 50677 | ENSRNOG00000006231 | ENSRNOT00000040576  ENSRNOT00000043505 | Ptpro | protein tyrosine phosphatase, receptor type, O [Source:RefSeq\_peptide;Acc:NP\_059032] | 5 |
| 1370249\_at | P16257 | 24230 | ENSRNOG00000010549 | ENSRNOT00000014089 | Bzrp | Translocator protein (Peripheral-type benzodiazepine receptor) (PBR) (PKBS) (Mitochondrial benzodiazepine receptor). [Source:UniProtKB/Swiss-Prot;Acc:P16257] | 5 |
| 1369268\_at | P29596 | 25389 | ENSRNOG00000003745 | ENSRNOT00000005085 | Atf3 | Cyclic AMP-dependent transcription factor ATF-3 (Activating transcription factor 3) (Liver regeneration factor 1) (LRF-1). [Source:UniProtKB/Swiss-Prot;Acc:P29596] | 5 |
| 1384530\_at |  |  | ENSRNOG00000005568 | ENSRNOT00000007369 | 1810048J11Rik | RIKEN cDNA 1810048J11 gene Gene [Source:MGI Symbol;Acc:MGI:1914958] | 5 |
| 1389873\_at |  | 282817 | ENSRNOG00000019675 | ENSRNOT00000026699 | Pycard | PYD and CARD domain containing [Source:RefSeq\_peptide;Acc:NP\_758825] | 5 |
| 1376100\_at |  | 307351 | ENSRNOG00000018371 | ENSRNOT00000024947 | Tubb6 | tubulin, beta 6 [Source:RefSeq\_peptide;Acc:NP\_001020846] | 5 |
| 1374449\_at | Q68FW2 | 297594 | ENSRNOG00000015529 | ENSRNOT00000020803 | Cdca3 | Cell division cycle-associated protein 3 (Trigger of mitotic entry protein 1) (TOME-1). [Source:UniProtKB/Swiss-Prot;Acc:Q68FW2] | 5 |
| 1387029\_at |  | 155012 | ENSRNOG00000030715 | ENSRNOT00000060111  ENSRNOT00000017749 | Cfh | complement component factor H [Source:RefSeq\_peptide;Acc:NP\_569093] | 5 |
| 1399023\_at | Q80ZG1 | 293614 | ENSRNOG00000013256 | ENSRNOT00000018470 | Ric8a | Synembryn-A (Protein Ric-8A). [Source:UniProtKB/Swiss-Prot;Acc:Q80ZG1] | 5 |
| 1372056\_at |  | 316035 | ENSRNOG00000010951 | ENSRNOT00000014585 | Cmtm6 | CKLF-like MARVEL transmembrane domain containing 6 [Source:RefSeq\_peptide;Acc:NP\_001007803] | 5 |
| 1368754\_at | Q63371 | 117264 | ENSRNOG00000019270 | ENSRNOT00000050227  ENSRNOT00000026072 | P2ry6 | P2Y purinoceptor 6 (P2Y6). [Source:UniProtKB/Swiss-Prot;Acc:Q63371] | 5 |
| 1388353\_at |  | 288778 | ENSRNOG00000004904 | ENSRNOT00000006578 | Pa2g4 | ErbB3-binding protein 1 [Source:RefSeq\_peptide;Acc:NP\_001004206] | 5 |
| 1391972\_a\_at |  | 304655 | ENSRNOG00000007582 | ENSRNOT00000010050 | NP\_001100633.1 | zinc finger, SWIM domain containing 4 [Source:RefSeq\_peptide;Acc:NP\_001100633] | 5 |
| 1389312\_at | Q4QQR9 | 298787 | ENSRNOG00000006340 | ENSRNOT00000008687 | RGD1309929 | Protein MEMO1 (Mediator of ErbB2-driven cell motility 1) (Protein memo). [Source:UniProtKB/Swiss-Prot;Acc:Q4QQR9] | 5 |
| 1374204\_at |  | 303336 | ENSRNOG00000012929 | ENSRNOT00000017949 | Wsb1 | WD repeat and SOCS box-containing 1 isoform 1 [Source:RefSeq\_peptide;Acc:NP\_001036026] | 5 |
| 1383952\_at |  | 294520 | ENSRNOG00000000307 | ENSRNOT00000000337  ENSRNOT00000057162 | NP\_001099867.1 | microtubule associated monoxygenase, calponin and LIM domain containing 1 [Source:RefSeq\_peptide;Acc:NP\_001099867] | 5 |
| 1382058\_at |  | 365355 | ENSRNOG00000012258 | ENSRNOT00000017199 | Rras2 | related RAS viral (r-ras) oncogene homolog 2 [Source:RefSeq\_peptide;Acc:NP\_001013452] | 5 |
| 1387221\_at | P22288 | 29244 | ENSRNOG00000011039 | ENSRNOT00000014821 | Gch | GTP cyclohydrolase I precursor (EC 3.5.4.16) (GTP-CH-I). [Source:UniProtKB/Swiss-Prot;Acc:P22288] | 5 |
| 1373197\_at |  |  | ENSRNOG00000008194 | ENSRNOT00000010817 | RGD1306344 | similar to Ab1-133 (RGD1306344), mRNA [Source:RefSeq\_dna;Acc:NM\_001047860] | 5 |
| 1385605\_at |  | 688730  684132 | ENSRNOG00000036834 | ENSRNOT00000055279 | LOC684132 | similar to G protein-coupled receptor 84 (LOC688730), mRNA [Source:RefSeq\_dna;Acc:NM\_001109509] | 5 |
| 1368558\_s\_at | P55009  P55007 | 29427 | ENSRNOG00000000853 | ENSRNOT00000001135  ENSRNOT00000001138 | Aif1 | Allograft inflammatory factor 1 (AIF-1) (Ionized calcium-binding adapter molecule 1) (Microglia response factor) (MRF-1). [Source:UniProtKB/Swiss-Prot;Acc:P55009] | 5 |
| 1392479\_at | Q62658 | 25639 | ENSRNOG00000008822 | ENSRNOT00000012608 | Fkbp1a | FK506-binding protein 1A (EC 5.2.1.8) (Peptidyl-prolyl cis-trans isomerase) (PPIase) (Rotamase) (12 kDa FKBP) (FKBP-12) (Immunophilin FKBP12). [Source:UniProtKB/Swiss-Prot;Acc:Q62658] | 5 |
| 1390767\_at |  | 361233 | ENSRNOG00000014165 | ENSRNOT00000060226  ENSRNOT00000019040 | Ssr1 | Translocon-associated protein subunit alpha precursor (TRAP-alpha) (Signal sequence receptor subunit alpha) (SSR-alpha) (Liver regeneration-related protein LRRG137). [Source:UniProtKB/Swiss-Prot;Acc:Q7TPJ0] | 5 |
| 1376390\_at |  | 361735 | ENSRNOG00000020991 | ENSRNOT00000033795 | Ms4a11\_predicted |  | 5 |
| 1384060\_at |  | 84598 | ENSRNOG00000011157 | ENSRNOT00000015438 | Jak1 | Tyrosine-protein kinase (EC 2.7.10.2) (Fragment). [Source:UniProtKB/TrEMBL;Acc:O35803] | 5 |
| 1372857\_at |  | 124461 | ENSRNOG00000009756 | ENSRNOT00000042978 | Pacsin2 | Protein kinase C and casein kinase substrate in neurons 2 protein (Synaptic dynamin-associated protein II) (Syndapin 2) (Syndapin-II) (SdpII). [Source:UniProtKB/Swiss-Prot;Acc:Q9QY17] | 5 |
| 1390659\_at | P26051 | 25406 | ENSRNOG00000006094 | ENSRNOT00000009000  ENSRNOT00000008760  ENSRNOT00000009073 | Cd44 | CD44 antigen precursor (Phagocytic glycoprotein 1) (PGP-1) (HUTCH-I) (Extracellular matrix receptor III) (ECMR-III) (GP90 lymphocyte homing/adhesion receptor) (Hermes antigen) (Hyaluronate receptor). [Source:UniProtKB/Swiss-Prot;Acc:P26051] | 5 |
| 1389129\_at |  | 303002 | ENSRNOG00000032152 | ENSRNOT00000027467  ENSRNOT00000027439 | RGD1308952 | Peroxisomal 2,4-dienoyl-CoA reductase (EC 1.3.1.34) (2,4-dienoyl-CoA reductase 2) (DCR-AKL) (pVI-AKL). [Source:UniProtKB/Swiss-Prot;Acc:Q9Z2M4] | 5 |
| 1395248\_at |  | 297504 | ENSRNOG00000007944 | ENSRNOT00000010810 | RGD1563633\_predicted |  | 5 |
| 1369964\_at | Q91ZN1 | 155151 | ENSRNOG00000019430 | ENSRNOT00000026496 | Coro1a | Coronin-1A (Coronin-like protein A) (Tryptophan aspartate-containing coat protein) (TACO). [Source:UniProtKB/Swiss-Prot;Acc:Q91ZN1] | 5 |
| 1387687\_at |  | 171064 | ENSRNOG00000017277 | ENSRNOT00000023262 | Igsf6 | immunoglobulin superfamily, member 6 [Source:RefSeq\_peptide;Acc:NP\_598226] | 5 |
| 1368539\_at | O08562 | 78956 | ENSRNOG00000006639 | ENSRNOT00000009668 | Scn9a | Sodium channel protein type 9 subunit alpha (Sodium channel protein type IX subunit alpha) (Voltage-gated sodium channel subunit alpha Nav1.7) (Peripheral sodium channel 1) (PN1). [Source:UniProtKB/Swiss-Prot;Acc:O08562] | 5 |
| 1370891\_at | P10252 | 245962 | ENSRNOG00000004737 | ENSRNOT00000006306 | Cd48 | CD48 antigen precursor (MRC OX-45 surface antigen) (BCM1 surface antigen) (BLAST-1). [Source:UniProtKB/Swiss-Prot;Acc:P10252] | 5 |
| 1370001\_at | P35280 | 117103 | ENSRNOG00000014621 | ENSRNOT00000020748 | Rab8a | Ras-related protein Rab-8A. [Source:UniProtKB/Swiss-Prot;Acc:P35280] | 5 |
| 1383899\_at | Q62940 | 25489 | ENSRNOG00000006683 | ENSRNOT00000009747 | Nedd4 | E3 ubiquitin-protein ligase NEDD4 (EC 6.3.2.-). [Source:UniProtKB/Swiss-Prot;Acc:Q62940] | 5 |
| 1373171\_at |  |  | ENSRNOG00000016249 | ENSRNOT00000021820 | Q4G043\_RAT | Ccdc21 protein (Fragment). [Source:UniProtKB/TrEMBL;Acc:Q4G043] | 5 |
| 1377623\_at |  | 299613 | ENSRNOG00000030830 | ENSRNOT00000040170 | Mex3d | mex3 homolog D (C. elegans) Gene [Source:MGI (curated);Acc:Mex3d-001] | 5 |
| 1383435\_at | Q9JK00 | 245956 | ENSRNOG00000006937 | ENSRNOT00000009367 | Scn3b | Sodium channel subunit beta-3 precursor. [Source:UniProtKB/Swiss-Prot;Acc:Q9JK00] | 5 |
| 1367925\_at | Q62667 | 64681 | ENSRNOG00000020182 | ENSRNOT00000027360 | Mvp | Major vault protein (MVP). [Source:UniProtKB/Swiss-Prot;Acc:Q62667] | 5 |
| 1388628\_at | Q6AY25 | 300888 | ENSRNOG00000013889 | ENSRNOT00000018603 | Tmed3 | Transmembrane emp24 domain-containing protein 3 precursor. [Source:UniProtKB/Swiss-Prot;Acc:Q6AY25] | 5 |
| 1388158\_at | Q63413 | 114612 | ENSRNOG00000000841 | ENSRNOT00000001115 | Bat1a | Spliceosome RNA helicase Bat1 (EC 3.6.1.-) (DEAD box protein UAP56) (56 kDa U2AF65-associated protein) (ATP-dependent RNA helicase p47). [Source:UniProtKB/Swiss-Prot;Acc:Q63413] | 5 |
| 1379365\_at |  | 305236 | ENSRNOG00000022298 | ENSRNOT00000031667 | Cxcl11 | chemokine (C-X-C motif) ligand 11 [Source:RefSeq\_peptide;Acc:NP\_891997] | 5 |
| 1383251\_at |  | 290027 | ENSRNOG00000008892 | ENSRNOT00000011840 | NP\_001099500.1 | poly (ADP-ribose) polymerase family, member 2 [Source:RefSeq\_peptide;Acc:NP\_001099500] | 5 |
| 1389581\_at | Q66H70 | 361749 | ENSRNOG00000016456 | ENSRNOT00000022056 | RGD1311155 | Interleukin-33 precursor (IL-33). [Source:UniProtKB/Swiss-Prot;Acc:Q66H70] | 5 |
| 1368308\_at | P09416 | 24577 | ENSRNOG00000004500 | ENSRNOT00000006188 | Myc | Myc proto-oncogene protein (c-Myc) (Transcription factor p64). [Source:UniProtKB/Swiss-Prot;Acc:P09416] | 5 |
| 1390707\_at | P49806 | 54290 | ENSRNOG00000020271 | ENSRNOT00000027375 | Rgs10 | Regulator of G-protein signaling 10 (RGS10) (Fragment). [Source:UniProtKB/Swiss-Prot;Acc:P49806] | 5 |
| 1386879\_at | P08699 | 83781 | ENSRNOG00000010645 | ENSRNOT00000014216 | Lgals3 | Galectin-3 (Galactose-specific lectin 3) (Mac-2 antigen) (IgE-binding protein) (35 kDa lectin) (Carbohydrate-binding protein 35) (CBP 35) (Laminin-binding protein) (Lectin L-29). [Source:UniProtKB/Swiss-Prot;Acc:P08699] | 5 |
| 1367574\_at | P31000 | 81818 | ENSRNOG00000018087 | ENSRNOT00000024430 | Vim | Vimentin. [Source:UniProtKB/Swiss-Prot;Acc:P31000] | 5 |
| 1389494\_at |  | 361715 | ENSRNOG00000021117 | ENSRNOT00000028678 | NP\_001101987.1 | ribosomal protein S6 kinase, polypeptide 4 [Source:RefSeq\_peptide;Acc:NP\_001101987] | 5 |
| 1369186\_at | P43527 | 25166 | ENSRNOG00000007372 | ENSRNOT00000009993 | Casp1 | Caspase-1 precursor (EC 3.4.22.36) (CASP-1) (Interleukin-1 beta convertase) (IL-1BC) (IL-1 beta-converting enzyme) (ICE) (Interleukin- 1 beta-converting enzyme) (p45) [Contains: Caspase-1 subunit p20; Caspase-1 subunit p10]. [Source:UniProtKB/Swiss-Prot;Acc:P43527] | 5 |
| 1371447\_at |  | 360914 | ENSRNOG00000002217 | ENSRNOT00000003012 | NP\_001101823.1 | placenta-specific 8 [Source:RefSeq\_peptide;Acc:NP\_001101823] | 5 |
| 1393217\_at |  | 289453 | ENSRNOG00000030216 | ENSRNOT00000061238  ENSRNOT00000042532  ENSRNOT00000061237 | Abcg3 | ATP-binding cassette, sub-family G (WHITE), member 3 [Source:RefSeq\_peptide;Acc:NP\_001004076] | 5 |
| 1376144\_at |  | 303905 | ENSRNOG00000023463 | ENSRNOT00000030975 | Parp9\_predicted | poly (ADP-ribose) polymerase family, member 9 [Source:RefSeq\_peptide;Acc:NP\_001096821] | 5 |
| 1398561\_at |  | 290749 | ENSRNOG00000009824 | ENSRNOT00000013118 | Irf2\_predicted | interferon regulatory factor 2 [Source:RefSeq\_peptide;Acc:NP\_001040551] | 5 |
| 1370177\_at |  | 25066 | ENSRNOG00000019202 | ENSRNOT00000043096 | PVR | poliovirus receptor (PVR), mRNA [Source:RefSeq\_dna;Acc:NM\_017076] | 5 |
| 1399034\_at |  |  | ENSRNOG00000007459 | ENSRNOT00000058217 | Pcnx | Pecanex 1 (Fragment). [Source:UniProtKB/TrEMBL;Acc:Q5G7W4] | 5 |
| 1373769\_at |  | 289632 | ENSRNOG00000002185 | ENSRNOT00000002977 | Pgm2 | phosphoglucomutase 2 [Source:RefSeq\_peptide;Acc:NP\_001099477] | 5 |
| 1369018\_at |  | 58921  683559  689619 | ENSRNOG00000005936  ENSRNOG00000028279 | ENSRNOT00000008003  ENSRNOT00000055890 | Foxm1  LOC689619 | Forkhead box protein M1 (Winged-helix factor from INS-1 cells) (INS-1 winged helix). [Source:UniProtKB/Swiss-Prot;Acc:P97691] | 5 |
| 1368421\_at | P35234 | 29644 | ENSRNOG00000013981 | ENSRNOT00000018860 | Ptpn5 | Tyrosine-protein phosphatase non-receptor type 5 (EC 3.1.3.48) (Protein-tyrosine phosphatase striatum-enriched) (STEP) (Neural- specific protein-tyrosine phosphatase). [Source:UniProtKB/Swiss-Prot;Acc:P35234] | 5 |
| 1371777\_at |  | 298510 | ENSRNOG00000028592  ENSRNOG00000015642 | ENSRNOT00000032308  ENSRNOT00000056592 | AL596386.16  Pabpc4 | Putative uncharacterized protein Fragment [Source:UniProtKB/TrEMBL;Acc:Q3UTA6]  Pabpc4 protein (Fragment). [Source:UniProtKB/TrEMBL;Acc:Q5I0G7] | 5 |
| 1377034\_at | Q4G075 | 291091 | ENSRNOG00000016581 | ENSRNOT00000059854  ENSRNOT00000045038  ENSRNOT00000059853  ENSRNOT00000059852  ENSRNOT00000059851  ENSRNOT00000059850  ENSRNOT00000022503  ENSRNOT00000059849  ENSRNOT00000059848 | Serpinb1a | Leukocyte elastase inhibitor A (Serpin B1a) (Serine protease inhibitor EIA). [Source:UniProtKB/Swiss-Prot;Acc:Q4G075] | 5 |
| 1382346\_at | Q07014 | 81515 | ENSRNOG00000008180 | ENSRNOT00000011130 | Lyn | Tyrosine-protein kinase Lyn (EC 2.7.10.2). [Source:UniProtKB/Swiss-Prot;Acc:Q07014] | 5 |
| 1387113\_at | Q9EQH5 |  | ENSRNOG00000017326 | ENSRNOT00000054901  ENSRNOT00000023574 | Ctbp2 | C-terminal-binding protein 2 (CtBP2). [Source:UniProtKB/Swiss-Prot;Acc:Q9EQH5] | 5 |
| 1374778\_at | P80067 | 25423 | ENSRNOG00000016496 | ENSRNOT00000022342 | Ctsc | Dipeptidyl-peptidase 1 precursor (EC 3.4.14.1) (Dipeptidyl-peptidase I) (DPP-I) (DPPI) (Cathepsin C) (Cathepsin J) (Dipeptidyl transferase) [Contains: Dipeptidyl-peptidase 1 exclusion domain chain (Dipeptidyl- peptidase I exclusion domain chain); Dipeptid [Source:UniProtKB/Swiss-Prot;Acc:P80067] | 5 |
| 1382492\_a\_at | Q6MGB5 | 361802 | ENSRNOG00000000466 | ENSRNOT00000000542 | Hsd17b8 | Estradiol 17-beta-dehydrogenase 8 (EC 1.1.1.62) (Testosterone 17-beta- dehydrogenase 8) (EC 1.1.1.63) (17-beta-HSD 8) (17-beta-hydroxysteroid dehydrogenase 8). [Source:UniProtKB/Swiss-Prot;Acc:Q6MGB5] | 5 |
| 1390268\_at | Q5U316 | 288700 | ENSRNOG00000022014 | ENSRNOT00000030031 | Rab35 | Ras-related protein Rab-35. [Source:UniProtKB/Swiss-Prot;Acc:Q5U316] | 5 |
| 1383060\_at | Q5XIG5 | 361202 | ENSRNOG00000019272 | ENSRNOT00000026070 | Gkap1 | G kinase-anchoring protein 1. [Source:UniProtKB/Swiss-Prot;Acc:Q5XIG5] | 5 |
| 1387922\_at |  | 171547 | ENSRNOG00000016752 | ENSRNOT00000023204 | Crispld2 | cysteine-rich secretory protein LCCL domain containing 2 [Source:RefSeq\_peptide;Acc:NP\_612527] | 5 |
| 1367815\_at | O70247 | 170551 | ENSRNOG00000006556 | ENSRNOT00000009042 | Slc5a6 | Sodium-dependent multivitamin transporter (Na(+)-dependent multivitamin transporter) (Solute carrier family 5 member 6). [Source:UniProtKB/Swiss-Prot;Acc:O70247] | 5 |
| 1388679\_at |  | 360956 | ENSRNOG00000006400 | ENSRNOT00000058414  ENSRNOT00000008579 | Tbc1d14 | TBC1 domain family, member 14 isoform 2 [Source:RefSeq\_peptide;Acc:NP\_001029193] | 5 |
| 1368037\_at | P47727 | 29224 | ENSRNOG00000032165  ENSRNOG00000032830 | ENSRNOT00000041838  ENSRNOT00000042283 | Cbr1  Q9QZI6\_RAT | Carbonyl reductase [NADPH] 1 (EC 1.1.1.184) (NADPH-dependent carbonyl reductase 1). [Source:UniProtKB/Swiss-Prot;Acc:P47727]  Carbonyl reductase isoform I (Fragment). [Source:UniProtKB/TrEMBL;Acc:Q9QZI6] | 5 |
| 1367859\_at | Q07258 | 25717 | ENSRNOG00000009867 | ENSRNOT00000013516 | Tgfb3 | Transforming growth factor beta-3 precursor (TGF-beta-3). [Source:UniProtKB/Swiss-Prot;Acc:Q07258] | 5 |
| 1370826\_at | Q9Z2G8 | 89825 | ENSRNOG00000003890 | ENSRNOT00000005286 | Nap1l1 | Nucleosome assembly protein 1-like 1 (NAP-1-related protein). [Source:UniProtKB/Swiss-Prot;Acc:Q9Z2G8] | 5 |
| 1373674\_at |  | 362429 | ENSRNOG00000015505 | ENSRNOT00000020812 | NP\_001102114.1 | microfibrillar associated protein 5 [Source:RefSeq\_peptide;Acc:NP\_001102114] | 5 |
| 1389078\_at |  | 362941 | ENSRNOG00000025497 | ENSRNOT00000035199 | Fbxl6 | F-box and leucine-rich repeat protein 6 [Source:RefSeq\_peptide;Acc:NP\_001005563] | 5 |
| 1389373\_at | P97588 | 25671 | ENSRNOG00000018483 | ENSRNOT00000025079 | Smad1 | Mothers against decapentaplegic homolog 1 (SMAD 1) (Mothers against DPP homolog 1). [Source:UniProtKB/Swiss-Prot;Acc:P97588] | 5 |
| 1379425\_at |  | 313563 | ENSRNOG00000037432 | ENSRNOT00000056644 | NP\_001101443.1 | similar to RIKEN cDNA 3110037I16 (predicted) (RGD1309802\_predicted), mRNA [Source:RefSeq\_dna;Acc:NM\_001107973] | 5 |
| 1372032\_at | Q04970 | 24605 | ENSRNOG00000023079 | ENSRNOT00000039572 | Nras | GTPase NRas precursor (Transforming protein N-Ras). [Source:UniProtKB/Swiss-Prot;Acc:Q04970] | 5 |
| 1378140\_at | Q5BK71 | 364396 | ENSRNOG00000014653 | ENSRNOT00000019646 | Arl11 | ADP-ribosylation factor-like protein 11. [Source:UniProtKB/Swiss-Prot;Acc:Q5BK71] | 5 |
| 1374600\_at | P62804 | 679983  291152  684621  500351  679840  682518  684686  684887  64627  680097  680430  295277  502913  684745 | ENSRNOG00000030142 | ENSRNOT00000043151 | Hist1h4b | Histone H4 [Contains: Osteogenic growth peptide (OGP)]. [Source:UniProtKB/Swiss-Prot;Acc:P62804] | 5 |
| 1370055\_at | Q63942 | 140665 | ENSRNOG00000011582 | ENSRNOT00000015609 | Rab3d | GTP-binding protein Rab-3D. [Source:UniProtKB/Swiss-Prot;Acc:Q63942] | 5 |
| 1368405\_at | P63322 | 81757 | ENSRNOG00000013454 | ENSRNOT00000018190 | Rala | Ras-related protein Ral-A precursor. [Source:UniProtKB/Swiss-Prot;Acc:P63322] | 5 |
| 1370213\_at | P62961 | 686530  500538 | ENSRNOG00000006596  ENSRNOG00000030807  ENSRNOG00000023786  ENSRNOG00000032902 | ENSRNOT00000008641  ENSRNOT00000011205  ENSRNOT00000058700  ENSRNOT00000015875  ENSRNOT00000030974  ENSRNOT00000050213 | LOC686530  Q3ZAV2\_RAT  Ybx1 | Ybx1 protein. [Source:UniProtKB/TrEMBL;Acc:Q3ZAV2]  Nuclease sensitive element-binding protein 1 (Y-box-binding protein 1) (Y-box transcription factor) (YB-1) (CCAAT-binding transcription factor I subunit A) (CBF-A) (Enhancer factor I subunit A) (EFI-A) (DNA-binding protein B) (DBPB). [Source:UniProtKB/Swiss-Prot;Acc:P62961] | 5 |
| 1370949\_at | P30009 | 681252  294446 | ENSRNOG00000000579 | ENSRNOT00000000707 | LOC294446 | Myristoylated alanine-rich C-kinase substrate (MARCKS) (Protein kinase C substrate 80 kDa protein). [Source:UniProtKB/Swiss-Prot;Acc:P30009] | 5 |
| 1387893\_at | Q6P6T1 | 192262 | ENSRNOG00000011971 | ENSRNOT00000016330  ENSRNOT00000056058 | C1s | Complement C1s subcomponent precursor (EC 3.4.21.42) (C1 esterase) [Contains: Complement C1s subcomponent heavy chain; Complement C1s subcomponent light chain]. [Source:UniProtKB/Swiss-Prot;Acc:Q6P6T1] | 5 |
| 1373025\_at | P31722 | 362634 | ENSRNOG00000012804 | ENSRNOT00000017065 | C1qg | Complement C1q subcomponent subunit C precursor. [Source:UniProtKB/Swiss-Prot;Acc:P31722] | 5 |
| 1371769\_at |  | 65168 | ENSRNOG00000019136 | ENSRNOT00000025842 | Scamp2 | secretory carrier membrane protein 2 [Source:RefSeq\_peptide;Acc:NP\_076445] | 5 |
| 1388337\_at |  | 290029 | ENSRNOG00000009982 | ENSRNOT00000013582 | NP\_001099501.1 | nucleoside phosphorylase [Source:RefSeq\_peptide;Acc:NP\_001099501] | 5 |
| 1383863\_at |  | 362176 | ENSRNOG00000009401 | ENSRNOT00000012625 | Lmo2 | LIM domain only 2 [Source:RefSeq\_peptide;Acc:NP\_001032435] | 5 |
| 1382661\_at |  |  | ENSRNOG00000028215 | ENSRNOT00000030750 | Nup160 | nucleoporin 160 Gene [Source:MGI (curated);Acc:Nup160-001] | 5 |
| 1387283\_at | P18589  P18590 | 286918 | ENSRNOG00000001963 | ENSRNOT00000002695 | Mx2 | Interferon-induced GTP-binding protein Mx3. [Source:UniProtKB/Swiss-Prot;Acc:P18590] | 5 |
| 1376674\_at |  |  | ENSRNOG00000012055 | ENSRNOT00000016281 | Man2a2 | mannosidase 2, alpha 2 Gene [Source:MGI Symbol;Acc:MGI:2150656] | 5 |
| 1368207\_at | P59647 | 60338 | ENSRNOG00000021062 | ENSRNOT00000028597 | Fxyd5 | FXYD domain-containing ion transport regulator 5 precursor. [Source:UniProtKB/Swiss-Prot;Acc:P59647] | 5 |
| 1373504\_at |  | 299783 | ENSRNOG00000026644 | ENSRNOT00000005399 | Glipr1 | GLI pathogenesis-related 1 (glioma) [Source:RefSeq\_peptide;Acc:NP\_001011987] | 5 |
| 1372941\_at | Q642A0 | 296278 | ENSRNOG00000008845 | ENSRNOT00000012141 | Pdrg1 | p53 and DNA damage-regulated protein 1. [Source:UniProtKB/Swiss-Prot;Acc:Q642A0] | 5 |
| 1385837\_at |  | 288152 | ENSRNOG00000001577 | ENSRNOT00000033511 | Hoxd3 | homeo box D3 Gene [Source:MGI (curated);Acc:Hoxd3-001] | 5 |
| 1373490\_at | Q80T18 | 113940 | ENSRNOG00000019838 | ENSRNOT00000026891 | Gmfg | Glia maturation factor gamma (GMF-gamma). [Source:UniProtKB/Swiss-Prot;Acc:Q80T18] | 5 |
| 1389615\_at |  | 689741  362912 | ENSRNOG00000005551 | ENSRNOT00000007484 | RGD1311835 | similar to RIKEN cDNA 1110021N07 (RGD1311835), mRNA [Source:RefSeq\_dna;Acc:NM\_001014202] | 5 |
| 1387342\_at | P63219 | 79218 | ENSRNOG00000015936 | ENSRNOT00000021358 | Gng5 | Guanine nucleotide-binding protein G(I)/G(S)/G(O) subunit gamma-5 precursor. [Source:UniProtKB/Swiss-Prot;Acc:P63219] | 5 |
| 1379282\_at | Q4V7E8 | 301035 | ENSRNOG00000021047 | ENSRNOT00000056060 | Lrrfip2 | Leucine-rich repeat flightless-interacting protein 2 (LRR FLII- interacting protein 2). [Source:UniProtKB/Swiss-Prot;Acc:Q4V7E8] | 5 |
| 1375091\_at |  | 363055 | ENSRNOG00000029826 | ENSRNOT00000022583 | RGD1561243\_predicted |  | 5 |
| 1398315\_at | P61314 | 245981  498143  311120  685415 | ENSRNOG00000008140  ENSRNOG00000025212  ENSRNOG00000030557  ENSRNOG00000023181 | ENSRNOT00000010759  ENSRNOT00000038919  ENSRNOT00000042779  ENSRNOT00000032528 | Rpl15    RGD1565767\_predicted | 60S ribosomal protein L15. [Source:UniProtKB/Swiss-Prot;Acc:P61314] | 5 |
| 1380546\_at |  | 298250 | ENSRNOG00000008907 | ENSRNOT00000011767 | LOC298250 | Putative uncharacterized protein LOC298250. [Source:UniProtKB/TrEMBL;Acc:Q5FVC3] | 5 |
| 1378049\_at |  | 297879 | ENSRNOG00000005886 | ENSRNOT00000007761 | RGD1562996\_predicted |  | 5 |
| 1387058\_at | P53809 | 29510 | ENSRNOG00000002425 | ENSRNOT00000003295 | Pctp | Phosphatidylcholine transfer protein (PC-TP) (StAR-related lipid transfer protein 2) (StARD2) (START domain-containing protein 2). [Source:UniProtKB/Swiss-Prot;Acc:P53809] | 5 |
| 1386987\_at | P22273 | 24499 | ENSRNOG00000020811 | ENSRNOT00000028234  ENSRNOT00000028230 | Il6ra | Interleukin-6 receptor alpha chain precursor (IL-6R-alpha) (IL-6R 1) (CD126 antigen). [Source:UniProtKB/Swiss-Prot;Acc:P22273] | 5 |
| 1389052\_at |  |  | ENSRNOG00000018884 | ENSRNOT00000038600  ENSRNOT00000025548 | Q3KR69\_RAT | Ttc13 protein (Fragment). [Source:UniProtKB/TrEMBL;Acc:Q3KR69] | 5 |
| 1392425\_x\_at |  | 360503 | ENSRNOG00000020488 | ENSRNOT00000060078 | Luc7l | LUC7-like [Source:RefSeq\_peptide;Acc:NP\_001019440] | 5 |
| 1385440\_at |  | 474146 | ENSRNOG00000020849 | ENSRNOT00000028287  ENSRNOT00000028285 | Hcst | hematopoietic cell signal transducer [Source:RefSeq\_peptide;Acc:NP\_001005900] | 5 |
| 1382206\_a\_at |  | 298024 | ENSRNOG00000011504 | ENSRNOT00000059613  ENSRNOT00000015576  ENSRNOT00000015310 | Akap2 | A kinase (PRKA) anchor protein 2 [Source:RefSeq\_peptide;Acc:NP\_001011974] | 5 |
| 1372254\_at | Q6P734 | 295703 | ENSRNOG00000007457 | ENSRNOT00000009817 | Serping1 | Plasma protease C1 inhibitor precursor (C1 Inh) (C1Inh) (C1 esterase inhibitor) (C1-inhibiting factor). [Source:UniProtKB/Swiss-Prot;Acc:Q6P734] | 5 |
| 1372886\_at |  | 360962 | ENSRNOG00000017259 | ENSRNOT00000032972  ENSRNOT00000057970  ENSRNOT00000057969  ENSRNOT00000057967 | Tacc3 | transforming, acidic coiled-coil containing protein 3 [Source:RefSeq\_peptide;Acc:NP\_001004424] | 5 |
| 1382398\_at |  | 498356 | ENSRNOG00000025670 | ENSRNOT00000036103 | NP\_001102557.1 | similar to MGC68837 protein (predicted) (RGD1565710\_predicted), mRNA [Source:RefSeq\_dna;Acc:NM\_001109087] | 5 |
| 1375008\_at |  | 292554 | ENSRNOG00000015825 | ENSRNOT00000021245 | NP\_001099691.1 | aurora kinase C [Source:RefSeq\_peptide;Acc:NP\_001099691] | 5 |
| 1392280\_at |  | 310553 | ENSRNOG00000009822 | ENSRNOT00000013025 | Tlr2 | toll-like receptor 2 [Source:RefSeq\_peptide;Acc:NP\_942064] | 5 |
| 1368420\_at | P13635 | 24268 | ENSRNOG00000011913 | ENSRNOT00000016083 | Cp | Ceruloplasmin precursor (EC 1.16.3.1) (Ferroxidase). [Source:UniProtKB/Swiss-Prot;Acc:P13635] | 5 |
| 1368238\_at | P25031 | 24618 | ENSRNOG00000006151 | ENSRNOT00000008212 | Pap | Regenerating islet-derived protein 3 beta precursor (Reg III-beta) (Pancreatitis-associated protein 1) (Peptide 23) (REG-2). [Source:UniProtKB/Swiss-Prot;Acc:P25031] | 5 |
| 1371079\_at | Q63203 | 289211 | ENSRNOG00000003138 | ENSRNOT00000035400  ENSRNOT00000058497 | Fcgr2b | Low affinity immunoglobulin gamma Fc region receptor II precursor (Fc- gamma RII) (FcRII) (IgG Fc receptor II beta) (CD32 antigen). [Source:UniProtKB/Swiss-Prot;Acc:Q63203] | 5 |
| 1394678\_at |  | 309653 | ENSRNOG00000000528 | ENSRNOT00000041665 | NP\_001101087.2 | FYVE, RhoGEF and PH domain containing 2 [Source:RefSeq\_peptide;Acc:NP\_001101087] | 5 |
| 1389566\_at |  | 363088 | ENSRNOG00000039916  ENSRNOG00000012678 | ENSRNOT00000061316  ENSRNOT00000017117 | Ccnb2 | cyclin B2 [Source:RefSeq\_peptide;Acc:NP\_001009470] | 5 |
| 1388514\_at |  | 259229 | ENSRNOG00000026905 | ENSRNOT00000031842 | Ppm1g | protein phosphatase 1G (formerly 2C), magnesium-dependent, gamma isoform [Source:RefSeq\_peptide;Acc:NP\_671742] | 5 |
| 1369456\_at | P30994 | 29581 | ENSRNOG00000017625 | ENSRNOT00000023829 | Htr2b | 5-hydroxytryptamine receptor 2B (5-HT-2B) (Serotonin receptor 2B) (5- HT-2F) (Stomach fundus serotonin receptor). [Source:UniProtKB/Swiss-Prot;Acc:P30994] | 5 |
| 1383614\_at | Q66HE5 | 289419 | ENSRNOG00000000034 | ENSRNOT00000049697  ENSRNOT00000000039 | Nuak2 | NUAK family SNF1-like kinase 2 (EC 2.7.11.1) (SNF1/AMP kinase-related kinase) (SNARK). [Source:UniProtKB/Swiss-Prot;Acc:Q66HE5] | 5 |
| 1388114\_at | P13832 |  | ENSRNOG00000015278 | ENSRNOT00000021048 | MLRA\_RAT | Myosin regulatory light chain 2-A, smooth muscle isoform (Myosin RLC- A). [Source:UniProtKB/Swiss-Prot;Acc:P13832] | 5 |
| 1374883\_at |  | 306490 | ENSRNOG00000011420 | ENSRNOT00000015625 | NP\_001100782.1 | myotubularin related protein 7 [Source:RefSeq\_peptide;Acc:NP\_001100782] | 5 |
| 1387134\_at |  |  | ENSRNOG00000021357 | ENSRNOT00000012859 | Slfn3 | schlafen 3 [Source:RefSeq\_peptide;Acc:NP\_446139] | 5 |
| 1382307\_at | Q10728 | 116670 | ENSRNOG00000004925 | ENSRNOT00000006792  ENSRNOT00000050037  ENSRNOT00000006773 | Ppp1r12a | Protein phosphatase 1 regulatory subunit 12A (Myosin phosphatase- targeting subunit 1) (Myosin phosphatase target subunit 1) (Protein phosphatase myosin-binding subunit) (MBSP) (Serine/threonine protein phosphatase PP1 smooth muscle regulatory subunit M11 [Source:UniProtKB/Swiss-Prot;Acc:Q10728] | 5 |
| 1392171\_at | Q9WTV1 | 89824 | ENSRNOG00000003312 | ENSRNOT00000060710  ENSRNOT00000004444 | Chi3l1 | Chitinase-3-like protein 1 precursor (Cartilage glycoprotein 39) (GP- 39). [Source:UniProtKB/Swiss-Prot;Acc:Q9WTV1] | 5 |
| 1388330\_at | Q6TEK4 | 309004 | ENSRNOG00000019399 | ENSRNOT00000026347 | Vkorc1 | Vitamin K epoxide reductase complex subunit 1 (EC 1.1.4.1) (Vitamin K1 2,3-epoxide reductase subunit 1). [Source:UniProtKB/Swiss-Prot;Acc:Q6TEK4] | 5 |
| 1377787\_at |  | 315997 | ENSRNOG00000018333 | ENSRNOT00000024939 | NP\_001101656.1 | similar to Rbm6 protein (predicted) (RGD1560367\_predicted), mRNA [Source:RefSeq\_dna;Acc:NM\_001108186] | 5 |
| 1367850\_at |  | 116591 | ENSRNOG00000003138  ENSRNOG00000003142 | ENSRNOT00000045427  ENSRNOT00000047349 | Fcgr2b  FCGR3\_RAT | Low affinity immunoglobulin gamma Fc region receptor II precursor (Fc- gamma RII) (FcRII) (IgG Fc receptor II beta) (CD32 antigen). [Source:UniProtKB/Swiss-Prot;Acc:Q63203]  Low affinity immunoglobulin gamma Fc region receptor III precursor (IgG Fc receptor III) (Fc-gamma RIII) (FcRIII) (CD16 antigen). [Source:UniProtKB/Swiss-Prot;Acc:P27645] | 5 |
| 1370186\_at | P28077 | 24967 | ENSRNOG00000000459 | ENSRNOT00000000532 | Psmb9 | Proteasome subunit beta type-9 precursor (EC 3.4.25.1) (Proteasome subunit beta-1i) (Proteasome chain 7) (Macropain chain 7) (Multicatalytic endopeptidase complex chain 7) (RING12 protein) (Low molecular mass protein 2). [Source:UniProtKB/Swiss-Prot;Acc:P28077] | 5 |
| 1373646\_at | P35289 | 299156 | ENSRNOG00000007364 | ENSRNOT00000010043 | Rab15 | Ras-related protein Rab-15. [Source:UniProtKB/Swiss-Prot;Acc:P35289] | 5 |
| 1389210\_at |  | 306071 | ENSRNOG00000010319 | ENSRNOT00000014502  ENSRNOT00000058275 | Lcp1 | lymphocyte cytosolic protein 1 [Source:RefSeq\_peptide;Acc:NP\_001012044] | 5 |
| 1374903\_at |  | 306860 | ENSRNOG00000023778 | ENSRNOT00000060336  ENSRNOT00000060335  ENSRNOT00000035448 | Gcnt2 | glucosaminyl (N-acetyl) transferase 2, I-branching enzyme [Source:RefSeq\_peptide;Acc:NP\_001001511] | 5 |
| 1389791\_at | Q6AYM9 | 306619 | ENSRNOG00000012565 | ENSRNOT00000016756 | Cln8 | Protein CLN8. [Source:UniProtKB/Swiss-Prot;Acc:Q6AYM9] | 5 |
| 1367776\_at | P39951 | 54237 | ENSRNOG00000000632 | ENSRNOT00000000783  ENSRNOT00000058027 | Cdc2a | Cell division control protein 2 homolog (EC 2.7.11.22) (EC 2.7.11.23) (p34 protein kinase) (Cyclin-dependent kinase 1) (CDK1). [Source:UniProtKB/Swiss-Prot;Acc:P39951] | 5 |
| 1372903\_at |  | 303575 | ENSRNOG00000021713 | ENSRNOT00000032339 | RGD1310360 | similar to 3000004C01Rik protein (RGD1310360), mRNA [Source:RefSeq\_dna;Acc:NM\_001039019] | 5 |
| 1399143\_at | Q9EQX9 | 683536  116725 | ENSRNOG00000009038 | ENSRNOT00000059025 | Ube2n | Ubiquitin-conjugating enzyme E2 N (EC 6.3.2.19) (Ubiquitin-protein ligase N) (Ubiquitin carrier protein N) (Bendless-like ubiquitin- conjugating enzyme). [Source:UniProtKB/Swiss-Prot;Acc:Q9EQX9] | 5 |
| 1367938\_at | O70199 | 83472 | ENSRNOG00000002643 | ENSRNOT00000003691 | Ugdh | UDP-glucose 6-dehydrogenase (EC 1.1.1.22) (UDP-Glc dehydrogenase) (UDP-GlcDH) (UDPGDH). [Source:UniProtKB/Swiss-Prot;Acc:O70199] | 5 |
| 1388519\_at |  | 298068 | ENSRNOG00000006345 | ENSRNOT00000008329 | NM\_001106654.1 | Sec61 beta subunit (predicted) (Sec61b\_predicted), mRNA [Source:RefSeq\_dna;Acc:NM\_001106654] | 5 |
| 1388590\_at | Q6MFY5 | 361784 | ENSRNOG00000000779 | ENSRNOT00000001012 | Znrd1 | DNA-directed RNA polymerase I subunit RPA12 (Zinc ribbon domain- containing protein 1). [Source:UniProtKB/Swiss-Prot;Acc:Q6MFY5] | 5 |
| 1374468\_at |  | 301059 | ENSRNOG00000013634 | ENSRNOT00000018341 | Myd88 | myeloid differentiation primary response gene 88 [Source:RefSeq\_peptide;Acc:NP\_937763] | 5 |
| 1398771\_at | Q794F9 | 50567 | ENSRNOG00000018487 | ENSRNOT00000025196 | Slc3a2 | 4F2 cell-surface antigen heavy chain (4F2hc). [Source:UniProtKB/Swiss-Prot;Acc:Q794F9] | 5 |
| 1376879\_at | Q569B7 | 502084 | ENSRNOG00000022500  ENSRNOG00000038178 | ENSRNOT00000035323  ENSRNOT00000058119 | Rwdd4a | RWD domain-containing protein 4. [Source:UniProtKB/Swiss-Prot;Acc:Q569B7] | 5 |
| 1368020\_at | Q62967 | 81726 | ENSRNOG00000013376 | ENSRNOT00000018145 | Mvd | Diphosphomevalonate decarboxylase (EC 4.1.1.33) (Mevalonate pyrophosphate decarboxylase) (Mevalonate (diphospho)decarboxylase). [Source:UniProtKB/Swiss-Prot;Acc:Q62967] | 5 |
| 1373432\_at | P30009 | 681252  294446 | ENSRNOG00000000579 | ENSRNOT00000000707 | LOC294446 | Myristoylated alanine-rich C-kinase substrate (MARCKS) (Protein kinase C substrate 80 kDa protein). [Source:UniProtKB/Swiss-Prot;Acc:P30009] | 5 |
| 1370509\_at | O88484 | 246311 | ENSRNOG00000012343 | ENSRNOT00000016462 | Pdp2 | [Pyruvate dehydrogenase [lipoamide]]-phosphatase 2, mitochondrial precursor (EC 3.1.3.43) (PDP 2) (Pyruvate dehydrogenase phosphatase, catalytic subunit 2) (PDPC 2). [Source:UniProtKB/Swiss-Prot;Acc:O88484] | 5 |
| 1384186\_at |  | 297504 | ENSRNOG00000007944 | ENSRNOT00000010810 | RGD1563633\_predicted |  | 5 |
| 1379380\_at |  |  | ENSRNOG00000025371 | ENSRNOT00000058495 | Spry1 | sprouty homolog 1 (Drosophila) Gene [Source:MGI (curated);Acc:Spry1-002] | 5 |
| 1367973\_at | P14844 | 24770 | ENSRNOG00000007159 | ENSRNOT00000009448 | Ccl2 | C-C motif chemokine 2 precursor (Small-inducible cytokine A2) (Monocyte chemotactic protein 1) (MCP-1) (Monocyte chemoattractant protein 1) (Immediate-early serum-responsive JE protein). [Source:UniProtKB/Swiss-Prot;Acc:P14844] | 5 |
| 1382467\_at | Q3MKQ1 | 363498 | ENSRNOG00000032729 | ENSRNOT00000042126 | LOC363498 | Protein BEX2 (Brain-expressed X-linked protein 2 homolog). [Source:UniProtKB/Swiss-Prot;Acc:Q3MKQ1] | 5 |
| 1387930\_at | P35231 | 171162 | ENSRNOG00000006360 | ENSRNOT00000008468 | Reg3a | Regenerating islet-derived protein 3 alpha precursor (Reg III-alpha) (Pancreatitis-associated protein 2) (Lithostathine 3) (Islet of Langerhans regenerating protein 3) (REG 3) (RegIII). [Source:UniProtKB/Swiss-Prot;Acc:P35231] | 5 |
| 1376502\_at | Q5RKH7 | 298851 | ENSRNOG00000009459 | ENSRNOT00000012558 | RGD1309228 | Uncharacterized protein C2orf18 homolog precursor. [Source:UniProtKB/Swiss-Prot;Acc:Q5RKH7] | 5 |
| 1370071\_at | Q920P6 | 24165 | ENSRNOG00000010265 | ENSRNOT00000014151 | Ada | Adenosine deaminase (EC 3.5.4.4) (Adenosine aminohydrolase). [Source:UniProtKB/Swiss-Prot;Acc:Q920P6] | 5 |
| 1370345\_at | P30277 | 25203 | ENSRNOG00000018635 | ENSRNOT00000025297  ENSRNOT00000031805 | Ccnb1 | G2/mitotic-specific cyclin-B1. [Source:UniProtKB/Swiss-Prot;Acc:P30277] | 5 |
| 1397437\_at |  | 316327 | ENSRNOG00000015382 | ENSRNOT00000059916  ENSRNOT00000020659 | Arid5a | AT rich interactive domain 5A (Mrf1 like) [Source:RefSeq\_peptide;Acc:NP\_001030106] | 5 |
| 1398847\_at | Q99MY2 | 94267 | ENSRNOG00000009094 | ENSRNOT00000012363 | Nudt4 | Diphosphoinositol polyphosphate phosphohydrolase 2 (EC 3.6.1.52) (DIPP-2) (rDIPP2) (Diadenosine 5',5'''-P1,P6-hexaphosphate hydrolase 2) (EC 3.6.1.-) (Nucleoside diphosphate-linked moiety X motif 4) (Nudix motif 4). [Source:UniProtKB/Swiss-Prot;Acc:Q99MY2] | 5 |
| 1372070\_at |  | 290644 | ENSRNOG00000019387 | ENSRNOT00000026225 | Ifi30 | interferon gamma inducible protein 30 [Source:RefSeq\_peptide;Acc:NP\_001025197] | 5 |
| 1387908\_at | Q9JKF8 | 64455 | ENSRNOG00000003348 | ENSRNOT00000004475 | Rasd1 | Dexamethasone-induced Ras-related protein 1 precursor. [Source:UniProtKB/Swiss-Prot;Acc:Q9JKF8] | 5 |
| 1393038\_at |  | 295279 | ENSRNOG00000021199 | ENSRNOT00000028780 | Fcgr1 | Fc receptor, IgG, high affinity I [Source:RefSeq\_peptide;Acc:NP\_001094306] | 5 |
| 1371428\_at |  | 681549  689134  499136 | ENSRNOG00000022917  ENSRNOG00000028787  ENSRNOG00000005203  ENSRNOG00000014455 | ENSRNOT00000033705  ENSRNOT00000047723  ENSRNOT00000006906  ENSRNOT00000019364 | Q6TXI0\_RAT  LOC689134    LOC499136 | LRRGT00019. [Source:UniProtKB/TrEMBL;Acc:Q6TXI0]    LRRGT00021 (LOC499136), mRNA [Source:RefSeq\_dna;Acc:NM\_001047937] | 5 |
| 1375469\_at |  |  | ENSRNOG00000009271 | ENSRNOT00000013165  ENSRNOT00000060927  ENSRNOT00000060926 | Q8K1P7\_RAT | Brahma-related protein 1 (Fragment). [Source:UniProtKB/TrEMBL;Acc:Q8K1P7] | 5 |
| 1392207\_at |  | 360503 | ENSRNOG00000020488 | ENSRNOT00000027812  ENSRNOT00000060078 | Luc7l | LUC7-like [Source:RefSeq\_peptide;Acc:NP\_001019440] | 5 |
| 1367846\_at | P05942 | 24615 | ENSRNOG00000011821 | ENSRNOT00000015958 | S100a4 | Protein S100-A4 (S100 calcium-binding protein A4) (Metastasin) (Placental calcium-binding protein) (Nerve growth factor-induced protein 42A) (P9K). [Source:UniProtKB/Swiss-Prot;Acc:P05942] | 5 |
| 1368014\_at |  | 59103 | ENSRNOG00000006320 | ENSRNOT00000045993  ENSRNOT00000008407 | Ptges | prostaglandin E synthase [Source:RefSeq\_peptide;Acc:NP\_067594] | 5 |
| 1390290\_at |  | 303076 | ENSRNOG00000005031 | ENSRNOT00000006699 | Surf6\_predicted | surfeit gene 6 (predicted) [Source:RefSeq\_peptide;Acc:NP\_001015014] | 5 |
| 1374551\_at |  | 287719 | ENSRNOG00000020678 | ENSRNOT00000028067 | Ifi35 | interferon-induced protein 35 [Source:RefSeq\_peptide;Acc:NP\_001009625] | 5 |
| 1387264\_at |  | 116491 | ENSRNOG00000020598 | ENSRNOT00000027974 | Kcnk6 | potassium channel, subfamily K, member 6 [Source:RefSeq\_peptide;Acc:NP\_446258] | 5 |
| 1389815\_at | Q8K3F3 | 259225 | ENSRNOG00000025790  ENSRNOG00000039080  ENSRNOG00000021151 | ENSRNOT00000030546  ENSRNOT00000028724  ENSRNOT00000051749 | Ppp1r14b | Protein phosphatase 1 regulatory subunit 14B (Phosphatase holoenzyme inhibitor 1) (PHI-1) (Ubiquitous PKC-potentiated PP1 inhibitor). [Source:UniProtKB/Swiss-Prot;Acc:Q8K3F3] | 5 |
| 1389778\_a\_at | Q63187 | 25562 | ENSRNOG00000010902 | ENSRNOT00000015649 | Tceb3 | Transcription elongation factor B polypeptide 3 (RNA polymerase II transcription factor SIII subunit A) (SIII p110) (Elongin-A) (EloA) (Elongin 110 kDa subunit). [Source:UniProtKB/Swiss-Prot;Acc:Q63187] | 5 |
| 1389080\_at |  | 293663 | ENSRNOG00000019664 | ENSRNOT00000026611 | Rbm4\_predicted | LOC474154 protein. [Source:UniProtKB/TrEMBL;Acc:Q5U212] | 5 |
| 1391545\_at | Q5RKG1 | 288111 | ENSRNOG00000039024 | ENSRNOT00000059676 | Ccdc52 | Coiled-coil domain-containing protein 52. [Source:UniProtKB/Swiss-Prot;Acc:Q5RKG1] | 5 |
| 1369720\_at | Q05096 | 117057 | ENSRNOG00000015227 | ENSRNOT00000051190  ENSRNOT00000034652  ENSRNOT00000045723 | Myo1b | Myosin-Ib (Myosin I alpha) (MMI-alpha) (MMIa) (Myosin heavy chain myr 1). [Source:UniProtKB/Swiss-Prot;Acc:Q05096] | 5 |
| 1369497\_at |  | 24906 | ENSRNOG00000002820 | ENSRNOT00000003797 | LOC24906 | RoBo-1 [Source:RefSeq\_peptide;Acc:NP\_113725] | 5 |
| 1373043\_at |  | 683036  680945 | ENSRNOG00000001859 | ENSRNOT00000002540 | LOC683036 | stromal cell-derived factor 2-like 1 (predicted) (Sdf2l1\_predicted), mRNA [Source:RefSeq\_dna;Acc:NM\_001109433] | 5 |
| 1370361\_at | P97586 | 245918 | ENSRNOG00000007923 | ENSRNOT00000010586 | Cgref1 | Cell growth regulator with EF hand domain protein 1 (Cell growth regulatory gene 11 protein). [Source:UniProtKB/Swiss-Prot;Acc:P97586] | 5 |
| 1374264\_at |  | 362304 | ENSRNOG00000011519 | ENSRNOT00000015426 | LOC362304 | similar to ORC5-related protein (LOC362304), mRNA [Source:RefSeq\_dna;Acc:NM\_001014186] | 5 |
| 1373722\_at |  | 361308 | ENSRNOG00000024428 | ENSRNOT00000033784 | NP\_001101896.1 | kinesin family member 20A [Source:RefSeq\_peptide;Acc:NP\_001101896] | 5 |
| 1372891\_at |  | 304330 | ENSRNOG00000001290 | ENSRNOT00000001735 | NP\_001100596.1 | similar to 2810437L13Rik protein (predicted) (RGD1305631\_predicted), mRNA [Source:RefSeq\_dna;Acc:NM\_001107126] | 5 |
| 1393987\_s\_at |  | 59076 | ENSRNOG00000014615 | ENSRNOT00000020214 | Gprk6 | G protein-coupled receptor kinase 6 (EC 2.7.11.16) (G protein-coupled receptor kinase GRK6). [Source:UniProtKB/Swiss-Prot;Acc:P97711] | 5 |
| 1384340\_a\_at |  | 363518 | ENSRNOG00000029373 | ENSRNOT00000051254 | Ard1\_predicted |  | 5 |
| 1371015\_at | P18588 | 24575 | ENSRNOG00000001959 | ENSRNOT00000039876 | Mx1 | Interferon-induced GTP-binding protein Mx1. [Source:UniProtKB/Swiss-Prot;Acc:P18588] | 5 |
| 1378482\_at |  |  | ENSRNOG00000031036 | ENSRNOT00000042273 | NP\_001099138.1 | MyoD family inhibitor domain containing protein [Source:RefSeq\_peptide;Acc:NP\_001099138] | 5 |
| 1372363\_at |  | 287988  498109 | ENSRNOG00000032442 | ENSRNOT00000052258 | Polr2h\_predicted | RPB17 (Fragment). [Source:UniProtKB/TrEMBL;Acc:Q9Z0W2] | 5 |
| 1383940\_at | Q6AYL9 | 304951 | ENSRNOG00000002711 | ENSRNOT00000003650 | Cdca1 | Kinetochore protein Nuf2 (Cell division cycle-associated protein 1). [Source:UniProtKB/Swiss-Prot;Acc:Q6AYL9] | 5 |
| 1393085\_at | Q5I0J5 | 363219 | ENSRNOG00000018467 | ENSRNOT00000024959 | RGD1307700 | MIT domain-containing protein 1. [Source:UniProtKB/Swiss-Prot;Acc:Q5I0J5] | 5 |
| 1383829\_at |  | 303970 | ENSRNOG00000001971 | ENSRNOT00000002700  ENSRNOT00000002707  ENSRNOT00000048117 | Bbx\_predicted | bobby sox homolog [Source:RefSeq\_peptide;Acc:NP\_001073407] | 5 |
| 1397852\_at | Q5BJQ7 | 360874 | ENSRNOG00000003816 | ENSRNOT00000058689  ENSRNOT00000005090 | Tada1l | Transcriptional adapter 1-like protein. [Source:UniProtKB/Swiss-Prot;Acc:Q5BJQ7] | 5 |
| 1377146\_at | P01283 | 117064 | ENSRNOG00000018808 | ENSRNOT00000025477 | Vip | VIP peptides precursor [Contains: Intestinal peptide PHV-42; Intestinal peptide PHI-27 (Peptide histidine isoleucinamide 27); Vasoactive intestinal peptide (VIP) (Vasoactive intestinal polypeptide)]. [Source:UniProtKB/Swiss-Prot;Acc:P01283] | 5 |
| 1376199\_at | Q4KM34 | 364666 | ENSRNOG00000017991 | ENSRNOT00000024466 | Ccrk | Cell cycle-related kinase (EC 2.7.11.22). [Source:UniProtKB/Swiss-Prot;Acc:Q4KM34] | 5 |
| 1369638\_at | P70531 | 25435 | ENSRNOG00000016448 | ENSRNOT00000022726 | Eef2k | Elongation factor 2 kinase (EC 2.7.11.20) (eEF-2 kinase) (eEF-2K) (Calcium/calmodulin-dependent eukaryotic elongation factor 2 kinase). [Source:UniProtKB/Swiss-Prot;Acc:P70531] | 5 |
| 1372739\_at | Q5U1V9 | 362890 | ENSRNOG00000025592 | ENSRNOT00000031272 | Tspan31 | Tetraspanin-31 (Tspan-31) (Sarcoma-amplified sequence homolog). [Source:UniProtKB/Swiss-Prot;Acc:Q5U1V9] | 5 |
| 1370927\_at | P70560 | 25683 | ENSRNOG00000010510 | ENSRNOT00000051159 | Col12a1 | Collagen alpha-1(XII) chain (Fragment). [Source:UniProtKB/Swiss-Prot;Acc:P70560] | 5 |
| 1370154\_at | P00697 | 25211  688047 | ENSRNOG00000005825 | ENSRNOT00000007747 | Lyz | Lysozyme C type 1 precursor (EC 3.2.1.17) (1,4-beta-N-acetylmuramidase C). [Source:UniProtKB/Swiss-Prot;Acc:P00697] | 5 |
| 1372726\_at | P62804 | 679983  291152  684621  500351  679840  682518  684686  684887  64627  680097  680430  295277  502913  684745 | ENSRNOG00000030142 | ENSRNOT00000043151 | Hist1h4b | Histone H4 [Contains: Osteogenic growth peptide (OGP)]. [Source:UniProtKB/Swiss-Prot;Acc:P62804] | 5 |
| 1371042\_at |  |  | ENSRNOG00000007172 | ENSRNOT00000010311 | Map4k3 | Mitogen-activated protein kinase kinase kinase kinase 3 (EC 2.7.11.1) (MAPK/ERK kinase kinase kinase 3) (MEK kinase kinase 3) (MEKKK 3) (Germinal center kinase-related protein kinase) (GLK). [Source:UniProtKB/Swiss-Prot;Acc:Q924I2] | 5 |
| 1369737\_at |  | 25620 | ENSRNOG00000014900 | ENSRNOT00000020160  ENSRNOT00000051715  ENSRNOT00000020057 | Crem | cAMP-responsive element modulator. [Source:UniProtKB/Swiss-Prot;Acc:Q03061] | 5 |
| 1369956\_at |  | 116465 | ENSRNOG00000012074 | ENSRNOT00000016286 | Ifngr1 | interferon gamma receptor 1 [Source:RefSeq\_peptide;Acc:NP\_446235] | 5 |
| 1372930\_at | Q3KRF1 | 301570 | ENSRNOG00000033747 | ENSRNOT00000056995  ENSRNOT00000029654 | Sp110 | Sp110 nuclear body protein (Intracellular pathogen resistance protein 1). [Source:UniProtKB/Swiss-Prot;Acc:Q3KRF1] | 5 |
| 1373146\_at | Q8CGZ2 | 308023 | ENSRNOG00000015425 | ENSRNOT00000020839 | Ssx2ip | Afadin- and alpha-actinin-binding protein (ADIP) (Afadin DIL domain- interacting protein). [Source:UniProtKB/Swiss-Prot;Acc:Q8CGZ2] | 5 |
| 1389248\_at |  | 287835 | ENSRNOG00000006359 | ENSRNOT00000008525 | Galk1 | galactokinase 1 [Source:RefSeq\_peptide;Acc:NP\_001008283] | 5 |
| 1398907\_at |  | 288783 | ENSRNOG00000030120 | ENSRNOT00000044292 | NP\_001099410.1 | ORM1-like 2 [Source:RefSeq\_peptide;Acc:NP\_001099410] | 5 |
| 1374643\_at |  | 365507  310341 | ENSRNOG00000028335 | ENSRNOT00000038703 | RGD1564291\_predicted |  | 5 |
| 1396383\_at | Q5XI42 | 309147 | ENSRNOG00000017512 | ENSRNOT00000023789 | Aldh3b1 | Aldehyde dehydrogenase 3B1 (EC 1.2.1.5). [Source:UniProtKB/Swiss-Prot;Acc:Q5XI42] | 5 |
| 1388547\_at |  | 304407 | ENSRNOG00000001476 | ENSRNOT00000002003 | Cldn4 | claudin 4 [Source:RefSeq\_peptide;Acc:NP\_001012022] | 5 |
| 1398387\_at | Q6P7B4 | 310540 | ENSRNOG00000010183 | ENSRNOT00000013524 | MGC72614 | Uncharacterized protein C4orf18 homolog. [Source:UniProtKB/Swiss-Prot;Acc:Q6P7B4] | 5 |
| 1388512\_at |  | 363272 | ENSRNOG00000018610 | ENSRNOT00000025262 | NP\_001102276.1 | phosphodiesterase 6D, cGMP-specific, rod, delta [Source:RefSeq\_peptide;Acc:NP\_001102276] | 5 |
| 1367939\_at | P02696 | 25056 | ENSRNOG00000013794 | ENSRNOT00000018622 | Rbp1 | Retinol-binding protein I, cellular (Cellular retinol-binding protein) (CRBP). [Source:UniProtKB/Swiss-Prot;Acc:P02696] | 5 |
| 1388244\_s\_at | P38983 | 688911  679411  683125  29236 | ENSRNOG00000038617  ENSRNOG00000038519  ENSRNOG00000018645 | ENSRNOT00000058938  ENSRNOT00000058733  ENSRNOT00000025225 | LOC688911    Rpsa | 40S ribosomal protein SA (p40) (37/67 kDa laminin receptor). [Source:UniProtKB/Swiss-Prot;Acc:P38983] | 5 |
| 1380943\_at | Q5HZX7 | 313949 | ENSRNOG00000021475 | ENSRNOT00000030244 | RGD1311648 | Uncharacterized protein C2orf43 homolog. [Source:UniProtKB/Swiss-Prot;Acc:Q5HZX7] | 5 |
| 1367974\_at | P14669 | 25291 | ENSRNOG00000002045 | ENSRNOT00000002806 | Anxa3 | Annexin A3 (Annexin-3) (Annexin III) (Lipocortin III) (Placental anticoagulant protein III) (PAP-III) (35-alpha calcimedin). [Source:UniProtKB/Swiss-Prot;Acc:P14669] | 5 |
| 1387856\_at | P37397 | 54321 | ENSRNOG00000011559 | ENSRNOT00000015579 | Cnn3 | Calponin-3 (Calponin, acidic isoform) (Calponin, non-muscle isoform). [Source:UniProtKB/Swiss-Prot;Acc:P37397] | 5 |
| 1383279\_at | Q6P798 | 290363 | ENSRNOG00000015054 | ENSRNOT00000020836 | Rcbtb2 | RCC1 and BTB domain-containing protein 2 (Regulator of chromosome condensation and BTB domain-containing protein 2) (Chromosome condensation 1-like). [Source:UniProtKB/Swiss-Prot;Acc:Q6P798] | 5 |
| 1376457\_at |  | 171547 | ENSRNOG00000016752 | ENSRNOT00000023204 | Crispld2 | cysteine-rich secretory protein LCCL domain containing 2 [Source:RefSeq\_peptide;Acc:NP\_612527] | 5 |
| 1389263\_at | Q5U312 | 294804 | ENSRNOG00000028872 | ENSRNOT00000040348  ENSRNOT00000046215 | Rai14 | Ankycorbin (Ankyrin repeat and coiled-coil structure-containing protein) (Retinoic acid-induced protein 14). [Source:UniProtKB/Swiss-Prot;Acc:Q5U312] | 5 |
| 1369024\_at |  |  | ENSRNOG00000018462 | ENSRNOT00000025043 | Rabep2 | rabaptin, RAB GTPase binding effector protein 2 Gene [Source:MGI (curated);Acc:Rabep2-001] | 5 |
| 1388109\_at | Q9WVT0 | 245977 | ENSRNOG00000011154 | ENSRNOT00000015223  ENSRNOT00000046601 | Gpr116 | Probable G-protein coupled receptor 116 precursor (G-protein coupled hepta-helical receptor Ig-hepta). [Source:UniProtKB/Swiss-Prot;Acc:Q9WVT0] | 5 |
| 1396231\_at |  | 314596 | ENSRNOG00000006167 | ENSRNOT00000008136 | LOC687320 |  | 5 |
| 1374124\_at |  | 360498 | ENSRNOG00000019729 | ENSRNOT00000026789 | RGD1307381 | similar to RIKEN cDNA 2610003J06 (RGD1307381), mRNA [Source:RefSeq\_dna;Acc:NM\_001014116] | 5 |
| 1379737\_a\_at |  | 362251 | ENSRNOG00000019848 | ENSRNOT00000026964 | Rnpc2 | RNA-binding region (RNP1, RRM) containing 2 [Source:RefSeq\_peptide;Acc:NP\_001013225] | 5 |
| 1375219\_a\_at | P27952 | 83789  682718  688473  301438 | ENSRNOG00000022934  ENSRNOG00000025470  ENSRNOG00000030174  ENSRNOG00000001936  ENSRNOG00000005986  ENSRNOG00000014179  ENSRNOG00000033244  ENSRNOG00000027662  ENSRNOG00000032289  ENSRNOG00000031693  ENSRNOG00000037380  ENSRNOG00000010848  ENSRNOG00000007930 | ENSRNOT00000048967  ENSRNOT00000035386  ENSRNOT00000038746  ENSRNOT00000035389  ENSRNOT00000043480  ENSRNOT00000002656  ENSRNOT00000007876  ENSRNOT00000019508  ENSRNOT00000060074  ENSRNOT00000035575  ENSRNOT00000058491  ENSRNOT00000045740  ENSRNOT00000047772  ENSRNOT00000044776  ENSRNOT00000044157  ENSRNOT00000014416  ENSRNOT00000045325  ENSRNOT00000010437  ENSRNOT00000047257 | O55213\_RAT  LOC367712  Q6P9V0\_RAT  RGD1559516\_predicted    Rps2  LOC688473  RGD1562399\_predicted  O55212\_RAT | Ribosomal protein S2. [Source:UniProtKB/TrEMBL;Acc:O55213]    LOC100125366 protein. [Source:UniProtKB/TrEMBL;Acc:Q6P9V0]  40S ribosomal protein S2. [Source:UniProtKB/Swiss-Prot;Acc:P27952]  Ribosomal protein S2. [Source:UniProtKB/TrEMBL;Acc:O55212] | 5 |
| 1389010\_at | P30349 | 299732 | ENSRNOG00000004494 | ENSRNOT00000005930 | LKHA4\_RAT | Leukotriene A-4 hydrolase (EC 3.3.2.6) (LTA-4 hydrolase) (Leukotriene A(4) hydrolase). [Source:UniProtKB/Swiss-Prot;Acc:P30349] | 5 |
| 1374050\_at |  | 360521 | ENSRNOG00000003536 | ENSRNOT00000004740 | Rufy1 | Rufy1 protein (Fragment). [Source:UniProtKB/TrEMBL;Acc:Q4FZR3] | 5 |
| 1379631\_at | Q8JZQ0 | 78965 | ENSRNOG00000018659 | ENSRNOT00000025222  ENSRNOT00000055920 | Csf1 | Macrophage colony-stimulating factor 1 precursor (CSF-1) (MCSF) [Contains: Processed macrophage colony-stimulating factor 1]. [Source:UniProtKB/Swiss-Prot;Acc:Q8JZQ0] | 5 |
| 1376986\_at |  | 361035 | ENSRNOG00000013111 | ENSRNOT00000017720  ENSRNOT00000017610 | Mettl3 | methyltransferase-like 3 [Source:RefSeq\_peptide;Acc:NP\_001019965] | 5 |
| 1383922\_a\_at |  | 363518 | ENSRNOG00000029373 | ENSRNOT00000051254 | Ard1\_predicted |  | 5 |
| 1375034\_at |  | 361401 | ENSRNOG00000019859 | ENSRNOT00000026996 | Lypla3 | lysophospholipase 3 [Source:RefSeq\_peptide;Acc:NP\_001004277] | 5 |
| 1383684\_at |  | 304648 | ENSRNOG00000005115 | ENSRNOT00000006964 | NP\_001100630.1 | ASF1 anti-silencing function 1 homolog B [Source:RefSeq\_peptide;Acc:NP\_001100630] | 5 |
| 1389123\_at | Q68FP3 | 287910 | ENSRNOG00000030021 | ENSRNOT00000045867 | Ccl6 | C-C motif chemokine 6 precursor (Small-inducible cytokine A6). [Source:UniProtKB/Swiss-Prot;Acc:Q68FP3] | 5 |
| 1389179\_at |  | 291541 | ENSRNOG00000018505 | ENSRNOT00000024968 | Cidea\_predicted |  | 5 |
| 1388335\_at |  | 304983 | ENSRNOG00000008301 | ENSRNOT00000011208 | Tagln2 | transgelin 2 [Source:RefSeq\_peptide;Acc:NP\_001013145] | 5 |
| 1380334\_at | Q66H68 | 305340 | ENSRNOG00000002408 | ENSRNOT00000003327 | RGD1359713 | RNA-binding protein 47 (RNA-binding motif protein 47). [Source:UniProtKB/Swiss-Prot;Acc:Q66H68] | 5 |
| 1387808\_at | Q9R0S5 | 83509 | ENSRNOG00000010296 | ENSRNOT00000060195  ENSRNOT00000014391 | Slc7a7 | Y+L amino acid transporter 1 (y(+)L-type amino acid transporter 1) (y+LAT-1) (Y+LAT1) (Solute carrier family 7 member 7). [Source:UniProtKB/Swiss-Prot;Acc:Q9R0S5] | 5 |
| 1384068\_at |  | 306575  687334 | ENSRNOG00000024650 | ENSRNOT00000029266  ENSRNOT00000043405 | Ckap2\_predicted |  | 5 |
| 1376247\_at |  | 361042 | ENSRNOG00000018536 | ENSRNOT00000025260 | NP\_001101847.1 | phosphoenolpyruvate carboxykinase 2 (mitochondrial) [Source:RefSeq\_peptide;Acc:NP\_001101847] | 5 |
| 1371659\_at | P61589 | 117273  295342 | ENSRNOG00000012630 | ENSRNOT00000017254 | RHOA\_RAT | Transforming protein RhoA precursor. [Source:UniProtKB/Swiss-Prot;Acc:P61589] | 5 |
| 1384364\_at |  | 310363 | ENSRNOG00000015622 | ENSRNOT00000020925 | NP\_001101142.1 | F-box only protein 4 [Source:RefSeq\_peptide;Acc:NP\_001101142] | 5 |
| 1369303\_at | P01143 | 81648 | ENSRNOG00000012703 | ENSRNOT00000016953 | Crh | Corticoliberin precursor (Corticotropin-releasing factor) (CRF) (Corticotropin-releasing hormone). [Source:UniProtKB/Swiss-Prot;Acc:P01143] | 5 |
| 1376407\_a\_at |  | 362829  686978  690350 | ENSRNOG00000019552 | ENSRNOT00000026551 | LOC690350 | LSM7 homolog, U6 small nuclear RNA associated [Source:RefSeq\_peptide;Acc:NP\_001102202] | 5 |
| 1388744\_at |  | 288532 | ENSRNOG00000001349 | ENSRNOT00000001825 | Mcm7 | minichromosome maintenance complex component 7 [Source:RefSeq\_peptide;Acc:NP\_001004203] | 5 |
| 1376255\_at |  | 292763 | ENSRNOG00000020505 | ENSRNOT00000027837 | NP\_001099713.1 | mitogen activated protein kinase kinase kinase kinase 1 [Source:RefSeq\_peptide;Acc:NP\_001099713] | 5 |
| 1377143\_at | Q6V7K3 | 287642 | ENSRNOG00000004510 | ENSRNOT00000006179 | Slc35b1 | Solute carrier family 35 member B1 (UDP-galactose transporter-related protein 1) (UGTrel1). [Source:UniProtKB/Swiss-Prot;Acc:Q6V7K3] | 5 |
| 1374076\_at |  | 361409 | ENSRNOG00000018933 | ENSRNOT00000025604 | LOC690743 |  | 5 |
| 1370887\_at | Q99PD6 | 84574 | ENSRNOG00000019965 | ENSRNOT00000027139  ENSRNOT00000054980  ENSRNOT00000054979 | Tgfb1i1 | Transforming growth factor beta-1-induced transcript 1 protein (Hydrogen peroxide-inducible clone 5 protein) (Hic-5) (Androgen receptor-associated protein of 55 kDa). [Source:UniProtKB/Swiss-Prot;Acc:Q99PD6] | 5 |
| 1388339\_at | Q5U318 | 364052 | ENSRNOG00000006854 | ENSRNOT00000009049 | Pea15 | Astrocytic phosphoprotein PEA-15. [Source:UniProtKB/Swiss-Prot;Acc:Q5U318] | 5 |
| 1370850\_at | Q9JK00 | 245956 | ENSRNOG00000006937 | ENSRNOT00000009367 | Scn3b | Sodium channel subunit beta-3 precursor. [Source:UniProtKB/Swiss-Prot;Acc:Q9JK00] | 5 |
| 1371002\_at | P47816 | 58934 | ENSRNOG00000001490 | ENSRNOT00000002040 | Pdcd2 | Programmed cell death protein 2 (Zinc finger protein Rp-8) (Fragment). [Source:UniProtKB/Swiss-Prot;Acc:P47816] | 5 |
| 1375010\_at |  | 287435 | ENSRNOG00000037563 | ENSRNOT00000056918 | Cd68 | CD68 antigen [Source:RefSeq\_peptide;Acc:NP\_001026808] | 5 |
| 1373823\_at |  | 498709 | ENSRNOG00000014130 | ENSRNOT00000019234 | RGD1562047\_predicted |  | 5 |
| 1373085\_at |  | 304078 | ENSRNOG00000001701 | ENSRNOT00000002310 | NP\_001100580.1 | carbonyl reductase 3 [Source:RefSeq\_peptide;Acc:NP\_001100580] | 5 |
| 1367635\_at | P04785 | 25506 | ENSRNOG00000036689 | ENSRNOT00000054958 | P4hb | Protein disulfide-isomerase precursor (EC 5.3.4.1) (PDI) (Prolyl 4- hydroxylase subunit beta) (Cellular thyroid hormone-binding protein). [Source:UniProtKB/Swiss-Prot;Acc:P04785] | 5 |
| 1368075\_at | Q64194 | 25055 | ENSRNOG00000019077 | ENSRNOT00000025845 | Lip1 | Lysosomal acid lipase/cholesteryl ester hydrolase precursor (EC 3.1.1.13) (LAL) (Acid cholesteryl ester hydrolase) (Sterol esterase) (Lipase A) (Cholesteryl esterase). [Source:UniProtKB/Swiss-Prot;Acc:Q64194] | 5 |
| 1385426\_at |  | 295462 | ENSRNOG00000009433 | ENSRNOT00000012587 | RGD1305326\_predicted |  | 5 |
| 1386882\_at | Q9Z336 | 83462 | ENSRNOG00000018207 | ENSRNOT00000024656 | Tctex1 | Dynein light chain Tctex-type 1 (T-complex testis-specific protein 1 homolog). [Source:UniProtKB/Swiss-Prot;Acc:Q9Z336] | 5 |
| 1375967\_a\_at |  | 361242 | ENSRNOG00000018218 | ENSRNOT00000024587 | NP\_001101882.1 | dual specificity phosphatase 22 [Source:RefSeq\_peptide;Acc:NP\_001101882] | 5 |
| 1388836\_at | Q64617 | 81749 | ENSRNOG00000004873 | ENSRNOT00000006729 | Prkch | Protein kinase C eta type (EC 2.7.11.13) (nPKC-eta) (PKC-L). [Source:UniProtKB/Swiss-Prot;Acc:Q64617] | 5 |
| 1377268\_at | Q4V891 | 294667 | ENSRNOG00000018193 | ENSRNOT00000061507  ENSRNOT00000034184 | RGD1310597 | Uncharacterized protein C5orf37 homolog. [Source:UniProtKB/Swiss-Prot;Acc:Q4V891] | 5 |
| 1380191\_s\_at | Q920J4 | 140922 | ENSRNOG00000018818 | ENSRNOT00000025510 | Txnl1 | Thioredoxin-like protein 1 (Thioredoxin-related protein). [Source:UniProtKB/Swiss-Prot;Acc:Q920J4] | 5 |
| 1375346\_at |  | 500993 | ENSRNOG00000005918 | ENSRNOT00000007754 | NP\_001102763.1 |  | 5 |
| 1373245\_at |  |  | ENSRNOG00000016281 | ENSRNOT00000057386 | Col4a1 | Col4a1 protein (Fragment). [Source:UniProtKB/TrEMBL;Acc:Q5FWY9] | 5 |
| 1373992\_at |  | 498871  307414 | ENSRNOG00000019542 | ENSRNOT00000050063 | MGC108823 | similar to interferon-inducible GTPase (MGC108823), mRNA [Source:RefSeq\_dna;Acc:NM\_001012353] | 5 |
| 1380512\_at | Q5M872 | 291984 | ENSRNOG00000023303 | ENSRNOT00000034722 | Dpep2 | Dipeptidase 2 precursor (EC 3.4.13.19). [Source:UniProtKB/Swiss-Prot;Acc:Q5M872] | 5 |
| 1378418\_at |  | 364674 | ENSRNOG00000011947 | ENSRNOT00000015878 | RGD1311681 | similar to MGC37193 protein (RGD1311681), mRNA [Source:RefSeq\_dna;Acc:NM\_001025029] | 5 |
| 1372013\_at |  | 293618 | ENSRNOG00000004273 | ENSRNOT00000005645 | NP\_001099784.1 | interferon induced transmembrane protein 1 [Source:RefSeq\_peptide;Acc:NP\_001099784] | 5 |
| 1371249\_at |  | 289754 | ENSRNOG00000010298 | ENSRNOT00000014044 | Xbp1 | X-box binding protein 1 (Xbp1), mRNA [Source:RefSeq\_dna;Acc:NM\_001004210] | 5 |
| 1374032\_at |  |  | ENSRNOG00000009566 | ENSRNOT00000056234 | Q5BJL0\_RAT | Phf12 protein. [Source:UniProtKB/TrEMBL;Acc:Q5BJL0] | 5 |
| 1383853\_at | Q4V8A3 | 304775 | ENSRNOG00000004870 | ENSRNOT00000006502 | Dyrk3 | Dual specificity tyrosine-phosphorylation-regulated kinase 3 (EC 2.7.12.1). [Source:UniProtKB/Swiss-Prot;Acc:Q4V8A3] | 5 |
| 1373670\_at |  | 288774 | ENSRNOG00000031081 | ENSRNOT00000049536 | Stat2 | signal transducer and activator of transcription 2 [Source:RefSeq\_peptide;Acc:NP\_001011905] | 5 |
| 1371237\_a\_at | P02803 | 24567 | ENSRNOG00000025764  ENSRNOG00000038047 | ENSRNOT00000038212  ENSRNOT00000057898 | Q91ZP8\_RAT  Mt1a | Metallothionein 1 (Fragment). [Source:UniProtKB/TrEMBL;Acc:Q91ZP8]  Metallothionein-1 (MT-1) (Metallothionein-I) (MT-I). [Source:UniProtKB/Swiss-Prot;Acc:P02803] | 5 |
| 1376799\_a\_at |  |  | ENSRNOG00000020030 | ENSRNOT00000060097 | NP\_001099544.1 | cytokine receptor-like factor 1 [Source:RefSeq\_peptide;Acc:NP\_001099544] | 5 |
| 1370161\_at | Q6QD51 | 64387 | ENSRNOG00000002052 | ENSRNOT00000059735  ENSRNOT00000002828 | Ssg1 | Coiled-coil domain-containing protein 80 precursor (Down-regulated by oncogenes 1 protein) (Steroid sensitive gene 1 protein) (SSG-1). [Source:UniProtKB/Swiss-Prot;Acc:Q6QD51] | 5 |
| 1383440\_at |  | 690470  311218  687451 | ENSRNOG00000009199 | ENSRNOT00000012214 | LOC687451 |  | 5 |
| 1370412\_at | Q7TNB2 | 171409 | ENSRNOG00000028041 | ENSRNOT00000034957  ENSRNOT00000050462  ENSRNOT00000058844  ENSRNOT00000058843 | Tnnt1 | Troponin T, slow skeletal muscle (TnTs) (Slow skeletal muscle troponin T) (sTnT). [Source:UniProtKB/Swiss-Prot;Acc:Q7TNB2] | 5 |
| 1382275\_at |  | 361232 | ENSRNOG00000023799 | ENSRNOT00000036195 | MGC125015 | similar to PAK/PLC-interacting protein 1 (MGC125015), mRNA [Source:RefSeq\_dna;Acc:NM\_001037356] | 5 |
| 1394419\_at |  | 296060 | ENSRNOG00000008115 | ENSRNOT00000056543  ENSRNOT00000010994 | RGD1309107 |  | 5 |
| 1390797\_at |  |  | ENSRNOG00000005620 | ENSRNOT00000046135 | Lcp2 | lymphocyte cytosolic protein 2 [Source:RefSeq\_peptide;Acc:NP\_569105] | 5 |
| 1368629\_at | P10758 | 24714 | ENSRNOG00000006486 | ENSRNOT00000057869  ENSRNOT00000008583  ENSRNOT00000057867 | Reg1 | Lithostathine precursor (Pancreatic stone protein) (PSP) (Pancreatic thread protein) (PTP) (Islet of Langerhans regenerating protein) (REG) (Islet cells regeneration factor) (ICRF). [Source:UniProtKB/Swiss-Prot;Acc:P10758] | 5 |
| 1388378\_at |  | 293484 | ENSRNOG00000018761 | ENSRNOT00000025782  ENSRNOT00000025374 | Eif3s8 | Eif3s8 protein (Fragment). [Source:UniProtKB/TrEMBL;Acc:Q3MIB2] | 5 |
| 1378361\_at |  |  | ENSRNOG00000006689 | ENSRNOT00000008901 |  |  | 5 |
| 1372871\_at | Q6P7B2 | 316530 | ENSRNOG00000018325 | ENSRNOT00000024975 | RGD735175 | Uncharacterized protein C2orf24 homolog. [Source:UniProtKB/Swiss-Prot;Acc:Q6P7B2] | 5 |
| 1373339\_at |  |  | ENSRNOG00000029855 | ENSRNOT00000042786 | Letmd1 | LETM1 domain containing 1 Gene [Source:MGI Symbol;Acc:MGI:1915864] | 5 |
| 1368266\_at | P07824 | 29221 | ENSRNOG00000013304 | ENSRNOT00000017911 | Arg1 | Arginase-1 (EC 3.5.3.1) (Type I arginase) (Liver-type arginase). [Source:UniProtKB/Swiss-Prot;Acc:P07824] | 5 |
| 1374560\_at | Q3T1I4 | 291444 | ENSRNOG00000016433 | ENSRNOT00000022017 | MGC124825 | Protein PPRC1. [Source:UniProtKB/Swiss-Prot;Acc:Q3T1I4] | 5 |
| 1377058\_at |  | 500973 | ENSRNOG00000039390 | ENSRNOT00000040106 | RGD1564160\_predicted |  | 5 |
| 1384708\_at | Q63942 | 140665 | ENSRNOG00000011582 | ENSRNOT00000015609 | Rab3d | GTP-binding protein Rab-3D. [Source:UniProtKB/Swiss-Prot;Acc:Q63942] | 5 |
| 1368027\_at | P49430 | 24886 | ENSRNOG00000007918 | ENSRNOT00000010796 | Tbxas1 | Thromboxane-A synthase (EC 5.3.99.5) (TXA synthase) (TXS) (Cytochrome P450 5A1). [Source:UniProtKB/Swiss-Prot;Acc:P49430] | 5 |
| 1369539\_at | Q02734 | 64445 | ENSRNOG00000019843 | ENSRNOT00000056842  ENSRNOT00000026990 | St3gal3 | CMP-N-acetylneuraminate-beta-1,4-galactoside alpha-2,3- sialyltransferase (EC 2.4.99.6) (N-acetyllactosaminide alpha-2,3- sialyltransferase) (Gal beta-1,3(4) GlcNAc alpha-2,3 sialyltransferase) (ST3N) (ST3GalIII) (Sialyltransferase 6) [Contains: CMP-N-ace [Source:UniProtKB/Swiss-Prot;Acc:Q02734] | 5 |
| 1369879\_a\_at | P55062 | 24822 | ENSRNOG00000016911 | ENSRNOT00000045927  ENSRNOT00000022838 | Tegt | Bax inhibitor 1 (BI-1) (Testis-enhanced gene transcript protein). [Source:UniProtKB/Swiss-Prot;Acc:P55062] | 5 |
| 1375849\_at |  | 308739 | ENSRNOG00000012874 | ENSRNOT00000017473 | NP\_001100994.1 | RGM domain family, member A [Source:RefSeq\_peptide;Acc:NP\_001100994] | 5 |
| 1379406\_at |  | 290235 | ENSRNOG00000020321 | ENSRNOT00000027604 | NP\_001099510.1 | similar to chromosome 14 open reading frame 21 (predicted) (RGD1308396\_predicted), mRNA [Source:RefSeq\_dna;Acc:NM\_001106040] | 5 |
| 1374561\_at |  |  | ENSRNOG00000013832 | ENSRNOT00000018616 | RGD1309634\_predicted |  | 5 |
| 1388392\_at |  |  | ENSRNOG00000019357 | ENSRNOT00000026310 | Tax1bp3 | Tax1-binding protein 3. [Source:UniProtKB/Swiss-Prot;Acc:Q4QQV1] | 5 |
| 1386913\_at |  |  | ENSRNOG00000014961 | ENSRNOT00000020316 | Pdpn | Podoplanin precursor (Glycoprotein 38) (Gp38) (OTS-8) (E11 antigen epitope) (T1A) (T1-alpha) (Type I cell 40 kDa protein) (RTI140). [Source:UniProtKB/Swiss-Prot;Acc:Q64294] | 5 |
| 1369294\_at | Q63072 | 81506 | ENSRNOG00000003064 | ENSRNOT00000004094 | Bst1 | ADP-ribosyl cyclase 2 precursor (EC 3.2.2.5) (Cyclic ADP-ribose hydrolase 2) (cADPr hydrolase 2) (Bone marrow stromal antigen 1) (BST- 1) (CD157 antigen). [Source:UniProtKB/Swiss-Prot;Acc:Q63072] | 5 |
| 1371909\_at | Q5EB59 | 309565 | ENSRNOG00000013422 | ENSRNOT00000032424 | Crsp3 | Mediator of RNA polymerase II transcription subunit 23 (Mediator complex subunit 23) (CRSP complex subunit 3) (Cofactor required for Sp1 transcriptional activation subunit 3). [Source:UniProtKB/Swiss-Prot;Acc:Q5EB59] | 5 |
| 1372977\_at |  | 503027 | ENSRNOG00000004923 | ENSRNOT00000006705 | RGD1562028\_predicted |  | 5 |
| 1367664\_at | Q8R560 | 27064 | ENSRNOG00000018598 | ENSRNOT00000025258 | Ankrd1 | Ankyrin repeat domain-containing protein 1 (Cardiac ankyrin repeat protein) (Cardiac adriamycin-responsive protein). [Source:UniProtKB/Swiss-Prot;Acc:Q8R560] | 5 |
| 1373847\_at |  | 295061 | ENSRNOG00000015812 | ENSRNOT00000021535 | NP\_001099904.1 | transmembrane 4 superfamily member 1 [Source:RefSeq\_peptide;Acc:NP\_001099904] | 5 |
| 1386925\_at | O88656 | 54227 | ENSRNOG00000000991 | ENSRNOT00000001315 | Arpc1b | Actin-related protein 2/3 complex subunit 1B (Arp2/3 complex 41 kDa subunit) (p41-ARC). [Source:UniProtKB/Swiss-Prot;Acc:O88656] | 5 |
| 1374362\_at |  | 307834 | ENSRNOG00000000262 | ENSRNOT00000000279 | RGD1306674\_predicted |  | 5 |
| 1368674\_at | P09811 | 64035 | ENSRNOG00000006388 | ENSRNOT00000009183 | Pygl | Glycogen phosphorylase, liver form (EC 2.4.1.1). [Source:UniProtKB/Swiss-Prot;Acc:P09811] | 5 |
| 1392770\_at | P97603 |  | ENSRNOG00000006490 | ENSRNOT00000009312  ENSRNOT00000009496 | NEO1\_RAT | Neogenin precursor (Fragment). [Source:UniProtKB/Swiss-Prot;Acc:P97603] | 5 |
| 1385404\_at | Q499M5 | 307102 | ENSRNOG00000028168 | ENSRNOT00000025011 | Ankrd16 | Ankyrin repeat domain-containing protein 16. [Source:UniProtKB/Swiss-Prot;Acc:Q499M5] | 5 |
| 1368143\_at |  | 155423 | ENSRNOG00000007136 | ENSRNOT00000010011  ENSRNOT00000061686 | Anxa7 | annexin A7 [Source:RefSeq\_peptide;Acc:NP\_569100] | 5 |
| 1382181\_at |  |  | ENSRNOG00000028801 | ENSRNOT00000039953 | Pion | pigeon homolog (Drosophila) Gene [Source:MGI Symbol;Acc:MGI:2442259] | 5 |
| 1376423\_at |  |  | ENSRNOG00000018986 | ENSRNOT00000049576 | Fbxl19 | F-box and leucine-rich repeat protein 19 Gene [Source:MGI Symbol;Acc:MGI:3039600] | 5 |
| 1390506\_at |  | 497991 | ENSRNOG00000005606 | ENSRNOT00000007842 | RGD1559552\_predicted |  | 5 |
| 1390743\_at | P62950 | 171113 | ENSRNOG00000024437 | ENSRNOT00000033525 | Blcap | Bladder cancer-associated protein (Bladder cancer 10 kDa protein) (Bc10). [Source:UniProtKB/Swiss-Prot;Acc:P62950] | 5 |
| 1382732\_at |  | 293186 | ENSRNOG00000026902 | ENSRNOT00000030474 | NP\_001099756.1 | extracellular link domain-containing 1 [Source:RefSeq\_peptide;Acc:NP\_001099756] | 5 |
| 1371478\_at |  | 296315 | ENSRNOG00000020317 | ENSRNOT00000027539 | RGD1307752 | RAB5-interacting protein [Source:RefSeq\_peptide;Acc:NP\_001013944] | 5 |
| 1392544\_at |  | 301513 | ENSRNOG00000016034 | ENSRNOT00000021548 | Rqcd1 | rcd1 (required for cell differentiation) homolog 1 [Source:RefSeq\_peptide;Acc:NP\_001009357] | 5 |
| 1387917\_at | Q5PQX1 | 246314 | ENSRNOG00000003946 | ENSRNOT00000005280  ENSRNOT00000005303 | Tor1aip1 | Torsin-1A-interacting protein 1 (Lamina-associated polypeptide 1B) (Lamina-associated polypeptide 1C). [Source:UniProtKB/Swiss-Prot;Acc:Q5PQX1] | 5 |
| 1392996\_at |  |  | ENSRNOG00000019161 | ENSRNOT00000014790 | CPEB1\_RAT | Cytoplasmic polyadenylation element-binding protein 1 (CPE-binding protein 1) (CPE-BP1) (CPEB-1) (CPEB). [Source:UniProtKB/Swiss-Prot;Acc:P0C279] | 5 |
| 1380621\_at |  | 361597 | ENSRNOG00000011683 | ENSRNOT00000016032 | NP\_001101958.1 | similar to tyrosine kinase Fps/Fes (predicted) (RGD1564385\_predicted), mRNA [Source:RefSeq\_dna;Acc:NM\_001108488] | 5 |
| 1387628\_at | Q9JKT9 | 78981 | ENSRNOG00000005646 | ENSRNOT00000007453 | Tas2r10 | Taste receptor type 2 member 107 (T2R107) (Taste receptor type 2 member 4) (T2R4). [Source:UniProtKB/Swiss-Prot;Acc:Q9JKT9] | 5 |
| 1369191\_at | P20607 | 24498 | ENSRNOG00000010278 | ENSRNOT00000013732 | Il6 | Interleukin-6 precursor (IL-6). [Source:UniProtKB/Swiss-Prot;Acc:P20607] | 5 |
| 1376709\_at | Q5FVQ0 | 295455 | ENSRNOG00000012508 | ENSRNOT00000033413 | Slc39a8 | Zinc transporter ZIP8 (Zrt- and Irt-like protein 8) (ZIP-8) (Solute carrier family 39 member 8). [Source:UniProtKB/Swiss-Prot;Acc:Q5FVQ0] | 5 |
| 1379665\_at |  | 294711 | ENSRNOG00000012505 | ENSRNOT00000016863 | NP\_001099876.1 | peptidylprolyl isomerase domain and WD repeat containing 1 [Source:RefSeq\_peptide;Acc:NP\_001099876] | 5 |
| 1381630\_at |  | 362175 | ENSRNOG00000010963 | ENSRNOT00000014767 | NP\_001102055.1 | similar to RIKEN cDNA A930018P22 (predicted) (RGD1563222\_predicted), mRNA [Source:RefSeq\_dna;Acc:NM\_001108585] | 5 |
| 1368915\_at | O88867 | 59113 | ENSRNOG00000003709 | ENSRNOT00000005005 | Kmo | Kynurenine 3-monooxygenase (EC 1.14.13.9) (Kynurenine 3-hydroxylase). [Source:UniProtKB/Swiss-Prot;Acc:O88867] | 5 |
| 1379766\_at |  |  | ENSRNOG00000005797 | ENSRNOT00000056738 | Sla | SRC-like-adapter (Src-like-adapter protein 1). [Source:UniProtKB/Swiss-Prot;Acc:P59622] | 5 |
| 1367754\_s\_at | P20673 | 59085 | ENSRNOG00000000903 | ENSRNOT00000001211 | Asl | Argininosuccinate lyase (EC 4.3.2.1) (Arginosuccinase) (ASAL). [Source:UniProtKB/Swiss-Prot;Acc:P20673] | 5 |
| 1375865\_at |  | 316233 | ENSRNOG00000018980 | ENSRNOT00000025660 | NP\_001101673.1 | tight junction associated protein 1 [Source:RefSeq\_peptide;Acc:NP\_001101673] | 5 |
| 1376605\_at |  | 363103 | ENSRNOG00000009330 | ENSRNOT00000012474 | Slc17a5 | solute carrier family 17 (anion/sugar transporter), member 5 [Source:RefSeq\_peptide;Acc:NP\_001009713] | 5 |
| 1367661\_at | P05964 | 85247 | ENSRNOG00000011647 | ENSRNOT00000015612 | S100a6 | Protein S100-A6 (S100 calcium-binding protein A6) (Calcyclin) (Prolactin receptor-associated protein). [Source:UniProtKB/Swiss-Prot;Acc:P05964] | 5 |
| 1392064\_at |  | 296500 | ENSRNOG00000001520 | ENSRNOT00000002078 | Dlx1 |  | 5 |
| 1395605\_at |  | 287942 | ENSRNOG00000001868 | ENSRNOT00000002552 | Crkl | v-crk sarcoma virus CT10 oncogene homolog (avian)-like [Source:RefSeq\_peptide;Acc:NP\_001008285] | 5 |
| 1396123\_at |  | 498095 | ENSRNOG00000001758 | ENSRNOT00000002397 | NP\_001102524.1 |  | 5 |
| 1380623\_at |  | 363521 | ENSRNOG00000037243 | ENSRNOT00000056290 | Taz | tafazzin [Source:RefSeq\_peptide;Acc:NP\_001020919] | 5 |
| 1389680\_at |  | 309918 | ENSRNOG00000027089 | ENSRNOT00000032974 | Ell2 | Ell2 protein (Fragment). [Source:UniProtKB/TrEMBL;Acc:Q2NL49] | 5 |
| 1367786\_at | P28064 | 24968 | ENSRNOG00000000456 | ENSRNOT00000000528 | Psmb8 | Proteasome subunit beta type-8 precursor (EC 3.4.25.1) (Proteasome subunit beta-5i) (Proteasome component C13) (Macropain subunit C13) (Multicatalytic endopeptidase complex subunit C13). [Source:UniProtKB/Swiss-Prot;Acc:P28064] | 5 |
| 1376663\_at |  | 304653 | ENSRNOG00000006551 | ENSRNOT00000008786  ENSRNOT00000060221 | NP\_001100632.1 |  | 5 |
| 1390941\_at |  | 308968 | ENSRNOG00000012806 | ENSRNOT00000049430  ENSRNOT00000035770 | Rbbp6 | Rbbp6 protein (Fragment). [Source:UniProtKB/TrEMBL;Acc:Q5EB85] | 5 |
| 1367650\_at | Q9EQT5 | 94174 | ENSRNOG00000013179 | ENSRNOT00000018464 | Lcn7 | Tubulointerstitial nephritis antigen-like precursor (Glucocorticoid- inducible protein 5). [Source:UniProtKB/Swiss-Prot;Acc:Q9EQT5] | 5 |
| 1379440\_at | Q99PW7 | 114031 | ENSRNOG00000009311 | ENSRNOT00000012578 | Fstl3 | Follistatin-related protein 3 precursor (Follistatin-like 3) (Follistatin-related gene protein). [Source:UniProtKB/Swiss-Prot;Acc:Q99PW7] | 5 |
| 1387453\_at | Q9WTY8 | 80338 | ENSRNOG00000011319 | ENSRNOT00000015171 | Zbtb10 | Zinc finger and BTB domain-containing protein 10 (Zinc finger protein RIN ZF). [Source:UniProtKB/Swiss-Prot;Acc:Q9WTY8] | 5 |
| 1369943\_at |  | 56083 | ENSRNOG00000012956 | ENSRNOT00000018328 | Tgm2 | transglutaminase 2, C polypeptide [Source:RefSeq\_peptide;Acc:NP\_062259] | 5 |
| 1384048\_at |  |  | ENSRNOG00000015560 | ENSRNOT00000020882  ENSRNOT00000043788  ENSRNOT00000030463 | 2210018M11Rik | RIKEN cDNA 2210018M11 gene Gene [Source:MGI Symbol;Acc:MGI:1924203] | 5 |
| 1367794\_at | P06238 | 24153 | ENSRNOG00000028896 | ENSRNOT00000019346  ENSRNOT00000044374 | A2m | Alpha-2-macroglobulin precursor (Alpha-2-M). [Source:UniProtKB/Swiss-Prot;Acc:P06238] | 5 |
| 1371602\_at |  | 312728 | ENSRNOG00000005442 | ENSRNOT00000007328 | NP\_001101360.1 | tetraspanin 9 [Source:RefSeq\_peptide;Acc:NP\_001101360] | 5 |
| 1382348\_at | Q63707 | 65156 | ENSRNOG00000015063 | ENSRNOT00000020302 | Dhodh | Dihydroorotate dehydrogenase, mitochondrial precursor (EC 1.3.3.1) (Dihydroorotate oxidase) (DHOdehase). [Source:UniProtKB/Swiss-Prot;Acc:Q63707] | 5 |
| 1371250\_at | P06765 | 360918 | ENSRNOG00000028015 | ENSRNOT00000033406 | Cxcl4 | Platelet factor 4 precursor (PF-4) (C-X-C motif chemokine 4). [Source:UniProtKB/Swiss-Prot;Acc:P06765] | 5 |
| 1398876\_at | Q6MG08 | 85493 | ENSRNOG00000000799 | ENSRNOT00000001049 | Abcf1 | ATP-binding cassette sub-family F member 1 (ATP-binding cassette 50). [Source:UniProtKB/Swiss-Prot;Acc:Q6MG08] | 5 |
| 1393641\_at |  | 499356 | ENSRNOG00000013967 | ENSRNOT00000019014 | Blnk | B-cell linker (Blnk), mRNA [Source:RefSeq\_dna;Acc:NM\_001025767] | 5 |
| 1371134\_at |  |  | ENSRNOG00000013305 | ENSRNOT00000018175 | Atp2c1 | Calcium-transporting ATPase type 2C member 1 (EC 3.6.3.8) (ATPase 2C1) (ATP-dependent Ca(2+) pump PMR1). [Source:UniProtKB/Swiss-Prot;Acc:Q64566] | 5 |
| 1387233\_at | Q62904 | 29540 | ENSRNOG00000002826 | ENSRNOT00000003812 | Hsd17b7 | 3-keto-steroid reductase (EC 1.1.1.270) (Estradiol 17-beta- dehydrogenase 7) (EC 1.1.1.62) (17-beta-HSD 7) (17-beta-hydroxysteroid dehydrogenase 7) (PRL receptor-associated protein) (PRAP). [Source:UniProtKB/Swiss-Prot;Acc:Q62904] | 5 |
| 1376062\_at | P26260 | 25216 | ENSRNOG00000006396 | ENSRNOT00000008582 | Sdc1 | Syndecan-1 precursor (SYND1) (CD138 antigen). [Source:UniProtKB/Swiss-Prot;Acc:P26260] | 5 |
| 1374991\_at |  | 287938 | ENSRNOG00000001861 | ENSRNOT00000002542 | LOC287938 |  | 5 |
| 1379741\_at |  |  | ENSRNOG00000013428 | ENSRNOT00000018180 | Atp6v0a4 | ATPase, H+ transporting, lysosomal V0 subunit A4 Gene [Source:MGI (curated);Acc:Atp6v0a4-001] | 5 |
| 1368921\_a\_at | P26051 | 25406 | ENSRNOG00000006094 | ENSRNOT00000009000  ENSRNOT00000008760  ENSRNOT00000009073  ENSRNOT00000049698 | Cd44 | CD44 antigen precursor (Phagocytic glycoprotein 1) (PGP-1) (HUTCH-I) (Extracellular matrix receptor III) (ECMR-III) (GP90 lymphocyte homing/adhesion receptor) (Hermes antigen) (Hyaluronate receptor). [Source:UniProtKB/Swiss-Prot;Acc:P26051] | 5 |
| 1385086\_at |  | 296137 | ENSRNOG00000032778 | ENSRNOT00000051783  ENSRNOT00000047475 | NP\_001099977.1 | budding uninhibited by benzimidazoles 1 homolog [Source:RefSeq\_peptide;Acc:NP\_001099977] | 5 |
| 1371487\_at |  | 298544 | ENSRNOG00000015967 | ENSRNOT00000021734 | NP\_001100158.1 | SH3 domain binding glutamic acid-rich protein-like 3 [Source:RefSeq\_peptide;Acc:NP\_001100158] | 6 |
| 1393370\_at |  | 310698 | ENSRNOG00000016550 | ENSRNOT00000057062 | Dclk2\_predicted | similar to doublecortin-like kinase 2 [Source:RefSeq\_peptide;Acc:NP\_001009691] | 6 |
| 1387995\_a\_at | P26376 | 361673 | ENSRNOG00000015078 | ENSRNOT00000020265 | Ifitm3 | Interferon-inducible protein. [Source:UniProtKB/Swiss-Prot;Acc:P26376] | 6 |
| 1393042\_at |  |  | ENSRNOG00000025496 | ENSRNOT00000050277 | Usp7 | Ubiquitin carboxyl-terminal hydrolase 7 (EC 3.1.2.15) (Ubiquitin thioesterase 7) (Ubiquitin-specific-processing protease 7) (Deubiquitinating enzyme 7) (Herpesvirus-associated ubiquitin-specific protease) (rHAUSP). [Source:UniProtKB/Swiss-Prot;Acc:Q4VSI4] | 6 |
| 1368353\_at | P47819 | 24387 | ENSRNOG00000002919 | ENSRNOT00000034401 | Gfap | Glial fibrillary acidic protein (GFAP). [Source:UniProtKB/Swiss-Prot;Acc:P47819] | 6 |
| 1373559\_at | P97738 | 288475 | ENSRNOG00000001006 | ENSRNOT00000001331 | Nptx2 | Neuronal pentraxin-2 precursor (NP2) (Neuronal pentraxin II) (NP-II) (Neuronal activity-regulated pentraxin). [Source:UniProtKB/Swiss-Prot;Acc:P97738] | 6 |
| 1377112\_at |  | 362638 | ENSRNOG00000015677 | ENSRNOT00000021036 | NP\_001102158.1 | cytidine deaminase [Source:RefSeq\_peptide;Acc:NP\_001102158] | 6 |
| 1367752\_at | Q63767 | 25414 | ENSRNOG00000019253 | ENSRNOT00000041815 | Bcar1 | Breast cancer anti-estrogen resistance protein 1 (CRK-associated substrate) (p130cas). [Source:UniProtKB/Swiss-Prot;Acc:Q63767] | 6 |
| 1368657\_at | P03957 | 171045 | ENSRNOG00000032626 | ENSRNOT00000012310 | Mmp3 | Stromelysin-1 precursor (EC 3.4.24.17) (Matrix metalloproteinase-3) (MMP-3) (Transin-1) (SL-1) (PTR1 protein). [Source:UniProtKB/Swiss-Prot;Acc:P03957] | 6 |
| 1387008\_at | Q9JHY2 | 65042 | ENSRNOG00000015442 | ENSRNOT00000021171 | Sfxn3 | Sideroflexin-3. [Source:UniProtKB/Swiss-Prot;Acc:Q9JHY2] | 6 |
| 1398829\_at | Q62658 | 25639 | ENSRNOG00000008822 | ENSRNOT00000012608 | Fkbp1a | FK506-binding protein 1A (EC 5.2.1.8) (Peptidyl-prolyl cis-trans isomerase) (PPIase) (Rotamase) (12 kDa FKBP) (FKBP-12) (Immunophilin FKBP12). [Source:UniProtKB/Swiss-Prot;Acc:Q62658] | 6 |
| 1372459\_at |  | 361517 | ENSRNOG00000016367 | ENSRNOT00000022214 | NP\_001101945.1 | vasodilator-stimulated phosphoprotein [Source:RefSeq\_peptide;Acc:NP\_001101945] | 6 |
| 1388784\_at |  | 307403 | ENSRNOG00000018414 | ENSRNOT00000024883 | Csf1r | colony stimulating factor 1 receptor [Source:RefSeq\_peptide;Acc:NP\_001025072] | 6 |
| 1368735\_a\_at | Q9WUD2 | 29465 | ENSRNOG00000003104 | ENSRNOT00000004248 | Trpv2 | Transient receptor potential cation channel subfamily V member 2 (TrpV2) (osm-9-like TRP channel 2) (OTRPC2) (Vanilloid receptor-like protein 1) (VRL-1) (Stretch-activated channel 2B). [Source:UniProtKB/Swiss-Prot;Acc:Q9WUD2] | 6 |
| 1371592\_at | P32577 | 315707 | ENSRNOG00000019374 | ENSRNOT00000026358 | Csk | Tyrosine-protein kinase CSK (EC 2.7.10.2) (C-SRC kinase). [Source:UniProtKB/Swiss-Prot;Acc:P32577] | 6 |
| 1372073\_at |  |  | ENSRNOG00000022173 | ENSRNOT00000027943 | Q5EB93\_RAT | GATA zinc finger domain containing 2A. [Source:UniProtKB/TrEMBL;Acc:Q5EB93] | 6 |
| 1374876\_at |  | 361160 | ENSRNOG00000012601 | ENSRNOT00000016823 | Leprotl1 | leptin receptor overlapping transcript-like 1 [Source:RefSeq\_peptide;Acc:NP\_001013206] | 6 |
| 1373737\_at |  | 367328 | ENSRNOG00000012504 | ENSRNOT00000016588 | ORF19 |  | 6 |
| 1368148\_at | P07174 | 24596 | ENSRNOG00000005392 | ENSRNOT00000007268 | Ngfr | Tumor necrosis factor receptor superfamily member 16 precursor (Low- affinity nerve growth factor receptor) (NGF receptor) (Gp80-LNGFR) (p75 ICD) (Low affinity neurotrophin receptor p75NTR). [Source:UniProtKB/Swiss-Prot;Acc:P07174] | 6 |
| 1379805\_at |  |  | ENSRNOG00000008713 | ENSRNOT00000011572 | Slc41a2 | solute carrier family 41, member 2 Gene [Source:MGI (curated);Acc:Slc41a2-001] | 6 |
| 1371049\_at | Q62951 | 25417 | ENSRNOG00000027582 | ENSRNOT00000029587 | Dpysl4 | Dihydropyrimidinase-related protein 4 (DRP-4) (Collapsin response mediator protein 3) (CRMP-3) (UNC33-like phosphoprotein 4) (ULIP4 protein) (Fragment). [Source:UniProtKB/Swiss-Prot;Acc:Q62951] | 6 |
| 1369950\_at | P35426 | 94201 | ENSRNOG00000025602 | ENSRNOT00000031796 | Cdk4 | Cell division protein kinase 4 (EC 2.7.11.22) (Cyclin-dependent kinase 4) (PSK-J3). [Source:UniProtKB/Swiss-Prot;Acc:P35426] | 6 |
| 1367465\_at | P61805 | 192275 | ENSRNOG00000009090 | ENSRNOT00000012233 | Dad1 | Dolichyl-diphosphooligosaccharide--protein glycosyltransferase subunit DAD1 (EC 2.4.1.119) (Oligosaccharyl transferase subunit DAD1) (Defender against cell death 1) (DAD-1). [Source:UniProtKB/Swiss-Prot;Acc:P61805] | 6 |
| 1382468\_at |  | 361201 | ENSRNOG00000019232 | ENSRNOT00000025997  ENSRNOT00000061249 | RGD1311345 | RGD1311345 protein. [Source:UniProtKB/TrEMBL;Acc:Q4KM26] | 6 |
| 1371560\_at |  | 292892 | ENSRNOG00000020443 | ENSRNOT00000027786 | Stk22s1 | Testis-specific serine kinase substrate (Testis-specific kinase substrate) (STK22 substrate 1). [Source:UniProtKB/Swiss-Prot;Acc:P60531] | 6 |
| 1391252\_at |  | 362814 | ENSRNOG00000023456 | ENSRNOT00000029778 | Rnf41 | ring finger protein 41 [Source:RefSeq\_peptide;Acc:NP\_001012195] | 6 |
| 1387007\_at | Q62997 | 25454 | ENSRNOG00000017438 | ENSRNOT00000023667 | Gfra1 | GDNF family receptor alpha-1 precursor (GFR-alpha-1) (GDNF receptor alpha) (GDNFR-alpha) (TGF-beta-related neurotrophic factor receptor 1) (RET ligand 1). [Source:UniProtKB/Swiss-Prot;Acc:Q62997] | 6 |
| 1382322\_a\_at |  |  | ENSRNOG00000020432 | ENSRNOT00000027710 | Cic\_predicted | capicua homolog [Source:RefSeq\_peptide;Acc:NP\_001100960] | 6 |
| 1383152\_at | A0JPQ9 | 293628 | ENSRNOG00000019351 | ENSRNOT00000026215  ENSRNOT00000026390  ENSRNOT00000044390 | RGD1308955 | Chitinase domain-containing protein 1 precursor. [Source:UniProtKB/Swiss-Prot;Acc:A0JPQ9] | 6 |
| 1372877\_at | Q5U367 | 288583 | ENSRNOG00000001417 | ENSRNOT00000001921  ENSRNOT00000035117 | Plod3 | Procollagen-lysine,2-oxoglutarate 5-dioxygenase 3 precursor (EC 1.14.11.4) (Lysyl hydroxylase 3) (LH3). [Source:UniProtKB/Swiss-Prot;Acc:Q5U367] | 6 |
| 1374564\_at |  | 304591 | ENSRNOG00000001432 | ENSRNOT00000001950 | LOC688965 | deltex 2 homolog [Source:RefSeq\_peptide;Acc:NP\_001100627] | 6 |
| 1371244\_at | P36972 | 292072 | ENSRNOG00000014405 | ENSRNOT00000019362 | Aprt\_predicted | Adenine phosphoribosyltransferase (EC 2.4.2.7) (APRT). [Source:UniProtKB/Swiss-Prot;Acc:P36972] | 6 |
| 1368488\_at | Q6IMZ0 | 114519 | ENSRNOG00000011668 | ENSRNOT00000015525 | Nfil3 | Nuclear factor interleukin-3-regulated protein (E4 promoter binding- protein 4). [Source:UniProtKB/Swiss-Prot;Acc:Q6IMZ0] | 6 |
| 1398438\_at |  |  | ENSRNOG00000023403 | ENSRNOT00000060355 | Gtpbp3 | tRNA modification GTPase GTPBP3, mitochondrial precursor (GTP-binding protein 3). [Source:UniProtKB/Swiss-Prot;Acc:Q5PQQ1] | 6 |
| 1371800\_at |  | 313038 | ENSRNOG00000006004 | ENSRNOT00000008550 | Phc2 |  | 6 |
| 1382325\_at |  | 366959 | ENSRNOG00000010596 | ENSRNOT00000014173 | Gcat | glycine C-acetyltransferase (2-amino-3-ketobutyrate-coenzyme A ligase) [Source:RefSeq\_peptide;Acc:NP\_001019448] | 6 |
| 1371382\_at |  | 293860 | ENSRNOG00000037239 | ENSRNOT00000008910 | Flna\_predicted |  | 6 |
| 1367577\_at | P42930 | 24471 | ENSRNOG00000023546 | ENSRNOT00000031555  ENSRNOT00000050688 | Hspb1 | Heat shock protein beta-1 (HspB1) (Heat shock 27 kDa protein) (HSP 27). [Source:UniProtKB/Swiss-Prot;Acc:P42930] | 6 |
| 1377809\_at |  | 681884  688786 | ENSRNOG00000040092 | ENSRNOT00000061666 | LOC688786 |  | 6 |
| 1371737\_at |  |  | ENSRNOG00000018622 | ENSRNOT00000025169 | Trim27 | tripartite motif-containing 27 Gene [Source:MGI Symbol;Acc:MGI:97904] | 6 |
| 1370162\_at | Q8VI02 | 140943 | ENSRNOG00000013733 | ENSRNOT00000019603 | Ppp4r1 | Serine/threonine-protein phosphatase 4 regulatory subunit 1. [Source:UniProtKB/Swiss-Prot;Acc:Q8VI02] | 6 |
| 1373970\_at | Q66H70 | 361749 | ENSRNOG00000016456 | ENSRNOT00000022056 | RGD1311155 | Interleukin-33 precursor (IL-33). [Source:UniProtKB/Swiss-Prot;Acc:Q66H70] | 6 |
| 1387972\_at | Q9JIK1 | 171554 | ENSRNOG00000017762 | ENSRNOT00000024031  ENSRNOT00000054872  ENSRNOT00000044238 | Mucdhl | Mucin and cadherin-like protein precursor (Mu-protocadherin) (GP100). [Source:UniProtKB/Swiss-Prot;Acc:Q9JIK1] | 6 |
| 1373204\_at | Q4G068 | 297077 | ENSRNOG00000023708 | ENSRNOT00000049891  ENSRNOT00000032504 | RGD1310725 | Transmembrane protein 176A. [Source:UniProtKB/Swiss-Prot;Acc:Q4G068] | 6 |
| 1390032\_at |  | 288771 | ENSRNOG00000003076 | ENSRNOT00000048848 | Rbms2 | RNA binding motif, single stranded interacting protein 2 [Source:RefSeq\_peptide;Acc:NP\_001020574] | 6 |
| 1371500\_at |  |  | ENSRNOG00000020871 | ENSRNOT00000028319  ENSRNOT00000028322 | Ltbp4 | latent transforming growth factor beta binding protein 4 Gene [Source:MGI (curated);Acc:Ltbp4-001] | 6 |
| 1372513\_at | Q6RUV5 | 363875 | ENSRNOG00000001068 | ENSRNOT00000060719  ENSRNOT00000001417 | Rac1 | Ras-related C3 botulinum toxin substrate 1 precursor (p21-Rac1). [Source:UniProtKB/Swiss-Prot;Acc:Q6RUV5] | 6 |
| 1399048\_at |  | 363026 | ENSRNOG00000031129 | ENSRNOT00000048245 | Prmt4 | Histone-arginine methyltransferase CARM1 (EC 2.1.1.125) (EC 2.1.1.-) (Protein arginine N-methyltransferase 4) (Coactivator-associated arginine methyltransferase 1). [Source:UniProtKB/Swiss-Prot;Acc:Q4AE70] | 6 |
| 1373570\_at |  | 311671 | ENSRNOG00000028384 | ENSRNOT00000034915 | NP\_001101276.1 | aminopeptidase-like 1 [Source:RefSeq\_peptide;Acc:NP\_001101276] | 6 |
| 1388828\_at |  | 315617 | ENSRNOG00000017871 | ENSRNOT00000024240 | NP\_001101612.1 | SID1 transmembrane family, member 2 [Source:RefSeq\_peptide;Acc:NP\_001101612] | 6 |
| 1367499\_at |  | 311204 | ENSRNOG00000007732 | ENSRNOT00000010278 | NP\_001101218.1 | solute carrier family 35, member C1 [Source:RefSeq\_peptide;Acc:NP\_001101218] | 6 |
| 1388481\_at | P62859 | 50718  684733  689805  501931 | ENSRNOG00000030242  ENSRNOG00000029899  ENSRNOG00000033709  ENSRNOG00000018787 | ENSRNOT00000051208  ENSRNOT00000040690  ENSRNOT00000045955  ENSRNOT00000025380 | LOC684733  RGD1564194\_predicted | 40S ribosomal protein S28. [Source:UniProtKB/Swiss-Prot;Acc:P62859] | 6 |
| 1372404\_at |  | 366957 | ENSRNOG00000007350 | ENSRNOT00000009994 | Rac2 | RAS-related C3 botulinum substrate 2 [Source:RefSeq\_peptide;Acc:NP\_001008385] | 6 |
| 1371950\_at |  | 302422 | ENSRNOG00000002838 | ENSRNOT00000003872 | NP\_001100415.1 | PDZ domain containing 11 [Source:RefSeq\_peptide;Acc:NP\_001100415] | 6 |
| 1389189\_at | Q9Z1P2 | 81634 | ENSRNOG00000004309 | ENSRNOT00000005990 | Actn1\_v3 | Alpha-actinin-1 (Alpha-actinin cytoskeletal isoform) (Non-muscle alpha-actinin-1) (F-actin cross-linking protein). [Source:UniProtKB/Swiss-Prot;Acc:Q9Z1P2] | 6 |
| 1373103\_at |  | 361724 | ENSRNOG00000019913 | ENSRNOT00000027141 | Mta2 | Mta2 protein (Fragment). [Source:UniProtKB/TrEMBL;Acc:Q4FZS6] | 6 |
| 1387942\_at | Q5RKL7 | 266687 | ENSRNOG00000004168 | ENSRNOT00000005646 | Slc35e4 | Solute carrier family 35 member E4. [Source:UniProtKB/Swiss-Prot;Acc:Q5RKL7] | 6 |
| 1372784\_at | Q5M9H1 | 362566 | ENSRNOG00000013642 | ENSRNOT00000018506 | Lrrc41 | Leucine-rich repeat-containing protein 41. [Source:UniProtKB/Swiss-Prot;Acc:Q5M9H1] | 6 |
| 1389660\_at | Q80ZD5 | 316003 | ENSRNOG00000019739 | ENSRNOT00000026740 | Amigo3 | Amphoterin-induced protein 3 precursor (AMIGO-3) (Alivin-3). [Source:UniProtKB/Swiss-Prot;Acc:Q80ZD5] | 6 |
| 1368010\_at | P81718 | 116689 | ENSRNOG00000014294 | ENSRNOT00000019462 | Ptpn6 | Tyrosine-protein phosphatase non-receptor type 6 (EC 3.1.3.48) (Protein-tyrosine phosphatase SHP-1). [Source:UniProtKB/Swiss-Prot;Acc:P81718] | 6 |
| 1372511\_at | P60486 | 300235 | ENSRNOG00000004628 | ENSRNOT00000055372  ENSRNOT00000006239  ENSRNOT00000055371 | Dazap2 | DAZ-associated protein 2 (Deleted in azoospermia-associated protein 2). [Source:UniProtKB/Swiss-Prot;Acc:P60486] | 6 |
| 1383945\_at |  | 304944  171570 | ENSRNOG00000003917 | ENSRNOT00000005213 | Uck2 | uridine-cytidine kinase 2 [Source:RefSeq\_peptide;Acc:NP\_001095878] | 6 |
| 1374110\_at |  |  | ENSRNOG00000012055 | ENSRNOT00000016281 | Man2a2 | mannosidase 2, alpha 2 Gene [Source:MGI Symbol;Acc:MGI:2150656] | 6 |
| 1398953\_at |  | 300036 | ENSRNOG00000009020 | ENSRNOT00000012306 | Tsta3\_predicted |  | 6 |
| 1372014\_at |  | 296612 | ENSRNOG00000013933 | ENSRNOT00000019157 | NP\_001100035.1 | similar to cofactor required for Sp1 transcriptional activation subunit 8 (predicted) (RGD1564993\_predicted), mRNA [Source:RefSeq\_dna;Acc:NM\_001106565] | 6 |
| 1386890\_at | P05943 | 81778 | ENSRNOG00000023226 | ENSRNOT00000035240 | S100a10 | Protein S100-A10 (S100 calcium-binding protein A10) (Calpactin-1 light chain) (Calpactin I light chain) (p10 protein) (p11) (Cellular ligand of annexin II) (Nerve growth factor-induced protein 42C). [Source:UniProtKB/Swiss-Prot;Acc:P05943] | 6 |
| 1380285\_at | Q63148 | 117275 | ENSRNOG00000001750 | ENSRNOT00000002394 | Chrd | Chordin precursor. [Source:UniProtKB/Swiss-Prot;Acc:Q63148] | 6 |
| 1395279\_at | Q4FZZ1 | 306203 | ENSRNOG00000008024 | ENSRNOT00000044321  ENSRNOT00000010763 | Pxk | PX domain-containing protein kinase-like protein (Modulator of Na,K- ATPase) (MONaKA). [Source:UniProtKB/Swiss-Prot;Acc:Q4FZZ1] | 6 |
| 1398245\_at | Q63544 | 64347 | ENSRNOG00000010437 | ENSRNOT00000014149 | Sncg | Gamma-synuclein (Persyn) (Sensory neuron synuclein). [Source:UniProtKB/Swiss-Prot;Acc:Q63544] | 6 |
| 1370368\_at |  |  | ENSRNOG00000001237 | ENSRNOT00000058253 | Cabin1 | Calcineurin-binding protein Cabin 1 (Calcineurin inhibitor) (CAIN). [Source:UniProtKB/Swiss-Prot;Acc:O88480] | 6 |
| 1370613\_s\_at | Q64634  Q64633  P08430  Q64637  P20720 | 301595  396552  154516  113992  396551  396527  574523 | ENSRNOG00000018740 | ENSRNOT00000025291  ENSRNOT00000025652  ENSRNOT00000032634  ENSRNOT00000045163  ENSRNOT00000044308 | Ugt1a3 | UDP-glucuronosyltransferase 1-1 precursor (EC 2.4.1.17) (UDPGT) (UGT1\*1) (UGT1-01) (UGT1.1) (UGT1A1) (B1). [Source:UniProtKB/Swiss-Prot;Acc:Q64550] | 6 |
| 1374310\_at | Q641Y6 | 295341 | ENSRNOG00000012481 | ENSRNOT00000016833 | Ppm1j | Protein phosphatase 1J (EC 3.1.3.16) (Protein phosphatase 2C isoform zeta) (PP2C-zeta). [Source:UniProtKB/Swiss-Prot;Acc:Q641Y6] | 6 |
| 1395642\_at |  | 313744 | ENSRNOG00000010109 | ENSRNOT00000013535 | Nol9 | Nol9 protein (Fragment). [Source:UniProtKB/TrEMBL;Acc:Q4G021] | 6 |
| 1369631\_at |  | 65261 | ENSRNOG00000004072 | ENSRNOT00000036666 | Myo1c | myosin IC [Source:RefSeq\_peptide;Acc:NP\_075580] | 6 |
| 1383917\_at |  | 303476 | ENSRNOG00000005575 | ENSRNOT00000007897 | Abi3 | ABI gene family, member 3 [Source:RefSeq\_peptide;Acc:NP\_001013136] | 6 |
| 1388457\_at | Q6P777 | 290635 | ENSRNOG00000017949 | ENSRNOT00000024413 | MGC72581 | Protein FAM125A. [Source:UniProtKB/Swiss-Prot;Acc:Q6P777] | 6 |
| 1374364\_at |  | 498703 | ENSRNOG00000039807 | ENSRNOT00000017349 | RGD1562335\_predicted |  | 6 |
| 1371724\_at | Q5U1X1 | 300689 | ENSRNOG00000018939 | ENSRNOT00000047633 | Rexo2 | Oligoribonuclease, mitochondrial precursor (EC 3.1.-.-) (Small fragment nuclease) (RNA exonuclease 2 homolog). [Source:UniProtKB/Swiss-Prot;Acc:Q5U1X1] | 6 |
| 1384771\_at |  | 287564 | ENSRNOG00000007455 | ENSRNOT00000055999 | RGD1565522\_predicted |  | 6 |
| 1372661\_at |  | 287120 | ENSRNOG00000013429 | ENSRNOT00000018405 | Tbl3 | transducin (beta)-like 3 [Source:RefSeq\_peptide;Acc:NP\_001008278] | 6 |
| 1367646\_at | P00787 | 64529 | ENSRNOG00000010331 | ENSRNOT00000014177 | Ctsb | Cathepsin B precursor (EC 3.4.22.1) (Cathepsin B1) (RSG-2) [Contains: Cathepsin B light chain; Cathepsin B heavy chain]. [Source:UniProtKB/Swiss-Prot;Acc:P00787] | 6 |
| 1382043\_at |  | 361689 | ENSRNOG00000017703 | ENSRNOT00000024018 | NP\_001101983.1 | unc-93 homolog B1 [Source:RefSeq\_peptide;Acc:NP\_001101983] | 6 |
| 1376362\_at |  |  | ENSRNOG00000016156 | ENSRNOT00000004703 | Nptxr | Neuronal pentraxin receptor. [Source:UniProtKB/Swiss-Prot;Acc:O35764] | 6 |
| 1387545\_at | Q9Z2Q4 | 81522 | ENSRNOG00000017593 | ENSRNOT00000023974 | Mtr | Methionine synthase (EC 2.1.1.13) (5-methyltetrahydrofolate-- homocysteine methyltransferase) (Vitamin-B12 dependent methionine synthase) (MS). [Source:UniProtKB/Swiss-Prot;Acc:Q9Z2Q4] | 6 |
| 1372491\_at |  | 313757 | ENSRNOG00000015007 | ENSRNOT00000020560 | RGD1565591\_predicted |  | 6 |
| 1383509\_at | Q4V887 | 291733 | ENSRNOG00000028703 | ENSRNOT00000036306 | Slc39a6 | Zinc transporter ZIP6 precursor (Zrt- and Irt-like protein 6) (ZIP-6) (Solute carrier family 39 member 6). [Source:UniProtKB/Swiss-Prot;Acc:Q4V887] | 6 |
| 1373366\_at |  | 293721 | ENSRNOG00000019724 | ENSRNOT00000026794 | NP\_001099804.1 | alpha glucosidase 2 alpha neutral subunit [Source:RefSeq\_peptide;Acc:NP\_001099804] | 6 |
| 1387062\_a\_at | Q91ZN7 | 140583 | ENSRNOG00000008181 | ENSRNOT00000011226 | Chek1 | Serine/threonine-protein kinase Chk1 (EC 2.7.11.1). [Source:UniProtKB/Swiss-Prot;Acc:Q91ZN7] | 6 |
| 1368460\_at | P43427 | 65197 | ENSRNOG00000017693 | ENSRNOT00000024054 | Slc2a5 | Solute carrier family 2, facilitated glucose transporter member 5 (Glucose transporter type 5, small intestine) (GLUT-5) (Fructose transporter). [Source:UniProtKB/Swiss-Prot;Acc:P43427] | 6 |
| 1390455\_at |  | 293050 | ENSRNOG00000017120 | ENSRNOT00000023506 | NP\_001099745.1 | abhydrolase domain containing 2 [Source:RefSeq\_peptide;Acc:NP\_001099745] | 6 |
| 1388740\_at |  | 309186 | ENSRNOG00000021161 | ENSRNOT00000028737 | RGD1310168\_predicted |  | 6 |
| 1394940\_at |  | 300870 | ENSRNOG00000010240 | ENSRNOT00000056937  ENSRNOT00000013586 | NP\_001100314.1 |  | 6 |
| 1367954\_at | Q62997 | 25454 | ENSRNOG00000017438 | ENSRNOT00000023667  ENSRNOT00000051709 | Gfra1 | GDNF family receptor alpha-1 precursor (GFR-alpha-1) (GDNF receptor alpha) (GDNFR-alpha) (TGF-beta-related neurotrophic factor receptor 1) (RET ligand 1). [Source:UniProtKB/Swiss-Prot;Acc:Q62997] | 6 |
| 1368121\_at | P38918 | 26760 | ENSRNOG00000017899 | ENSRNOT00000024160 | Akr7a3 | Aflatoxin B1 aldehyde reductase member 3 (EC 1.-.-.-) (AFB1-AR) (rAFAR1) (Aflatoxin B1 aldehyde reductase member 1). [Source:UniProtKB/Swiss-Prot;Acc:P38918] | 6 |
[truncated: 338,197 more chars]
