# Supplementary material for: Transcriptional regulation of gene expression clusters in motor neurons following spinal cord injury
Source: BMC Genomics. 2010 Jun 9;11:365. doi: 10.1186/1471-2164-11-365 (PMC2900267; doi:10.1186/1471-2164-11-365)
Supplement: Additional file 4 — R scripts example. Text file illustrating an example of how to run scripts (details on ClustreLustre and ASAP usage is described in original papers) [file 1471-2164-11-365-S4.RTF]

Example of ClustreLustre and ASAP analysis on micorarray data set combined with R and bioconductor packages.Require pre-installed bioconductor packages: affy, biomaRt, annotate, cluster, pls, amap Example run in R## source the R scripts, e.g. > source(".../xxx.R")## Read in data and calculate expression values (AffyBatch object and Exprs object)> data <- ReadAffy()> eset.data <- RMA(data)## annotate genes on microarray and create an annotation object for later annotation retrieval. Requires internet access and might take quite some time to run (overnight)! chip.type depends on the tip of Affymetrix type used and must be specified according to biomaRt annotation. > ann.obj <- Annotate(data, chip.type="affy_rat230_2")## calculate differentially expressed genes (DEG) using limma, samr, Cyber-T or other method of choice providing a matrix of gene names and adjusted p-values for a given FDR (DEG-matrix)… ## Define class labels and class names of the microarray experiments > sampleNames(eset.data)"chip1.condition1.cel" "chip2.condition1.cel" "chip3.condition1.cel""chip4.condition2.cel""chip5.condition2.cel" "chip6.condition2.cel" "chip7.condition3.cel" "chip8.condition3.cel"> class.labels <- cbind(class.labels, c(rep("condition1",3), rep("condition2", 3), rep("condition1", 2),))>  class.names <- unique(class.labels[,2])## condition could also be time, e.g. time-0, time-1, time-2….ClustreLustre## export DEG genes for ClustreLustre analysis> Clustre.Lustre.Export(eset.data,  rownames(DEG.matrix))## run ClustreLustre MatLab script on export TAB delimited file… Move the ClustreLustre output file to the R working directory and import the output> clusters.DEG  <- CL.import()## plot heatmap of each cluster, in the below example K was set to 12 in the ClustreLustre giving 12 clusters (eset.is.log=TRUE if working with RMA expression values(on log2 scale), otherwise set to set.is.log=FALSE). > for(i in 1:12) plot.cluster(eset.data, class.labels, class.names, eset.is.log=T, ClustreLustre=T, cl.obj=clusters.DEG, h.clust=F,heatmap=T,div.clust=F,pca.clust=F, cl.cluster=i)## Export gene IDs and their annotation sorted according to clusters. > gene.html.export(clusters.DEG, ann.obj, file.name="gene-list-RMA-anova-fdr2.cl1of12.html")ASAP promoter analysis## extract promoter sequences for the DE genes using biomaRt. In this case there were 12 cluster, so they are exported in separate files. > for(i in 1:12) extract.biomart.sequences(clusters.DEG, ann.obj, filter="ensembl_gene_id", seqType="coding_gene_flank", downstream=200, upstream=1000,filter.genes=T, cluster=i)## background sequence set of all DE genes> extract.biomart.sequences(clusters.DEG, ann.obj, filter="ensembl_gene_id", seqType="coding_gene_flank", downstream=200, upstream=1000,filter.genes=T, file.name="sequences_BG.fasta")## Run ASAP python script on fasta files for each cluster with sequences_BG.fasta as reference/background and merge result into a comma separated matrix ("asap_clusters.csv") containing as the first column all the transcription factor (TF) names, and in all subsequent columns the corresponding z-scores of the ASAP output. > TF.stats <- read.csv("asap_clusters.csv", header=F)## organize TF.stats such that row names are the transcription factors, and columns are names for the clusters ("Cluster 1", "Cluster 2", "Cluster 3,…) for column 1,2,3,…> plot.cluster(TF.stats, heatmap=T, h.clust=F, pca.clust=T, colv=NULL, method.linkage="correlation", scaleH="col")> plot.cluster(TF.stats, heatmap=T, h.clust=F, pca.clust=T, colv=NULL, method.dist="euclidian", scaleH="col")## or>  heatmap(as.matrix(TF.stats), scale="col",col=rgcolors.func(100))## possibly do manual normalization of z-score vectors of each cluster setting it to have a variance of 1 (again this example as 12 clusters). > TF.stats.sd <- apply(TF.stats,2,sd)> TF.stats.norm <- TF.stats> for(i in 1:12) TF.stats.norm[,i] <-TF.stats[,i]/TF.stats.sd[i] ## run cluster plot on TF.stats.sd> plot.cluster(TF.stats.norm, heatmap=T, h.clust=F, pca.clust=T, colv=NULL, method.linkage="correlation", scaleH="col")## plot separation of transcription factor (for which there was ASAP data) expression patterns contained in each cluster > plot.cluster(rma.data, heatmap=T, h.clust=T, pca.clust=T, pc.ids="numeric", class.names=class.names, class.labels=class.labels, gene.subset=T, top.genes= TF.stats[,1], eset.is.log=T)
